# Anion-controlled ion pairing and assembly of π-electronic cations with orthogonal π-systems

**DOI:** 10.1039/d6sc05424b

**Published:** 2026-08-17

**Authors:** Yohei Haketa, Tomoka Inoue, Taichi Abiko, Shunsuke Kobashi, Ryota Sato, Yoichi Kobayashi, Kotaro Matsumoto, Tomoyuki Hamachi, Gaku Fukuhara, Tatsuki Morimoto, Rio Kugizaki, Hiromitsu Maeda

**Affiliations:** a Department of Applied Chemistry, College of Life Sciences, Ritsumeikan University Kusatsu 525-8577 Japan maedahir@ph.ritsumei.ac.jp ykobayas@fc.ritsumei.ac.jp; b Department of Chemistry, Institute of Science Tokyo Tokyo 152-8551 Japan; c Institute for Materials Chemistry and Engineering, Kyushu University Fukuoka 819-0395 Japan gaku@ms.ifoc.kyushu-u.ac.jp; d Department of Applied Chemistry, School of Engineering, Tokyo University of Technology Hachioji 192-0982 Japan

## Abstract

Anion-responsive dipyrrolyldiketone and phenalenyl units covalently linked by a spiroboronium unit form π-electronic cations with orthogonally arranged π-systems. Conformations of the anion-binding unit are dependent on the coexisting counteranions in the solution and solid states. Orthogonally arranged π-electronic cations afford solid-state ion-pairing assemblies *via* double ^*i*^π–^*i*^π interactions at the dipyrrolyldiketone anion complex and the phenalenyl units. Transient absorption spectra revealed electron transfer from anion-binding π-units to orthogonally arranged π-electronic cations, which could be modulated by the coexisting counteranions. Orthogonally arranged π-electronic cations exhibit pressure-responsive photophysical properties.

## Abstract 2

Anion-responsive π-electronic cations with orthogonal π-systems undergo counteranion-dependent conformational changes, forming ion-pairing assemblies, and modulation of photoinduced electron transfer and pressure-responsive photophysical properties.

## Graphical abstract

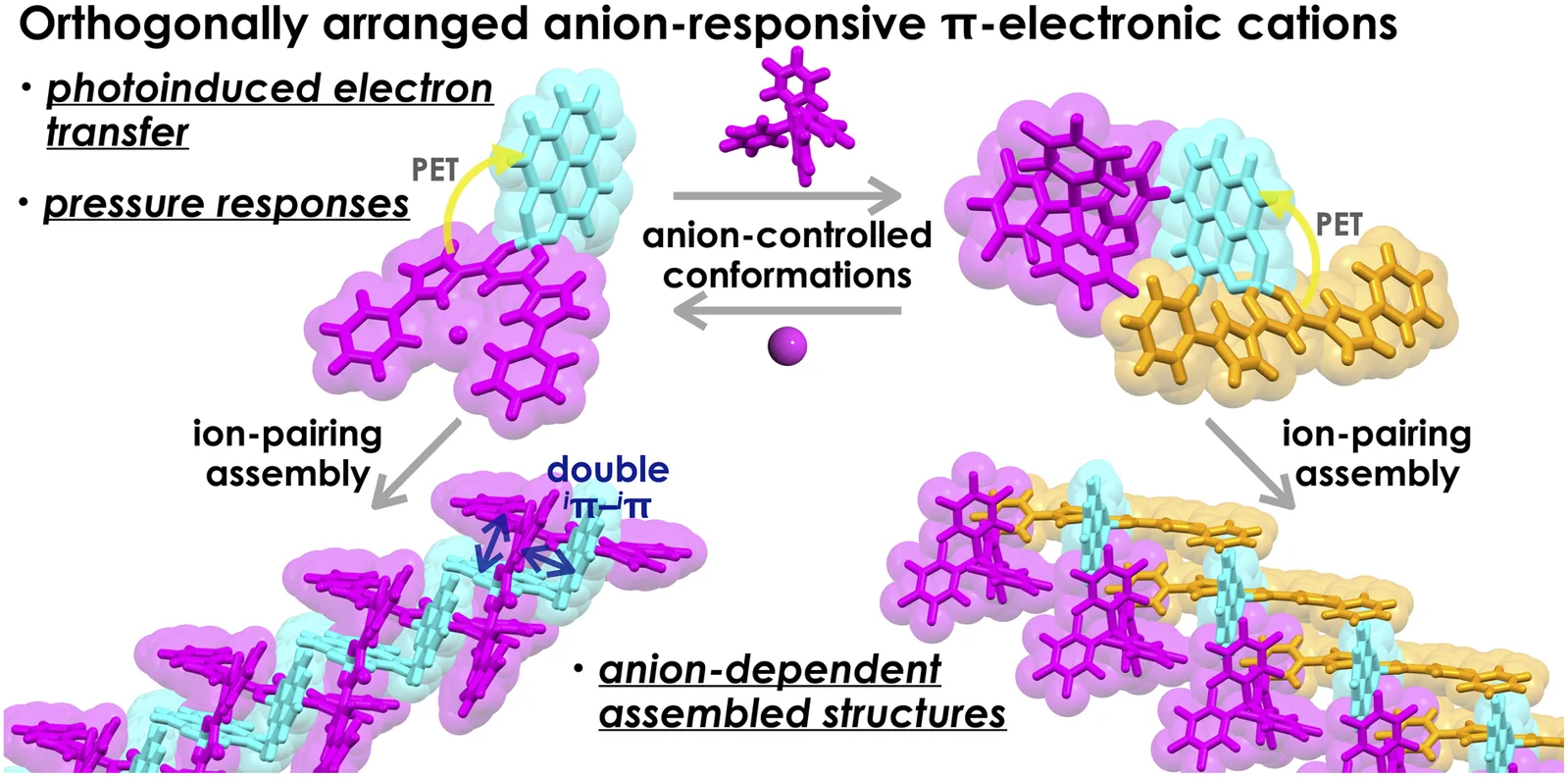

## Introduction

**Fig. 1:**
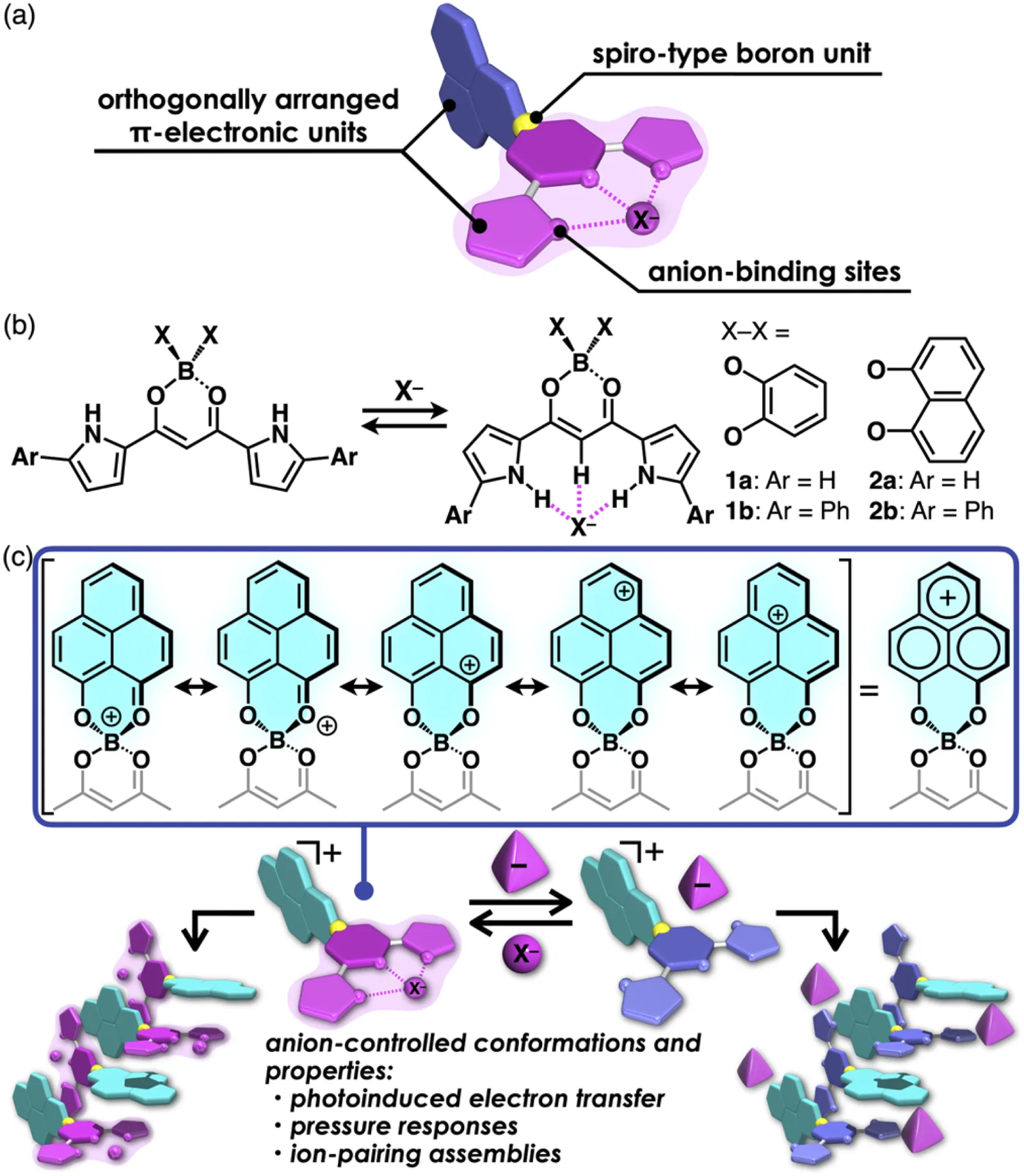
(a) Conceptual diagram of anion complexes (pseudo-π-electronic anions) of boron-spiro-centred anion-responsive molecules, (b) anion complexation of dipyrrolyldiketone boron complexes 1a,b and 2a,b and (c) representative resonance structures of cationic 9-oxidophenalenone-modified 1,3-diketone-boron complexes (the coordination mode around boron is illustrated to emphasize the stereochemistry) (top) and conceptual diagram of anion-dependent conformation changes and their ion-pairing assemblies (bottom).

Appropriately arranged π-electronic systems comprising two different electronic states with electron-donating and electron-accepting properties show fascinating electronic and photophysical properties.^1^ In particular, a rigid orthogonal arrangement of two π-electronic systems induces minimized electronic interactions, which have been used for photoinduced electron transfer (PET).^2^ For example, an orthogonally arranged mesitylmethylacridinium cation shows efficient photoredox catalytic behaviours that can be initiated by PET using visible light.^3^ Boron-spiro-centred structures of aryldiols provide orthogonally arranged monoanionic π-electronic systems.^4^ π-Electronic ligands with an appropriate charge enable the preparation of tetracoordinated spiro-type boron complexes. Spiro-type boron complexes comprising 1,3-diketonate and aryldiolate afford electronically neutral orthogonally arranged π-electronic systems (Fig. 1a). For example, dipyrrolyldiketonate-catecholate boron complexes 1a,b and 2a,b show anion-binding abilities to form planar anion complexes as pseudo-π-electronic anions for ion-pairing assemblies (Fig. 1b).^5^ Extension of π-conjugation through the introduction of naphthalenediolate facilitates the formation of diverse ion-pairing assemblies driven by stacking at both orthogonally arranged π-units.^5*c*^ Anion-binding π-electronic units can act as electron donors depending on the energy levels of potential electron-accepting counterparts.^6^ Thus, modulations of the electronic states of aryldiolate units would induce PET behaviours. Given these points, boron complexation by 1,3-diketones and 9-oxidophenalenone^7,8^ can provide a positive charge delocalized at the boron and phenalenyl units, resulting in electron-deficient cationic π-electronic systems including a spiroboronium structure (Fig. 1c top). This study includes the synthesis of anion-responsive π-electronic cations with orthogonally arranged π-systems that exhibit anion-controlled electronic and photophysical properties and ion-pairing assembly (Fig. 1c bottom). These findings provide a new design principle for charged π-electronic systems through ion pairing and assembly.

## Results and discussion

### Synthesis and characterization of orthogonally arranged π-electronic cations

**Fig. 2:**
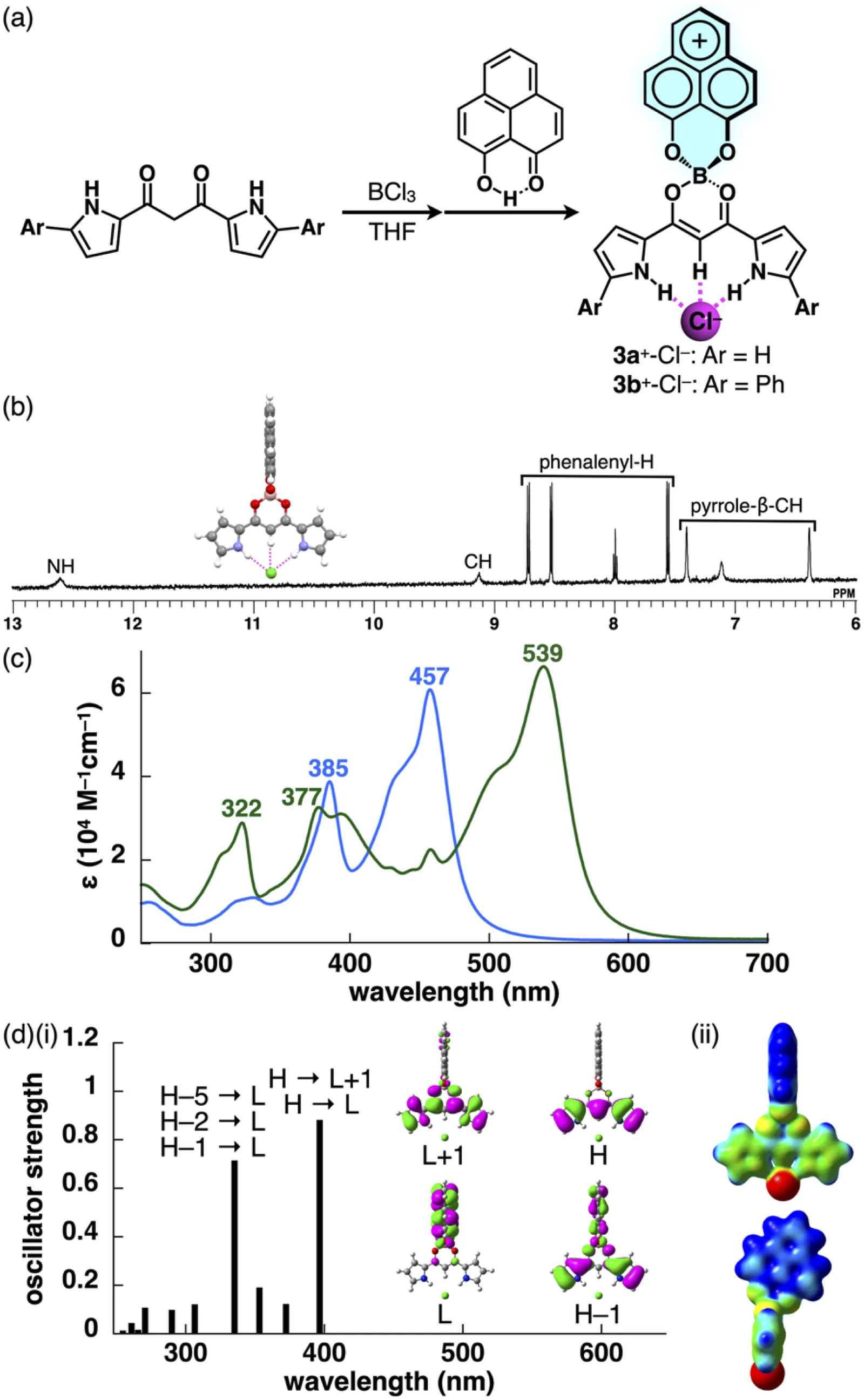
(a) Synthesis of orthogonally arranged anion-responsive π-electronic cations as Cl^−^ ion pairs, (b) ^1^H NMR spectrum of 3a^+^-Cl^−^ in CD_2_Cl_2_ (0.25 mM) with the optimized structure calculated at CPCM-CAM-B3LYP/6-31++G(d,p) (CH_2_Cl_2_) (inset), (c) UV/vis absorption spectra of 3a^+^-Cl^−^ (blue) and 3b^+^-Cl^−^ (green) in CH_2_Cl_2_ (1 × 10^−5^ M) and (d)(i) TD-DFT-based UV/vis absorption stick spectra (inset: selected MOs) and (ii) top and side views of electrostatic potentials (ESP) mapped onto the electron density isosurfaces (*δ* = 0.01) of 3a^+^-Cl^−^ at CPCM-CAM-B3LYP/6-31++G(d,p) (CH_2_Cl_2_).

Boron complexation of 9-oxidophenalenone and dipyrrolyldiketones was achieved according to the previously reported synthetic method for boron complexation of aryldiols.^5^ Treatment of dipyrrolyldiketones with BCl_3_ in THF followed by the addition of 9-hydroxyphenalenone^7^ afforded cationic spiroboronium Cl^−^ ion pairs 3a^+^-Cl^−^ and 3b^+^-Cl^−^ as red solids in 26% and 24% yields, respectively (Fig. 2a). The obtained ion pairs were characterized by ^1^H NMR and ESI-TOF-MS. The ^1^H NMR spectrum of 3a^+^-Cl^−^ in CD_2_Cl_2_ (0.25 mM) showed the signals at 12.61 and 9.13 ppm for NH and bridging CH, respectively (Fig. 2b and S75a(i)). The signals appeared downfield relative to 1,^5*a*^ indicating pyrrole inversion upon Cl^−^ binding through hydrogen bonding. Similarly, 3b^+^-Cl^−^ exhibited NH and bridging CH signals at 12.66 and 9.55 ppm, respectively, in the pyrrole-inverted anion-binding form (Fig. S75b(i)). ^11^B NMR of 3a^+^-Cl^−^ and 3b^+^-Cl^−^ showed signals at 3.92 and 3.87 ppm, respectively (Fig. S76a(i) and b(i)).^9^ Optimized structures for 3a^+^-Cl^−^ and 3b^+^-Cl^−^ calculated at the CPCM-CAM-B3LYP/6-31++G(d,p) (CH_2_Cl_2_) level showed that the phenalenyl unit was arranged orthogonally to the receptor units (Fig. 2b inset, S35 and S36).^10^ The Cl^−^ ion pairs 3a^+^-Cl^−^ and 3b^+^-Cl^−^ exhibited pyrrole-inverted Cl^−^-binding structures that are more stable by 3.00 and 4.31 kcal mol^−1^, respectively, than the corresponding pyrrole-uninverted structures with Cl^−^ interacting with a single pyrrole NH site. UV/vis absorption spectra of 3a^+^-Cl^−^ and 3b^+^-Cl^−^ in CH_2_Cl_2_ (1 × 10^−5^ M) showed maxima (*λ*_max_) at 457 and 539 nm, respectively (Fig. 2c and S10), suggesting the extension of the π-conjugation by the introduction of phenyl units. TD-DFT calculations at the CPCM-CAM-B3LYP/6-31++G(d,p) (CH_2_Cl_2_) level revealed that both the major absorption bands of the Cl^−^ complexes were mainly assigned to the HOMO → LUMO+1 and HOMO → LUMO transitions (Fig. 2d and S56). Notably, the orthogonal arrangement of the phenalenyl and Cl^−^-complexing units in 3a^+^-Cl^−^ and 3b^+^-Cl^−^ resulted in pseudo-zwitterionic structures. Electrostatic potential mapping (ESP) based on the optimized structures at the CPCM-CAM-B3LYP/6-31++G(d,p) (CH_2_Cl_2_) level clearly revealed electron-deficient phenalenyl and electron-rich Cl^−^ complexing units (Fig. 2d(ii), S37 and S38).

**Fig. 3:**
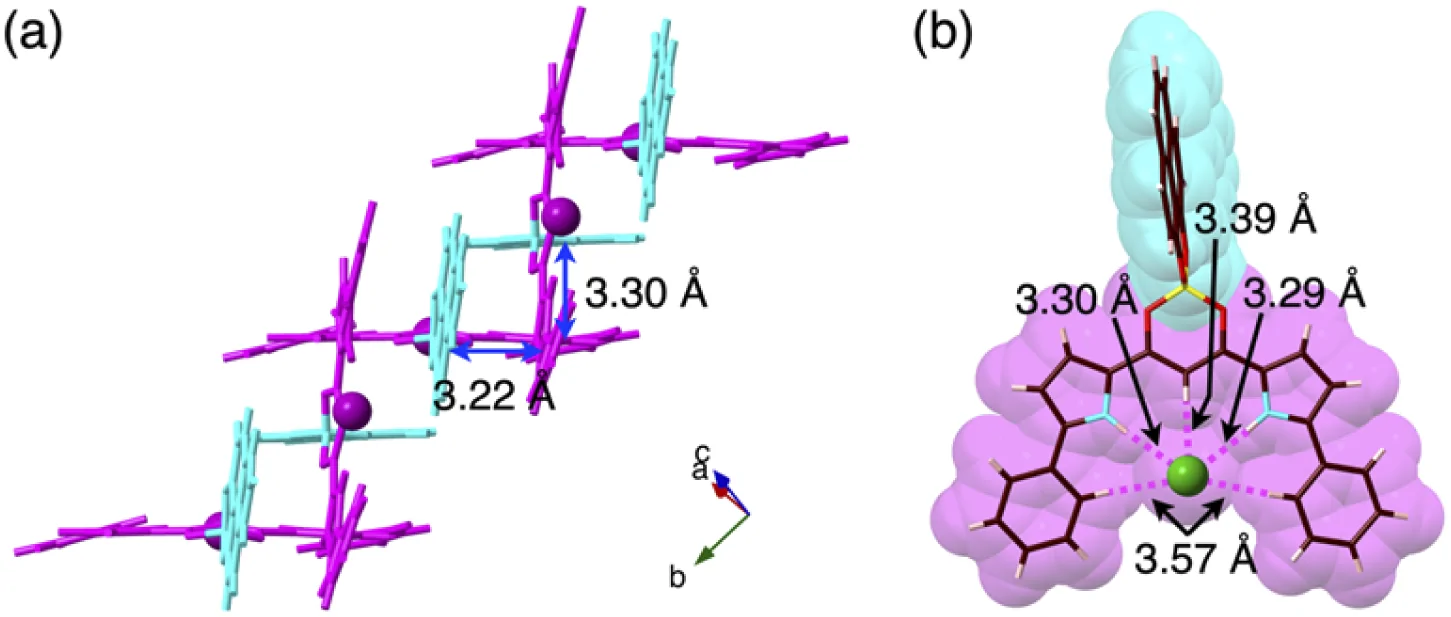
Single-crystal X-ray structure of 3b^+^-Cl^−^ as (a) packing structure and (b) ion pair (anion complex). Colour code (also applicable to the following figures) in (a) and (b): cyan and magenta refer to 9-oxidophenalenone-boron complex and receptor–Cl^−^ complex unit, respectively. Atom colour code in (b): brown, pink, yellow, blue, red and green (spherical) refer to carbon, hydrogen, boron, nitrogen, oxygen and chlorine, respectively.

The exact structure of 3b^+^-Cl^−^ was revealed by X-ray analysis of a single crystal obtained by vapour diffusion of *n*-hexane into a CH_2_Cl_2_/MeOH/C_6_H_5_CN mixed solution. As seen in 3b^+^-Cl^−^ in solution, the solid-state ion pair 3b^+^-Cl^−^ formed a doubly pyrrole-inverted conformation by hydrogen bonding with distances of 3.29/3.30, 3.39 and 3.57 Å for N(–H)⋯Cl^−^, C(–H)⋯Cl^−^ and phenyl-*o*-C(–H)⋯Cl^−^, respectively (Fig. 3 and S23). The planar Cl^−^ complex unit was stacked with proximally located phenalenyl unit with the stacking distances of 3.22 and 3.30 Å, forming a 1D-array structure through double ^*i*^π–^*i*^π interactions.^11^ Hirshfeld surface analysis clearly visualized the stacking structure, showing a bowtie arrangement of red and blue triangles in the shape-index property and flat regions in the curvedness property (Fig. S31).^12^ Energy decomposition analysis (EDA) calculations were performed to obtain the details for the interaction energy of the crystal packing structure.^13–16^ EDA calculations based on FMO2-MP2/cc-pVDZ for the proximally located stacking structure of dipyrrolyldiketone Cl^−^ complex and phenalenyl units in the packing structure of 3b^+^-Cl^−^ showed a large negative *E*_tot_ of −73.52 kcal mol^−1^, with favourable contributions from *E*_es_ and *E*_disp_ of −36.22 and −58.23 kcal mol^−1^, respectively, arising from double ^*i*^π–^*i*^π interactions (Fig. S71). The two stacked parts between the orthogonally arranged pseudo-zwitterionic structures for double ^*i*^π–^*i*^π interactions afforded ion-pairing assemblies with large interaction energies.

**Fig. 4:**
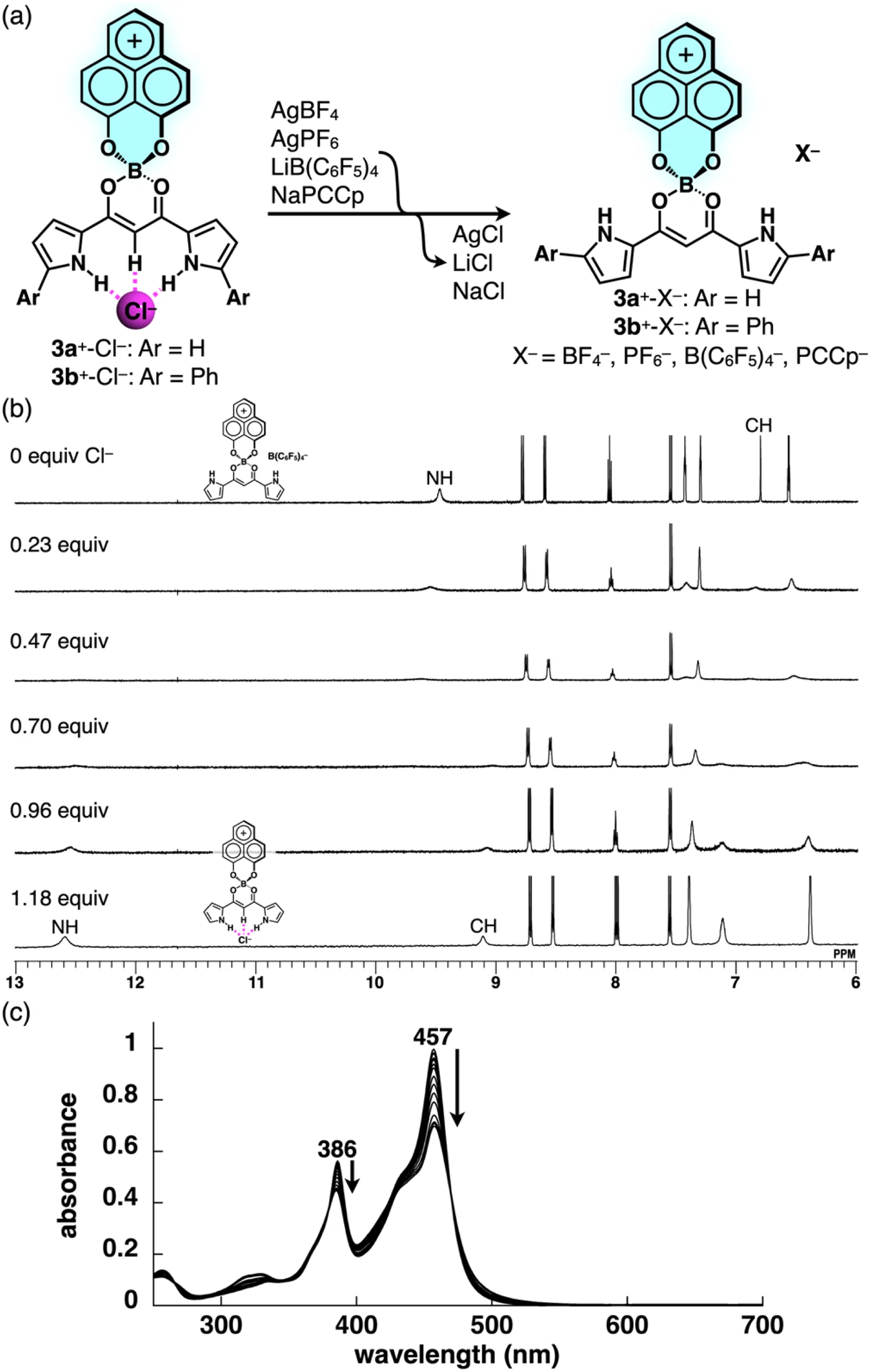
(a) Ion-pair metathesis of orthogonally arranged anion-responsive π-electronic cations as ion pairs with counteranions, (b) ^1^H NMR spectral changes observed for 3a^+^-B(C_6_F_5_)_4_^−^ upon the addition of TBACl in CD_2_Cl_2_ (1 × 10^−3^ M) and (c) UV/vis absorption spectral changes of 3a^+^-B(C_6_F_5_)_4_^−^ upon the addition of 2 equiv. of TBACl in CH_2_Cl_2_ (1.2 × 10^−5^ M).

Cl^−^ in 3a^+^-Cl^−^ and 3b^+^-Cl^−^ were further exchanged with other anions, such as BF_4_^−^, PF_6_^−^, B(C_6_F_5_)_4_^−^ and pentacyanocyclopentadienide (PCCp^−^),^17^ in 60–100% yield by ion-pair metathesis using the metal salts of desired anions (Fig. 4a). Ion pairs were purified through silica gel based on differences in anion-dependent polarities except for 3b^+^-BF_4_^−^, which could not be isolated because its *R*_f_ value was similar to that of 3b^+^-Cl^−^. In contrast to the downfield ^1^H NMR signals of 3a^+^-Cl^−^, the NH and bridging CH signals in ion pairs with larger anions appeared more upfield. For example, the NH and bridging CH signals in 3a^+^-B(C_6_F_5_)_4_^−^ were observed more upfield at 9.50 and 6.80 ppm, respectively, in CD_2_Cl_2_ (0.25 mM), suggesting the Cl^−^-free pyrrole-uninverted conformation (Fig. S75a(ii)). Similar ^1^H NMR signals were observed for 3b^+^-B(C_6_F_5_)_4_^−^ (Fig. S75b(ii)). ^11^B NMR signals of 3a^+^-B(C_6_F_5_)_4_^−^ and 3b^+^-B(C_6_F_5_)_4_^−^ were observed at 3.60 and 3.59 ppm, respectively, which are slightly shifted upfield relative to those of the Cl^−^ ion pairs (Fig. S76a(ii) and b(ii)).^9^ Ion pairs 3a^+^-X^−^ and 3b^+^-X^−^ (X^−^ = BF_4_^−^, PF_6_^−^, B(C_6_F_5_)_4_^−^ and PCCp^−^) (1 × 10^−5^ M) showed the *λ*_max_ values at 457 and 536 nm, respectively, similar to those of the corresponding Cl^−^ ion pairs, but with absorbances larger than those of the Cl^−^ ion pairs (Fig. S10). These differences were ascribed to the anion-binding forms with two pyrrole inversions for the Cl^−^ ion pairs, which was also supported by ^1^H NMR and UV/vis examinations. Cl^−^ binding of the B(C_6_F_5_)_4_^−^ ion pairs was elucidated by ^1^H NMR spectra upon the addition of Cl^−^ as a tetrabutylammonium (TBA) salt in CD_2_Cl_2_ (1 × 10^−3^ M). Upon the addition of Cl^−^ to 3a^+^-B(C_6_F_5_)_4_^−^, the signals of the pyrrole NH and bridging CH were gradually shifted to 12.60 and 9.12 ppm, respectively, suggesting the formation of a Cl^−^ complex with the inversion of two pyrrole rings (Fig. 4b and S77). The Cl^−^-binding behaviour of the B(C_6_F_5_)_4_^−^ ion pairs was also elucidated by UV/vis absorption spectral changes upon the addition of TBACl. Addition of 2 equiv. of TBACl in CH_2_Cl_2_ (1.2 × 10^−5^ M) to 3a^+^-B(C_6_F_5_)_4_^−^ afforded decreased absorbance at 386 and 457 nm in the UV/vis absorption spectrum, suggesting the formation of the Cl^−^ complex (Fig. 4c). TD-DFT calculations at the CPCM-CAM-B3LYP/6-31++G(d,p) (CH_2_Cl_2_) level also suggested a smaller oscillator strength for the pyrrole-inverted structures (Fig. S56 and S57). 3a^+^-B(C_6_F_5_)_4_^−^ and 3b^+^-B(C_6_F_5_)_4_^−^ provided extremely high binding constants (*K*_a_) of 3.8 × 10^7^ and 1.0 × 10^8^ M^−1^, respectively, for the formation of 3a^+^-Cl^−^ and 3b^+^-Cl^−^, respectively, suggesting effective Cl^−^ binding by these positively charged π-electronic systems (Fig. S79).

### Intramolecular photoinduced electron transfer

**Fig. 5:**
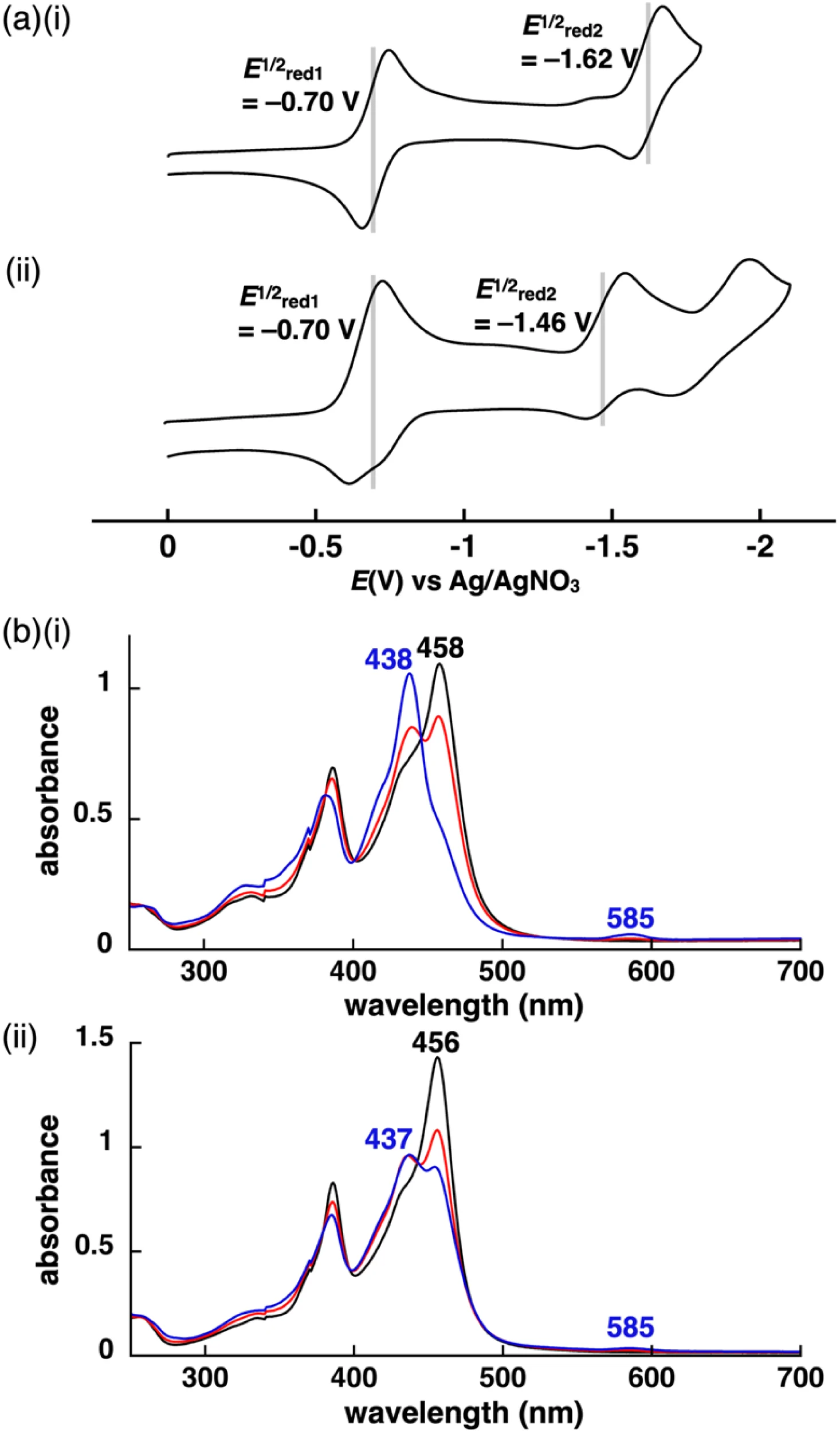
(a) Cyclic voltammograms of (i) 3a^+^-Cl^−^ and (ii) 3a^+^-PF_6_^−^ in CH_2_Cl_2_ (0.25 mM) containing TBACl and TBAPF_6_ (0.1 M for each), respectively, as electrolytes and (b) spectroelectrochemical analysis for UV/vis absorption spectral changes of (i) 3a^+^-Cl^−^ and (ii) 3a^+^-PF_6_^−^ for the oxidation states at −0.71/−0.70 V (red) and −0.91/−0.90 V (blue) (*versus* Ag/AgNO_3_), respectively, in CH_2_Cl_2_ (1.5 × 10^−4^ M) containing 0.1 M TBACl in (i) and TBAPF_6_ in (ii) as electrolytes under Ar atmosphere.

The orthogonal arrangement of the electron-rich and electron-deficient π-electronic systems linked by a spiroboronium unit results in intramolecular electron transfer. Electron-accepting behaviour was evaluated using cyclic voltammetry (CV). CV of the spiroboronium ion pairs 3a^+^-Cl^−^ and 3a^+^-PF_6_^−^ showed first and second reduction potentials (*E*_red1,2_^1/2^, *versus* Ag/AgNO_3_) of −0.70/−1.62 and −0.70/−1.46 V, respectively (Fig. 5a and S80a, b).^18^ These *E*_red1_^1/2^ are ascribed to a reduction in the phenalenyl unit. On the other hand, the reduced *E*_red2_^1/2^ for 3a^+^-Cl^−^ with the Cl^−^-binding form relative to 3a^+^-PF_6_^−^ with the anion-free form suggests that *E*_red2_^1/2^ is derived from the dipyrrolyldiketone unit. The α-phenyl-substituted ion pairs 3b^+^-Cl^−^ and 3b^+^-PF_6_^−^ exhibited similar reduction tendency (−0.71/−1.57 and −0.71/−1.41 V, respectively) (Fig. S80c and d). Spectroelectrochemical analysis upon one-electron reduction of 3a^+^-Cl^−^ exhibited decreased absorption at *λ*_max_ (458 nm) and increased absorption at 438 nm with slightly enhanced absorbance at 585 nm (Fig. 5b(i) and S81a). This observation suggests that the reduced phenalenyl unit influences the electronic state of the dipyrrolyldiketone unit *via* the bridging boron. 3a^+^-PF_6_^−^ also exhibited a decreased absorbance at 456 nm, with a less enhanced absorbance at 437 nm in the reduced state (Fig. 5b(ii) and S81b). 3b^+^-Cl^−^ and 3b^+^-PF_6_^−^ exhibited similar spectral changes upon one-electron reduction (Fig. S81c and d). TD-DFT calculations at CPCM-CAM-B3LYP/6-31++G(d,p) (CH_2_Cl_2_) level for one-electron-reduced state of the Cl^−^ ion pairs supported a blue shift of *λ*_max_ with increased absorbance and slightly enhanced absorbance at 585 nm (Fig. S57 and S62). The blue shifts of *λ*_max_ for the one-electron-reduced PF_6_^−^ ion pairs, which adopt pyrrole-uninverted conformations, were also supported by TD-DFT calculations (Fig. S58 and S63).

**Fig. 6:**
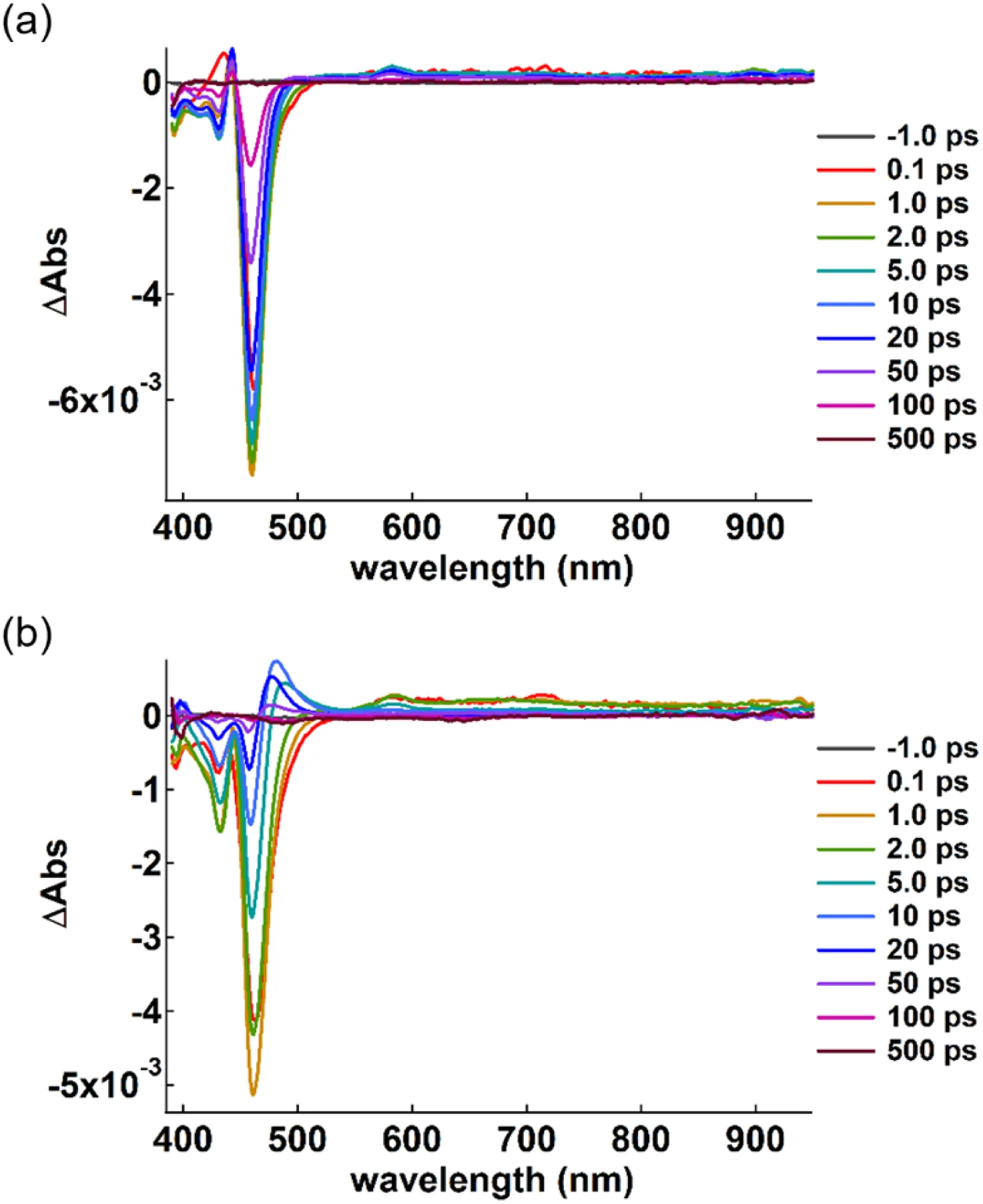
Transient absorption spectra of 3a^+^-B(C_6_F_5_)_4_^−^ in CH_2_Cl_2_ (5.0 × 10^−5^ M) (a) without and (b) with 2 equiv. of TBACl.

The redox behaviour observed for spiroboronium ion pairs can also be triggered by photoexcitation. To gain detailed insight into these processes, transient absorption measurements were performed using femtosecond laser pulses. For 3a^+^-B(C_6_F_5_)_4_^−^ in CH_2_Cl_2_, ground-state bleach (GSB) signals at 430 and 450 nm and positive transient absorption bands at approximately 520–900 nm were observed at 0.1 ps after photoexcitation (Fig. 6a and S82). Subsequently, other positive transient absorption bands appeared at 590 and 700 nm. The signals gradually decayed within 100 ps. In 3a^+^-B(C_6_F_5_)_4_^−^ with 2 equiv. of TBACl in CH_2_Cl_2_, in which 3a^+^-Cl^−^ was produced, positive transient absorption bands at 590 and 700 nm were more clearly observed, even instantaneously after excitation (Fig. 6b and S83). By contrast, positive signals from 590 to 900 nm decayed on a picosecond timescale, and another positive transient absorption was observed at 490 nm. These signals gradually decayed within tens of picoseconds.

The transient absorption band at 585 nm for 3a^+^-B(C_6_F_5_)_4_^−^ is similar to that of the absorption spectrum of 3a^+^-PF_6_^−^ at a constant voltage of −0.70 V (Fig. 5b(ii) and S81b). Moreover, similar but broader transient absorption bands were observed for 3b^+^-B(C_6_F_5_)_4_^−^ and 3b^+^-Cl^−^ (Fig. S84 and S85). Importantly, 1b does not exhibit a positive transient absorption band in the absence or presence of excess TBACl (Fig. S86 and S87). These results suggest that the observed transient absorption band can be ascribed to the reduced phenalenyl unit generated by photoinduced electron transfer from the dipyrrolyldiketone unit. Global analyses suggested that the time constants of the photoinduced electron transfer for 3b^+^-B(C_6_F_5_)_4_^−^ and 3b^+^-Cl^−^ were 200 fs and faster than the instrumental response function (150 fs), respectively (Fig. S85). The faster electron transfer and the pronounced radical anion feature may be due to the Cl^−^ binding to the dipyrrolyldiketone unit, which elevates the HOMO and facilitates electron transfer. On the other hand, the increase in the HOMO level by Cl^−^ binding and extended π-conjugation may also accelerate the back electron transfer, which explains the much longer lifetime of the radical anion of phenalenyl unit for 3a^+^-B(C_6_F_5_)_4_^−^ (64 ps) than others (0.8–3.6 ps).

### Pressure-responsive photophysical properties

**Fig. 7:**
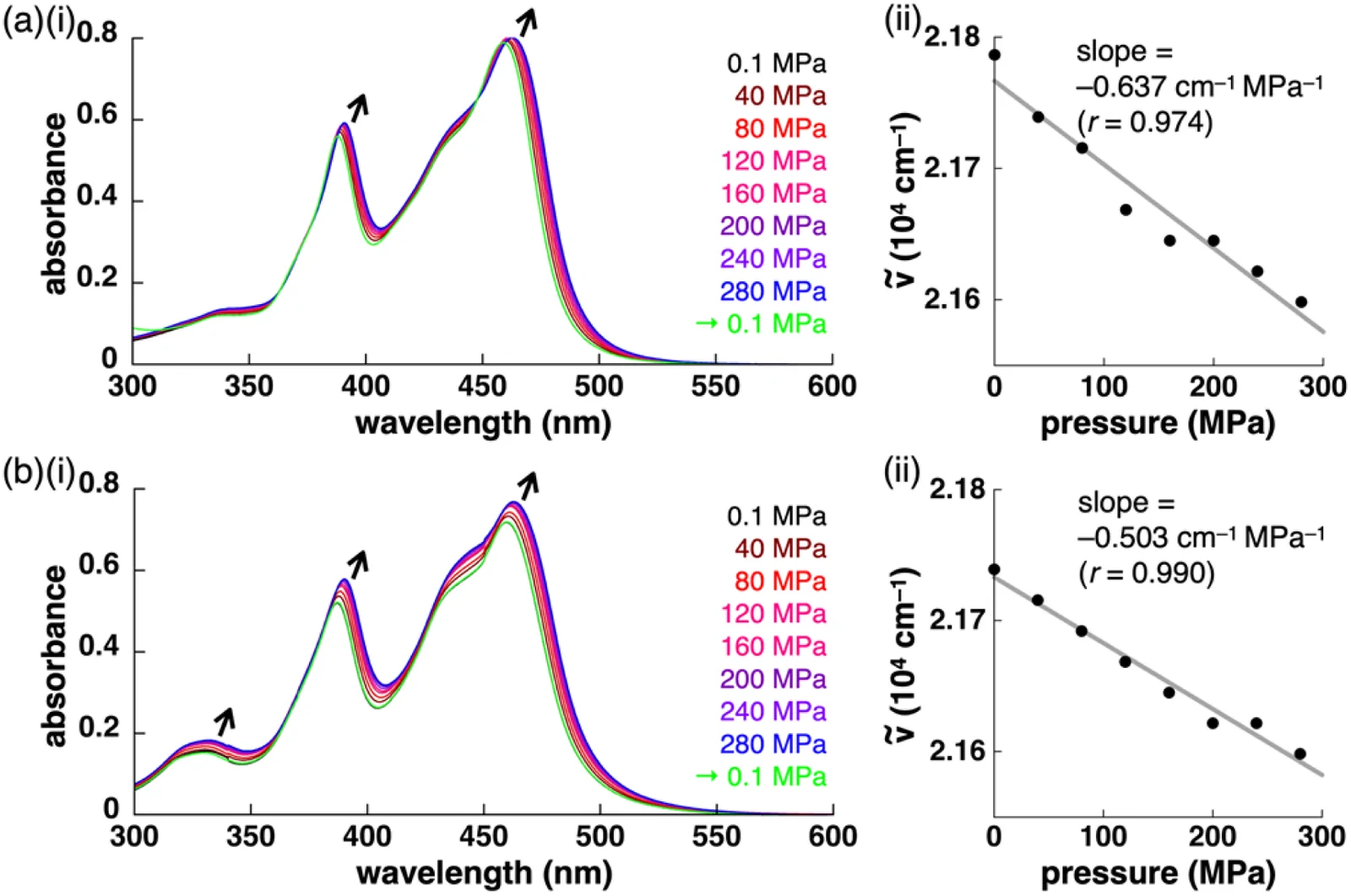
(i) Pressure-dependent UV/vis absorption spectra and (ii) plots of wavenumber changes for the absorption maxima of (a) 3a^+^-B(C_6_F_5_)_4_^−^ and (b) 3a^+^-Cl^−^ in CH_2_Cl_2_ (7.5 × 10^−5^ and 8.7 × 10^−5^ M, respectively).

Covalently linked π-electronic systems with large dihedral angles show pressure-responsive photophysical properties depending on the linker structures.^19^ Thus, pressure–responsive properties of the spiroboronium ion pairs were evaluated by monitoring UV/vis absorption spectral changes in CH_2_Cl_2_ at r.t. under elevated hydrostatic pressures (up to 280 MPa).^20^ Under increasing pressure, gradual red shifts with increased absorbances were observed for 3a^+^-B(C_6_F_5_)_4_^−^ and 3b^+^-B(C_6_F_5_)_4_^−^ (Fig. 7a and S89a, b). The original spectral profiles observed at 0.1 MPa were recovered upon returning the pressure to 0.1 MPa, indicating that the pressurization cycles were reversible. Such red shifts are presumably attributable to the changes in solvent polarizability and density under hydrostatic pressure,^21^ whereas the increased absorbances resulted from an effective increase in the concentration.^22^ Plots of the absorption maxima as a function of applied pressure exhibited a larger slope for 3b^+^-B(C_6_F_5_)_4_^−^ (−0.714 cm^−1^ MPa^−1^) than that for 3a^+^-B(C_6_F_5_)_4_^−^ (−0.637 cm^−1^ MPa^−1^), indicating a slightly stronger pressure response for the former.^23^3a^+^-Cl^−^ and 3b^+^-Cl^−^, prepared by the addition of 1 equiv. of TBACl to the B(C_6_F_5_)_4_^−^ ion pairs, also exhibited pressure-responsive UV/vis absorption behaviours (Fig. 7b and S89c, d). The slopes for the Cl^−^ ion pairs were −0.503 and −0.616 cm^−1^ MPa^−1^, respectively, which are smaller than those observed for the corresponding B(C_6_F_5_)_4_^−^ ion pairs. The smaller pressure response of the Cl^−^ ion pairs was attributed to the structural rigidification caused by Cl^−^ binding. Remarkably, anion-dependent conformational changes induce a characteristic pressure-responsive behaviour even though the spiroboronium ion pairs are sufficiently rigid to reduce the pressure responses.

### Solid-state anion-controlled ion-pairing assemblies

**Fig. 8:**
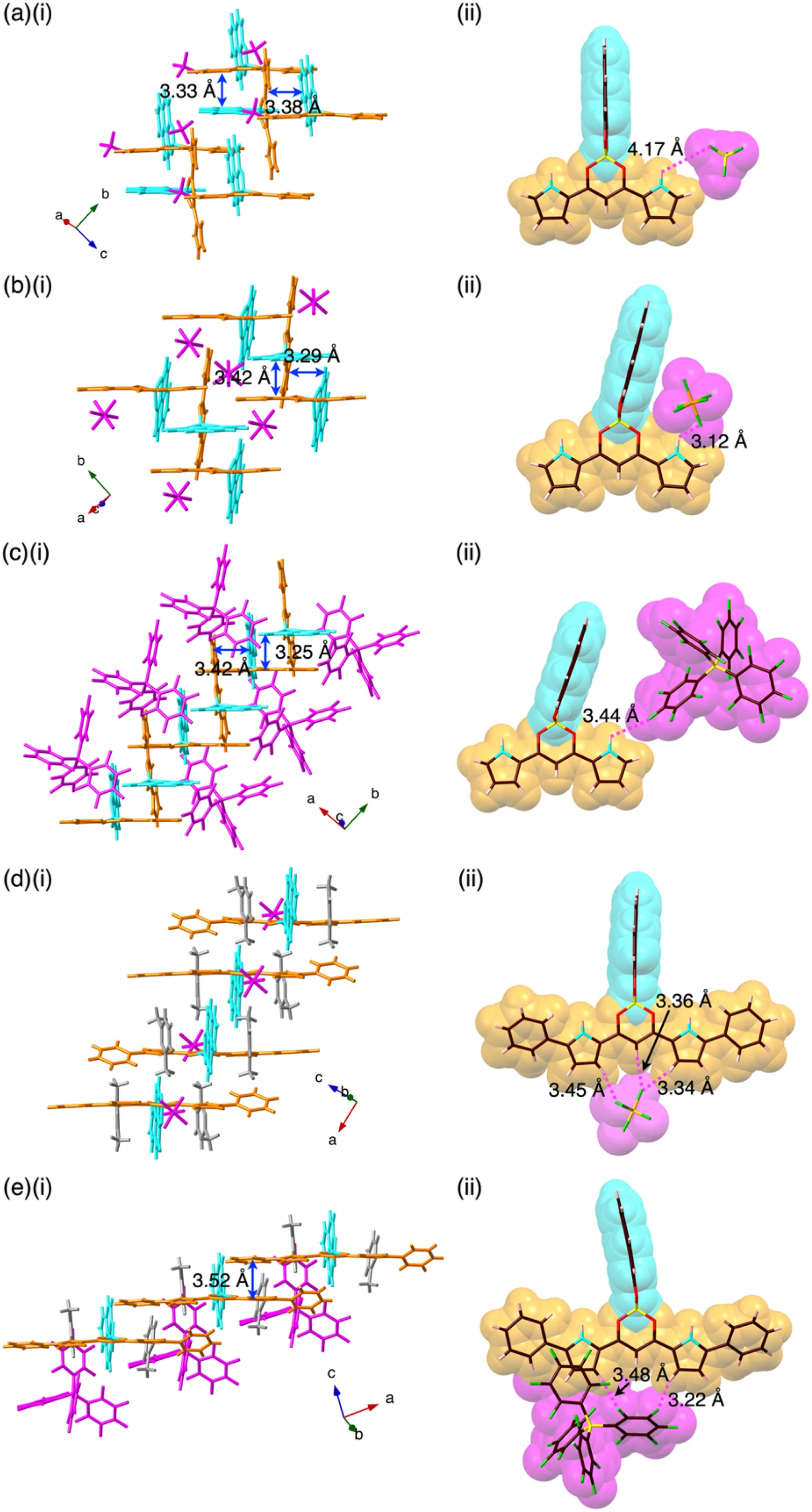
Single-crystal X-ray structures of (a) 3a^+^-BF_4_^−^, (b) 3a^+^-PF_6_^−^, (c) 3a^+^-B(C_6_F_5_)_4_^−^, (d) 3b^+^-PF_6_^−^ and (e) 3b^+^-B(C_6_F_5_)_4_^−^ as (i) packing structures and (ii) ion pairs (proximally located anions through hydrogen bonding are shown). Colour code in (i): grey refers to solvent molecules. Atom colour code in (ii): tangerine refers to phosphorus.

The results obtained under hydrostatic pressure in solution prompted us to examine anion-dependent crystal structures, in which differences in cation arrangement may be viewed as chemical pressure within analogous packing frameworks.^24^ Thus, investigating the effects of counteranion variation on the molecular conformations and arrangement of the spiroboronium ion pairs in the crystalline state is of particular interest. Single-crystal X-ray analysis of 3a^+^-BF_4_^−^, 3a^+^-PF_6_^−^ and 3a^+^-B(C_6_F_5_)_4_^−^ revealed pyrrole-uninverted conformation and orthogonally arranged geometries for the spiroboronium structure (Fig. 8a–c and S19–S21). Anions (BF_4_^−^, PF_6_^−^ and B(C_6_F_5_)_4_^−^) and pyrrole NH formed hydrogen bonding with the N(–H)⋯F distances of 4.17, 3.12 and 3.44 Å, respectively. The ion pairs formed 1D-array structures *via* stacking of the phenalenyl and dipyrrolyldiketone units, with stacking distances of 3.25–3.42 Å. Doubly stacked phenalenyl and dipyrrolyldiketone units were observed in 3a^+^-PF_6_^−^ and 3a^+^-B(C_6_F_5_)_4_^−^. Such stacking structures were also confirmed by Hirshfeld surface analysis,^10^ which showed a bowtie-shaped arrangement of red and blue triangles in the shape-index property and flat regions in the curvedness property, suggesting typical characteristics of π-plane stacking (Fig. S27–S29). 3b^+^-PF_6_^−^ and 3b^+^-B(C_6_F_5_)_4_^−^ showed pyrrole-uninverted structures, which formed hydrogen bonding of PF_6_^−^ and B(C_6_F_5_)_4_^−^ with the bridging CH and pyrrole-β-CH (Fig. 8d, e, S24 and S25). 2,6-Lutidine and 2-picoline, used as additives for crystallization, located proximally at the pyrrole NH through hydrogen bonding with the N(–H)⋯N distances of 2.89–2.95 Å. 2,6-Lutidine also formed stacking with the phenalenyl unit (3.35 and 3.37 Å). Solvents bearing a hydrogen-bonding acceptor unit were necessary for the crystallization. EDA calculations based on FMO2-MP2/cc-pVDZ for the two proximally located stacked 3a^+^ revealed total energies *E*_tot_ of 21.14, 7.94 and 6.32 kcal mol^−1^ for BF_4_^−^, PF_6_^−^ and B(C_6_F_5_)_4_^−^ ion pairs, respectively, which are mainly ascribable to favourable dispersion interaction energy *E*_disp_ and unfavourable electrostatic interaction energy *E*_es_ arising from the stacking of phenalenyl and dipyrrolyldiketone units (Fig. S67–S69). The smaller negative *E*_tot_ values for 3a^+^-BF_4_^−^ relative to those of 3a^+^-PF_6_^−^ and 3a^+^-B(C_6_F_5_)_4_^−^ are attributed to the singly stacked structures of the phenalenyl and dipyrrolyldiketone units.

**Fig. 9:**
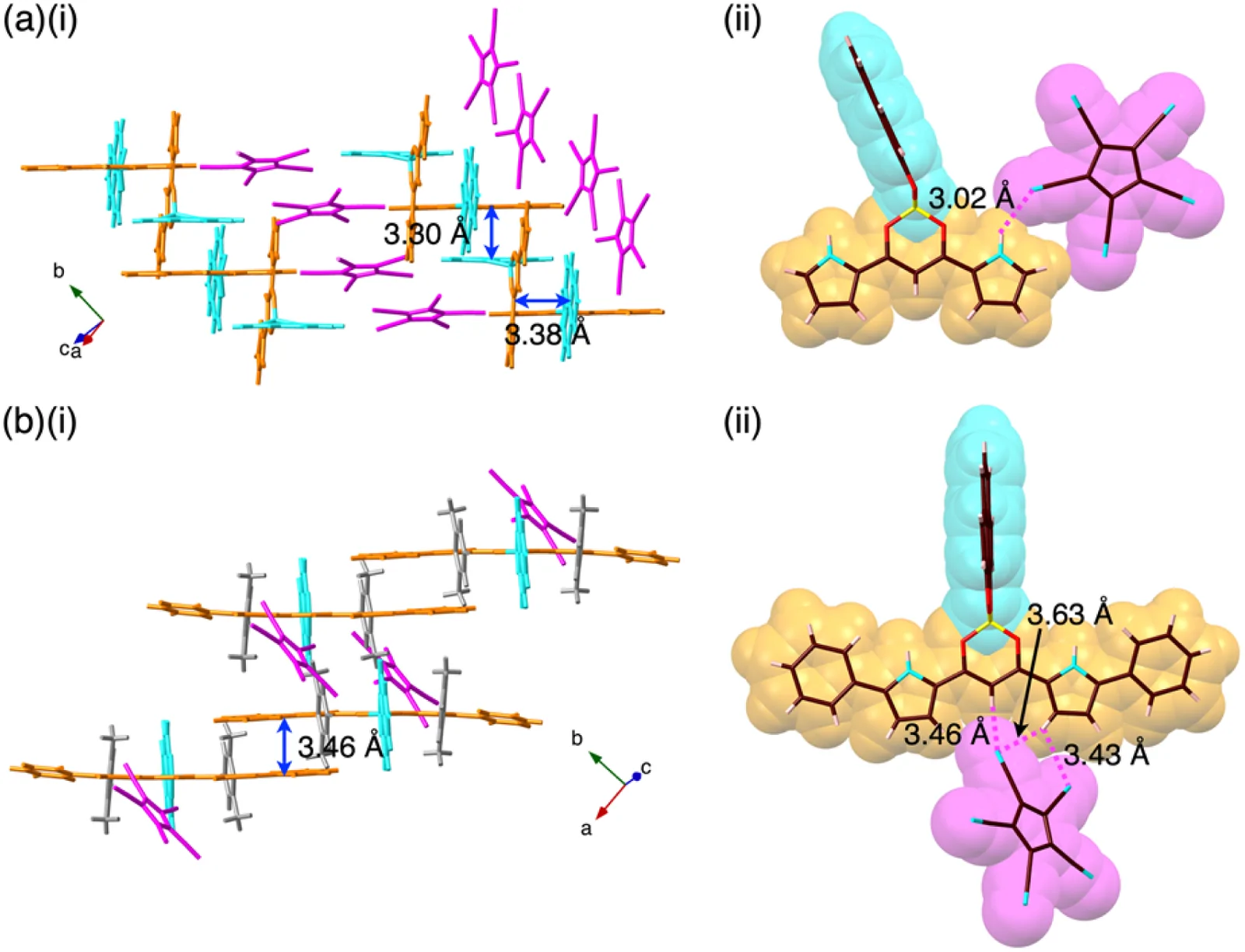
Single-crystal X-ray structures of (a) 3a^+^-PCCp^−^ and (b) 3b^+^-PCCp^−^ as (i) packing structures and (ii) ion pairs (proximally located anions through hydrogen bonding are shown).

The stacked 1D-array structure seen in the ion pairs of 3a^+^ was also observed in the packing structure of 3a^+^-PCCp^−^ (Fig. 9a and S22). The stacking distances for the phenalenyl and dipyrrolyldiketone units were 3.30 and 3.38 Å. PCCp^−^ and pyrrole NH formed hydrogen bonding with the N(–H)⋯N distance of 3.02 Å. By contrast, 3b^+^-PCCp^−^ formed a packing structure similar to that of 3b^+^-PF_6_^−^, including 2,6-lutidines at the top of the pyrrole NH to form hydrogen bonding (Fig. 9b and S26). PCCp^−^ is located proximal to the bridging CH and pyrrole-β-CH of 3b^+^*via* hydrogen bonding. EDA calculations based on FMO2-MP2/cc-pVDZ for the two proximally located stacked 3a^+^ revealed an *E*_tot_ of 7.90 kcal mol^−1^, similar to those of the PF_6_^−^ and B(C_6_F_5_)_4_^−^ ion pairs, exhibiting doubly stacked structures involving phenalenyl and dipyrrolyldiketone units (Fig. S70). Meanwhile, the *E*_tot_ between 3a^+^ and the nearest PCCp^−^ was −59.88 kcal mol^−1^. The partial stacking of PCCp^−^ and phenalenyl unit contributes to the favourable *E*_disp_, which stabilizes the ion-pairing structure. Given the role of PET in charge-carrier generation, the present findings provide valuable insights into the design of charge-transporting materials based on the ion-pairing assemblies.

## Conclusions

Orthogonally arranged π-electronic cations comprising anion-responsive dipyrrolyldiketone and phenalenyl units linked by a spiroboronium unit were synthesized. Ion pairs with Cl^−^ exhibit a Cl^−^-bound structure based on a pyrrole-inverted conformation, whereas those with larger anions introduced *via* ion-pair metathesis form unbound structures. These counteranion-dependent structural differences affect the electronic states, as evidenced by their ground- and excited-state behaviours. In particular, the photoexcitation of ion pairs induces electron transfer from the dipyrrolyldiketone unit to the phenalenyl unit, which is modulated by counteranion binding. Moreover, the orthogonally arranged π-electronic cations form a 1D-array structure in the solid state based on the doubly stacked phenalenyl and dipyrrolyldiketone units. π-Electronic cations with orthogonal π-systems exhibit characteristic pressure-responsive behaviours driven by anion-dependent conformational changes. Further design and synthesis of orthogonally arranged charged π-electronic systems exhibiting PET behaviour can promote charge-carrier generation in ion-pairing assemblies and enhancing electric conductivity for functional devices.

## Author contributions

H. M. designed and conducted the project. Y. H., T. I., T. A. and R. K. carried out the synthesis, characterization and property examinations. S. K., R. S. and Y. K. evaluated the transient absorption spectra. K. M., T. H. and G. F. evaluated the pressure-responsive absorption spectra. T. M. conducted the spectroelectrochemical analysis.

## Conflicts of interest

There are no conflicts of interest to declare.

## Supplementary Material

SC-017-D6SC05424B-s001

SC-017-D6SC05424B-s002

## Data Availability

CCDC 2561908–2561915 contain the supplementary crystallographic data for this paper.^25*a*–*h*^ Data supporting the work in this publication are available *via* the supplementary information (SI) and associated crystallographic data. Supplementary information: synthetic procedures, analytical data, computational details and CIF files for the single-crystal X-ray structural analysis. See DOI: https://doi.org/10.1039/d6sc05424b.
