# Supplementary material for: Anion-controlled ion pairing and assembly of π-electronic cations with orthogonal π-systems
Source: Chem Sci. 2026 Aug 17;17(36):17261–8. doi: 10.1039/d6sc05424b (PMC13502467; doi:10.1039/d6sc05424b)
Supplement: Supplementary file 1 [file SC-017-D6SC05424B-s001.pdf]

Supplementary Information (SI) for Chemical Science.
This journal is © The Royal Society of Chemistry 2026

Electronic Supplementary Information

Anion-controlled ion pairing and assembly of π-electronic cations with orthogonal π-systems

Yohei Haketa, Tomoka Inoue, Taichi Abiko, Shunsuke Kobashi, Ryota Sato, Yoichi Kobayashi,* Kotaro Matsumoto, Tomoyuki Hamachi, Gaku Fukuhara,* Tatsuki Morimoto, Rio Kugizaki and Hiromitsu Maeda*

Department of Applied Chemistry, College of Life Sciences, Ritsumeikan University, Kusatsu 525–8577, Japan, Fax: +81 77 561 3729; Tel: +81 77 561 5969; E-mail: maedahir@ph.ritsumei.ac.jp and ykobayas@fc.ritsumei.ac.jp, Department of Chemistry, Institute of Science Tokyo, Tokyo 152–8551, Japan, Institute for Materials Chemistry and Engineering, Kyushu University, Fukuoka 819–0395, Japan; E-mail: gaku@ms.ifoc.kyushu-u.ac.jp, and Department of Applied Chemistry, School of Engineering, Tokyo University of Technology, Hachioji 192–0982, Japan

Table of contents

1. Synthetic procedures and spectroscopic data S2
Fig. S1–9 ^{1}H, ^{13}C, and ^{19}F NMR spectra. S6
Fig. S10 UV/vis absorption spectra. S15
2. X-ray crystallographic data S16
Fig. S11–18 Ortep drawings. S17
Fig. S19–26 Packing diagrams. S22
Fig. S27–34 Hirshfeld surface analysis. S30
3. Theoretical studies S33
Fig. S35,36 Optimized structures. S33
Fig. S37,38 ESP mapping. S34
Fig. S39–66 Molecular orbitals and TD-DFT calculations. S35
Fig. S67–74 Single-crystal X-ray structures for EDA analysis. S57
Cartesian coordination of optimized structures S60
4. Solution-state properties S68
Fig. S75,76 Summarized ^{1}H and ^{11}B NMR spectra. S68
Fig. S77,78 ^{1}H NMR spectral changes upon the addition of Cl^{-}. S70
Fig. S79 UV/vis absorption spectral changes upon the addition of Cl^{-}. S72
Fig. S80 Cyclic voltammograms. S73
Fig. S81 Spectroelectrochemical analysis. S74
5. Transient absorption spectroscopic data S75
Fig. S82–88 Transient UV/vis absorption spectra and evolution-associated spectra. S75
6. Pressure-dependent UV/vis absorption spectra S78
Fig. S89 Pressure-dependent UV/vis absorption spectra. S78

1. Synthetic procedures and spectroscopic data

General Procedures. Starting materials were purchased from FUJIFILM Wako Pure Chemical Corp., Nacalai Tesque Inc., and Sigma-Aldrich Co. and used without further purification unless otherwise stated. NMR spectra used in the characterization of products were recorded on a JEOL ECA-600 600 MHz spectrometers. ^{1}H and ^{13}C NMR spectra were referenced to solvent, whereas ^{19}F and ^{11}B NMR spectra were referenced to C_6F_6 and $BF_3{\cdot}OEt_2$, respectively. UV-visible absorption spectra were recorded on a Hitachi U-3500 spectrometer. High-resolution (HR) electrospray ionization mass spectrometry (ESI-MS) was recorded on a BRUKER microTOF using ESI-TOF method. TLC analyses were carried out on aluminum sheets coated with silica gel 60 (Merck 5554). Column chromatography was performed on Wakogel C-300.

9-Oxidophenalenone boron complex of 1,3-dipyrrol-2-yl-1,3-propanedione as a Cl^{-} ion pair, 3a^{+}-Cl^{-}. According to the literature procedures,[S1] to a THF solution (5 mL) of 1,3-dipyrrol-2-yl-1,3-propanedione[S2] **1a** (91.0 mg, 0.45 mmol) was added a 1 M CH_2Cl_2 solution of BCl_3 (2.2 mL, 2.2 mmol) at r.t., and the reaction mixture was stirred for 1 h at the same temperature. After the addition of 9-hydroxyphenalenone (102.0 mg, 0.52 mmol), the reaction mixture was stirred for 12 h. The residue was purified by silica gel column chromatography (Wakogel C-300; eluent: 10% MeOH/CH_2Cl_2) and recrystallized from CH_2Cl_2/*n*-hexane to afford **3a**$^{+}$-Cl^{-} (52.68 mg, 0.12 mmol, 26%) as an orange solid. R_f = 0.30 (10% MeOH/CH_2Cl_2). ^{1}H NMR (600 MHz, DMSO-d_6, 5.0 × 10^{-2} M, 20 °C): δ (ppm) 12.55 (br, 2H, NH), 9.15 (d, J = 8.4 Hz, 2H, phenalenyl-H), 8.89 (d, J = 7.8 Hz, 2H, phenalenyl-H), 8.18 (t, J = 7.8 Hz, 1H, phenalenyl-H), 7.82 (d, J = 9.6 Hz, 2H, phenalenyl-H), 7.57 (br, 2H, pyrrole-H), 7.49 (br, 1H, CH), 7.44 (s, 2H, pyrrole-H), 6.51–6.50 (m, 2H, pyrrole-H). ^{13}C{^{1}H} NMR could not be measured due to the low solubility. ^{11}B NMR (192.5 MHz, CD_2Cl_2, 2.5 × 10^{-4} M, 20 °C): δ (ppm) 3.92. UV/vis (CH_2Cl_2, λ_{max}[nm] (ε, 10^5 $M^{-1}cm^{-1}$)): 386 (0.39), 458 (0.61). HRMS (ESI-TOF): (pos) *m/z* (% intensity): 407.1198. Calcd for $C_{24}H_{16}BN_2O_4^{+}$ ([M – Cl]$^{+}$): 407.1198.

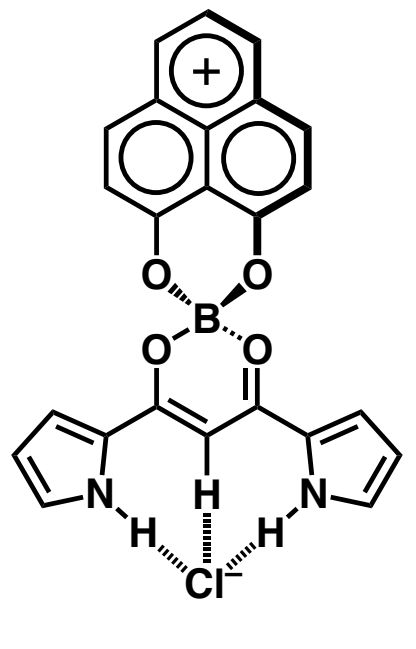

9-Oxidophenalenone boron complex of 1,3-dipyrrol-2-yl-1,3-propanedione as a BF_4^{-} ion pair, 3a^{+}-BF_4^{-}. To a CH_2Cl_2 solution (5 mL) of **3a**$^{+}$-Cl^{-} (7.92 mg, 0.018 mmol) was added $AgBF_4$ (7.85 mg, 0.040 mmol) and the reaction mixture was stirred at r.t. for 10 min, followed by filtration and evaporation to dryness. The residue was purified by silica gel column chromatography (Wakogel C-300; eluent: 10% MeOH/CH_2Cl_2) and recrystallized from CH_2Cl_2/*n*-hexane to afford **3a**$^{+}$-BF_4^{-} (6.44 mg, 0.013 mmol, 72%) as an orange solid. R_f = 0.35 (10% MeOH/CH_2Cl_2). ^{1}H NMR (600 MHz, DMSO-d_6, 5.0 × 10^{-2} M, 20 °C): δ (ppm) 12.54 (br, 2H, NH), 9.15 (d, J = 8.4 Hz, 2H, phenalenyl-H), 8.89 (d, J = 7.8 Hz, 2H, phenalenyl-H), 8.18 (t, J = 7.2 Hz, 1H, phenalenyl-H), 7.82 (d, J = 9.6 Hz, 2H, phenalenyl-H), 7.57 (br, 2H, pyrrole-H), 7.48 (br, 2H, pyrrole-H), 7.43 (s, 1H, CH), 6.51–6.50 (m, 2H, pyrrole-H). ^{13}C{^{1}H} NMR could not be measured due to the low solubility. ^{19}F NMR (564 MHz, DMSO-d_6, 20 °C): δ (ppm) –137.89 (s, $^{10}BF_4^{-}$), –137.93 (s, $^{11}BF_4^{-}$). UV/vis (CH_2Cl_2, λ_{max}[nm] (ε, 10^5 $M^{-1}cm^{-1}$)): 386 (0.49), 457 (0.84). HRMS (ESI-TOF): (pos) *m/z* (% intensity): 407.1198. Calcd for $C_{24}H_{16}BN_2O_4^{+}$ ([M – BF_4]$^{+}$): 407.1198. (neg) *m/z* (% intensity): 87.0035. Calcd for BF_4^{-} ([M – $C_{24}H_{16}BN_2O_4$]$^{-}$): 87.0035. This compound was further characterized by single-crystal X-ray diffraction analysis.

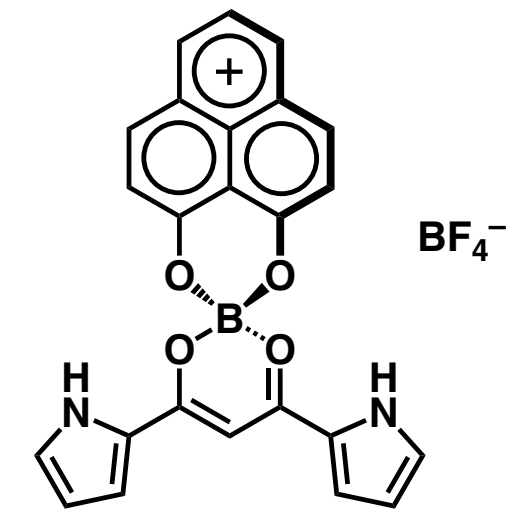

9-Oxidophenalenone boron complex of 1,3-dipyrrol-2-yl-1,3-propanedione as a PF_6^{-} ion pair, 3a^{+}-PF_6^{-}. To a CH_2Cl_2 solution (5 mL) of **3a**$^{+}$-Cl^{-} (8.11 mg, 0.018 mmol) was added $AgPF_6$ (10.46 mg, 0.041 mmol) and the reaction mixture was stirred at r.t. for 10 min, followed by filtration and evaporation to dryness. The residue was purified by silica gel column chromatography (Wakogel C-300; eluent: 5% MeOH/CH_2Cl_2) and recrystallized from CH_2Cl_2/*n*-hexane to afford **3a**$^{+}$-PF_6^{-} (8.32 mg, 0.015 mmol, 84%) as an orange solid. R_f = 0.30 (5% MeOH/CH_2Cl_2). ^{1}H NMR (600 MHz, DMSO-d_6, 5.0 × 10^{-2} M, 20 °C): δ (ppm) 12.53 (br, 2H, NH), 9.14 (d, J = 8.4 Hz, 2H, phenalenyl-H), 8.88 (d, J = 7.2 Hz, 2H, phenalenyl-H), 8.16 (t, J = 6.6 Hz, 1H, phenalenyl-H), 7.81 (d, J = 9.0 Hz, 2H, phenalenyl-H), 7.56 (br, 2H, pyrrole-H), 7.47 (br, 2H, pyrrole-H), 7.42 (s, 1H, CH), 6.49 (s, 2H, pyrrole-H). ^{13}C{^{1}H} NMR could not be measured due to the low solubility. ^{19}F NMR (564 MHz, DMSO-d_6, 20 °C): δ (ppm) –59.81 (d, J = 755.4 Hz, 6F). UV/vis (CH_2Cl_2, λ_{max}[nm] (ε, 10^5 $M^{-1}cm^{-1}$)): 386 (0.47), 457 (0.81). HRMS (ESI-TOF): (pos) *m/z* (% intensity): 407.1198. Calcd for $C_{24}H_{16}BN_2O_4^{+}$ ([M – F_6P]$^{+}$): 407.1198. (neg) *m/z* (% intensity): 144.9649. Calcd for F_6P^{-} ([M – $C_{24}H_{16}BN_2O_4$]$^{-}$): 144.9647. This compound was further characterized by single-crystal X-ray diffraction analysis.

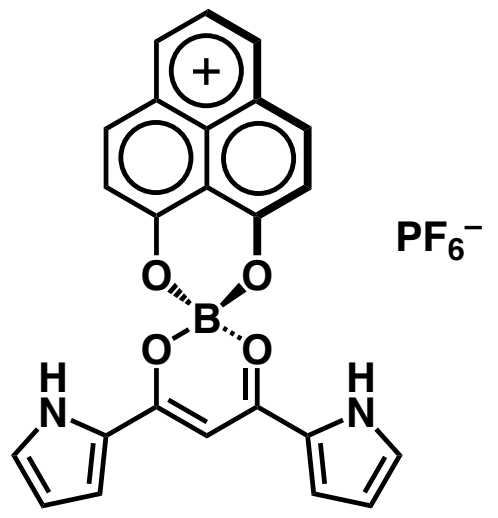

9-Oxidophenalenone boron complex of 1,3-dipyrrol-2-yl-1,3-propanedione as a $B(C_6F_5)_4^-$ ion pair, $3a^+$-$B(C_6F_5)_4^-$. To a CH_2Cl_2 solution (5 mL) of **3a**$^+$-Cl^- (8.34 mg, 0.019 mmol) was added lithium tetrakis(pentafluorophenyl)borate ($LiB(C_6F_5)_4$) (22.21 mg, 0.033 mmol) and the reaction mixture was stirred at r.t. for 10 min, followed by filtration and evaporation to dryness. The residue was purified by silica gel column chromatography (Wakogel C-300; eluent: 5% $MeOH/CH_2Cl_2$) and recrystallized from CH_2Cl_2/*n*-hexane to afford **3a**$^+$-$B(C_6F_5)_4^-$ (12.69 mg, 0.012 mmol, 61%) as an orange solid. R_f = 0.30 (5% $MeOH/CH_2Cl_2$). ^1H NMR (600 MHz, DMSO-d_6, 5.0 × 10^{-2} M, 20 °C): δ (ppm) 12.53 (br, 2H, NH), 9.15 (d, J = 9.0 Hz, 2H, phenalenyl-H), 8.89 (d, J = 6.6 Hz, 2H, phenalenyl-H), 8.18 (t, J = 7.8 Hz, 1H, phenalenyl-H), 7.82 (d, J = 7.8 Hz, 2H, phenalenyl-H), 7.57 (br, 2H, pyrrole-H), 7.48 (br, 2H, pyrrole-H), 7.42 (s, 1H, CH), 6.51–6.50 (m, 2H, pyrrole-H). $^{13}C\{^1H\}$ NMR (151 MHz, DMSO-d_6, 20 °C): δ (ppm) 170.83, 165.86, 150.33, 147.50 (dm, $J_{13C-19F}$ = 246 Hz, $B(C_6F_5)_4^-$), 140.27, 137.64 (dm, $J_{13C-19F}$ = 246 Hz, $B(C_6F_5)_4^-$), 135.58 (dm, $J_{13C-19F}$ = 246 Hz, $B(C_6F_5)_4^-$), 131.19, 127.91, 126.02, 125.32, 123.18, 121.16, 113.59, 109.13, 90.03 (A signal is missing probably due to the overlapping with another signal and a multiplet signal at ~123 ppm is observed for one of the $B(C_6F_5)_4^-$-C). ^{11}B NMR (192.5 MHz, CD_2Cl_2, 2.5 × 10^{-4} M, 20 °C): δ (ppm) 3.60 (**3a**$^+$-B), –16.68 ($B(C_6F_5)_4^-$). ^{19}F NMR (564 MHz, DMSO-d_6, 20 °C): δ (ppm) –122.12 (s, 8F), –150.92 (t, J = 22.8 Hz, 4F), –155.55 (t, J = 18.6 Hz, 8F). UV/vis (CH_2Cl_2, λ_{max}[nm] (ε, 10^5 $M^{-1}cm^{-1}$)): 386 (0.49), 457 (0.86). HRMS (ESI-TOF): (pos) *m/z* (% intensity): 407.1198. Calcd for $C_{24}H_{16}BN_2O_4^+$ ([M – $C_{24}BF_{20}$]$^+$): 407.1198. (neg) *m/z* (% intensity): 678.9779. Calcd for $C_{24}BF_{20}^-$ ([M – $C_{24}H_{16}BN_2O_4$]$^-$): 678.9779. This compound was further characterized by single-crystal X-ray diffraction analysis.

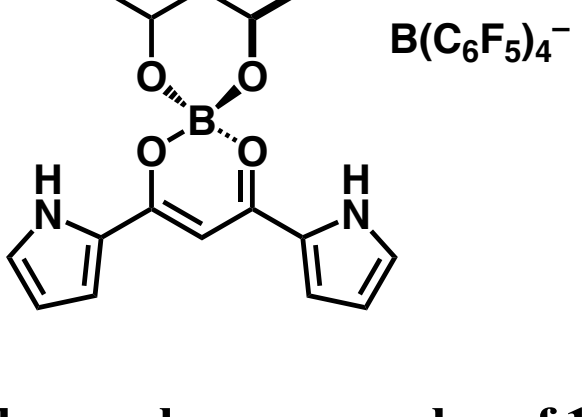

9-Oxidophenalenone boron complex of 1,3-dipyrrol-2-yl-1,3-propanedione as a PCCp$^-$ ion pair, 3a$^+$-PCCp$^-$. To a CH_2Cl_2 solution (5 mL) of **3a**$^+$-Cl^- (7.38 mg, 0.017 mmol) was added sodium pentacyanocyclopentadienide (NaPCCp)[S3] (5.23 mg, 0.028 mmol) and the reaction mixture was stirred at r.t. for 10 min, followed by filtration and evaporation to dryness. The residue was purified by silica gel column chromatography (Wakogel C-300; eluent: 5% $MeOH/CH_2Cl_2$) and recrystallized from CH_2Cl_2/*n*-hexane to afford **3a**$^+$-PCCp$^-$ (9.90 mg, 0.017 mmol, 100%) as an orange solid. R_f = 0.30 (3% $MeOH/CH_2Cl_2$). ^1H NMR (600 MHz, DMSO-d_6, 5.0 × 10^{-2} M, 20 °C): δ (ppm) 12.54 (br, 2H, NH), 9.15 (d, J = 9.6 Hz, 2H, phenalenyl-H), 8.89 (d, J = 7.8 Hz, 2H, phenalenyl-H), 8.18 (t, J = 7.8 Hz, 1H, phenalenyl-H), 7.82 (d, J = 9.0 Hz, 2H, phenalenyl-H), 7.57 (br, 2H, pyrrole-H), 7.49 (br, 2H, pyrrole-H), 7.43 (s, 1H, CH), 6.51–6.50 (m, 2H, pyrrole-H). $^{13}C\{^1H\}$ NMR could not be measured due to the low solubility. UV/vis (CH_2Cl_2, λ_{max}[nm] (ε, 10^5 $M^{-1}cm^{-1}$)): 386 (0.47), 457 (0.83). HRMS (ESI-TOF): (pos) *m/z* (% intensity): 407.1198. Calcd for $C_{24}H_{16}BN_2O_4^+$ ([M – $C_{10}N_5$]$^+$): 407.1198. (neg) *m/z* (% intensity): 190.0159. Calcd for $C_{10}N_5^-$ ([M – $C_{24}H_{16}BN_2O_4$]$^-$): 190.0159. This compound was further characterized by single-crystal X-ray diffraction analysis.

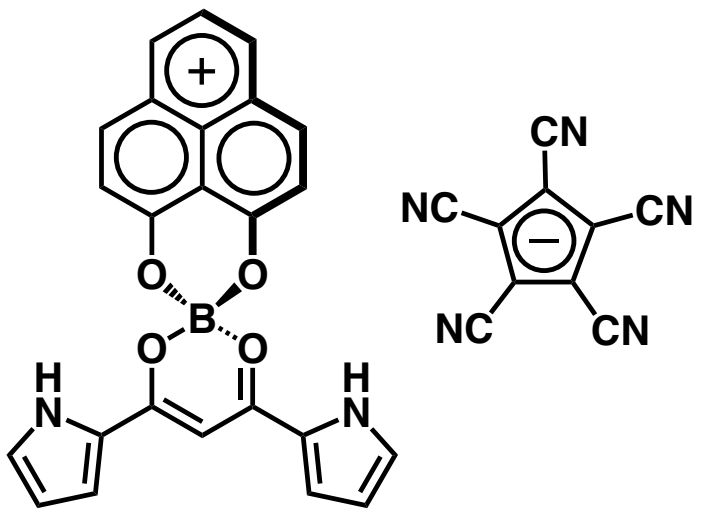

9-Oxidophenalenone boron complex of 1,3-di(5-phenylpyrrol-2-yl)-1,3-propanedione as a Cl^- ion pair, 3b$^+$-Cl^-. According to the literature procedures,[S1] to a THF solution (15 mL) of 1,3-di(5-phenylpyrrol-2-yl)-1,3-propanedione[S4] **1b** (85.9 mg, 0.24 mmol) was added a 1 M CH_2Cl_2 solution of BCl_3 (0.73 mL, 0.24 mmol) at r.t., and the reaction mixture was stirred for 1 h at the same temperature. After the addition of 9-hydroxyphenalenone (47.5 mg, 0.24 mmol), the reaction mixture was stirred for 1.5 h. The residue was purified by silica gel column chromatography (Wakogel C-300; eluent: 8% $MeOH/CH_2Cl_2$) to afford **3b**$^+$-Cl^- (34.9 mg, 0.059 mmol, 24%) as a red solid. R_f = 0.29 (8% $MeOH/CH_2Cl_2$). ^1H NMR (600 MHz, DMSO-d_6, 5.0 × 10^{-2} M, 20 °C): δ (ppm) 12.48 (br, 2H, NH), 9.15 (d, J = 9.6 Hz, 2H, phenalenyl-H), 8.89 (d, J = 7.8 Hz, 2H, phenalenyl-H), 8.18 (t, J = 7.8 Hz, 1H, phenalenyl-H), 7.93 (d, J = 7.2 Hz, 4H, phenyl-H), 7.85 (d, J = 9.0 Hz, 2H, phenalenyl-H), 7.69 (br, 2H, pyrrole-H), 7.64 (s, 1H, CH), 7.44 (t, J = 7.2 Hz, 4H, phenyl-H), 7.38 (t, J = 7.2 Hz, 2H, phenyl-H), 7.06 (br, 2H, pyrrole-H). $^{13}C\{^1H\}$ NMR could not be measured due to the low solubility. ^{11}B NMR (192.5 MHz, CD_2Cl_2, 2.5 × 10^{-4} M, 20 °C): δ (ppm) 3.87. UV/vis (CH_2Cl_2, λ_{max}[nm] (ε, 10^5 $M^{-1}cm^{-1}$)): 301 (0.50), 341 (0.28), 539 (0.66). HRMS (ESI-TOF): (pos) *m/z* (% intensity): 559.1824. Calcd for $C_{36}H_{24}BN_2O_4^+$ ([M – Cl]$^+$): 559.1824. This compound was further characterized by single-crystal X-ray diffraction analysis.

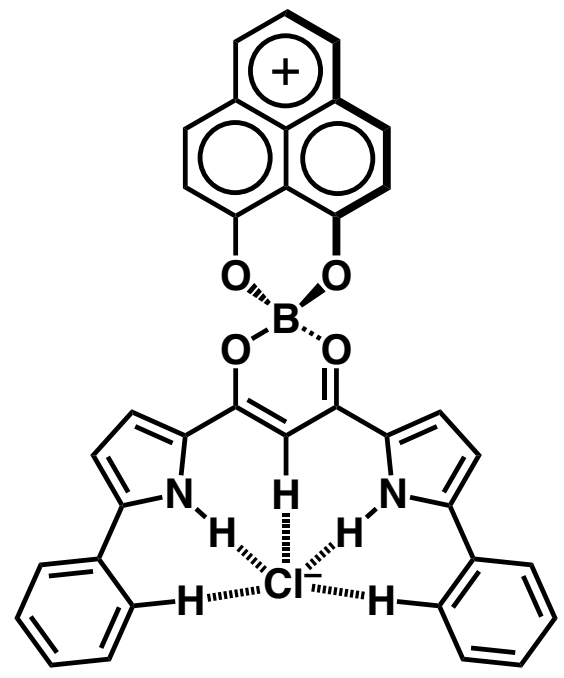

9-Oxidophenalenone boron complex of 1,3-di(5-phenylpyrrol-2-yl)-1,3-propanedione as a PF_6^- ion pair, $3b^+$-PF_6^-. To a THF/MeOH solution (2/8 mL) of **3b**$^+$-Cl^- (6.08 mg, 0.010 mmol) was added $AgPF_6$ (8.93 mg, 0.035 mmol) and the reaction mixture was stirred at r.t. for 30 min, followed by filtration and evaporation to dryness. The residue was purified by silica gel column chromatography (Wakogel C-300; eluent: 4% $MeOH/CH_2Cl_2$) and recrystallized from CH_2Cl_2/*n*-hexane to afford **3b**$^+$-PF_6^- (4.23 mg, 6.0 μmol, 60%) as a red solid. R_f = 0.16 (4% $MeOH/CH_2Cl_2$). ^{1}H NMR (600 MHz, DMSO-d_6, 5.0 × 10^{-2} M, 20 °C): δ (ppm) 12.45 (br, 2H, NH), 9.15 (d, J = 9.0 Hz, 2H, phenalenyl-H), 8.89 (d, J = 7.8 Hz, 2H, phenalenyl-H), 8.18 (t, J = 7.8 Hz, 1H, phenalenyl-H), 7.92 (d, J = 7.2 Hz, 4H, phenyl-H), 7.85 (d, J = 9.6 Hz, 2H, phenalenyl-H), 7.69 (br, 2H, pyrrole-H), 7.58 (s, 1H, CH), 7.44 (t, J = 7.8 Hz, 4H, phenyl-H), 7.38 (d, J = 7.2 Hz, 2H, phenyl-H), 7.06 (br, 2H, pyrrole-H). $^{13}C\{^1H\}$ NMR could not be measured due to the low solubility. ^{19}F NMR (564 MHz, DMSO-d_6, 20 °C): δ (ppm) –59.77 (d, J = 755.4 Hz, 6F). UV/vis (CH_2Cl_2, λ_{max}[nm] (ε, 10^5 $M^{-1}cm^{-1}$)): 374 (0.17), 536 (0.85). HRMS (ESI-TOF): (pos) *m/z* (% intensity): 559.1824. Calcd for $C_{36}H_{24}BN_2O_4$ ([M – F_6P]$^+$): 559.1824. (neg) *m/z* (% intensity): 144.9649. Calcd for F_6P^- ([M – $C_{36}H_{24}BN_2O_4$]$^-$): 144.9647. This compound was further characterized by single-crystal X-ray diffraction analysis.

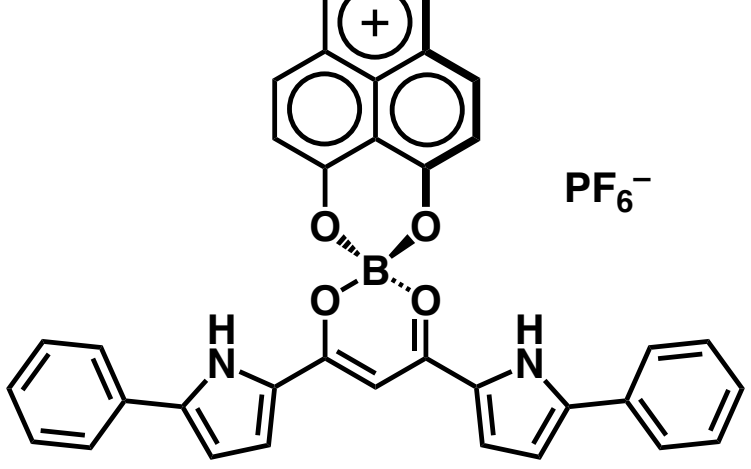

9-Oxidophenalenone boron complex of 1,3-di(5-phenylpyrrol-2-yl)-1,3-propanedione as a $B(C_6F_5)_4^-$ ion pair, $3b^+$-$B(C_6F_5)_4^-$. To a THF/MeOH solution (2/8 mL) of **3b**$^+$-Cl^- (6.00 mg, 0.010 mmol) was added $LiB(C_6F_5)_4$ (23.7 mg, 0.035 mmol) and the reaction mixture was stirred at r.t. for 30 min, followed by filtration and evaporation to dryness. The residue was purified by silica gel column chromatography (Wakogel C-300; eluent: 1% $MeOH/CH_2Cl_2$) and recrystallized from CH_2Cl_2/*n*-hexane to afford **3b**$^+$-$B(C_6F_5)_4^-$ (12.3 mg, 0.099 mmol, 99%) as a red solid. R_f = 0.45 (1% $MeOH/CH_2Cl_2$). ^{1}H NMR (600 MHz, DMSO-d_6, 5.0 × 10^{-2} M, 20 °C): δ (ppm) 12.46 (br, 2H, NH), 9.15 (d, J = 9.6 Hz, 2H, phenalenyl-H), 8.89 (d, J = 7.8 Hz, 2H, phenalenyl-H), 8.18 (t, J = 7.8 Hz, 1H, phenalenyl-H), 7.92 (d, J = 7.8 Hz, 4H, phenyl-H), 7.86 (d, J = 9.0 Hz, 2H, phenalenyl-H), 7.69 (br, 2H, pyrrole-H), 7.58 (s, 1H, CH), 7.44 (t, J = 7.8 Hz, 4H, phenyl-H), 7.38 (t, J = 7.2 Hz, 2H, phenyl-H), 7.06 (br, 2H, pyrrole-H). $^{13}C\{^1H\}$ NMR (151 MHz, DMSO-d_6, 20 °C): δ (ppm) 170.84, 164.30, 150.23, 147.50 (dm, $J_{13C-19F}$ = 240 Hz, $B(C_6F_5)_4^-$), 143.55, 140.17, 137.65 (dm, $J_{13C-19F}$ = 249 Hz, $B(C_6F_5)_4^-$), 135.59 (dm, $J_{13C-19F}$ = 246 Hz, $B(C_6F_5)_4^-$), 129.94, 129.03, 128.90, 127.88, 126.78, 126.26, 125.99, 123.22, 122.71, 121.19, 112.36, 108.89, 90.88 (A multiplet signal at ~123 ppm is observed for one of the $B(C_6F_5)_4^-$-C). ^{11}B NMR (192.5 MHz, CD_2Cl_2, 2.5 × 10^{-4} M, 20 °C): δ (ppm) 3.59 (**3b**$^+$-B), –16.67 ($B(C_6F_5)_4^-$). ^{19}F NMR (564 MHz, DMSO-d_6, 20 °C): δ (ppm) –122.11 (d, J = 13.8 Hz, 8F), –150.91 (t, J = 18.6 Hz, 4F), –155.54 (t, J = 18.6 Hz, 8F). UV/vis (CH_2Cl_2, λ_{max}[nm] (ε, 10^5 $M^{-1}cm^{-1}$)): 374 (0.18), 536 (0.92). HRMS (ESI-TOF): (pos) *m/z* (% intensity): 559.1824. Calcd for $C_{36}H_{24}BN_2O_4^+$ ([M – $C_{24}BF_{20}$]$^+$): 559.1824. (neg) *m/z* (% intensity): 678.9779. Calcd for $C_{24}BF_{20}^-$ ([M – $C_{36}H_{24}BN_2O_4$]$^-$): 678.9779. This compound was further characterized by single-crystal X-ray diffraction analysis.

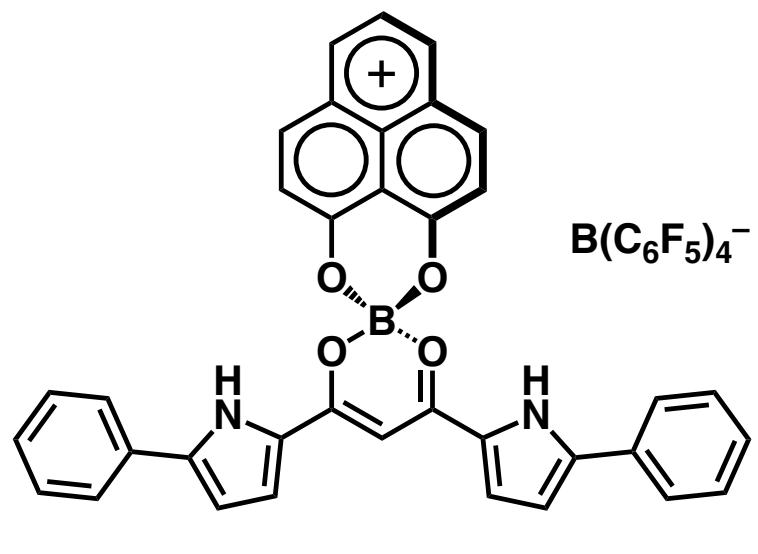

9-Oxidophenalenone boron complex of 1,3-di(5-phenylpyrrol-2-yl)-1,3-propanedione as a $PCCp^-$ ion pair, $3b^+$-$PCCp^-$. To a THF/MeOH solution (2/8 mL) of **3b**$^+$-Cl^- (5.19 mg, 8.7 μmol) was added NaPCCp[S3] (5.93 mg, 0.028 mmol) and the reaction mixture was stirred at r.t. for 30 min, followed by filtration and evaporation to dryness. The residue was purified by silica gel column chromatography (Wakogel C-300; eluent: 6% $MeOH/CH_2Cl_2$) and recrystallized from CH_2Cl_2/*n*-hexane to afford **3b**$^+$-$PCCp^-$ (5.89 mg, 7.9 μmol, 90%) as a red solid. R_f = 0.46 (6% $MeOH/CH_2Cl_2$). ^{1}H NMR (600 MHz, DMSO-d_6, 5.0 × 10^{-2} M, 20 °C): δ(ppm) 12.46 (br, 2H, NH), 9.15 (d, J = 9.0 Hz, 2H, phenalenyl-H), 8.89 (d, J = 7.8 Hz, 2H, phenalenyl-H), 8.18 (t, J = 7.8 Hz, 1H, phenalenyl-H), 7.92 (d, J = 7.8 Hz, 4H, phenyl-H), 7.86 (d, J = 9.6 Hz, 2H, phenalenyl-H), 7.69 (br, 2H, pyrrole-H), 7.58 (s, 1H, CH), 7.44 (t, J = 7.2 Hz, 4H, phenyl-H), 7.38 (t, J = 7.2 Hz, 2H, phenyl-H), 7.07–7.06 (m, 2H, pyrrole-H). $^{13}C\{^1H\}$ NMR could not be measured due to the low solubility. UV/vis (CH_2Cl_2, λ_{max}[nm] (ε, 10^5 $M^{-1}cm^{-1}$)): 374 (0.17), 536 (0.90). HRMS (ESI-TOF): (pos) *m/z* (% intensity): 559.1824. Calcd for $C_{36}H_{24}BN_2O_4^+$ ([M – $C_{10}N_5$]$^+$): 559.1824. (neg) *m/z* (% intensity): 190.0159. Calcd for $C_{10}N_5^-$ ([M – $C_{36}H_{24}BN_2O_4$]$^-$): 190.0159.

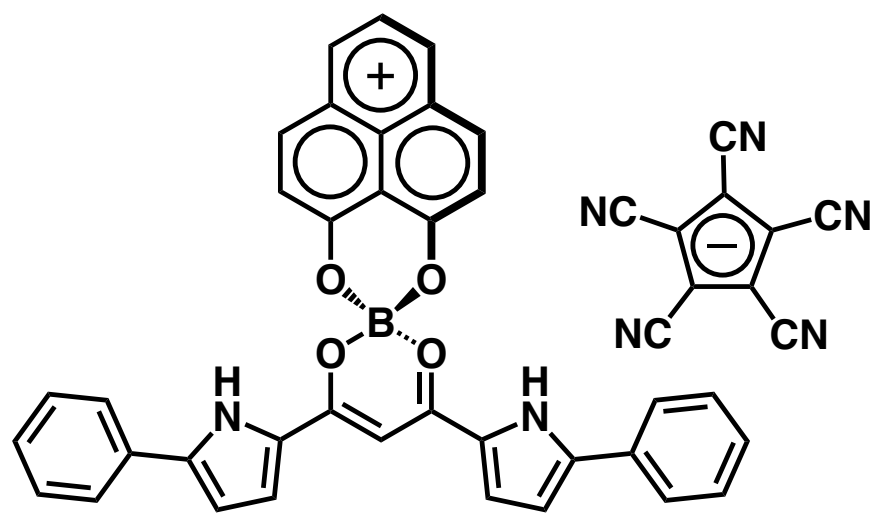

[S1] (a) H. Maeda, Y. Fujii and Y. Mihashi, *Chem. Commun.*, 2008, 4285–4287; (b) H. Maeda, M. Takayama, K. Kobayashi and H. Shinmori, *Org. Biomol. Chem.*, 2010, **8**, 4308–4315; (c) N. Koda, Y. Haketa, M. Yokoyama, N. Yasuda and H. Maeda, *Org. Lett.*, 2023, **25**, 1120–1125.
[S2] (a) B. Oddo and C. Dainotti, *Gazz. Chim. Ital.*, 1912, **42**, 716–726; (b) H. Maeda and Y. Kusunose, *Chem. Eur. J.*, 2005, **11**, 5661–5666.
[S3] (a) O. W. Webster, *J. Am. Chem. Soc.*, 1965, **87**, 1820–1821; (b) T. Sakai, S. Seo, J. Matsuoka and Y. Mori, *J. Org. Chem.*, 2013, **78**, 10978–10985.
[S4] H. Maeda, Y. Haketa and T. Nakanishi, *J. Am. Chem. Soc.*, 2007, **129**, 13661–13674.

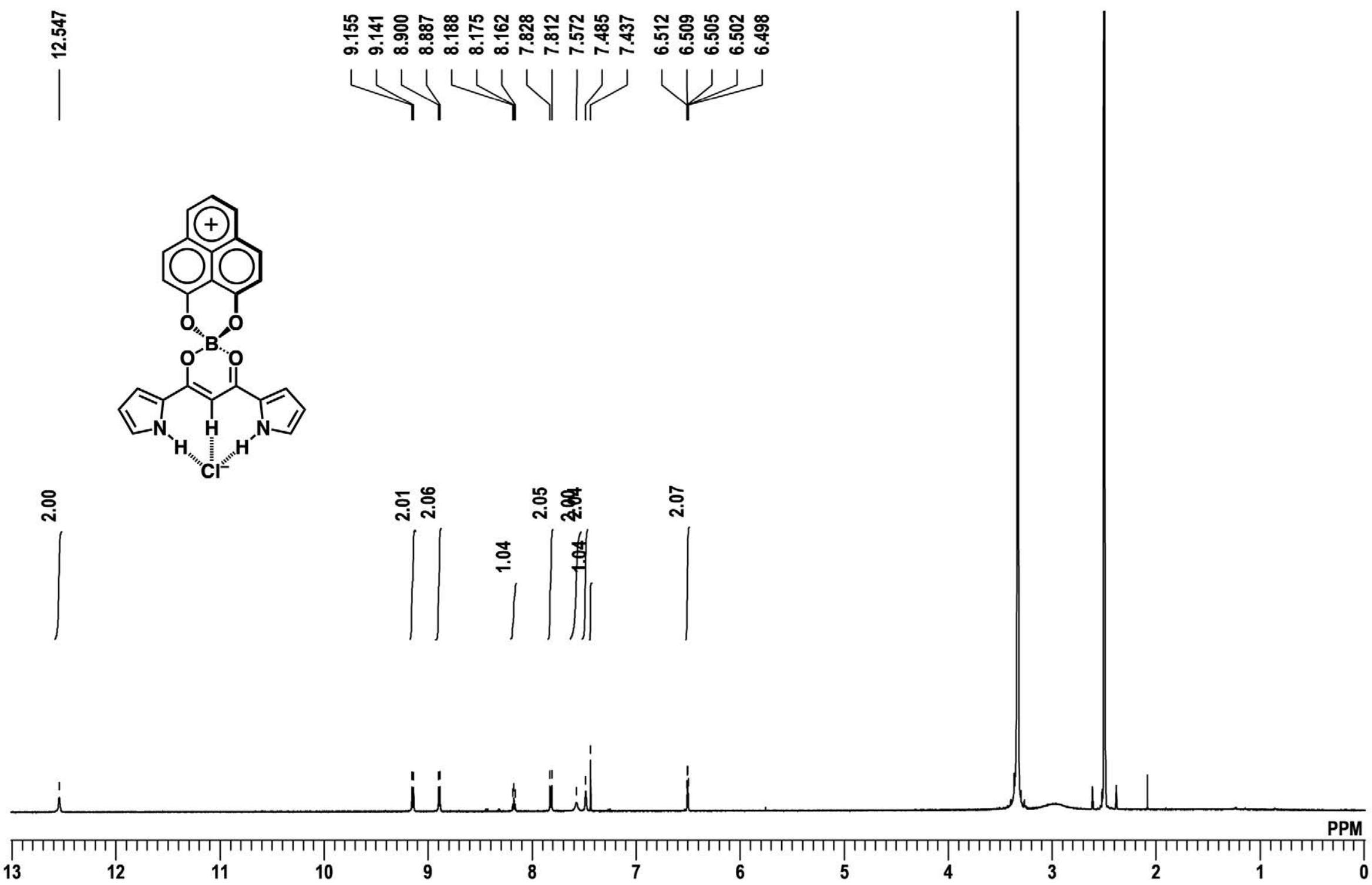

Fig. S1 ^{1}H NMR spectrum of **3a**$^+$-Cl$^-$ in DMSO-d_6.

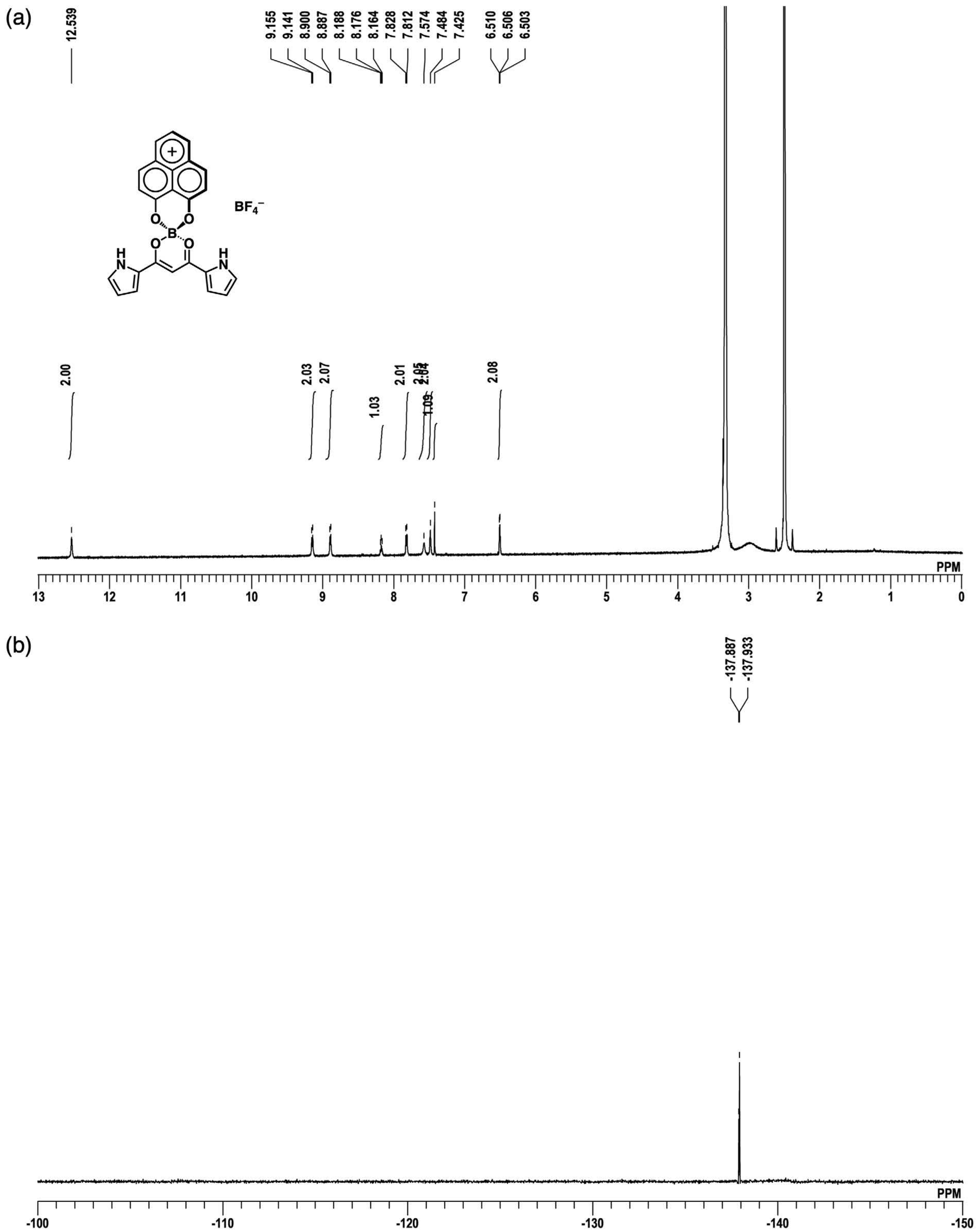

Fig. S2 (a) ^{1}H NMR and (b) ^{19}F NMR spectra of **3a**$^{+}$-BF_4^{-} in DMSO-d_6.

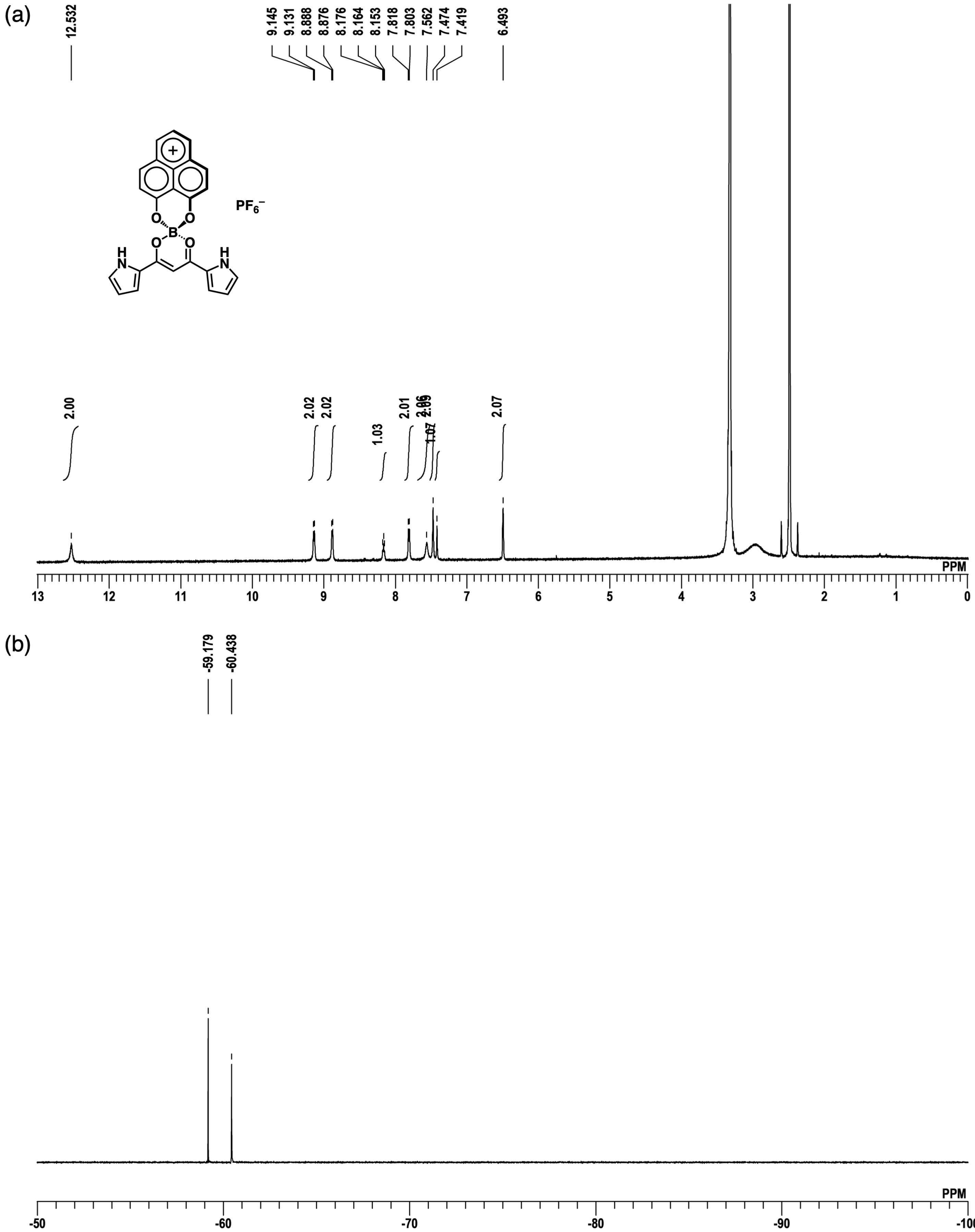

Fig. S3 (a) ^{1}H NMR and (b) ^{19}F NMR spectra of **3a**$^{+}$-PF_6^{-} in DMSO-d_6.

(a)

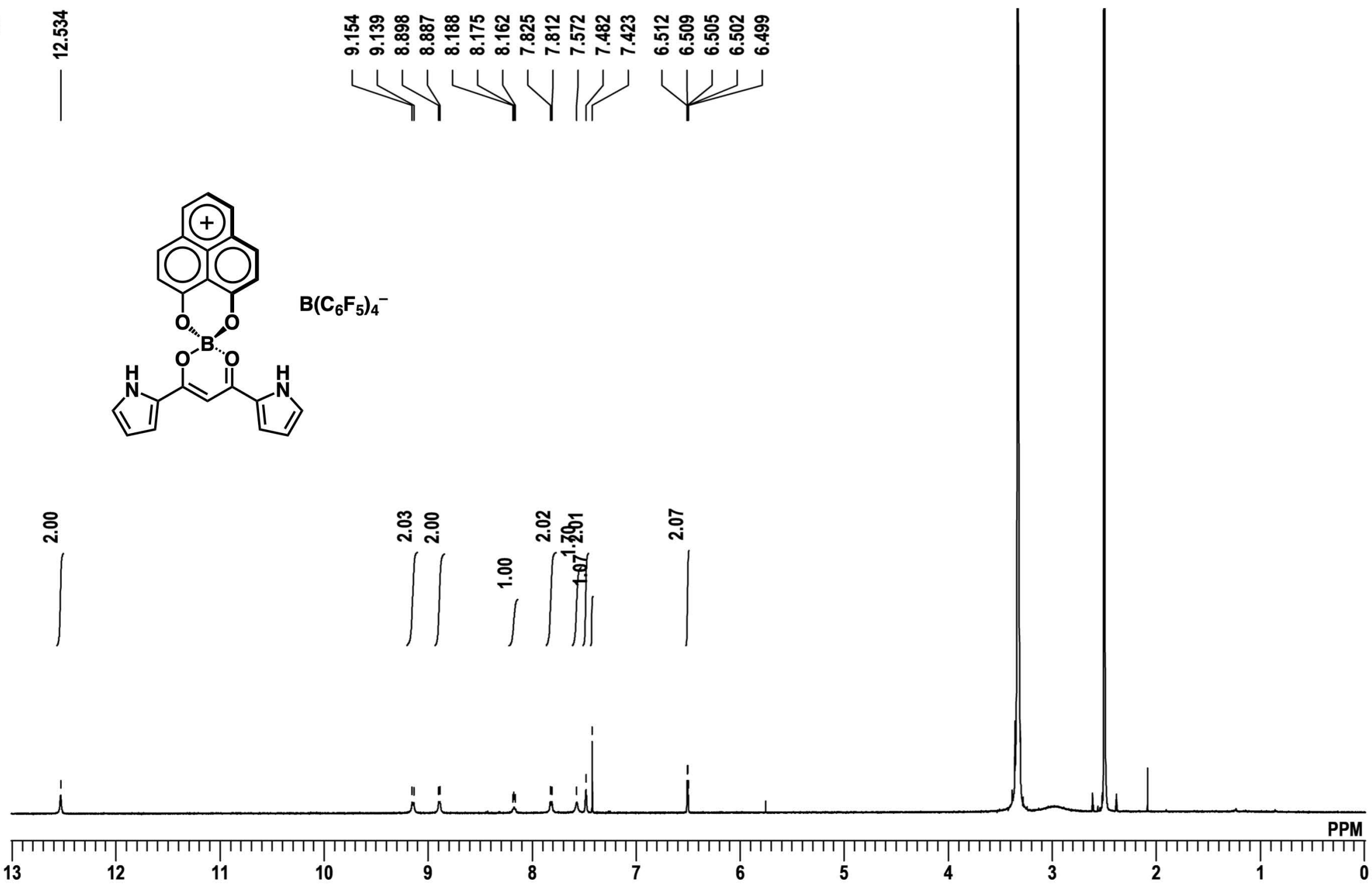

(b)

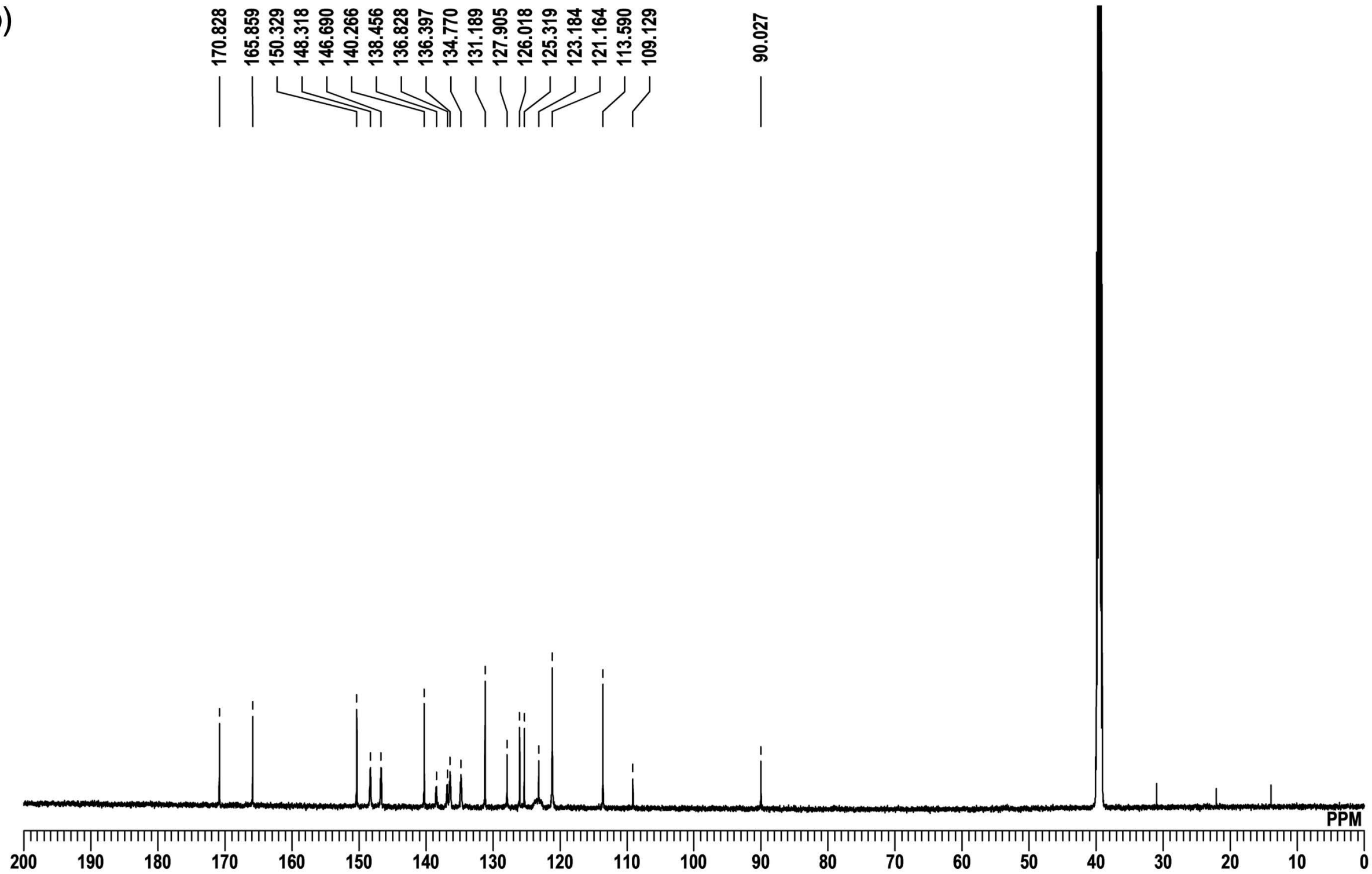

Fig. S4 (a) ^{1}H NMR, (b) ^{13}C{^{1}H} NMR, and (c) ^{19}F NMR spectra of **3a**$^{+}$-$B(C_6F_5)_4^{-}$ in DMSO-*d*$_6$.

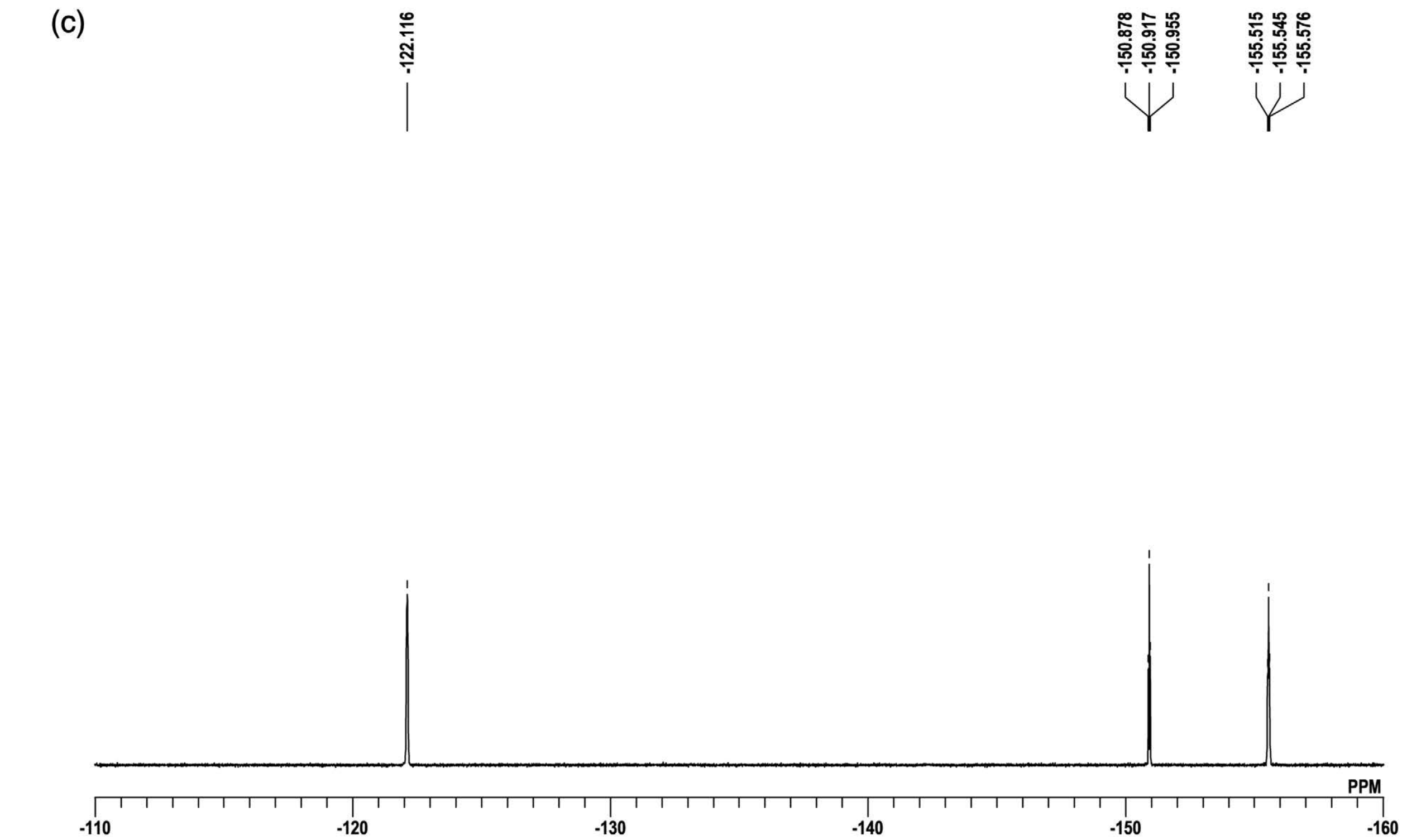

Fig. S4 (Continued)

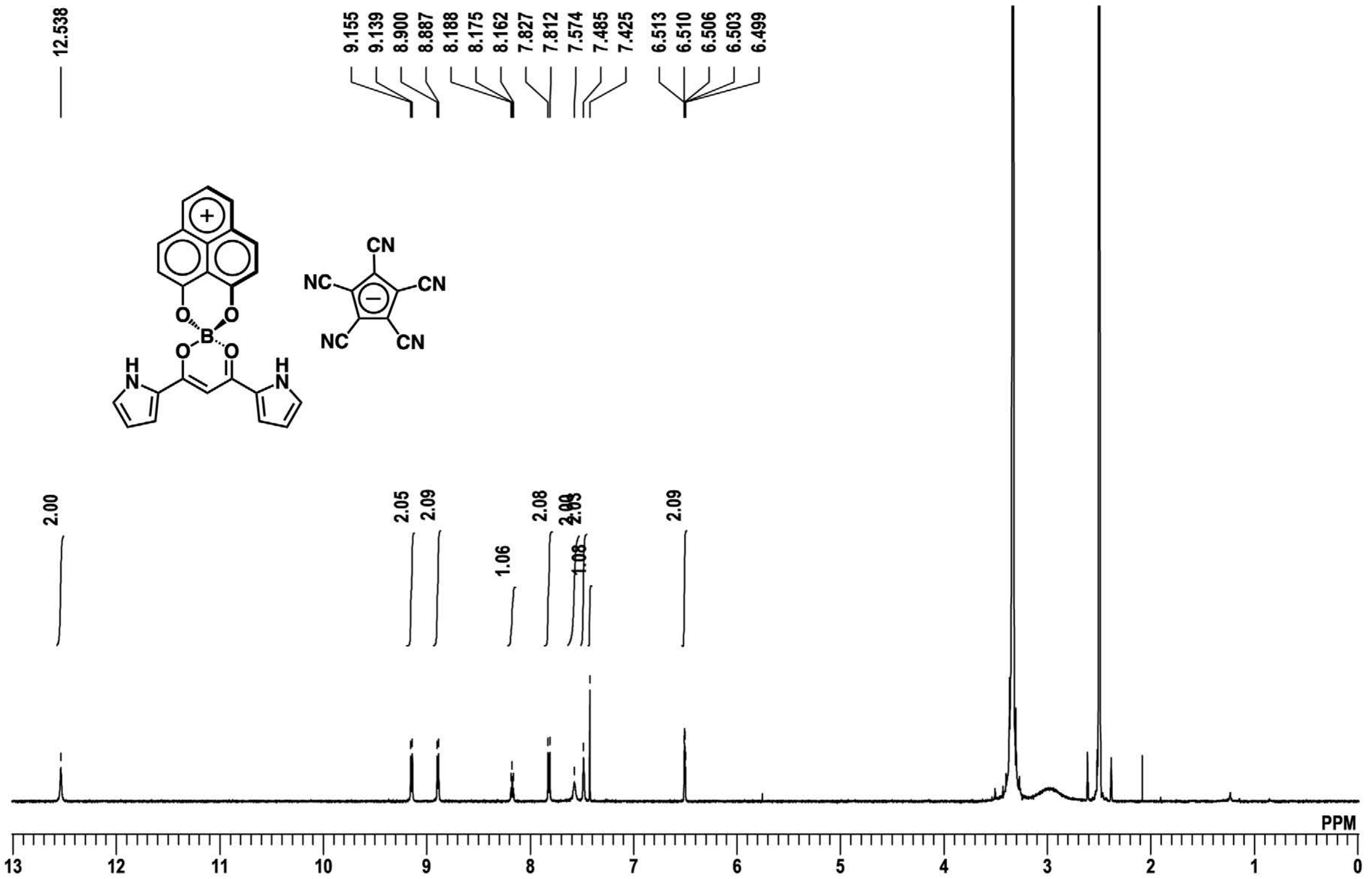

Fig. S5 ^{1}H NMR spectrum of **3a**$^{+}$-PCCp^{-} in DMSO-d_6.

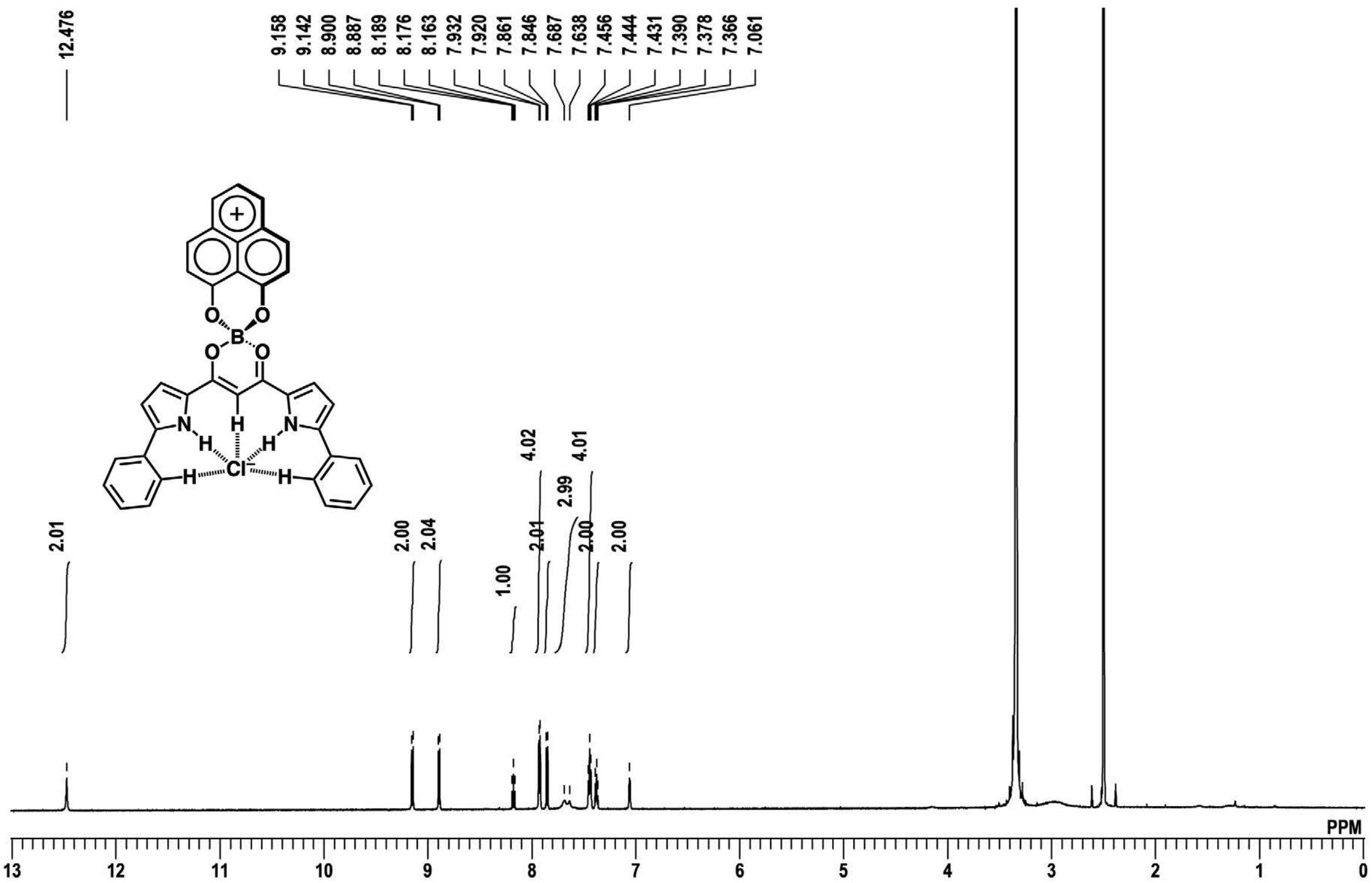

Fig. S6 ^{1}H NMR spectrum of **3b**$^{+}$-Cl^{-} in DMSO-d_6.

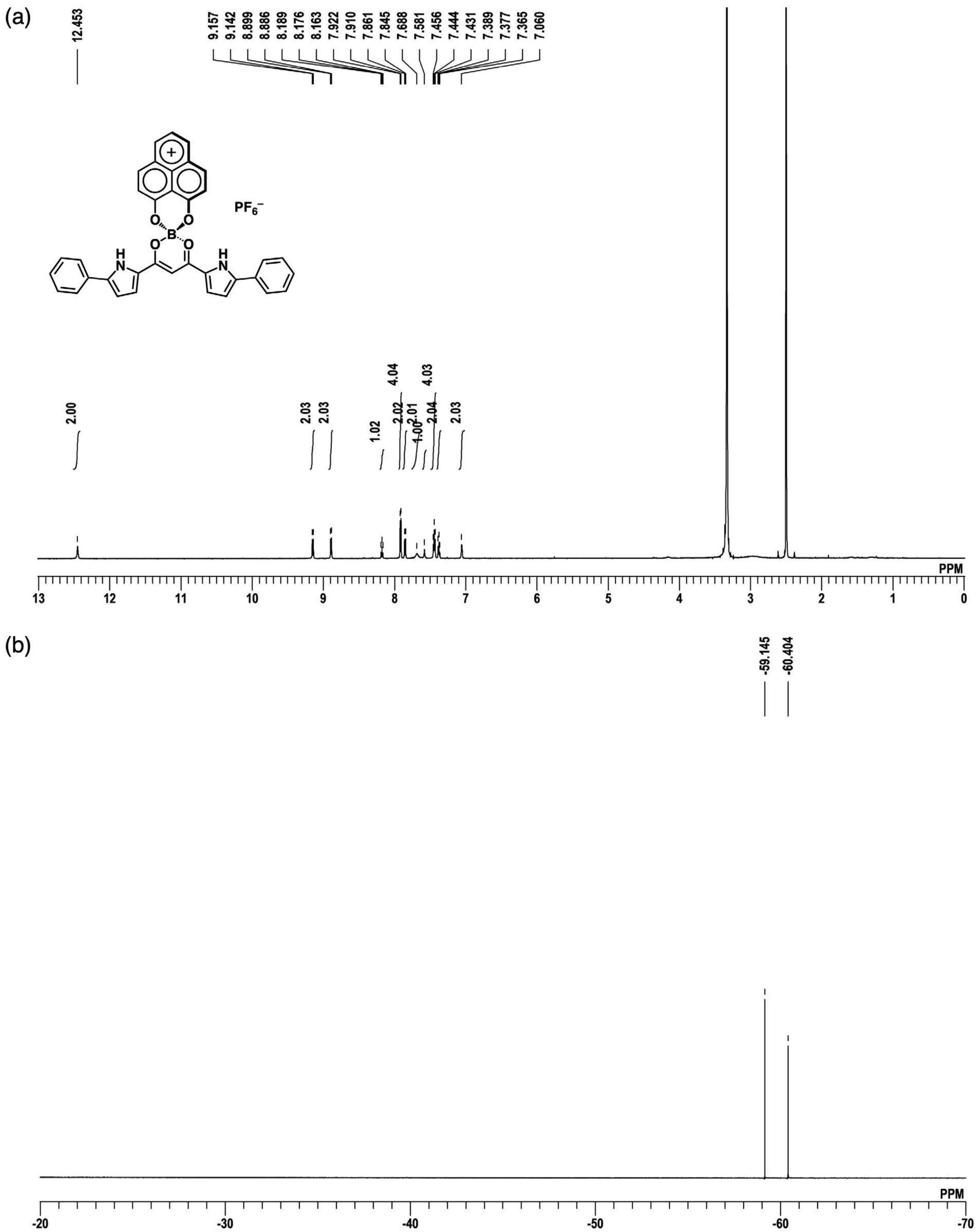

Fig. S7 (a) ^{1}H NMR and (b) ^{19}F NMR spectra of **3b**$^+$-PF_6^- in DMSO-d_6.

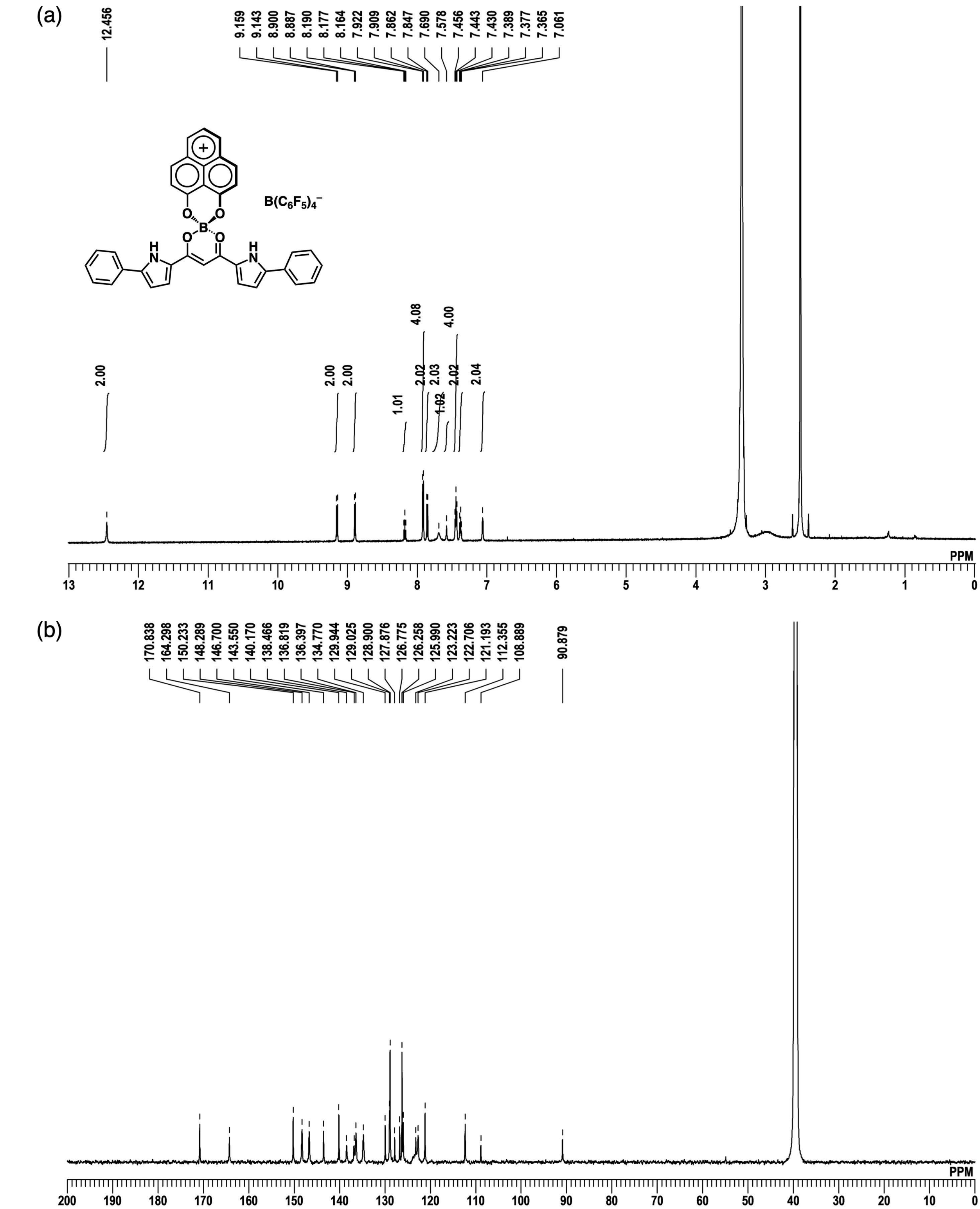

Fig. S8 (a) ^{1}H NMR, (b) ^{13}C{^{1}H} NMR, and (c) ^{19}F NMR spectra of **3b**$^{+}$-B$(C_6F_5)_4^{-}$ in DMSO-d_6.

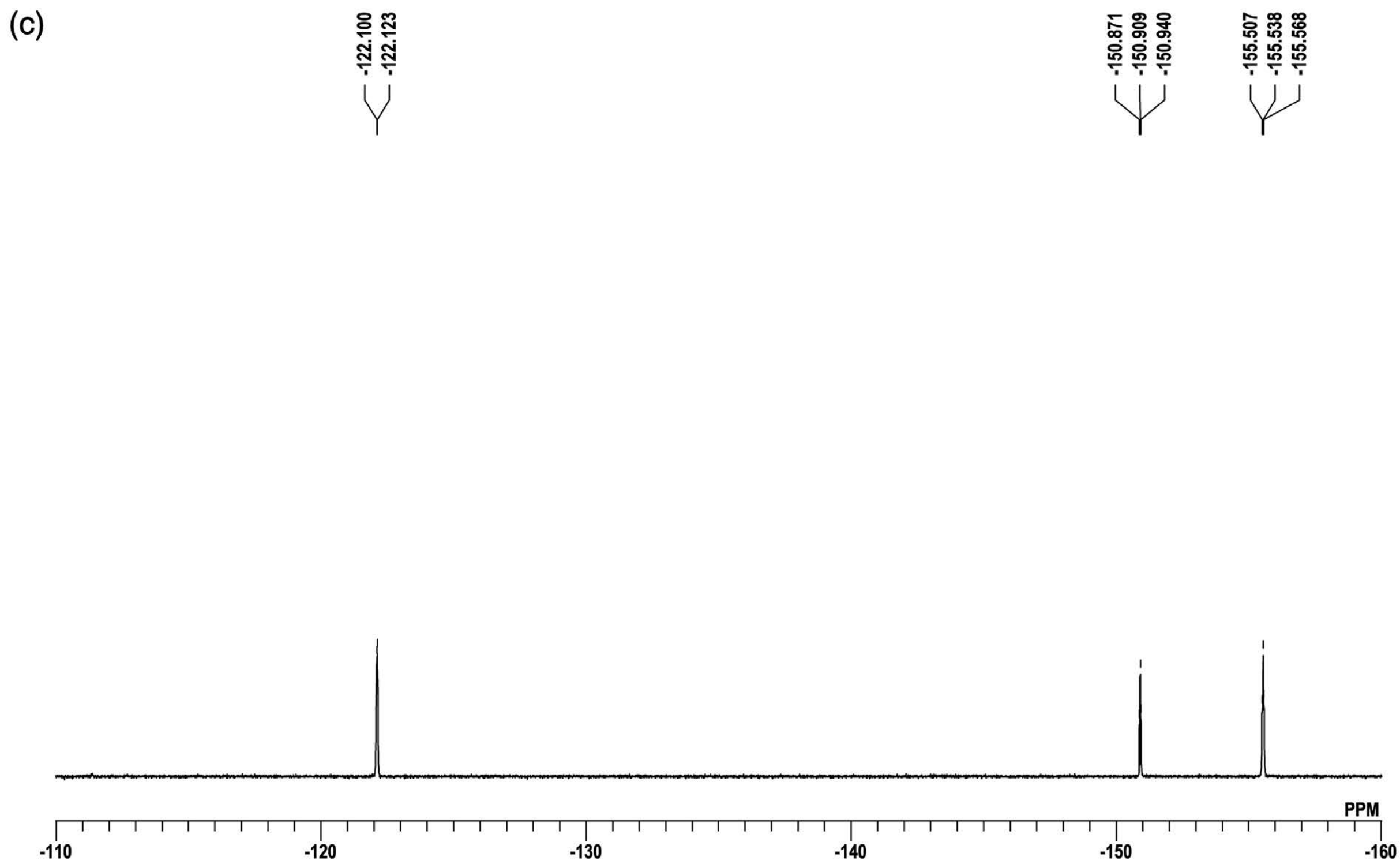

Fig. S8 (Continued)

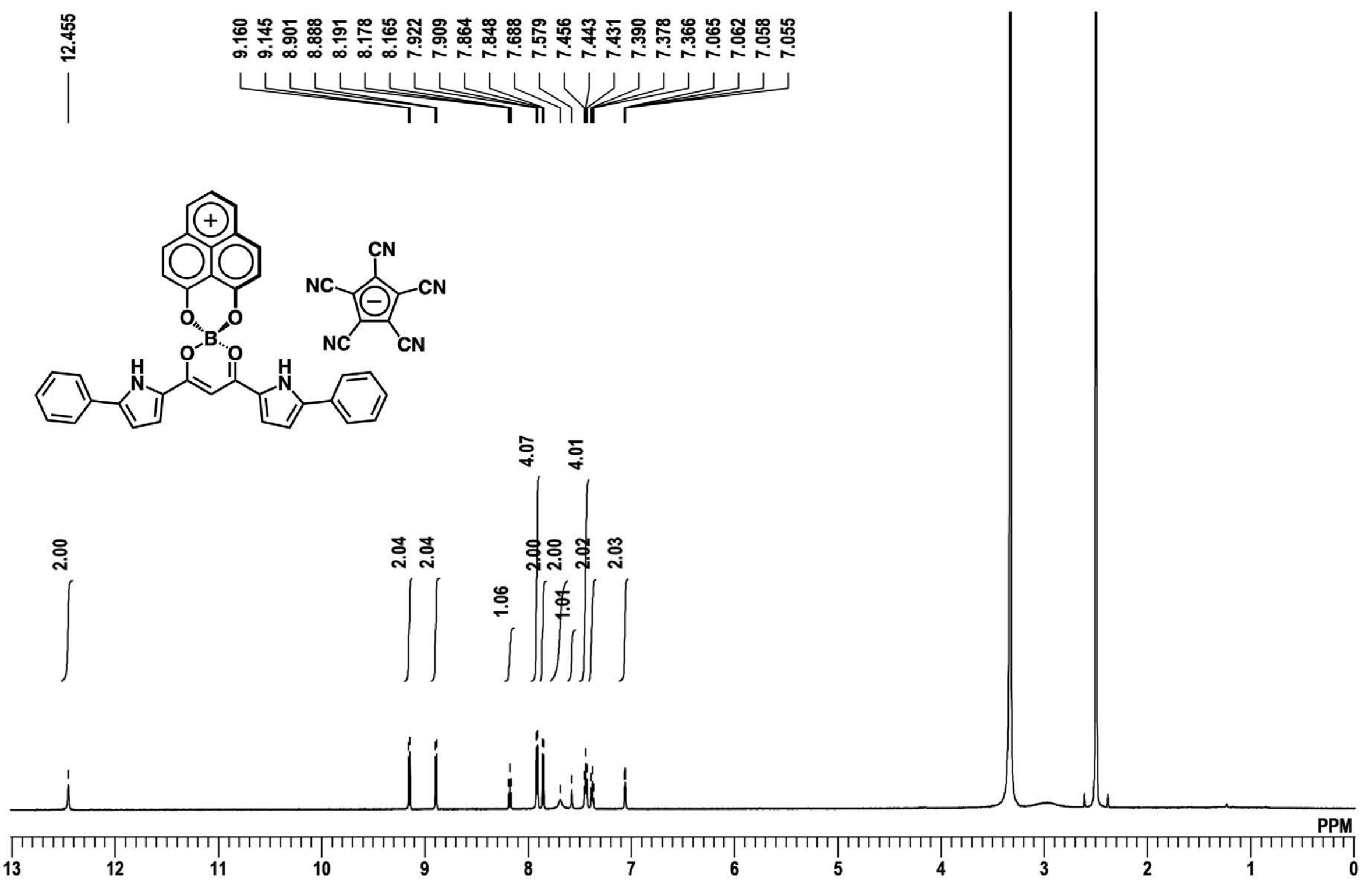

Fig. S9 ^{1}H NMR spectrum of **3b**$^{+}$-PCCp^{-} in DMSO-d_6.

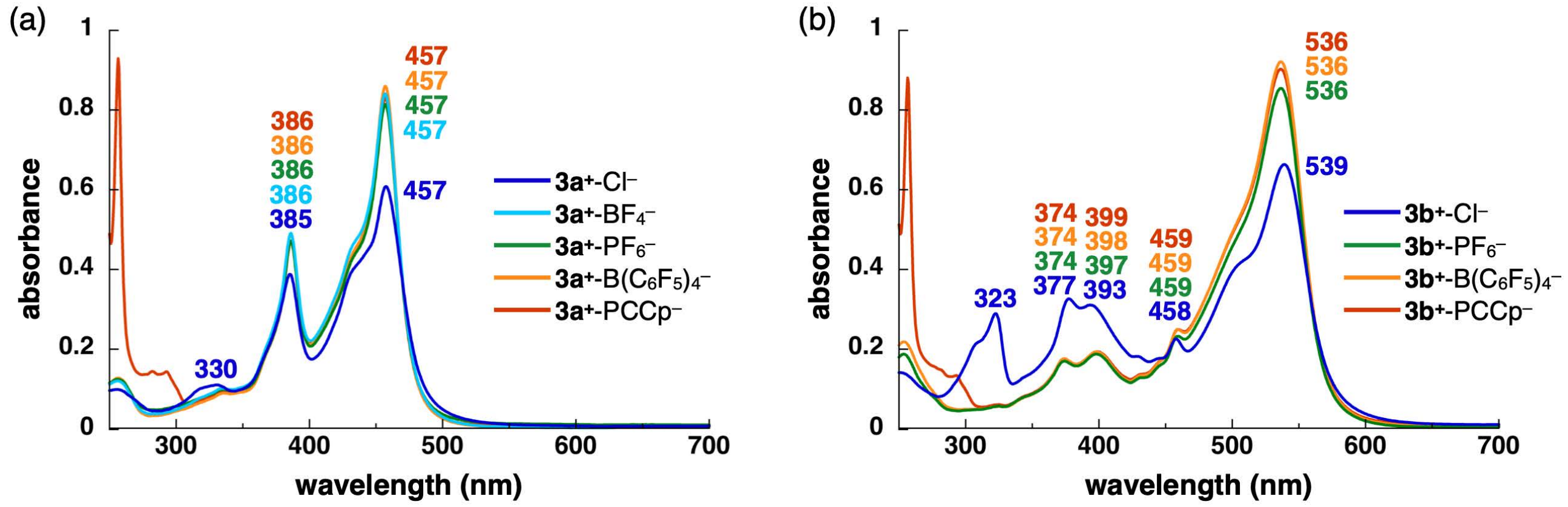

Fig. S10 UV/vis absorption spectra in CH_2Cl_2 (0.01 mM): (a) **3a**$^+$-Cl^- (blue), **3a**$^+$-BF_4^- (light blue), **3a**$^+$-PF_6^- (light green), **3a**$^+$-$B(C_6F_5)_4^-$ (orange), and **3a**$^+$-$PCCp^-$ (red) and (b) **3b**$^+$-Cl^- (blue), **3b**$^+$-PF_6^- (light green), **3b**$^+$-$B(C_6F_5)_4^-$ (orange), and **3b**$^+$-$PCCp^-$ (red).

2. X-ray crystallographic data

Method for single-crystal X-ray analysis. Crystallographic data are summarized in Table S1. A single crystal of **3a**$^+$-BF_4^- was obtained by vapor diffusion of *n*-hexane into an acetone solution. The data crystal was an orange prism of approximate dimensions 0.050 mm × 0.050 mm × 0.005 mm. A single crystal of **3a**$^+$-PF_6^- was obtained by vapor diffusion of *n*-hexane into a THF/MeOH solution. The data crystal was an orange prism of approximate dimensions 0.080 mm × 0.030 mm × 0.005 mm. A single crystal of **3a**$^+$-$B(C_6F_5)_4^-$ was obtained by vapor diffusion of water into an CH_3CN solution. The data crystal was an orange prism of approximate dimensions 0.30 mm × 0.05 mm × 0.05 mm. A single crystal of **3a**$^+$-PCCp$^-$ was obtained by vapor diffusion of *n*-hexane into a CH_2Cl_2 solution. The data crystal was an orange prism of approximate dimensions 0.30 mm × 0.05 mm × 0.05 mm. A single crystal of **3b**$^+$-Cl^- was obtained by vapor diffusion of *n*-hexane into a CH_2Cl_2/MeOH/C_6H_5CN solution. The data crystal was a red needle of approximate dimensions 0.05 mm × 0.01 mm × 0.01 mm. A single crystal of **3b**$^+$-PF_6^- was obtained by vapor diffusion of *n*-hexane into a CH_2Cl_2/2-methylpyridine solution. The data crystal was an orange prism of approximate dimensions 0.05 mm × 0.03 mm × 0.02 mm. A single crystal of **3b**$^+$-$B(C_6F_5)_4^-$ was obtained by vapor diffusion of *n*-hexane into a CH_2Cl_2/2-methylpyridine solution. The data crystal was an orange prism of approximate dimensions 0.02 mm × 0.02 mm × 0.01 mm. A single crystal of **3b**$^+$-PCCp$^-$ was obtained by vapor diffusion of *n*-hexane into a CH_2Cl_2/2,6-lutidine solution. The data crystal was an orange prism of approximate dimensions 0.05 mm × 0.02 mm × 0.02 mm. The data of **3a**$^+$-BF_4^- and **3a**$^+$-PF_6^- were collected at 100 K on a Rigaku Pliatus 3 CdTe 1M with Si (311) monochromated synchrotron radiation (λ = 0.4140 Å) at BL02B1 (SPring-8),[S5] whereas those of **3a**$^+$-$B(C_6F_5)_4^-$, **3b**$^+$-Cl^-, **3b**$^+$-PF_6^-, **3b**$^+$-$B(C_6F_5)_4^-$, and **3b**$^+$-PCCp$^-$ were collected at 100 K except for **3a**$^+$-$B(C_6F_5)_4^-$ (90 K) on a Dectris EIGER X 1M diffractometer with Si (111) monochromated synchrotron radiation (λ = 0.8120 Å except for **3a**$^+$-$B(C_6F_5)_4^-$ (0.8105 Å)) at BL40XU (SPring-8).[S6] The data of **3a**$^+$-PCCp$^-$ was collected at 90 K on a Bruker D8 Venture diffractometer with MoKα radiation (λ = 0.71073 Å) focused by multilayer confocal mirror. All the structures were solved by dual-space method. The structures were refined by a full-matrix least-squares method by using a SHELXL 2014[S7] (Yadokari-XG).[S8] In each structure, the non-hydrogen atoms were refined anisotropically. CIF files (CCDC-2561908–2561915) can be obtained free of charge from the Cambridge Crystallographic Data Centre via www.ccdc.cam.ac.uk/data_request/cif.

Table S1 Crystallographic details.

	**3a**$^+$-BF_4^-	**3a**$^+$-PF_6^-	**3a**$^+$-$B(C_6F_5)_4^-$	**3a**$^+$-PCCp$^-$	**3b**$^+$-Cl^-
formula	$C_{24}H_{16}BN_2O_4 \cdot BF_4 \cdot C_3H_6O$	$C_{24}H_{16}BN_2O_4 \cdot F_6P \cdot CH_4O$	$C_{24}H_{16}BN_2O_4 \cdot C_{24}BF_{20} \cdot 2C_2H_3N$	$C_{24}H_{16}BN_2O_4 \cdot C_{10}N_5$	$C_{36}H_{24}BN_2O_4 \cdot Cl \cdot C_6H_{14}$
fw	552.08	584.21	1168.35	597.35	681.00
crystal size, mm	0.050 × 0.050 × 0.005	0.080 × 0.030 × 0.005	0.03 × 0.03 × 0.01	0.30 × 0.05 × 0.05	0.05 × 0.01 × 0.01
crystal system	orthorhombic	monoclinic	monoclinic	monoclinic	monoclinic
space group	*P*ca2$_1$ (no. 29)	*P*2$_1$/*c* (no. 14)	*P*2$_1$ (no. 4)	*P*2$_1$/*c* (no. 14)	*P*2$_1$ (no. 4)
a, Å	15.42(2)	10.32(6)	15.5600(2)	11.0231(17)	11.8621(4)
b, Å	9.247(8)	8.91(6)	9.16930(10)	9.0967(11)	9.7431(3)
c, Å	17.48(3)	27.64(16)	17.4534(3)	27.917(4)	13.9019(4)
α, °	90	90	90	90	90
β, °	90	90.82(3)	108.024(2)	94.513(5)	100.691(3)
γ, °	90	90	90	90	90
V, Å^3	2493(6)	2541(27)	2367.95(6)	2790.6(7)	1578.80(9)
ρ_{calcd}, g cm^{-3}	1.471	1.527	1.639	1.422	1.433
Z	4	4	2	4	2
T, K	90(2)	90(2)	90(2)	90(2)	100(2)
μ, mm^{-1}	0.046 ^{a}	0.061 ^{a}	0.216 ^{a}	0.097 ^{b}	0.239 ^{a}
no. of reflns	52265	69112	27799	48160	19531
no. of unique reflns	5690	5714	10336	4917	6793
variables	364	375	742	415	397
λ, Å	0.4140 ^{a}	0.4140 ^{a}	0.8105 ^{a}	0.71073 ^{b}	0.8120 ^{a}
R_1 ($I > 2\sigma(I)$)	0.0925	0.0750	0.0551	0.0368	0.0631
wR_2 ($I > 2\sigma(I)$)	0.1994	0.1891	0.1285	0.1031	0.1426
GOF	0.976	1.073	1.041	1.082	0.965

^{a} Synchrotron radiation. ^{b} MoKα radiation.

Table S1 (Continued)

	**3b**$^+$-PF_6^-	**3b**$^+$-$B(C_6F_5)_4^-$	**3b**$^+$-$PCCp^-$
formula	$C_{36}H_{24}BN_2O_4{\cdot}F_6P{\cdot}2C_7H_9N{\cdot}CH_2Cl_2$	$C_{36}H_{24}BN_2O_4{\cdot}C_{24}BF_{20}{\cdot}2C_6H_7N$	$C_{36}H_{24}BN_2O_4{\cdot}C_{10}N_5{\cdot}2C_7H_9N{\cdot}2C_6H_{14}$
fw	1003.58	1424.68	1136.18
crystal size, mm	0.05 × 0.03 × 0.02	0.02 × 0.02 × 0.01	0.05 × 0.02 × 0.02
crystal system	triclinic	monoclinic	triclinic
space group	$P\bar{1}$ (no. 2)	$P2_1/c$ (no. 14)	$P\bar{1}$ (no. 2)
a, Å	12.8203(5)	10.71440(10)	13.6103(14)
b, Å	14.1922(6)	34.8332(2)	14.0011(17)
c, Å	14.5514(7)	16.94180(10)	18.6511(16)
α, °	106.659(4)	90	97.329(8)
β, °	104.819(4)	104.2060(10)	108.752(8)
γ, °	103.470(3)	90	107.041(10)
V, Å^3	2314.16(19)	6129.60(8)	3119.7(6)
ρ_{calcd}, g cm^{-3}	1.440	1.544	1.210
Z	2	4	2
T, K	100(2)	100(2)	100(2)
μ, mm^{-1}	0.354 [a]	0.188 [a]	0.100 [a]
no. of reflns	28387	74535	36895
no. of unique reflns	10448	14327	13936
variables	700	917	760
λ, Å	0.8120 [a]	0.8120 [a]	0.8120 [a]
R_1 ($I > 2\sigma(I)$)	0.0619	0.0498	0.1310
wR_2 ($I > 2\sigma(I)$)	0.1659	0.1404	0.3278
GOF	1.034	1.106	0.924

[a] Synchrotron radiation. [b] MoKα radiation.

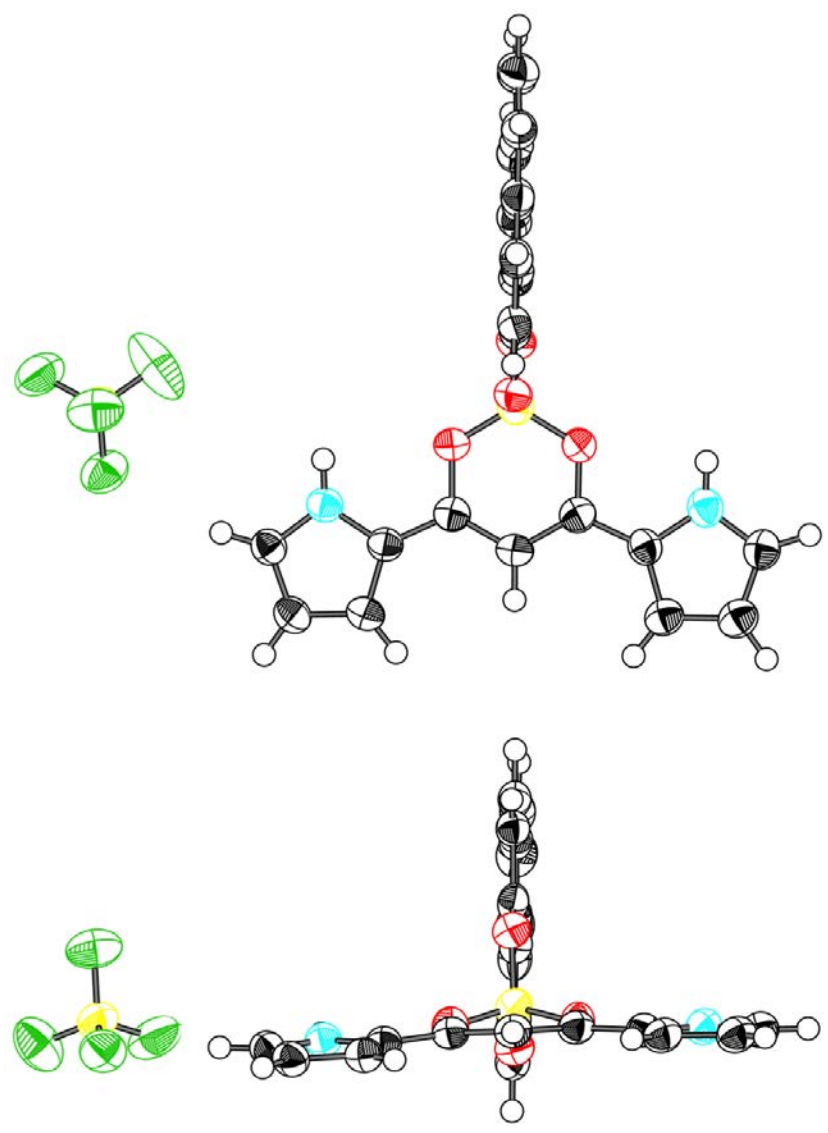

Fig. S11 Ortep drawing of single-crystal X-ray structure (top and side views) of **3a**$^+$-BF_4^-. Thermal ellipsoids are scaled to the 50% probability level. Solvent molecules are omitted for clarity. Atom color code: black, white (sphere), yellow, blue, red, and green refer to carbon, hydrogen, boron, nitrogen, oxygen, and fluorine, respectively.

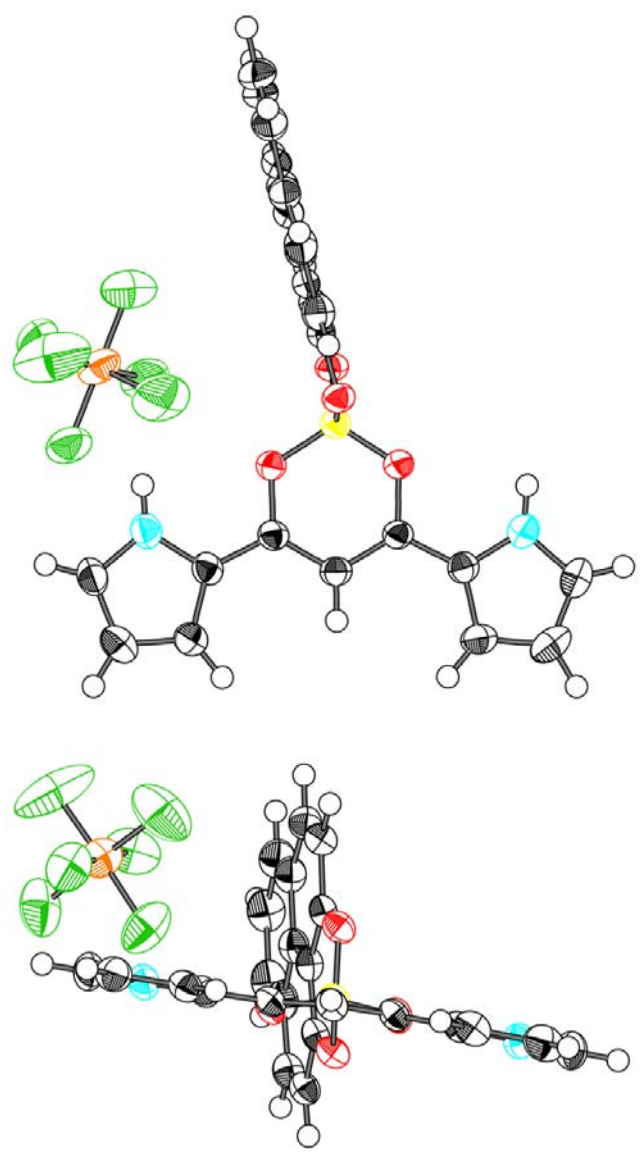

Fig. S12 Ortep drawing of single-crystal X-ray structure (top and side views) of **3a**$^+$-PF_6^-. Thermal ellipsoids are scaled to the 50% probability level. Solvent molecules are omitted for clarity. Atom color code: black, white (sphere), yellow, blue, red, green, and tangerine refer to carbon, hydrogen, boron, nitrogen, oxygen, fluorine, and phosphorus, respectively.

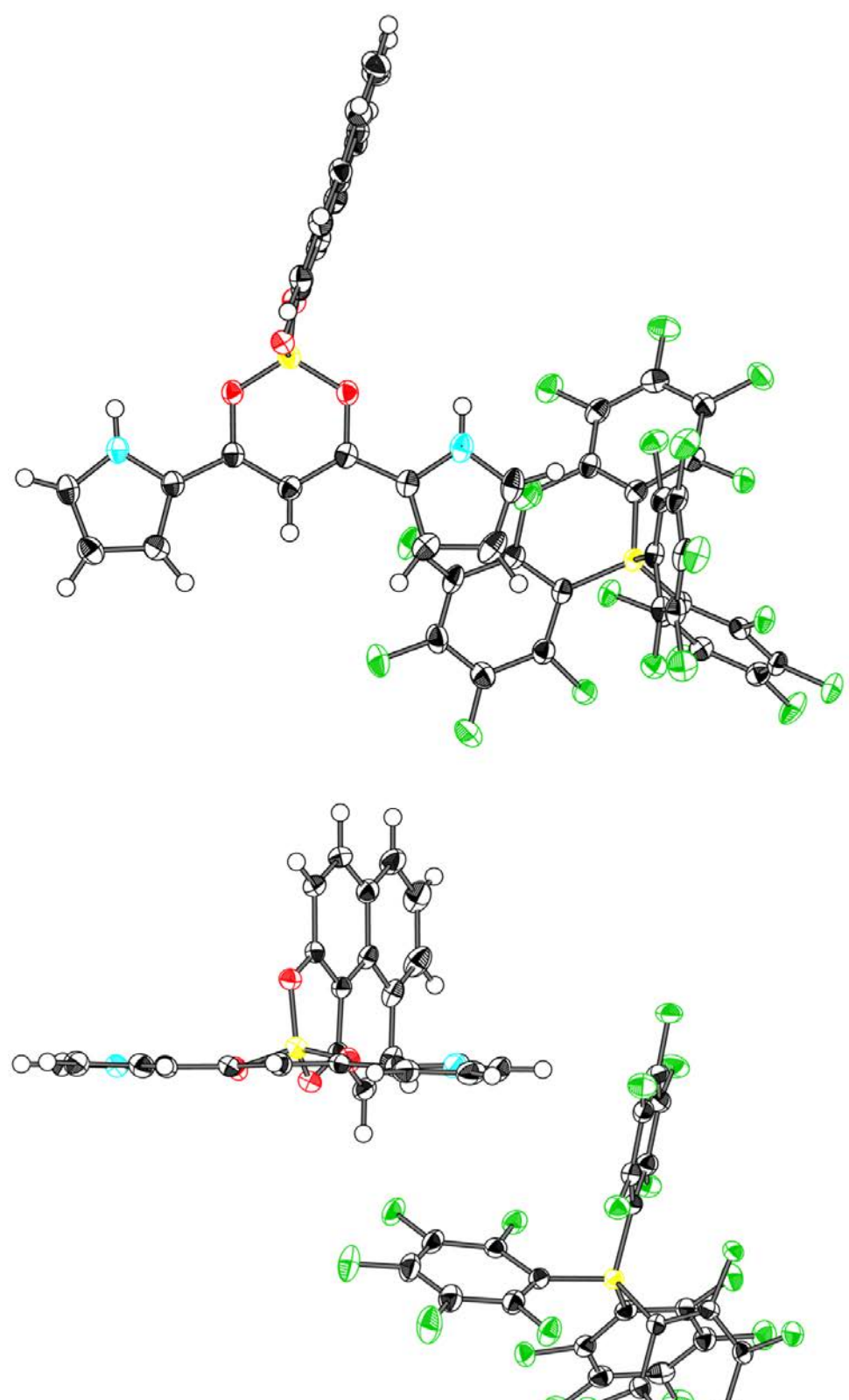

Fig. S13 Ortep drawing of single-crystal X-ray structure (top and side views) of **3a**$^+$-$B(C_6F_5)_4^-$. Thermal ellipsoids are scaled to the 50% probability level. Solvent molecules are omitted for clarity. Atom color code: black, white (sphere), yellow, blue, red, and green refer to carbon, hydrogen, boron, nitrogen, oxygen, and fluorine, respectively.

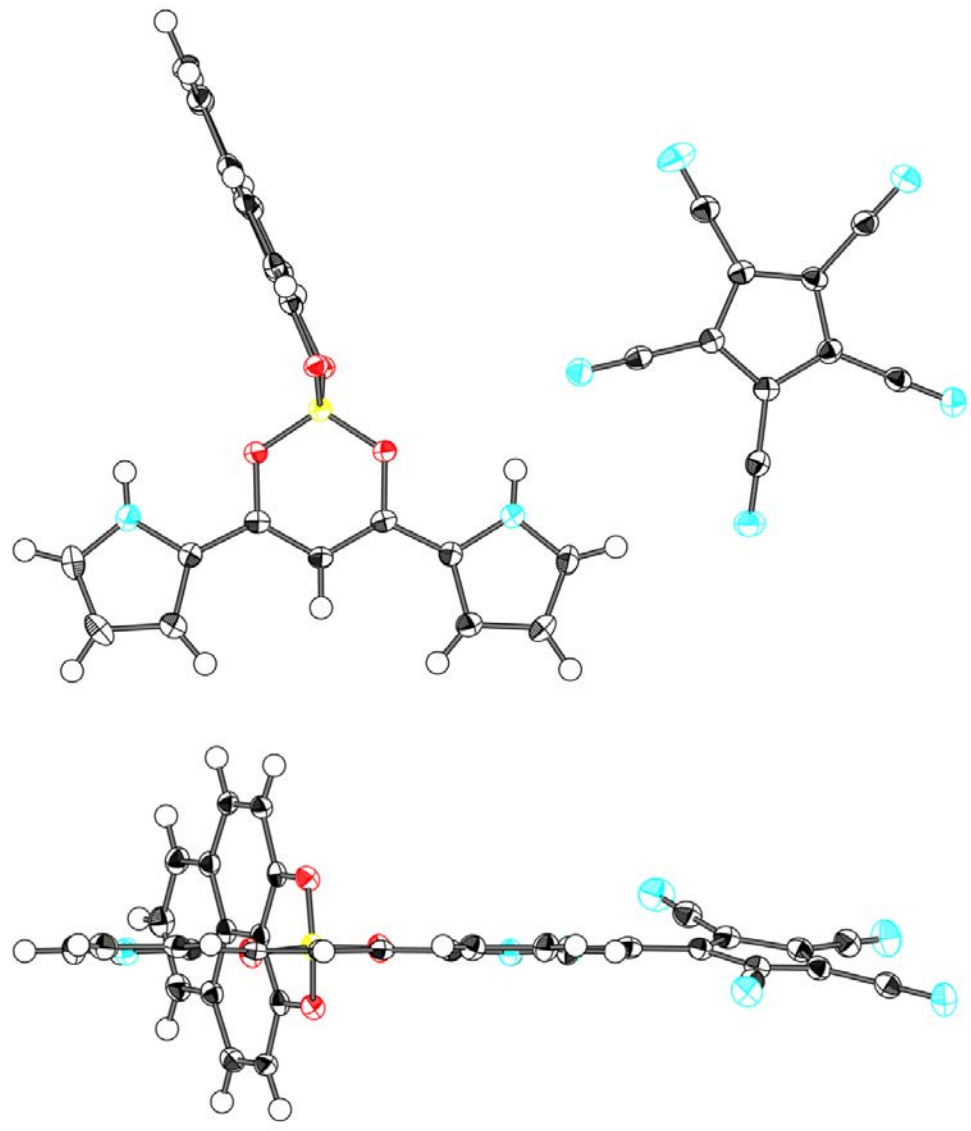

Fig. S14 Ortep drawing of single-crystal X-ray structure (top and side views) of **3a**$^+$-PCCp$^-$. Thermal ellipsoids are scaled to the 50% probability level. Atom color code: black, white (sphere), yellow, blue, and red refer to carbon, hydrogen, boron, nitrogen, and oxygen, respectively.

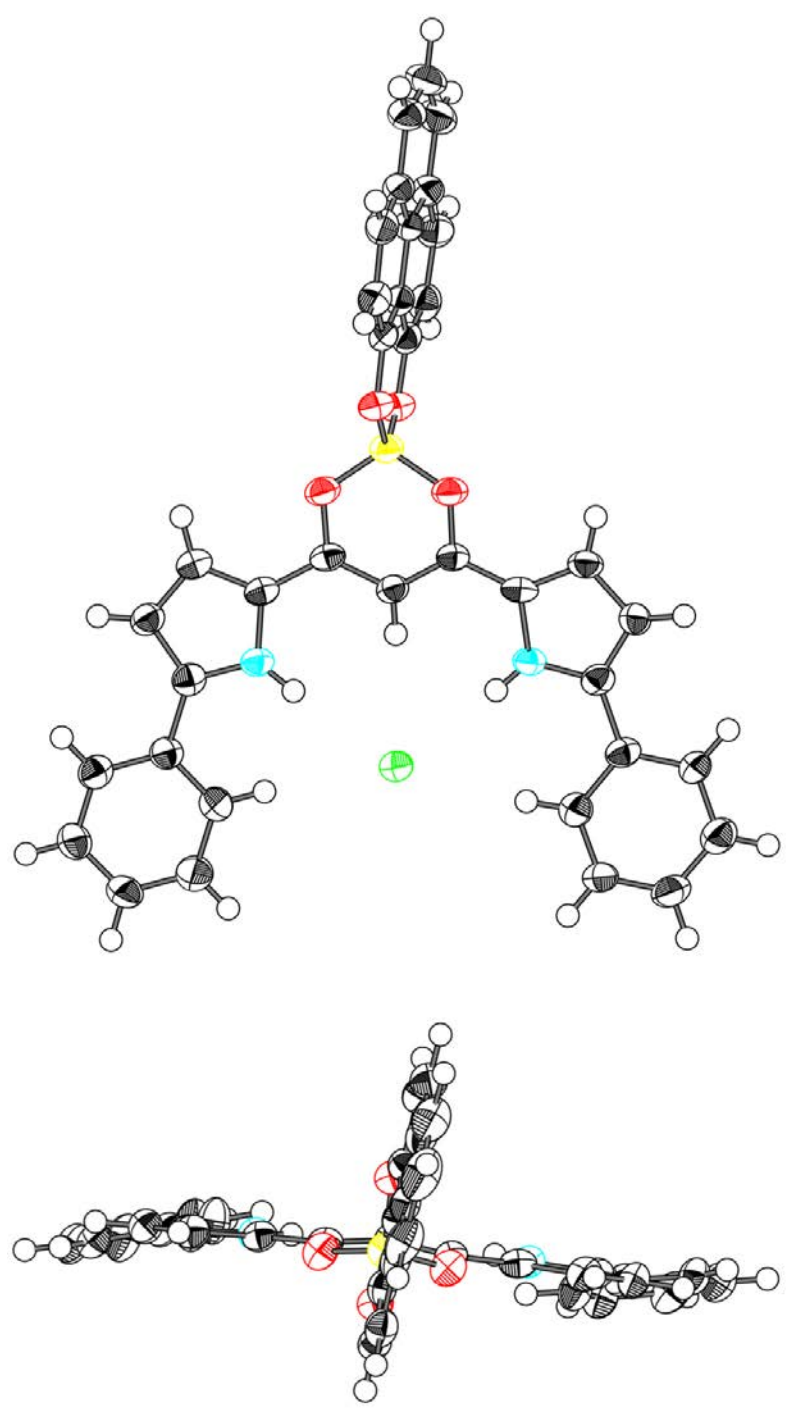

Fig. S15 Ortep drawing of single-crystal X-ray structure (top and side views) of **3b**$^+$-Cl$^-$. Thermal ellipsoids are scaled to the 50% probability level. Solvent molecules are omitted for clarity. Atom color code: black, white (sphere), yellow, blue, red, and yellow green refer to carbon, hydrogen, boron, nitrogen, oxygen, and chlorine respectively.

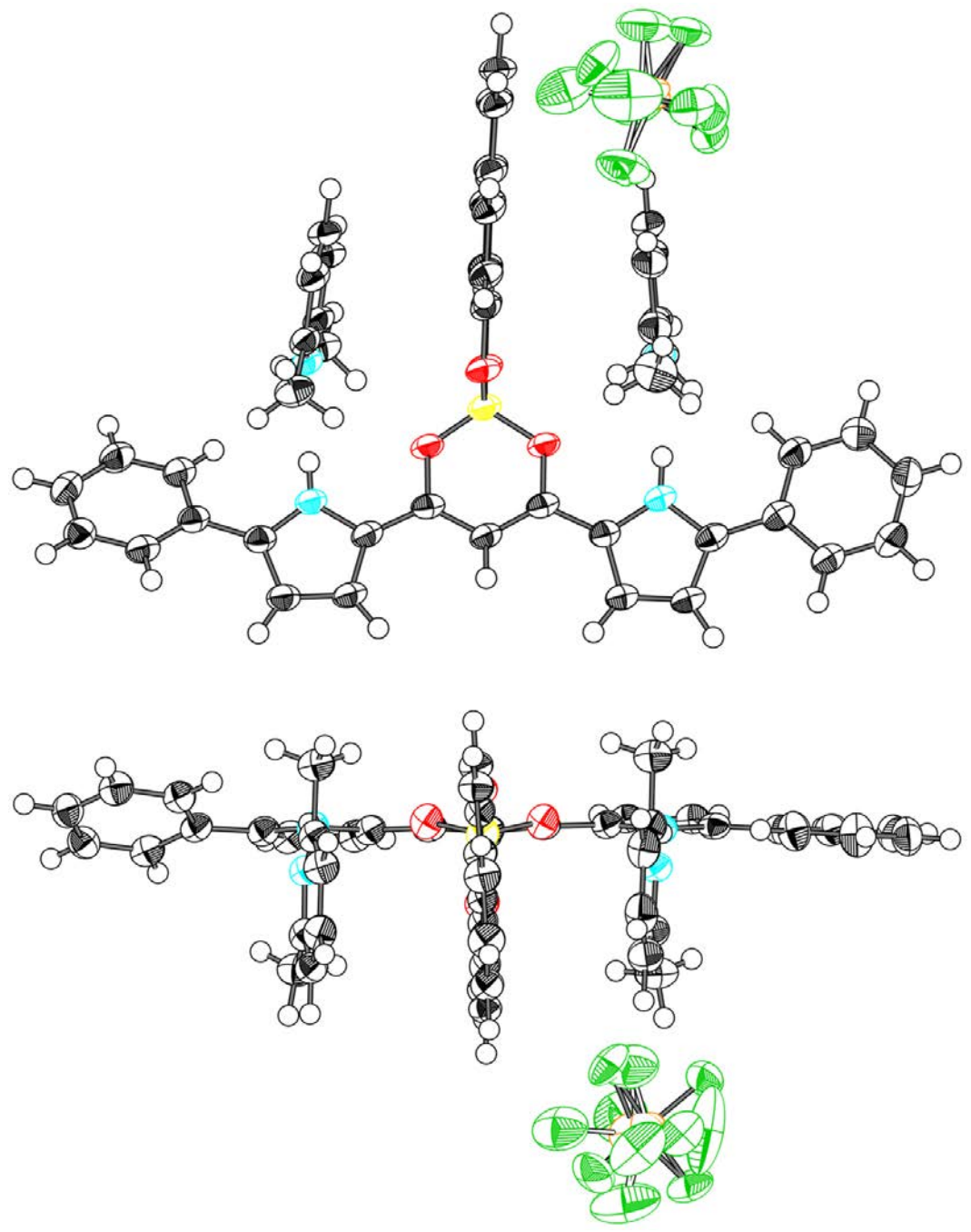

Fig. S16 Ortep drawing of single-crystal X-ray structure (top and side views) of **3b**$^+$-PF_6^-. Thermal ellipsoids are scaled to the 50% probability level. A PF_6^- has disordered structures in the ratio of 75 (gray bond) : 25 (white bond). Solvent molecules (CH_2Cl_2) are omitted for clarity. Atom color code: black, white (sphere), yellow, blue, red, green, and tangerine refer to carbon, hydrogen, boron, nitrogen, oxygen, fluorine, and phosphorus, respectively.

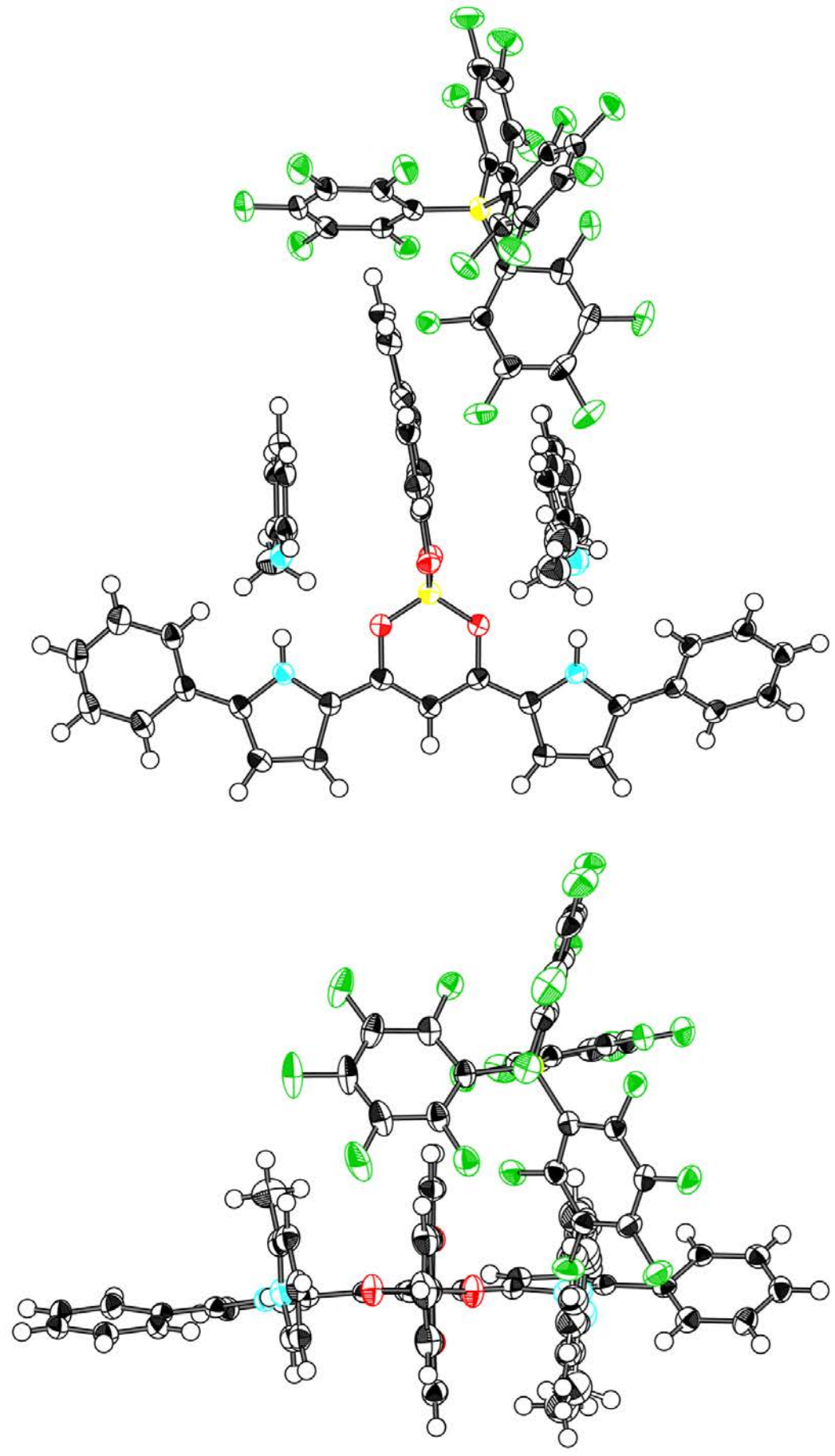

Fig. S17 Ortep drawing of single-crystal X-ray structure (top and side views) of **3b**$^+$-$B(C_6F_5)_4^-$. Thermal ellipsoids are scaled to the 50% probability level. A 2-picoline has disordered structures in the ratio of 60 (gray bond) : 40 (white bond). Atom color code: black, white (sphere), yellow, blue, red, and green refer to carbon, hydrogen, boron, nitrogen, oxygen, and fluorine, respectively.

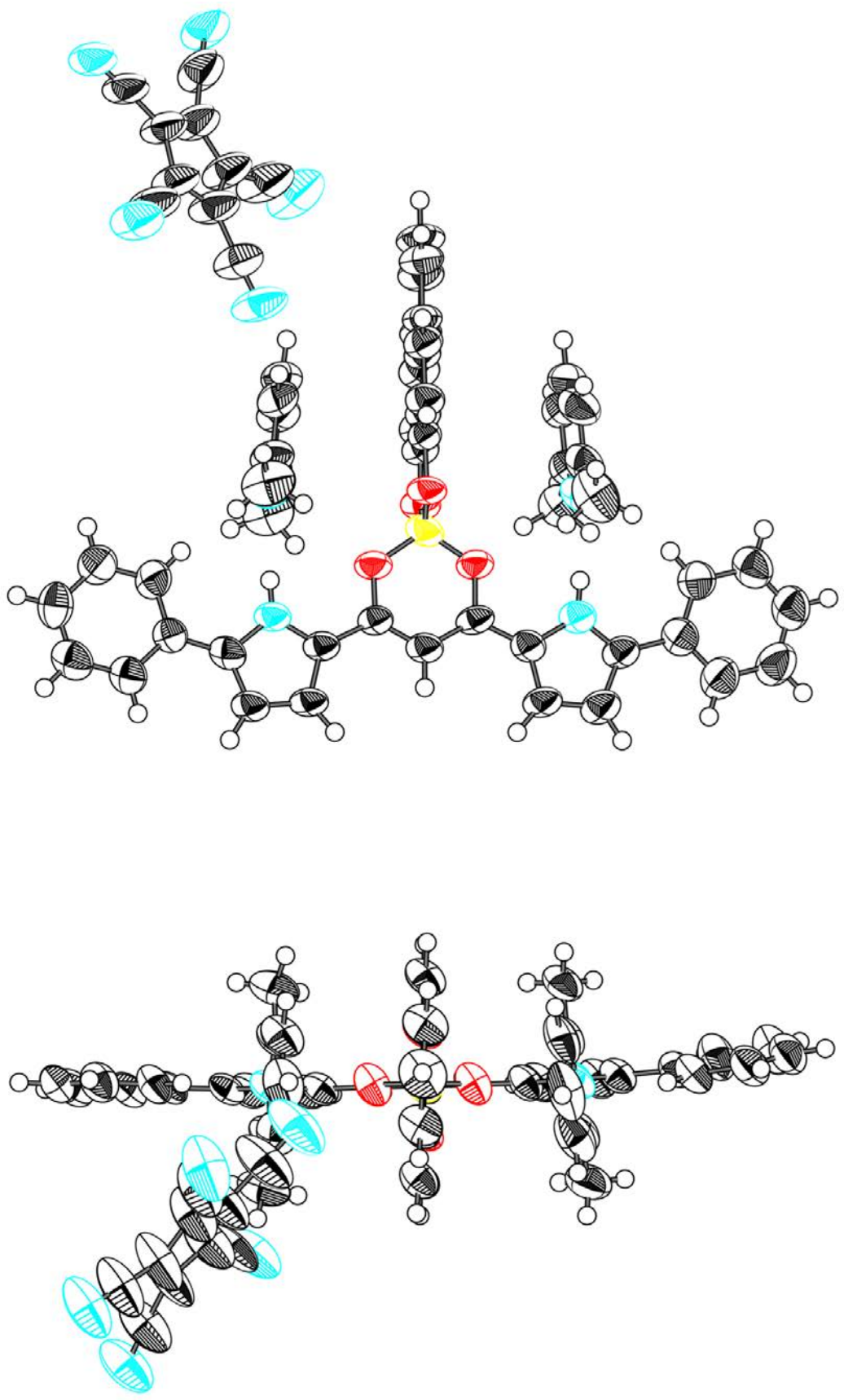

Fig. S18 Ortep drawing of single-crystal X-ray structure (top and side views) of **3b**$^{+}$-PCCp^{-}. Thermal ellipsoids are scaled to the 50% probability level. Solvent molecules (*n*-hexane) are omitted for clarity. Atom color code: black, white (sphere), yellow, blue, and red refer to carbon, hydrogen, boron, nitrogen, and oxygen, respectively.

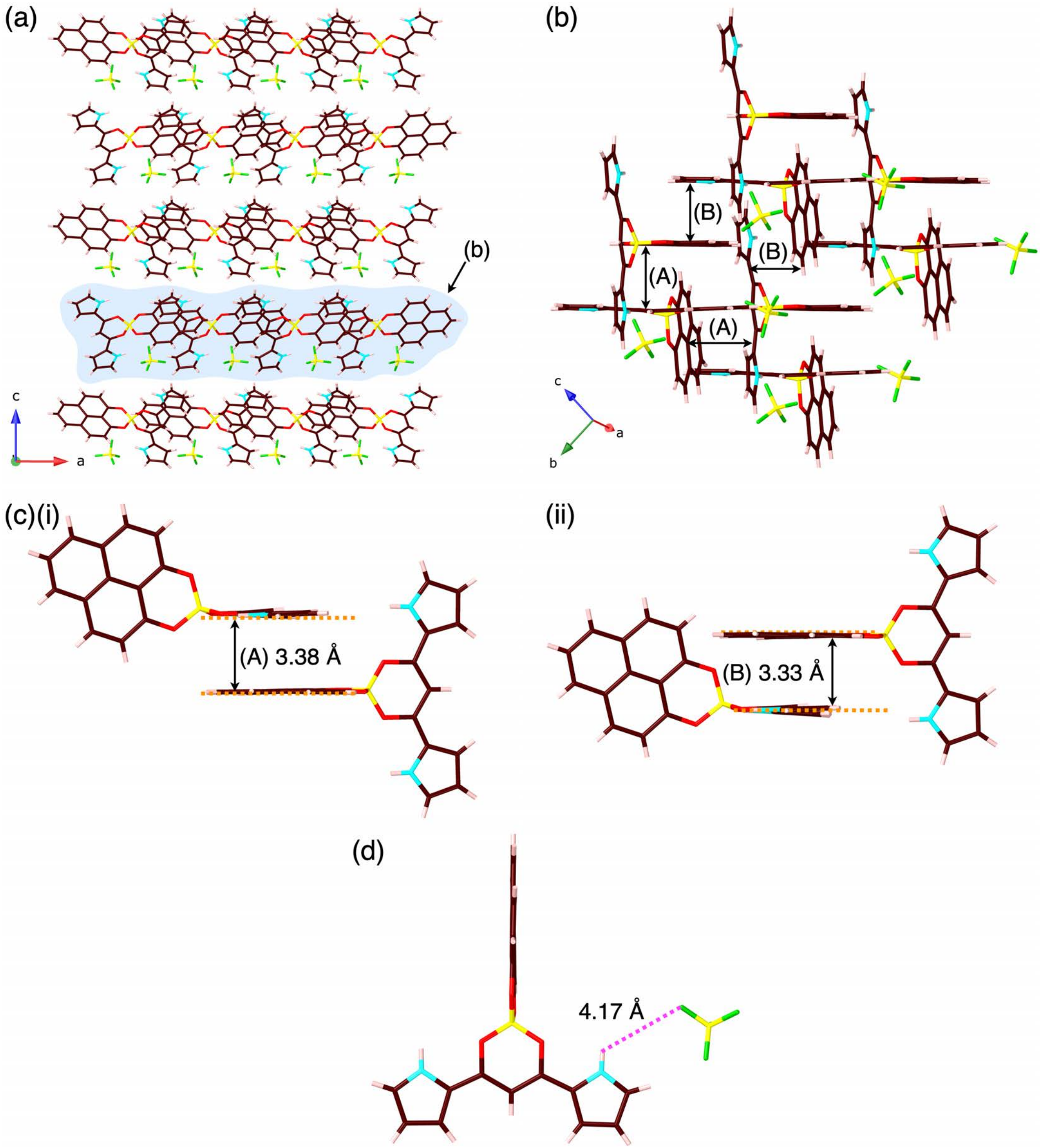

Fig. S19 Packing diagram (stacking assembly) of **3a**$^{+}$-BF_4^{-} as (a) top and (b) side views, (c) top views of the dimers, and (d) enlarged view of the ion pair. The distances between two **3a**$^{+}$ and the N(–H)···F distance are 3.38/3.33 and 4.17 Å, respectively. In this diagram, two planes are defined as (i) the phenalenyl-based plane (core 16 atoms including the boron atom) and (ii) the dipyrrolyldiketone-based plane (core 16 atoms including the boron atom). Solvent molecules are omitted for clarity. Atom color code: brown, pink, yellow, blue, red, and green refer to carbon, hydrogen, boron, nitrogen, oxygen, and fluorine, respectively.

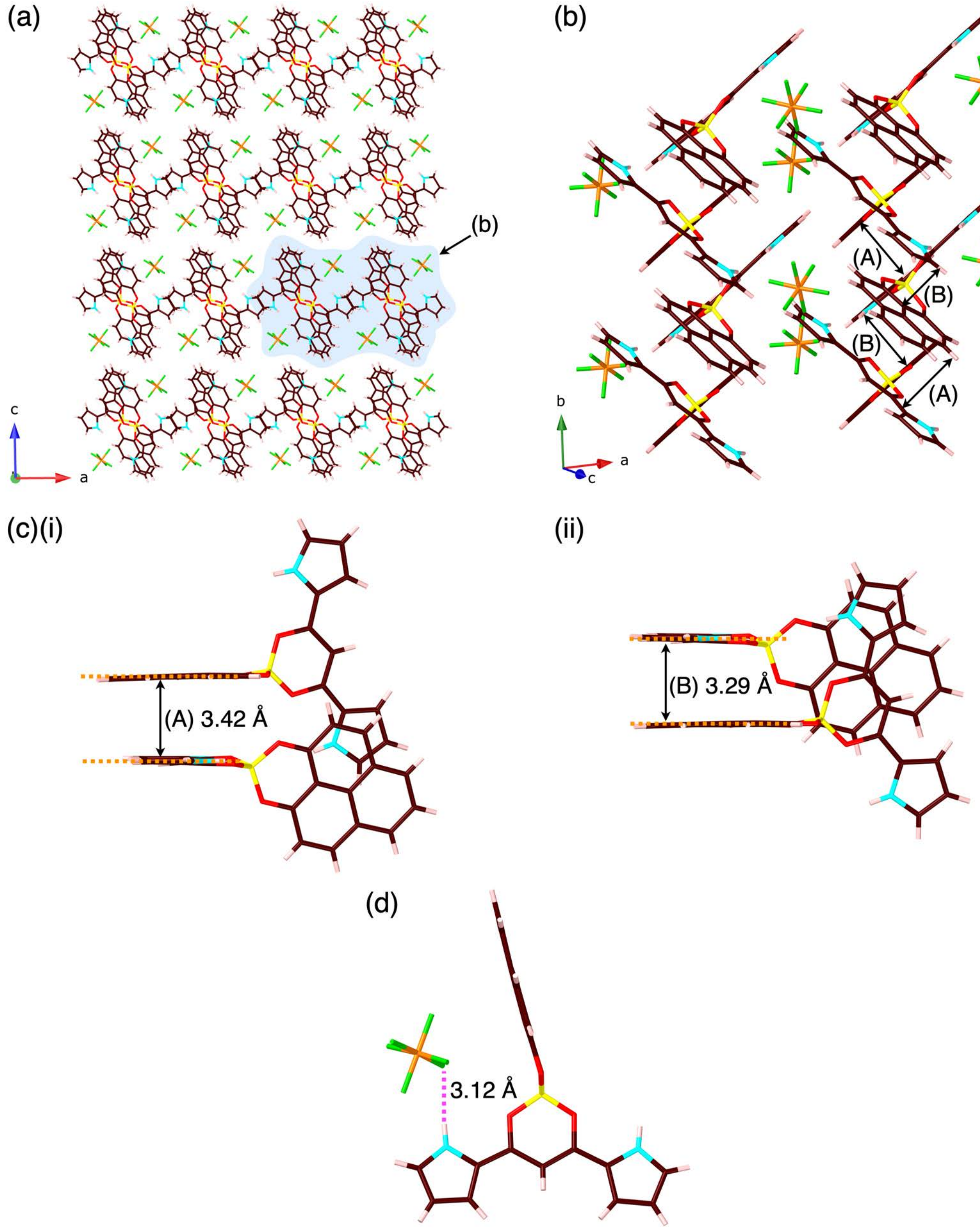

Fig. S20 Packing diagram (stacking assembly) of **3a**$^{+}$-PF_6^{-} as (a) top and (b) side views, ((c) top views of the dimers, and (d) enlarged view of the ion pair. The distances between two **3a**$^{+}$ and the N(–H)···F distance are 3.42/3.29 and 3.12/3.14 Å, respectively. In this diagram, two planes are defined as (i) the phenalenyl-based plane (core 16 atoms including the boron atom) and (ii) the dipyrrolyldiketone-based plane (core 16 atoms including the boron atom). Solvent molecules are omitted for clarity. Atom color code: brown, pink, yellow, blue, red, green, and tangerine refer to carbon, hydrogen, boron, nitrogen, oxygen, fluorine, and phosphorus, respectively.

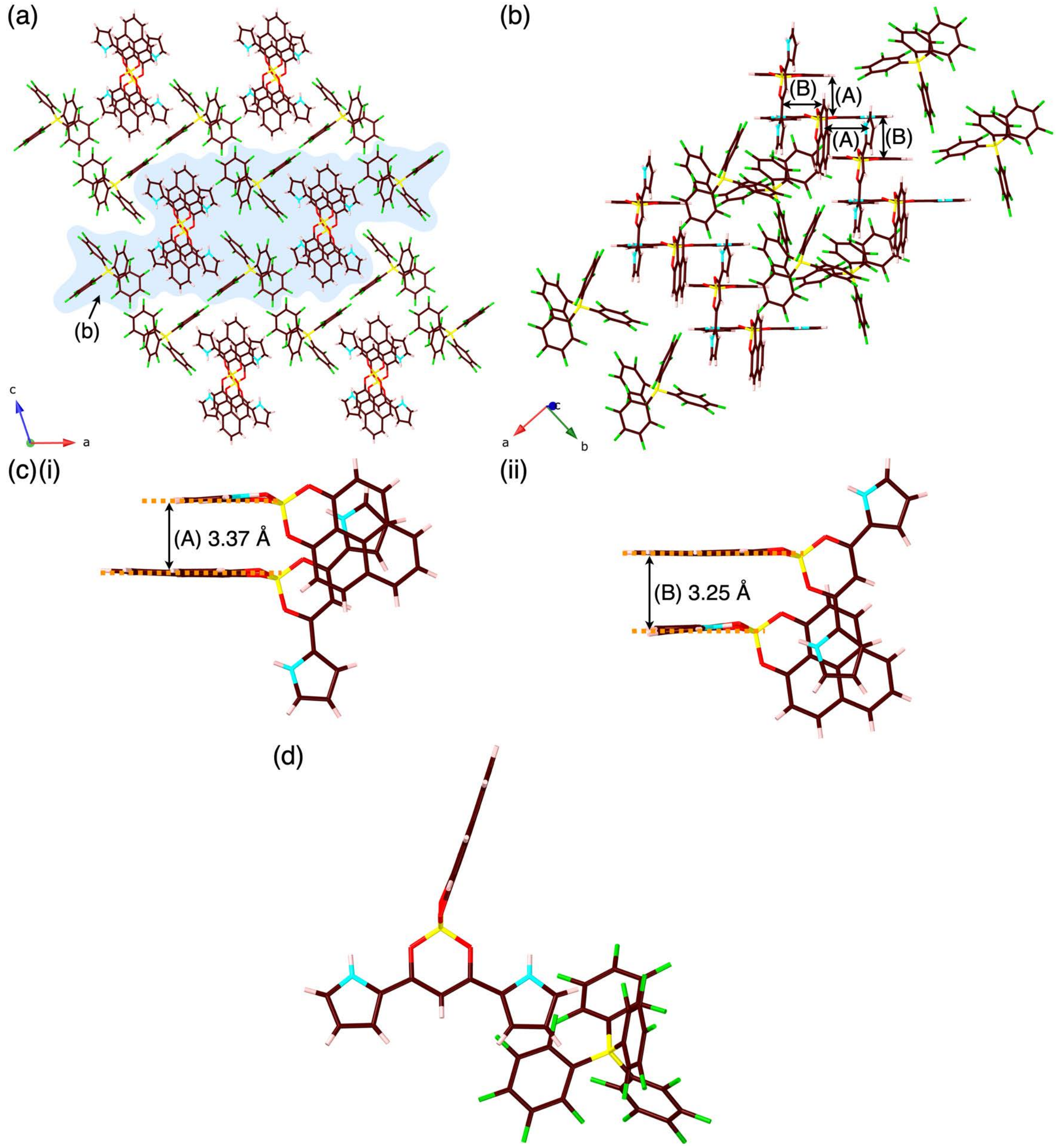

Fig. S21 Packing diagram (stacking assembly) of **3a**$^+$-B(C_6F_5)$_4^-$ as (a) top and (b) side views, (c) top views of the dimers, and (d) enlarged view of the ion pair. The distances between two **3a**$^+$ are 3.37/3.25 Å. In this diagram, two planes are defined as (i) the phenalenyl-based plane (core 16 atoms including the boron atom) and (ii) the dipyrrolyldiketone-based plane (core 16 atoms including the boron atom). Solvent molecules are omitted for clarity. Atom color code: brown, pink, yellow, blue, red, and green refer to carbon, hydrogen, boron, nitrogen, oxygen, and fluorine, respectively.

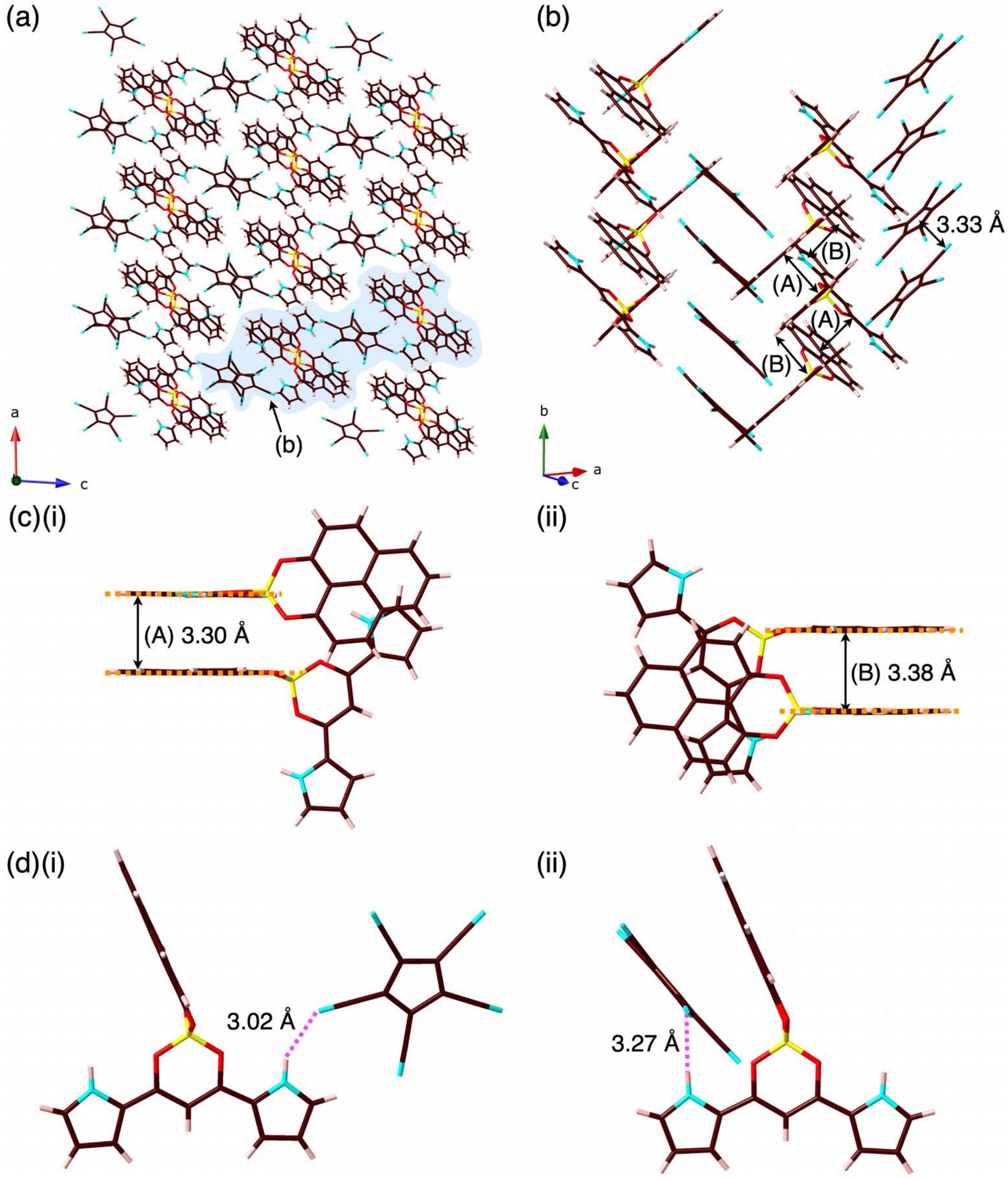

Fig. S22 Packing diagram (stacking assembly) of **3a**$^{+}$-PCCp^{-} as (a) top and (b) side views, (c) top views of the dimers, and (d) enlarged view of the ion pair. The distances between two **3a**$^{+}$, that between two PCCp^{-}, and the N(–H)···N distance are 3.30/3.38, 3.33, and 3.02/3.27 Å, respectively. In this diagram, two planes are defined as (i) the phenalenyl-based plane (core 16 atoms including the boron atom) and (ii) the dipyrrolyldiketone-based plane (core 16 atoms including the boron atom). Atom color code: brown, pink, yellow, blue, and red refer to carbon, hydrogen, boron, nitrogen, and oxygen, respectively.

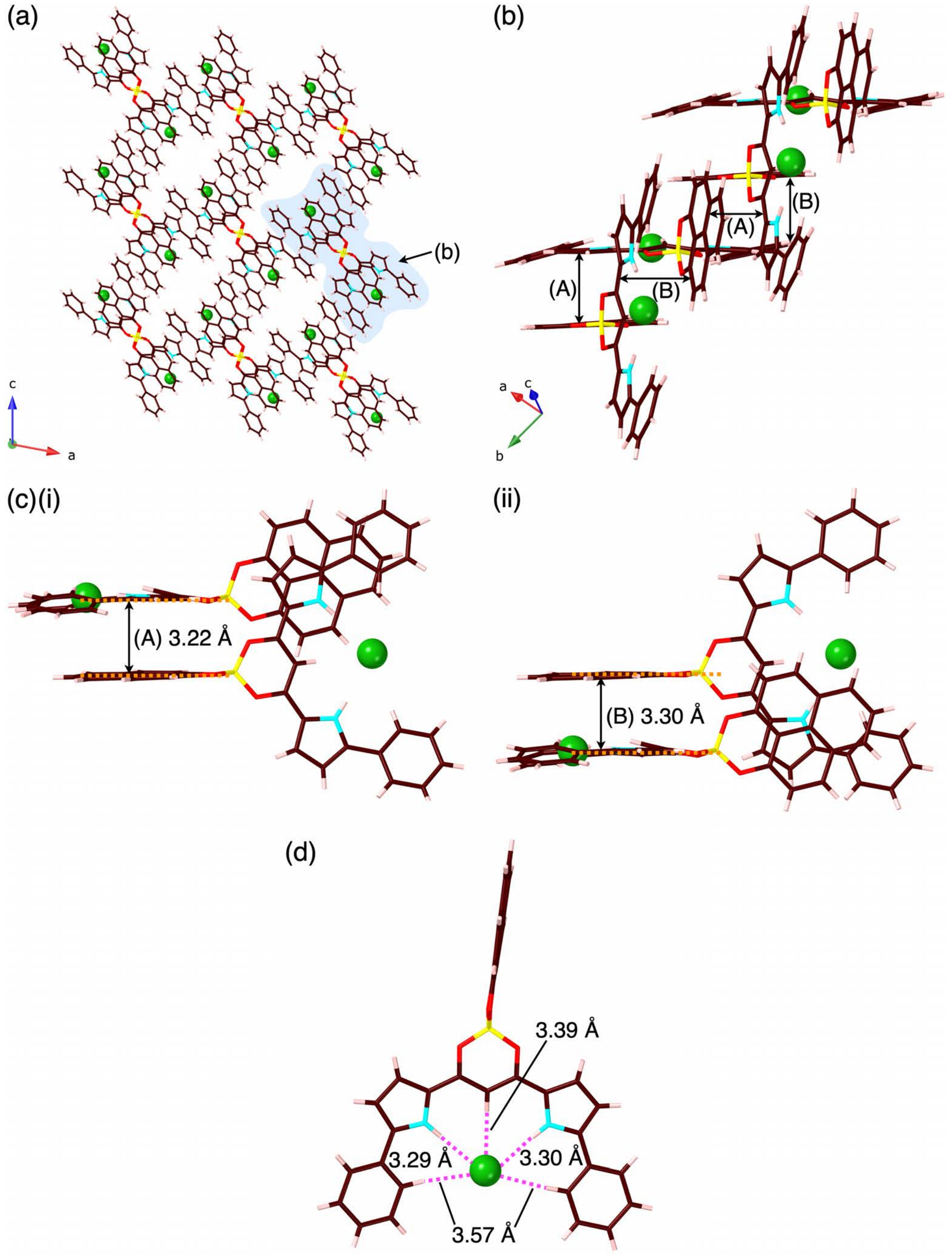

Fig. S23 Packing diagram (stacking assembly) of **3b**$^{+}$-Cl^{-} as (a) top and (b) side views, (c) top views of the dimers, and (d) enlarged view of the ion pair. The distances between two **3b**$^{+}$, the N(–H)···Cl^{-} distances, and the C(–H)···Cl^{-} distances are 3.22/3.30, 3.29/3.30, and 3.39/3.57 Å, respectively. In this diagram, two planes are defined as (i) the phenalenyl-based plane (core 16 atoms including the boron atom) and (ii) the dipyrrolyldiketone-based plane (core 29 atoms including the boron and chlorine atoms). Solvent molecules are omitted for clarity. Atom color code: brown, pink, yellow, blue, red, and green (sphere) refer to carbon, hydrogen, boron, nitrogen, oxygen, and chlorine, respectively.

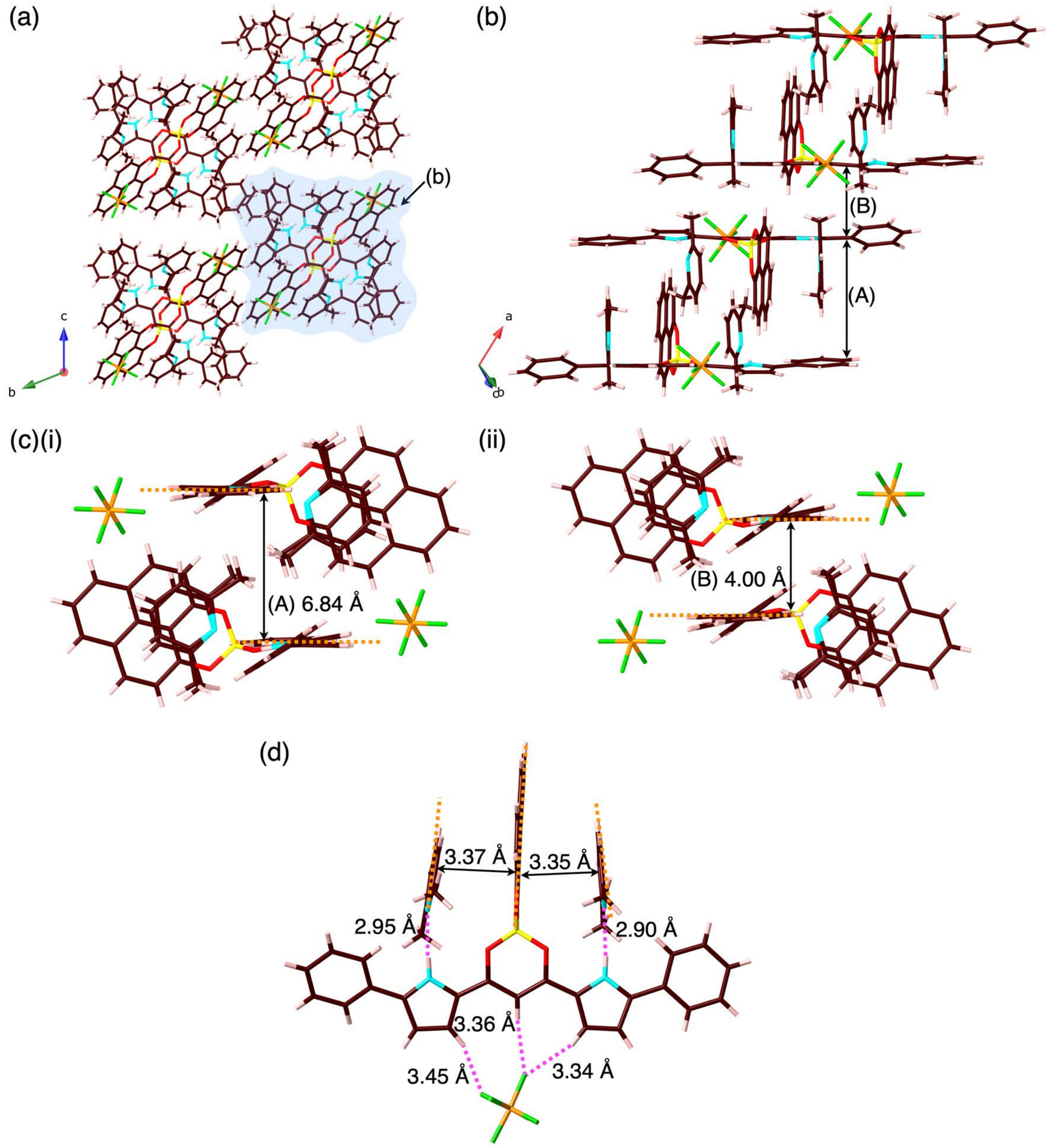

Fig. S24 Packing diagram (stacking assembly) of **3b**$^+$-PF_6^- as (a) top and (b) side views, (c) top views of the dimers, and (d) enlarged view of the ion pair. The distances between two **3b**$^+$, those between **3b**$^+$ and solvent, C(–H)···F, and the N(–H)···N distances are 6.84/4.00, 3.35/3.37, 3.34/3.36/3.45, and 2.90/2.95 Å, respectively. In this diagram, two planes are defined as (i) the phenalenyl-based plane (core 16 atoms including the boron atom) and (ii) the dipyrrolyldiketone-based plane (core 28 atoms including the boron atom). Solvent molecules (CH_2Cl_2) are omitted for clarity. Atom color code: brown, pink, yellow, blue, red, green, and tangerine refer to carbon, hydrogen, boron, nitrogen, oxygen, fluorine, and phosphorus, respectively.

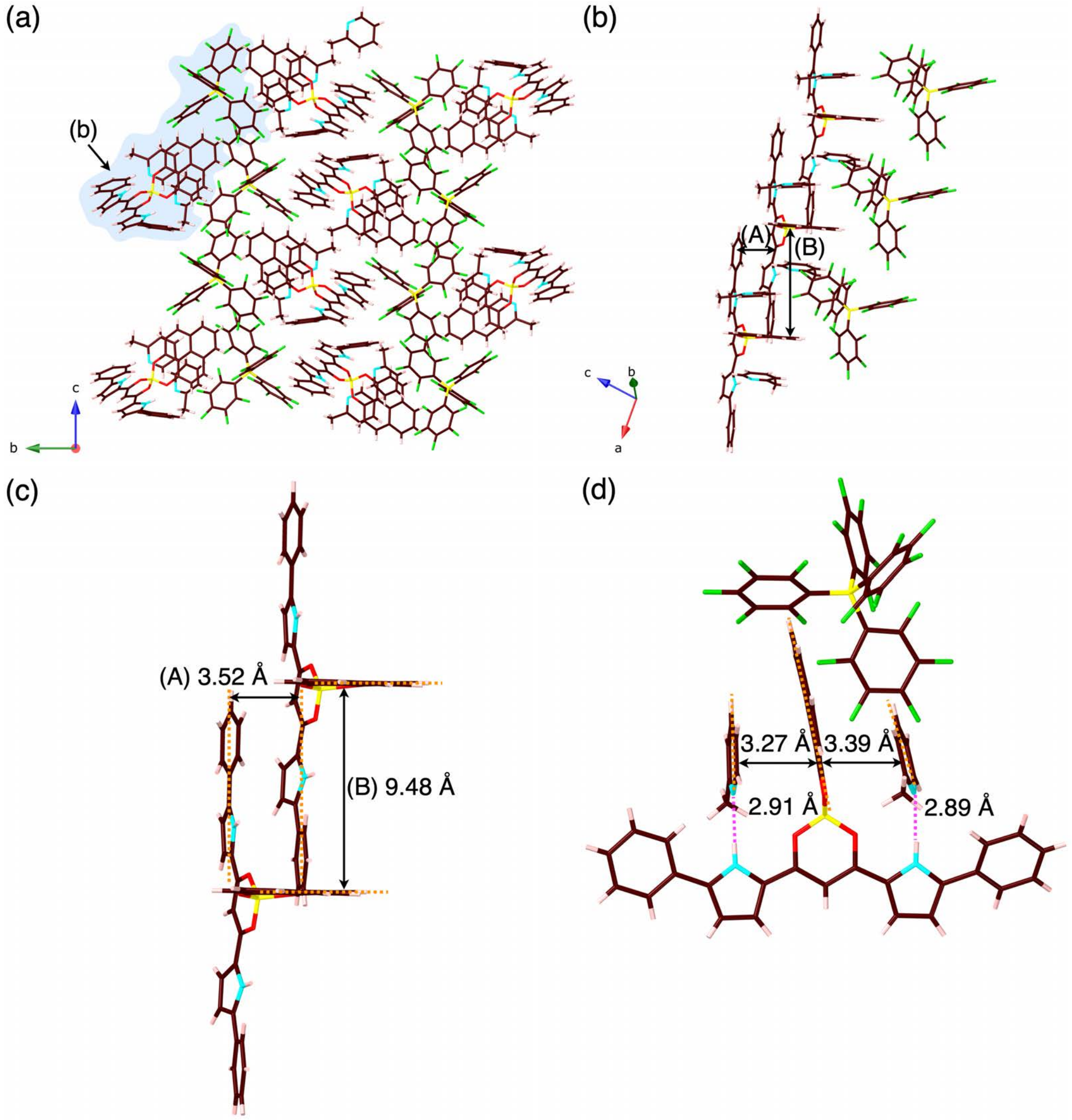

Fig. S25 Packing diagram (stacking assembly) of **3b**$^+$-B(C_6F_5)$_4^-$ as (a) top and (b) side views, (c) top views of the dimers, and (d) enlarged view of the ion pair. The distances between two **3b**$^+$, those between **3b**$^+$ and solvent, the C(–H)···F distances, and the N(–H)···N distances are 3.52/9.48, 3.27/3.39, 3.22/3.48, and 2.91/2.89 Å, respectively. In this diagram, two planes are defined as (i) the phenalenyl-based plane (core 16 atoms including the boron atom) and (ii) the dipyrrolyldiketone-based plane (core 28 atoms including the boron atom). Atom color code: brown, pink, yellow, blue, red, and green refer to carbon, hydrogen, boron, nitrogen, oxygen, and fluorine, respectively.

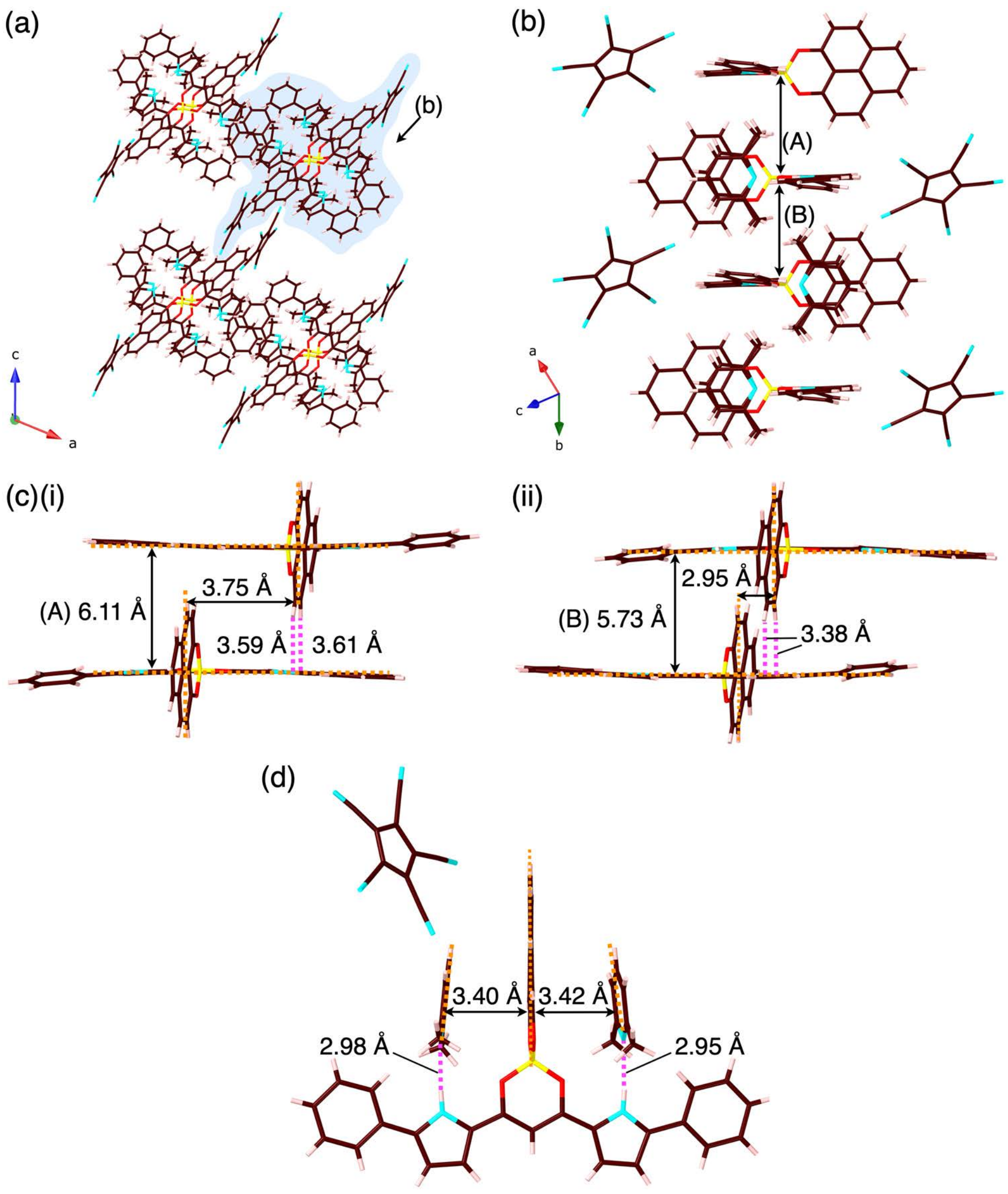

Fig. S26 Packing diagram (stacking assembly) of **3b**$^+$-PCCp$^-$ as (a) top and (b) side views, (c) top views of the dimers, and (d) enlarged view of the ion pair. The distances between two **3b**$^+$, those between **3b**$^+$ and solvent, and the N(–H)···N distances are 6.11/5.73, 3.40/3.42, and 2.95/2.98 Å, respectively. In this diagram, two planes are defined as (i) the phenalenyl-based plane (core 16 atoms including the boron atom) and (ii) the dipyrrolyldiketone-based plane (core 28 atoms including the boron atom). Solvent molecules (*n*-hexane) are omitted for clarity. Atom color code: brown, pink, yellow, blue, and red refer to carbon, hydrogen, boron, nitrogen, and oxygen, respectively.

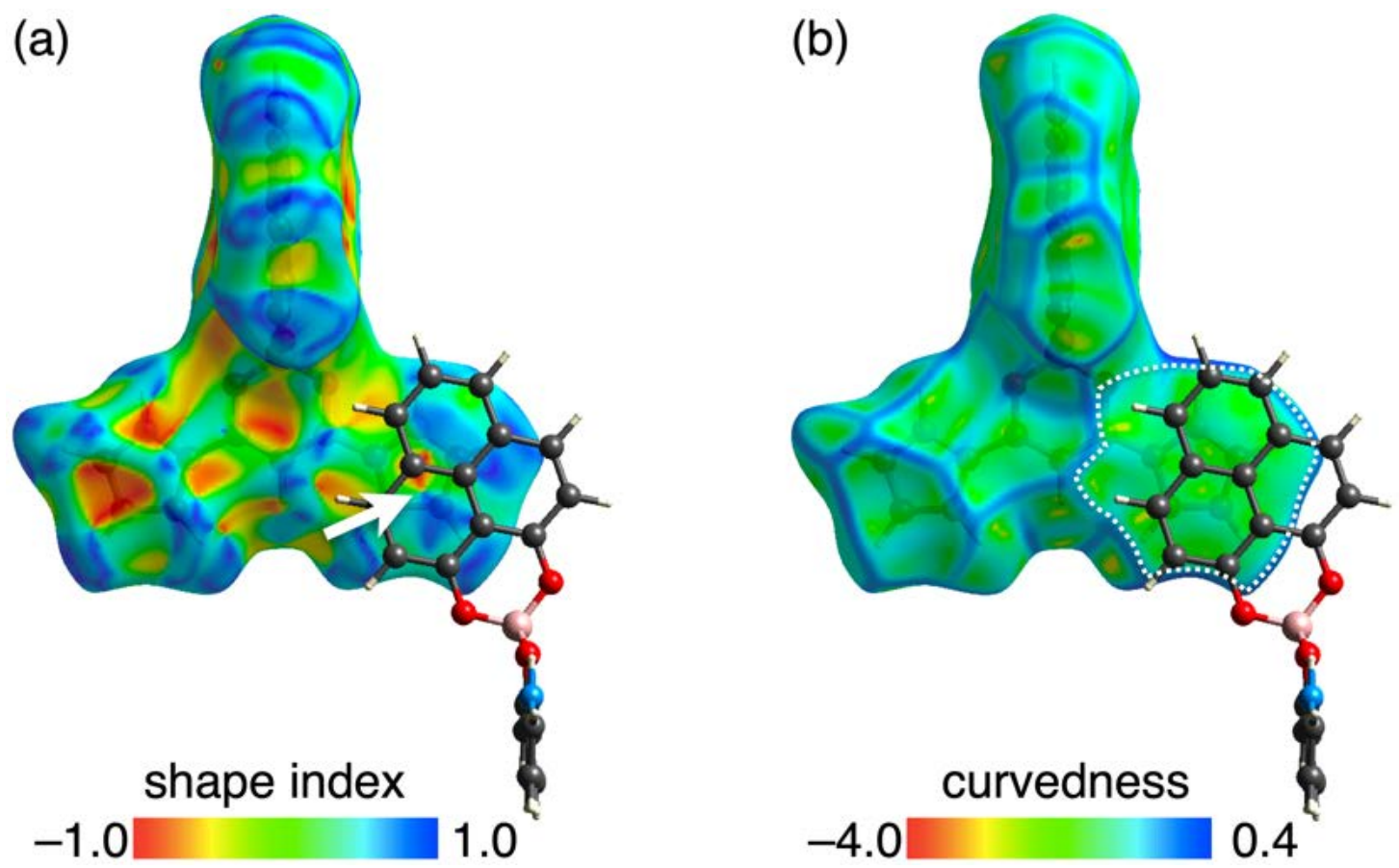

Fig. S27 Hirshfeld surfaces[S9] of **3a**$^{+}$ in **3a**$^{+}$-BF_4^{-} from the crystal structure: (a) shape index and (b) curvedness. Shape index is a qualitative measure of shape and is sensitive to subtle changes in surface shape, particularly in a flat region by differing by sign represent complementary bumps (blue) and hollows (red), whereas curvedness is a function of the root-mean-square curvature of the surface, and maps of curvedness typically show large regions of green (relatively flat) separated by dark blue edges (large positive curvature). Atom color code: gray, white, pink, blue, and red refer to carbon, hydrogen, boron, nitrogen, and oxygen, respectively.

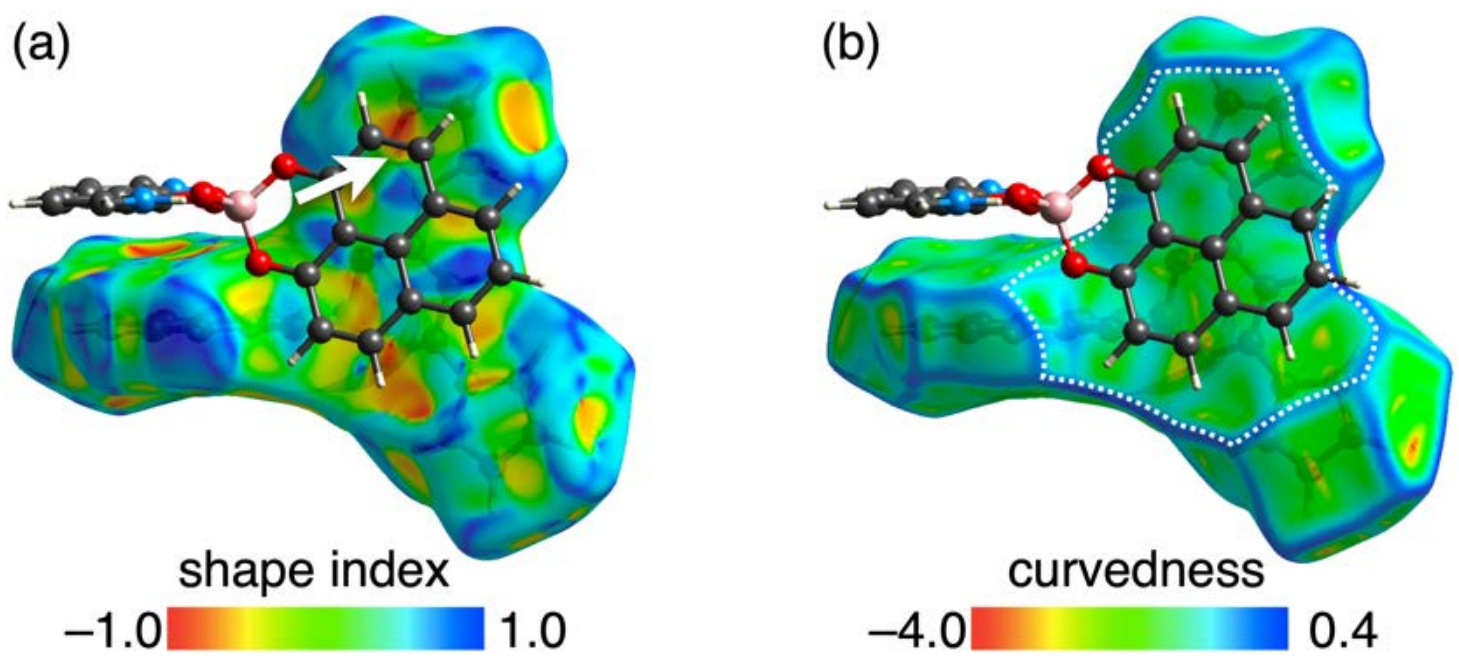

Fig. S28 Hirshfeld surfaces[S9] of **3a**$^{+}$ in **3a**$^{+}$-PF_6^{-} from the crystal structure: (a) shape index and (b) curvedness. Shape index is a qualitative measure of shape and is sensitive to subtle changes in surface shape, particularly in a flat region by differing by sign represent complementary bumps (blue) and hollows (red), whereas curvedness is a function of the root-mean-square curvature of the surface, and maps of curvedness typically show large regions of green (relatively flat) separated by dark blue edges (large positive curvature). Atom color code: gray, white, pink, blue, and red refer to carbon, hydrogen, boron, nitrogen, and oxygen, respectively.

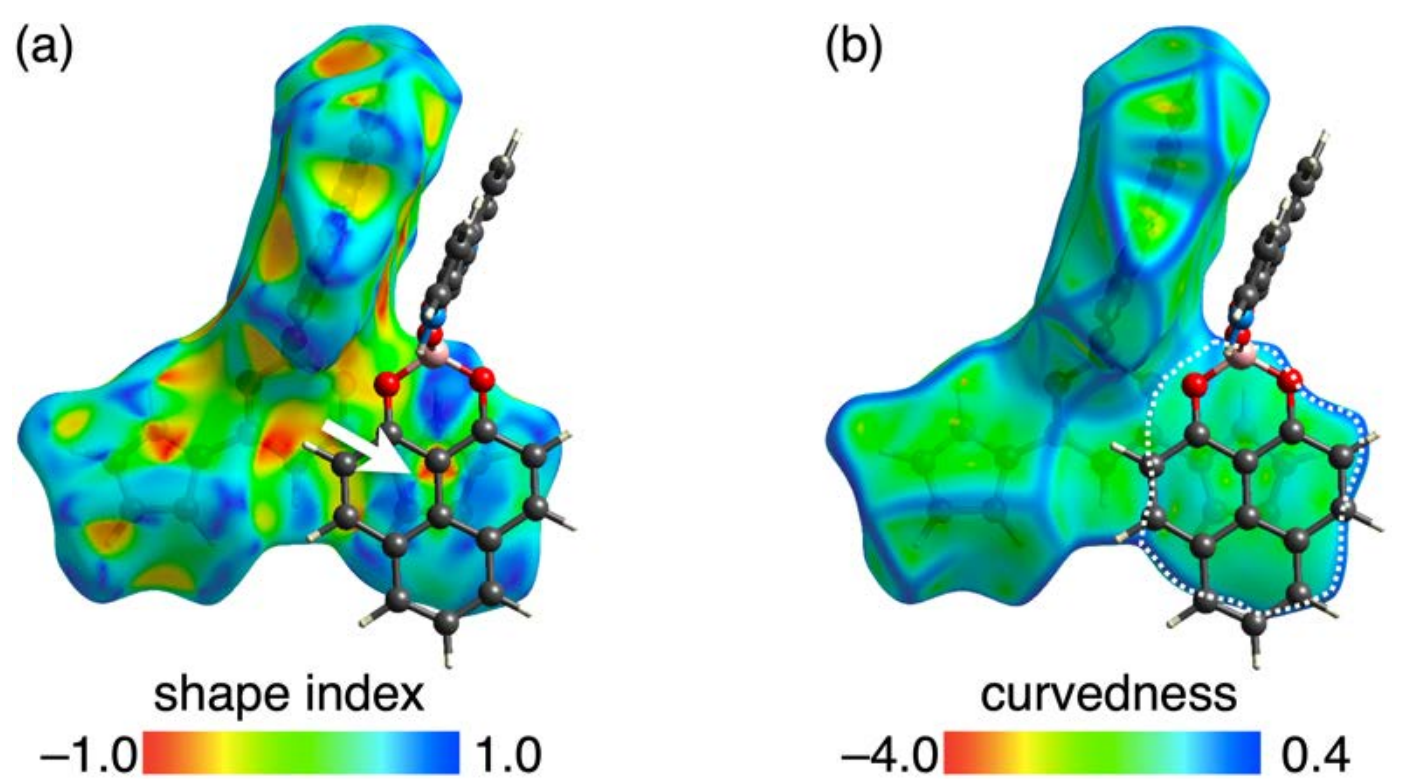

Fig. S29 Hirshfeld surfaces[S9] of **3a**$^{+}$ in **3a**$^{+}$-$B(C_6F_5)_4^{-}$ from the crystal structure: (a) shape index and (b) curvedness. Shape index is a qualitative measure of shape and is sensitive to subtle changes in surface shape, particularly in a flat region by differing by sign represent complementary bumps (blue) and hollows (red), whereas curvedness is a function of the root-mean-square curvature of the surface, and maps of curvedness typically show large regions of green (relatively flat) separated by dark blue edges (large positive curvature). Atom color code: gray, white, pink, blue, and red refer to carbon, hydrogen, boron, nitrogen, and oxygen, respectively.

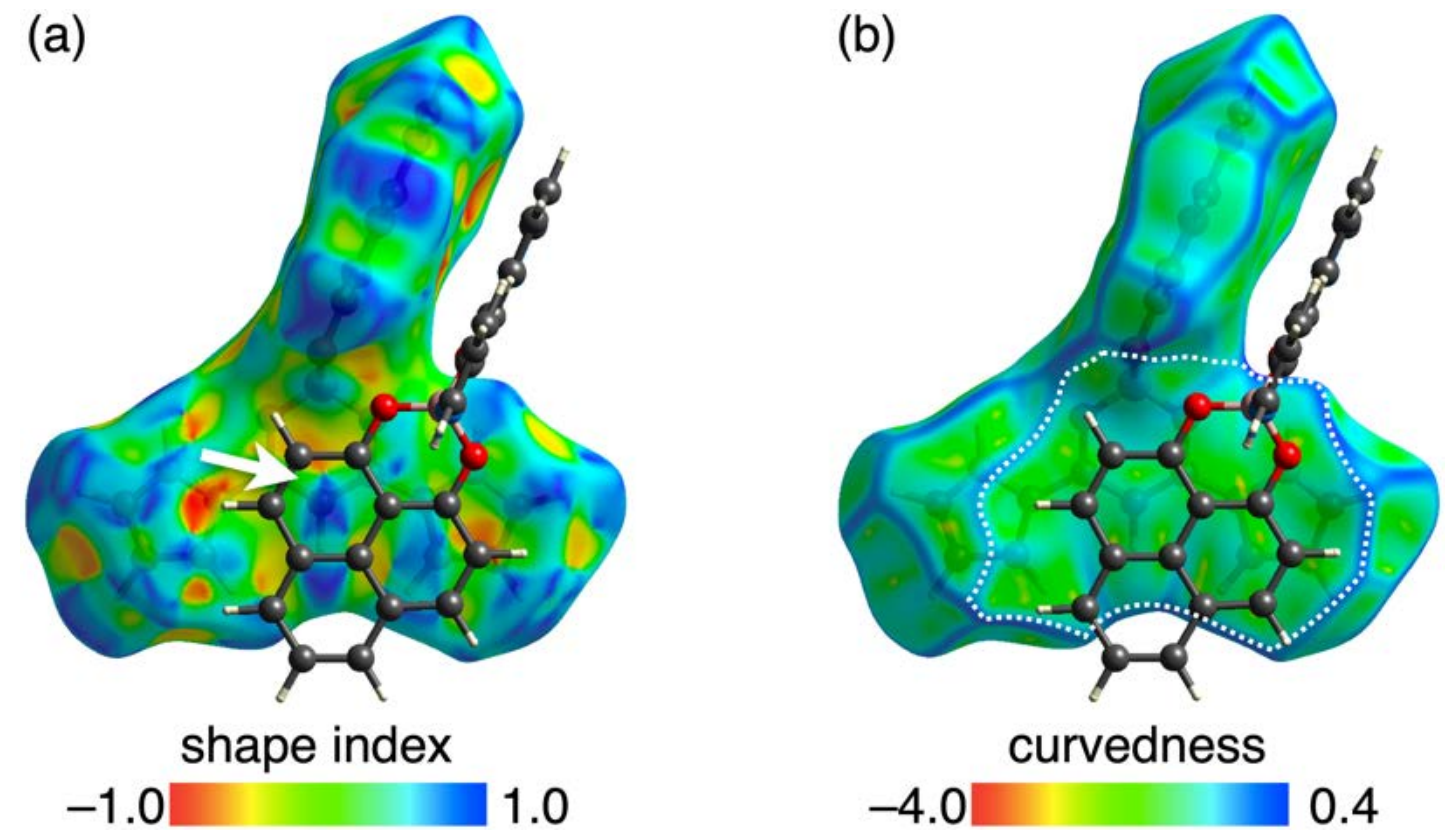

Fig. S30 Hirshfeld surfaces[S9] of **3a**$^+$ in **3a**$^+$-PCCp$^-$ from the crystal structure: (a) shape index and (b) curvedness. Shape index is a qualitative measure of shape and is sensitive to subtle changes in surface shape, particularly in a flat region by differing by sign represent complementary bumps (blue) and hollows (red), whereas curvedness is a function of the root-mean-square curvature of the surface, and maps of curvedness typically show large regions of green (relatively flat) separated by dark blue edges (large positive curvature). Atom color code: gray, white, pink, blue, and red refer to carbon, hydrogen, boron, nitrogen, and oxygen, respectively.

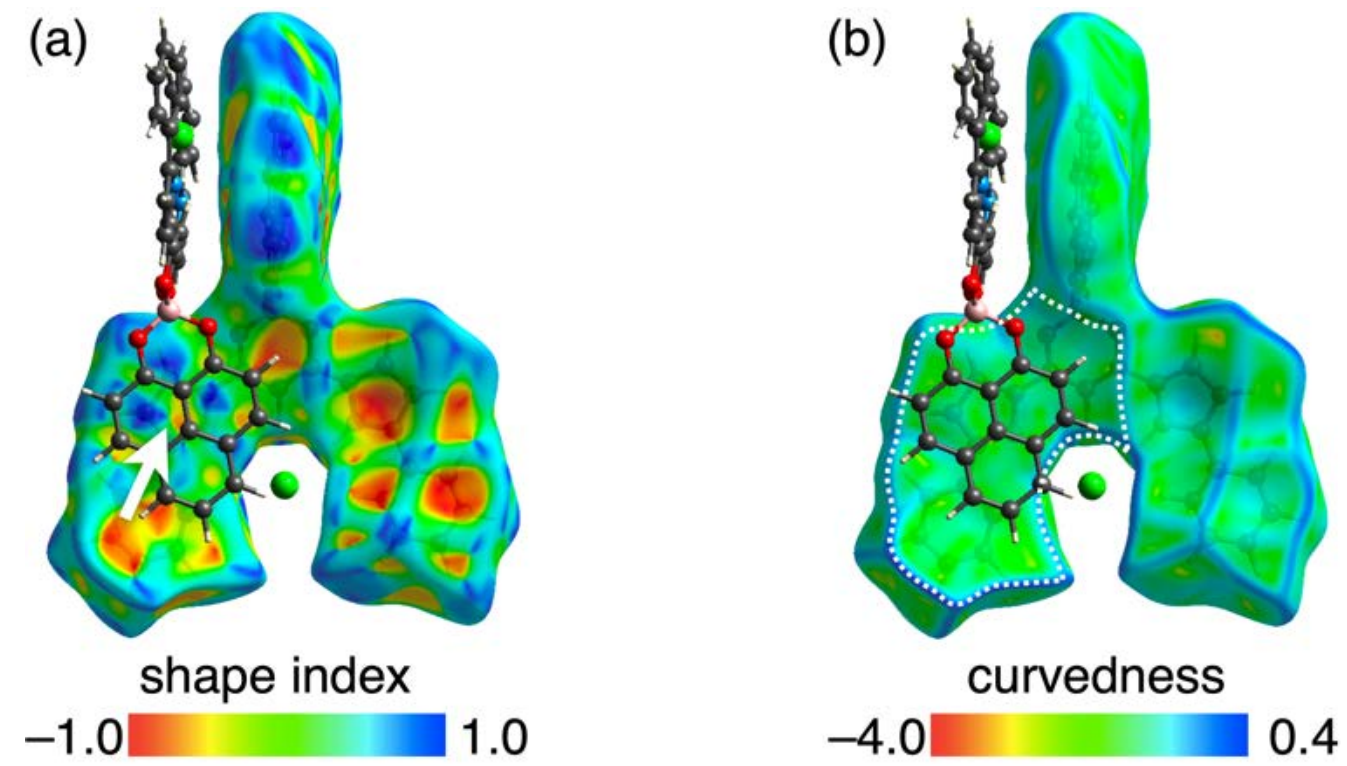

Fig. S31 Hirshfeld surfaces[S9] of **3b**$^+$-Cl$^-$ from the crystal structure: (a) shape index and (b) curvedness. Shape index is a qualitative measure of shape and is sensitive to subtle changes in surface shape, particularly in a flat region by differing by sign represent complementary bumps (blue) and hollows (red), whereas curvedness is a function of the root-mean-square curvature of the surface, and maps of curvedness typically show large regions of green (relatively flat) separated by dark blue edges (large positive curvature). Atom color code: gray, white, pink, blue, red, and green refer to carbon, hydrogen, boron, nitrogen, oxygen, and chlorine, respectively.

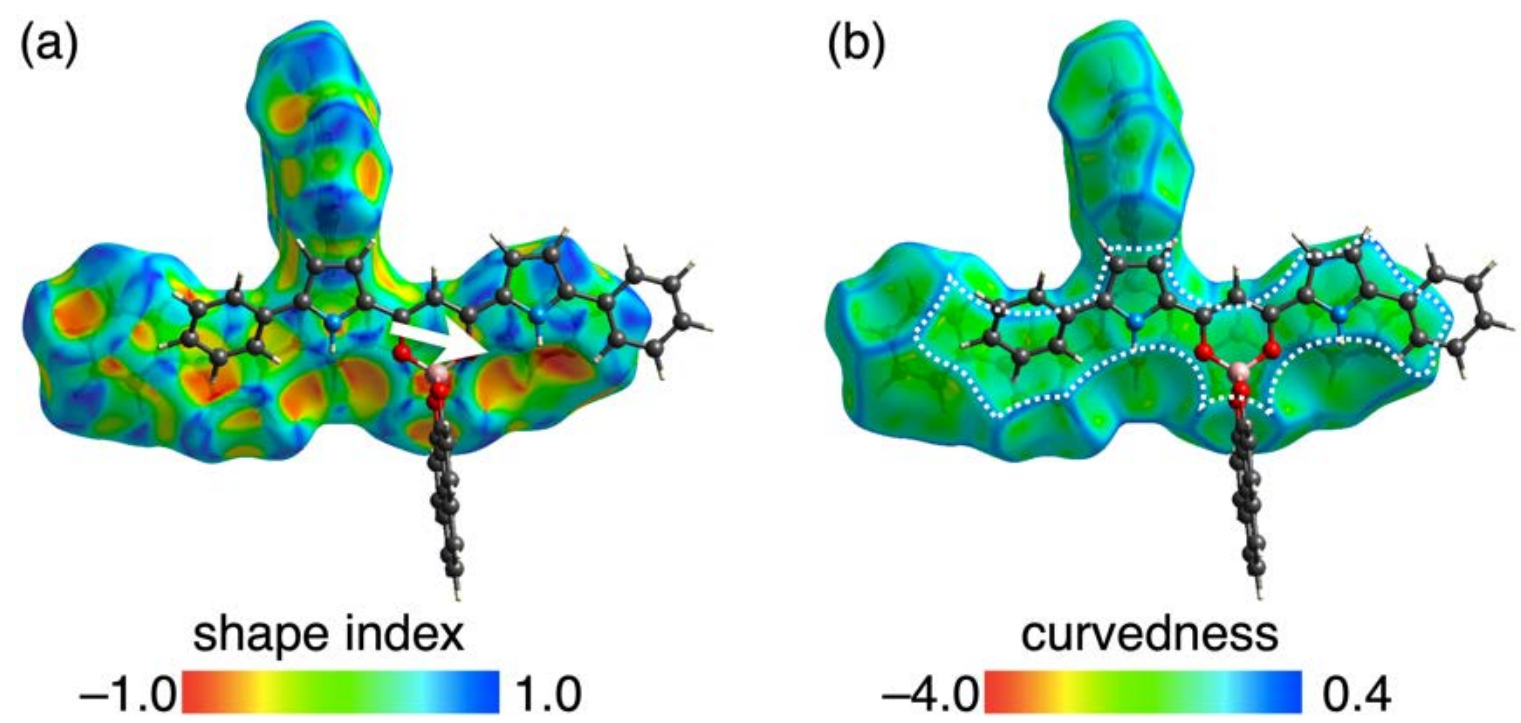

Fig. S32 Hirshfeld surfaces[S9] of **3b**$^+$ in **3b**$^+$-PF$_6^-$ from the crystal structure: (a) shape index and (b) curvedness. Shape index is a qualitative measure of shape and is sensitive to subtle changes in surface shape, particularly in a flat region by differing by sign represent complementary bumps (blue) and hollows (red), whereas curvedness is a function of the root-mean-square curvature of the surface, and maps of curvedness typically show large regions of green (relatively flat) separated by dark blue edges (large positive curvature). Atom color code: gray, white, pink, blue, and red refer to carbon, hydrogen, boron, nitrogen, and oxygen, respectively.

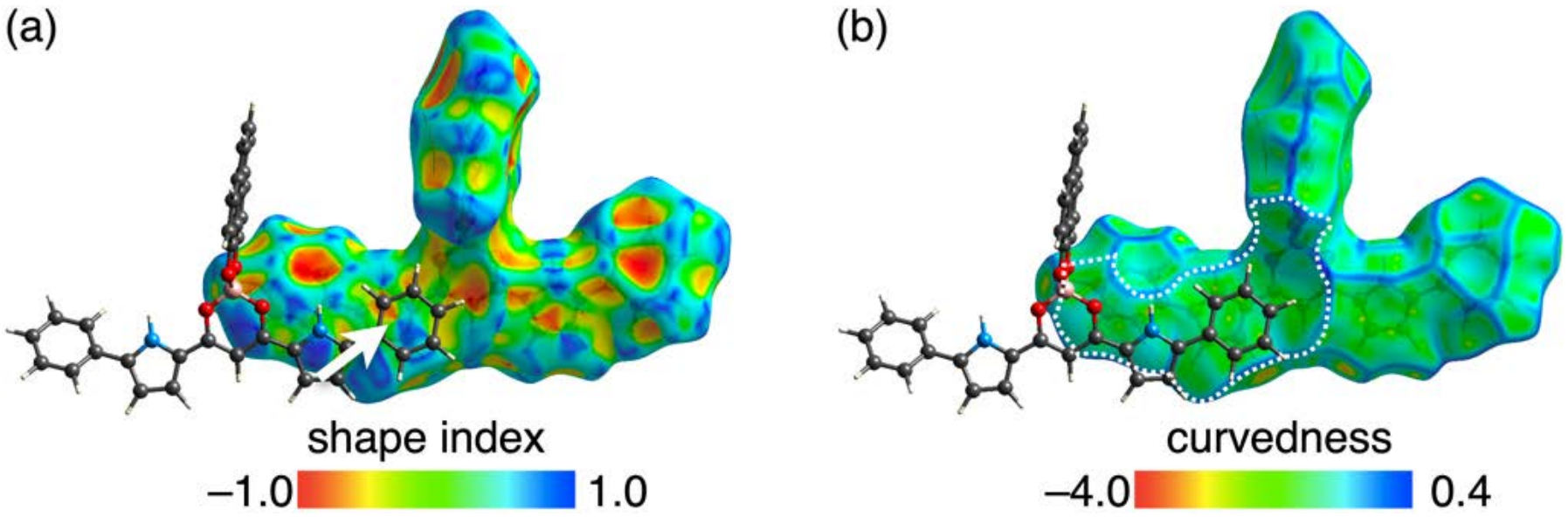

Fig. S33 Hirshfeld surfaces[S9] of **3b**$^+$ in **3b**$^+$-B(C_6F_5)$_4^-$ from the crystal structure: (a) shape index and (b) curvedness. Shape index is a qualitative measure of shape and is sensitive to subtle changes in surface shape, particularly in a flat region by differing by sign represent complementary bumps (blue) and hollows (red), whereas curvedness is a function of the root-mean-square curvature of the surface, and maps of curvedness typically show large regions of green (relatively flat) separated by dark blue edges (large positive curvature). Atom color code: gray, white, pink, blue, and red refer to carbon, hydrogen, boron, nitrogen, and oxygen, respectively.

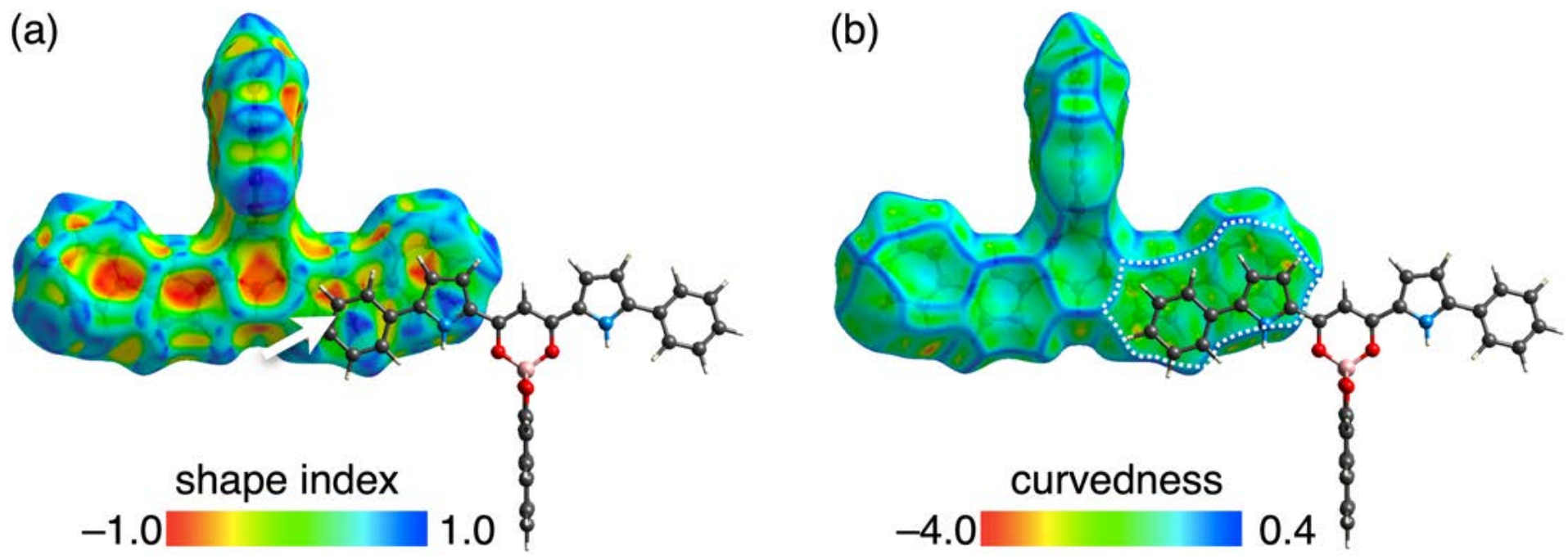

Fig. S34 Hirshfeld surfaces[S9] of **3b**$^+$ in **3b**$^+$-PCCp$^-$ from the crystal structure: (a) shape index and (b) curvedness. Shape index is a qualitative measure of shape and is sensitive to subtle changes in surface shape, particularly in a flat region by differing by sign represent complementary bumps (blue) and hollows (red), whereas curvedness is a function of the root-mean-square curvature of the surface, and maps of curvedness typically show large regions of green (relatively flat) separated by dark blue edges (large positive curvature). Atom color code: gray, white, pink, blue, and red refer to carbon, hydrogen, boron, nitrogen, and oxygen, respectively.

[S5] K. Sugimoto, H. Ohsumi, S. Aoyagi, E. Nishibori, C. Moriyoshi, Y. Kuroiwa, H. Sawa and M. Takata, *AIP Conf. Proc.*, 2010, **1234**, 887–890.

[S6] (a) N. Yasuda, H. Murayama, Y. Fukuyama, J. E. Kim, S. Kimura, K. Toriumi, Y. Tanaka, Y. Moritomo, Y. Kuroiwa, K. Kato, H. Tanaka and M. Takata, *J. Synchrotron Rad.*, 2009, **16**, 352–357; (b) N. Yasuda, Y. Fukuyama, K. Toriumi, S. Kimura and M. Takata, *AIP Conf. Proc.*, 2010, **1234**, 147–150.

[S7] G. M. Sheldrick, *Acta Crystallogr. Sect. A*, 2008, **64**, 112–122.

[S8] (a) K. Wakita, *Yadokari-XG*, *Software for Crystal Structure Analyses*, 2001; (b) C. Kabuto, S. Akine, T. Nemoto and E. Kwon, *J. Cryst. Soc. Jpn.*, 2009, **51**, 218–224.

[S9] P. R. Spackman, M. J. Turner, J. J. McKinnon, S. K. Wolff, D. J. Grimwood, D. Jayatilaka and M. A. Spackman, *J. Appl. Cryst.*, 2021, **54**, 1006–1011.

3. Theoretical studies

DFT calculations. DFT calculations for the geometrical optimizations were carried out by using the *Gaussian 16* program.[S10]

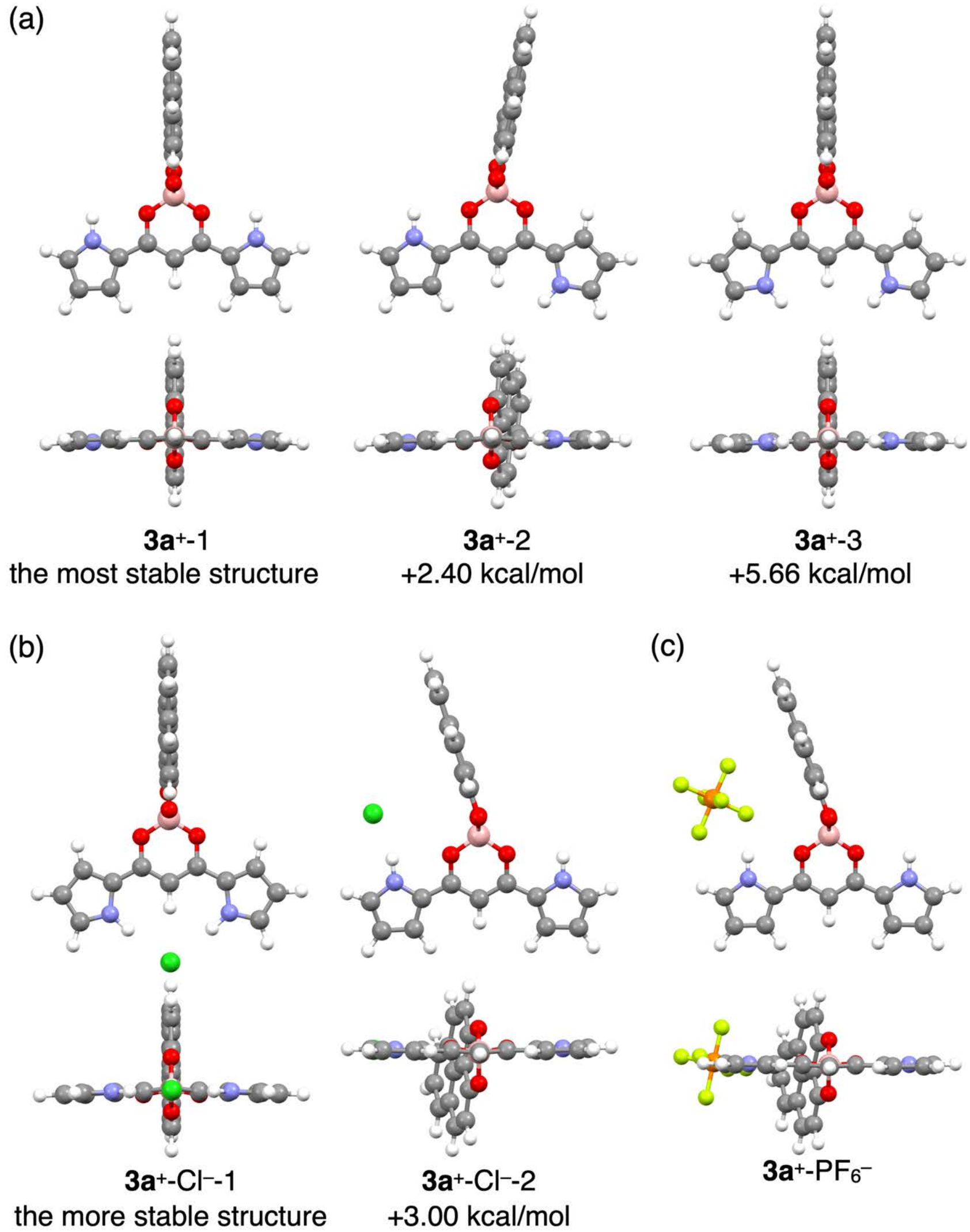

Fig. S35 Optimized structures (top and side views) of (a) **3a**$^+$ (three conformations) at CAM-B3LYP/6-31+G(d,p) and (b) **3a**$^+$-Cl^- (two conformations) and (c) **3a**$^+$-PF_6^- at CPCM-CAM-B3LYP/6-31++G(d,p) (CH_2Cl_2).

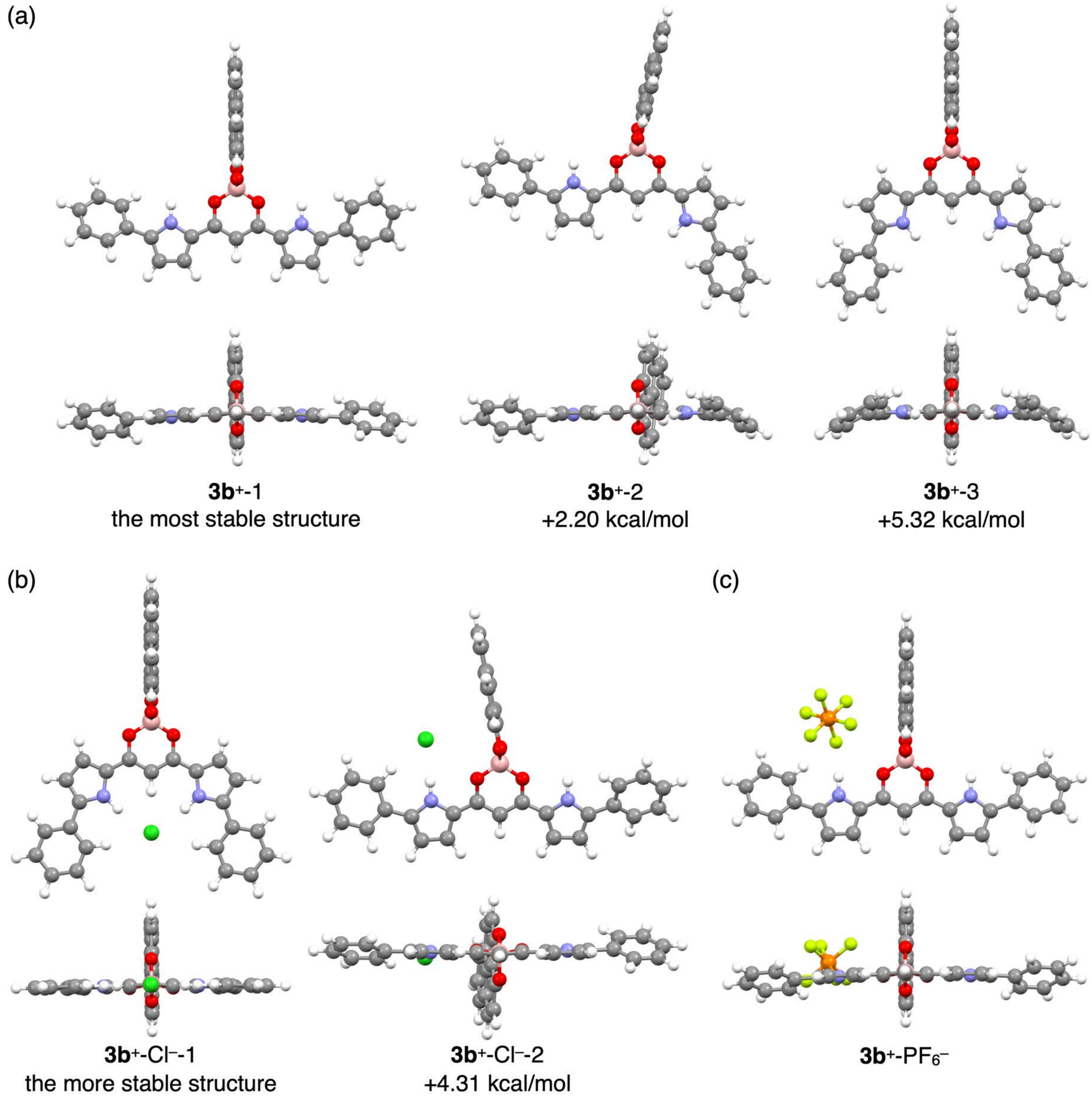

Fig. S36 Optimized structures (top and side views) of (a) **3b**$^+$ (three conformations) at CAM-B3LYP/6-31+G(d,p) and (b) **3b**$^+$-Cl^- (two conformations) and (c) **3b**$^+$-PF_6^- at CPCM-CAM-B3LYP/6-31++G(d,p) (CH_2Cl_2).

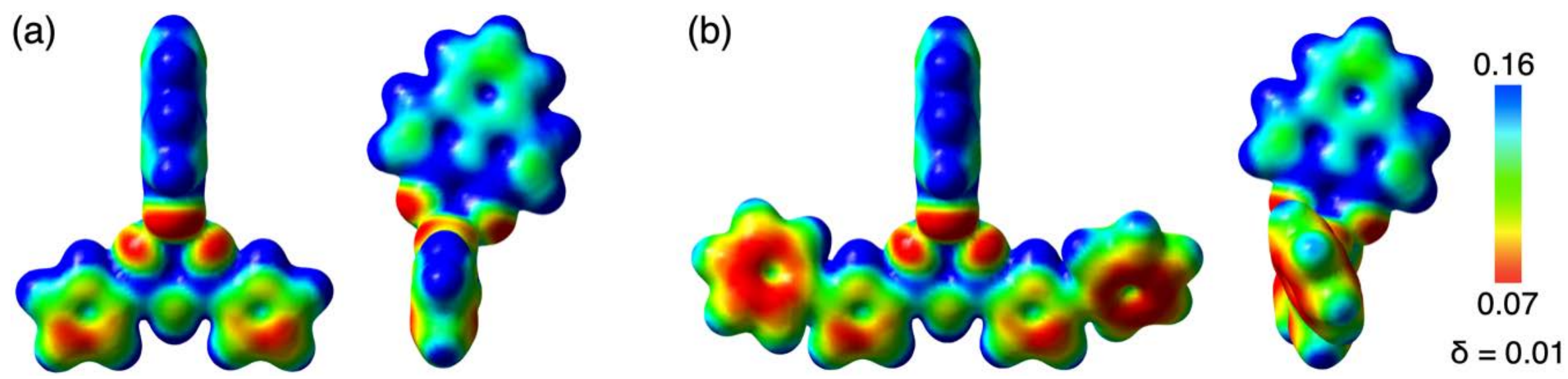

Fig. S37 Electrostatic potentials (ESP) mapped onto the electron density isosurfaces ($\delta = 0.01$) of (a) **3a**$^+$ and (b) **3b**$^+$ at CPCM-CAM-B3LYP/6-31++G(d,p) (CH_2Cl_2).

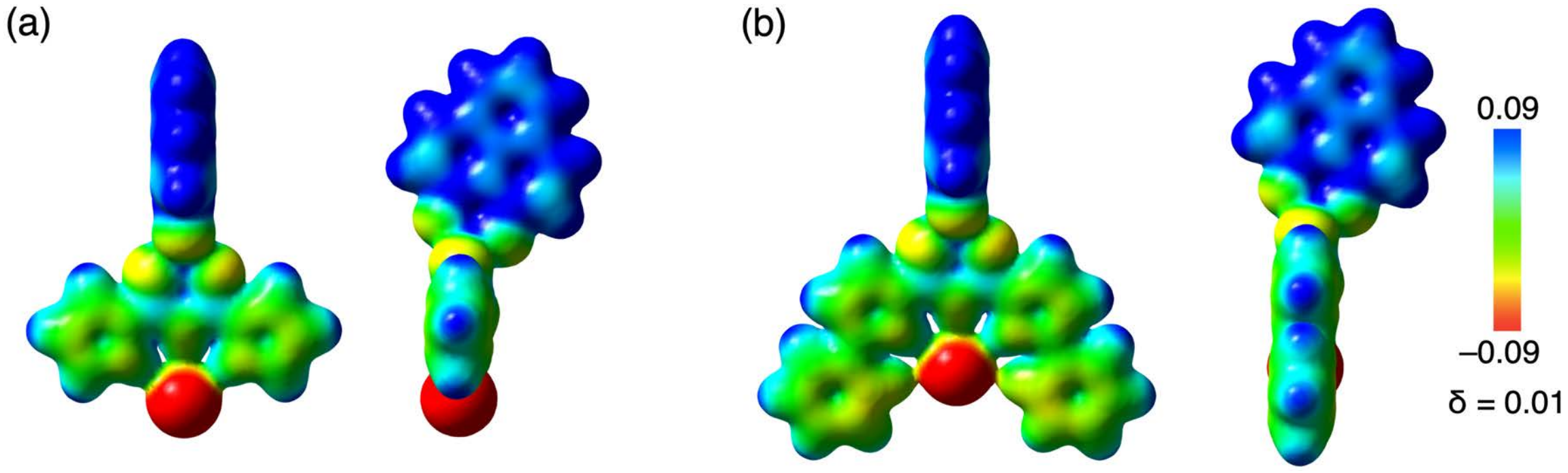

Fig. S38 Electrostatic potentials (ESP) mapped onto the electron density isosurfaces (δ = 0.01) of (a) **3a**$^{+}$-Cl^{-} and (b) **3b**$^{+}$-Cl^{-} at CPCM-CAM-B3LYP/6-31++G(d,p) (CH_2Cl_2).

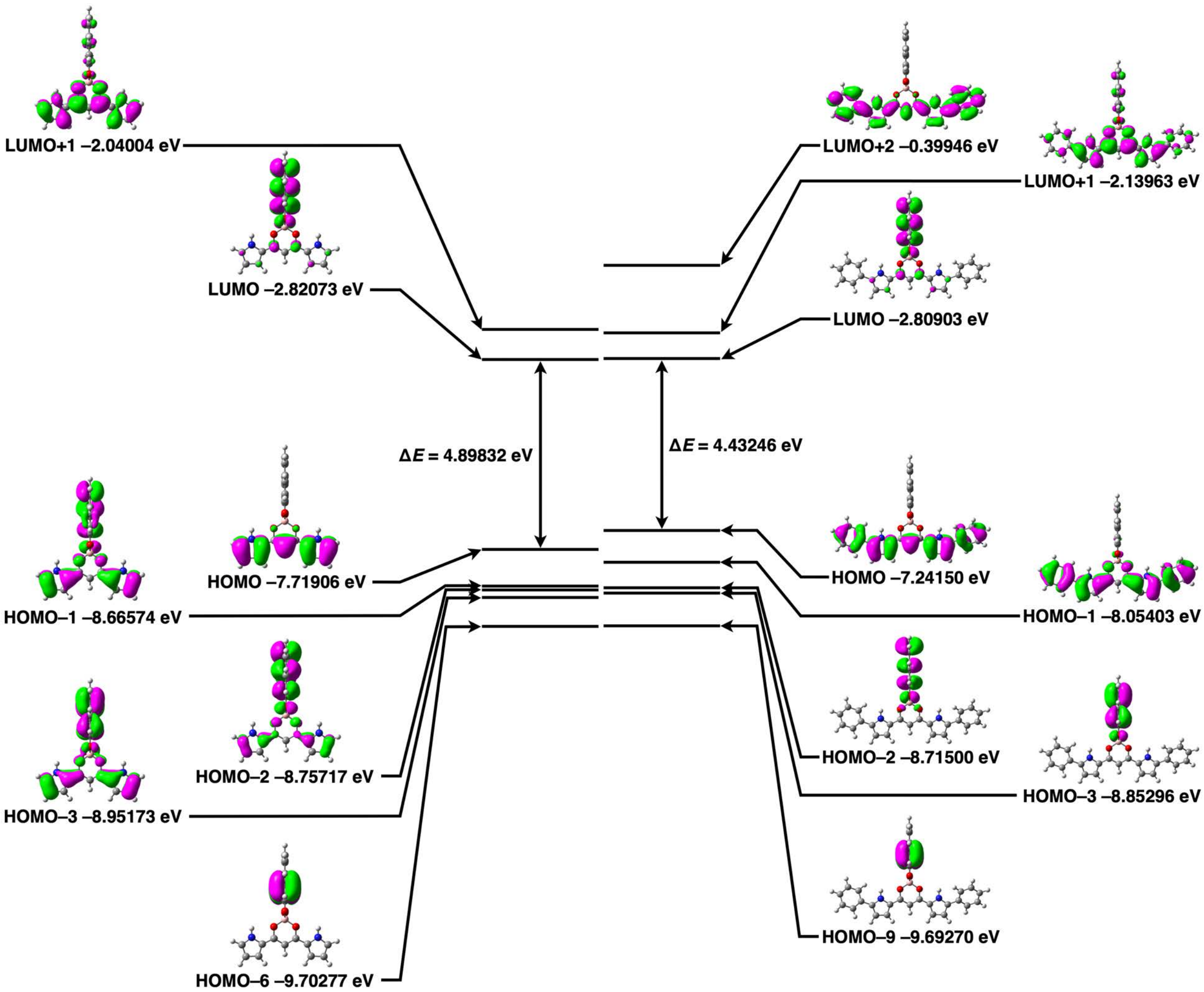

Fig. S39 Molecular orbitals (HOMO/LUMO) of **3a**$^{+}$ (left) and **3b**$^{+}$ (right) estimated at CPCM-CAM-B3LYP/6-31++G(d,p) (CH_2Cl_2).

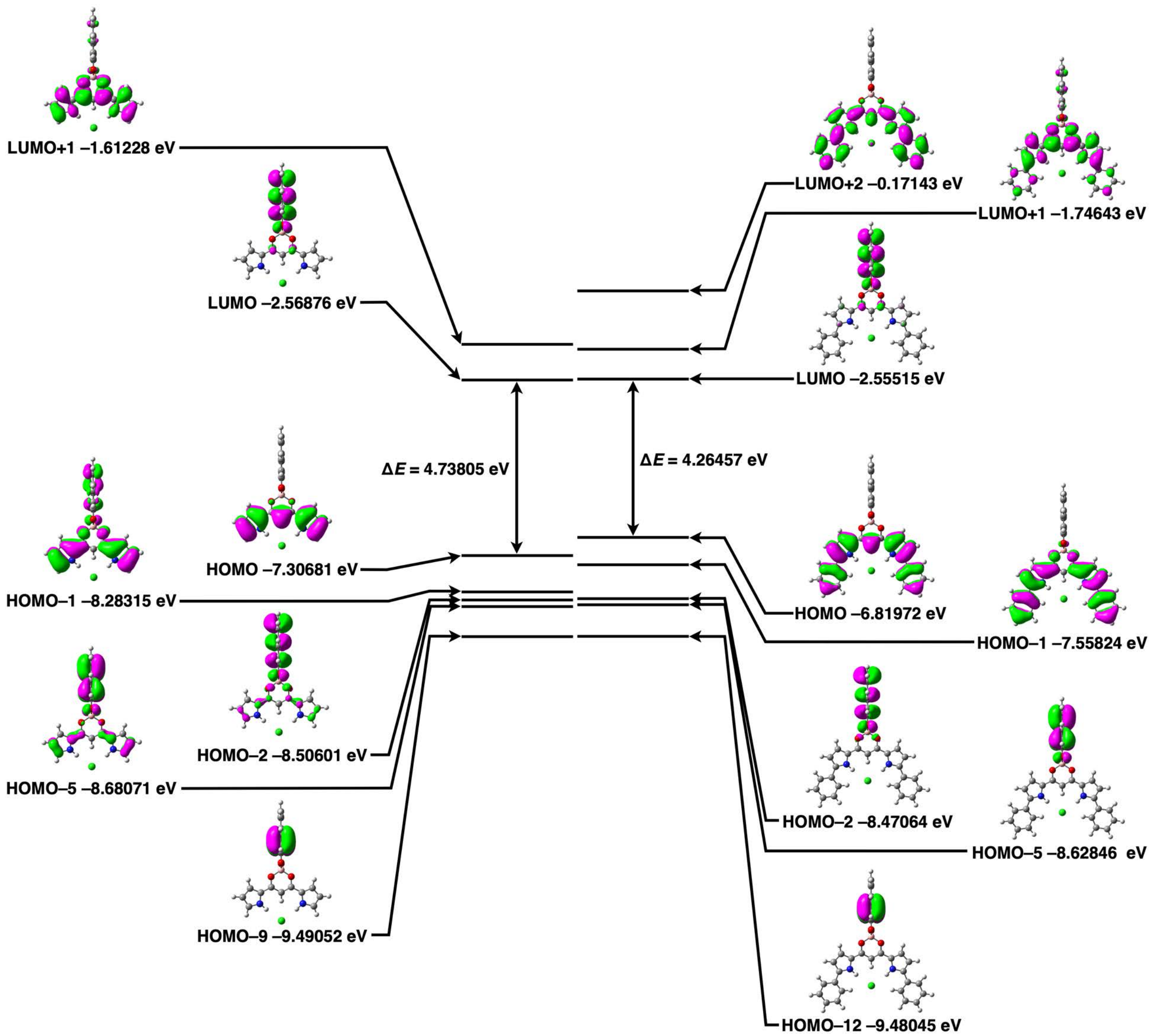

Fig. S40 Molecular orbitals (HOMO/LUMO) of **3a**$^{+}$-Cl^{-} (left) and **3b**$^{+}$-Cl^{-} (right) estimated at CPCM-CAM-B3LYP/6-31++G(d,p) (CH_2Cl_2).

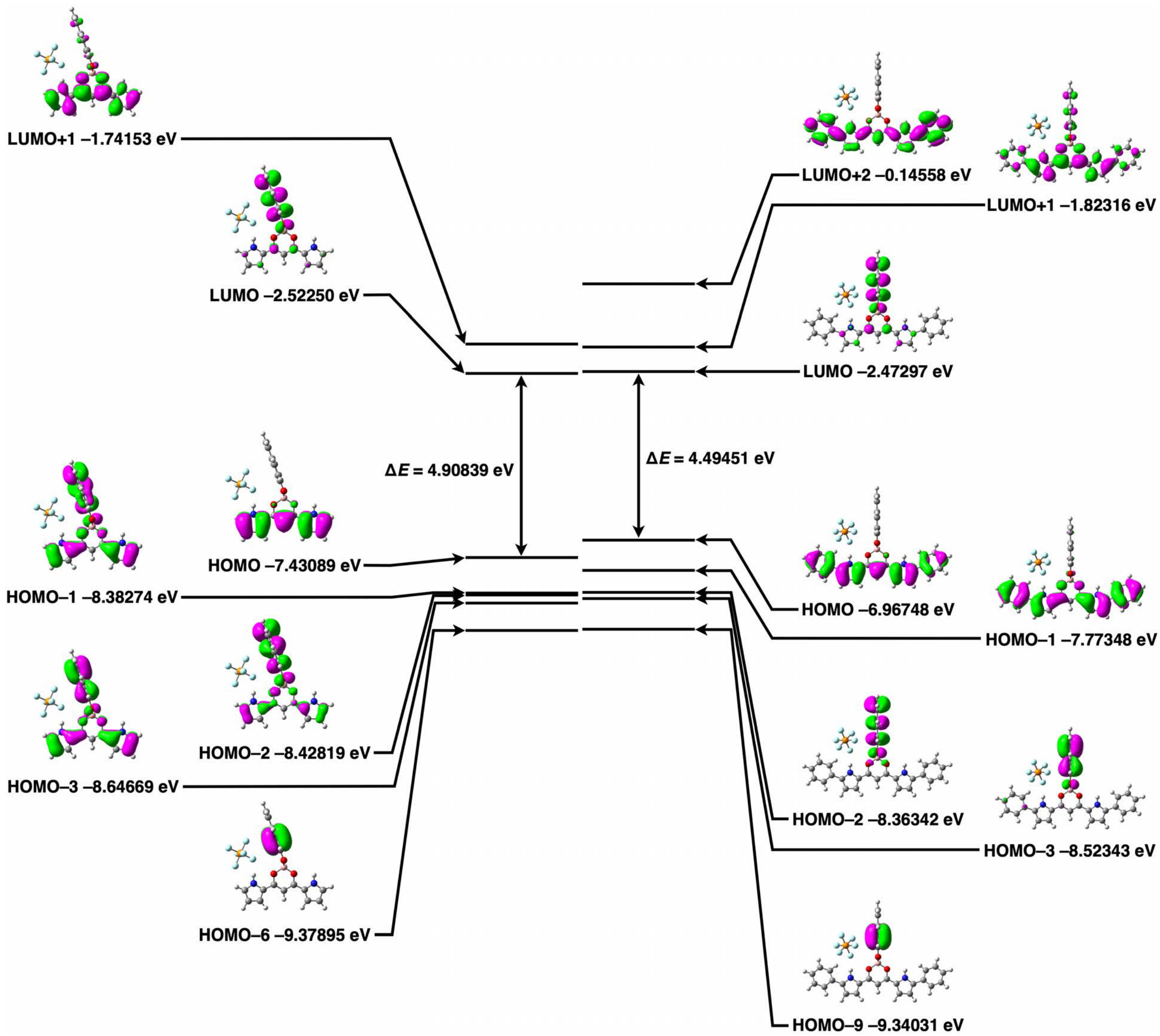

Fig. S41 Molecular orbitals (HOMO/LUMO) of **3a**$^+$-PF_6^- (left) and **3b**$^+$-PF_6^- (right) estimated at CPCM-CAM-B3LYP/6-31++G(d,p) (CH_2Cl_2).

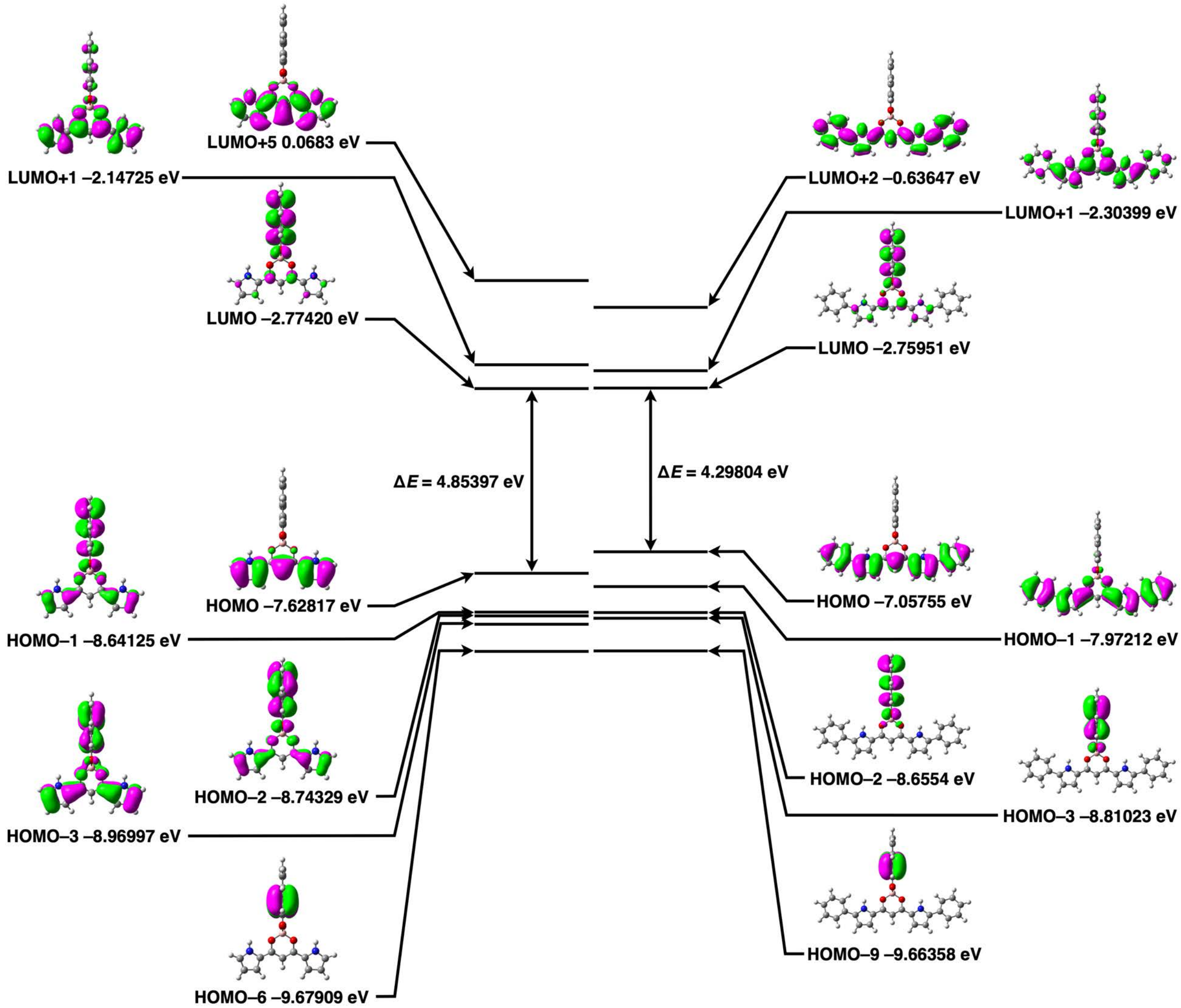

Fig. S42 Molecular orbitals (HOMO/LUMO) of S_1-state **3a**$^+$ (left) and **3b**$^+$ (right) estimated at CPCM-CAM-B3LYP/6-31++G(d,p) (CH_2Cl_2).

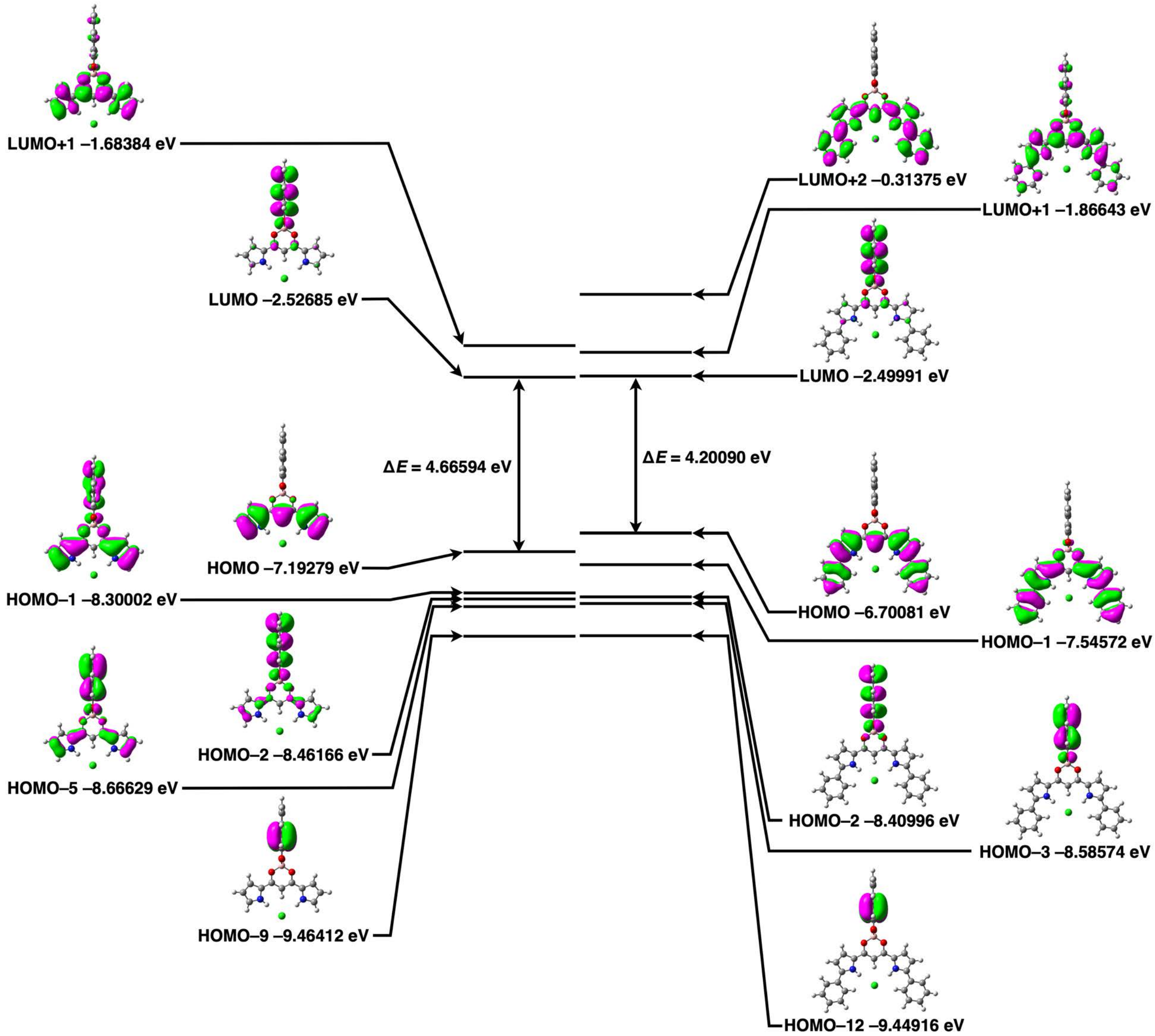

Fig. S43 Molecular orbitals (HOMO/LUMO) of S_1-state **3a**$^+$-Cl^- (left) and **3b**$^+$-Cl^- (right) estimated at CPCM-CAM-B3LYP/6-31++G(d,p) (CH_2Cl_2).

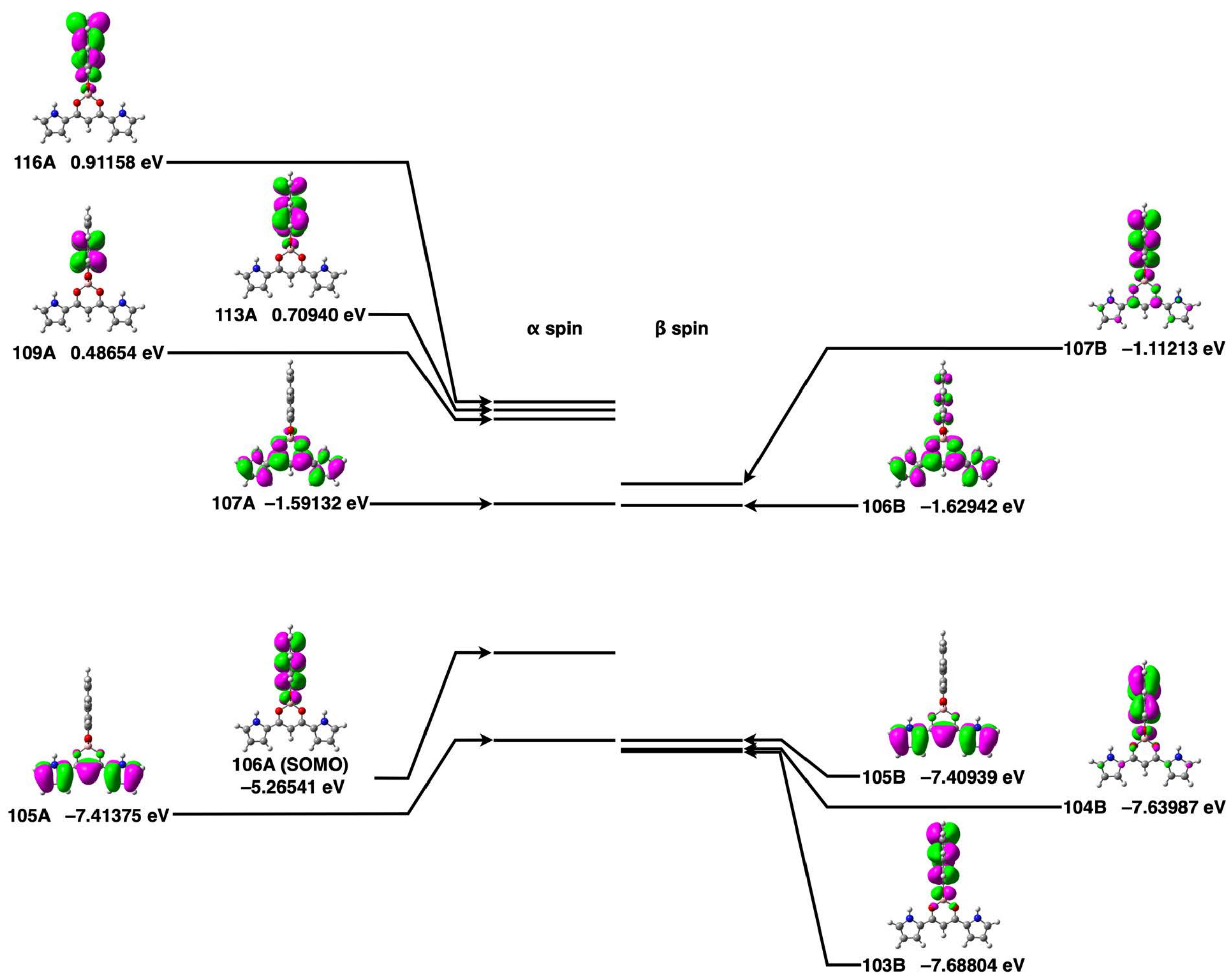

Fig. S44 Molecular orbitals (HOMO/LUMO) of reduced-state **3a**$^{+}$ with α-spin (left) and β-spin (right) estimated at CPCM-CAM-B3LYP/6-31++G(d,p) (CH_2Cl_2). In 106A, the SOMO is localized on the phenalenyl unit, suggesting electron-accepting behavior of the phenalenyl unit.

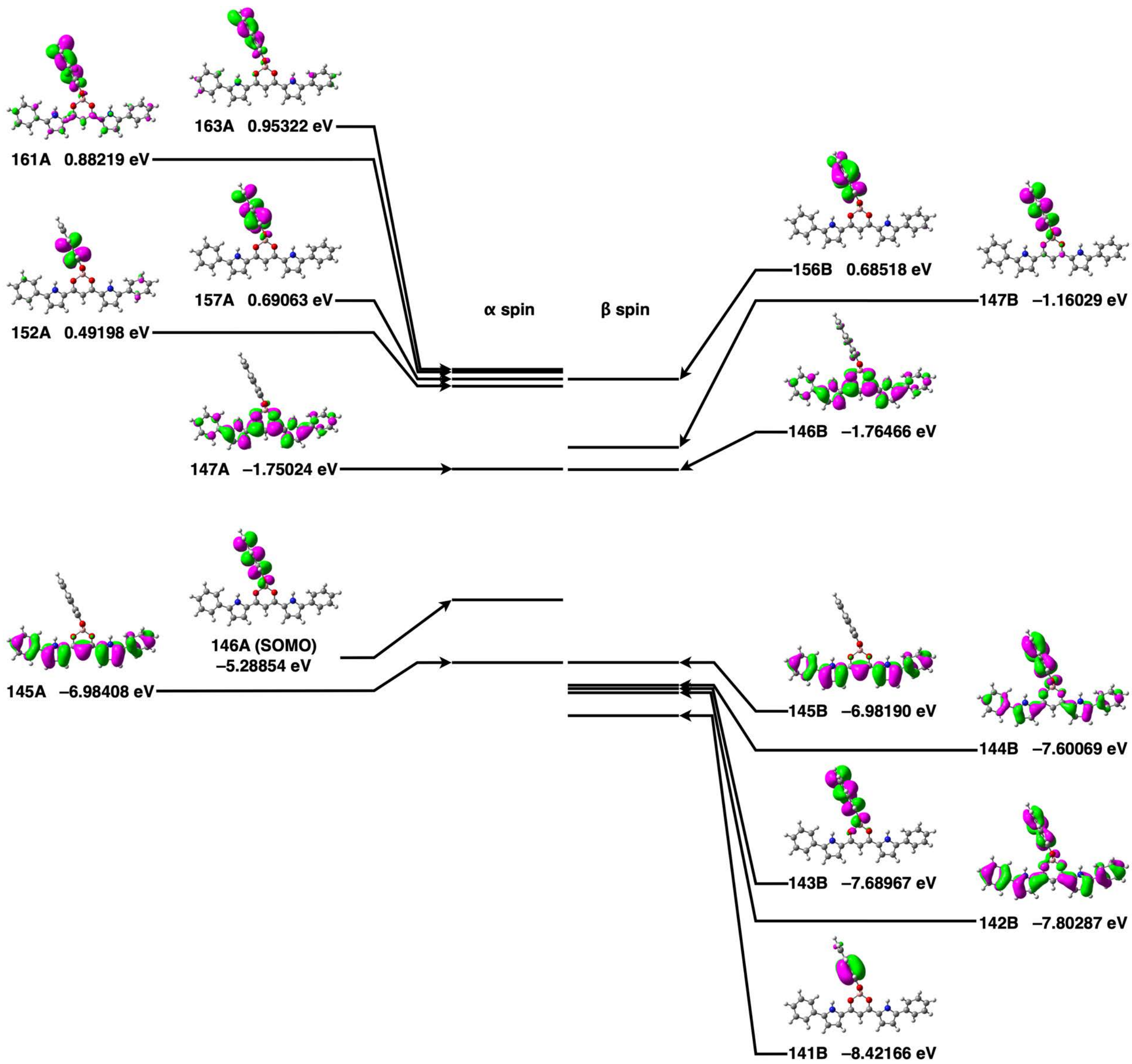

Fig. S45 Molecular orbitals (HOMO/LUMO) of reduced-state **3b**$^{+}$ with α-spin (left) and β-spin (right) estimated at CPCM-CAM-B3LYP/6-31++G(d,p) (CH_2Cl_2). In 146A, the SOMO is localized on the phenalenyl unit, suggesting electron-accepting behavior of the phenalenyl unit.

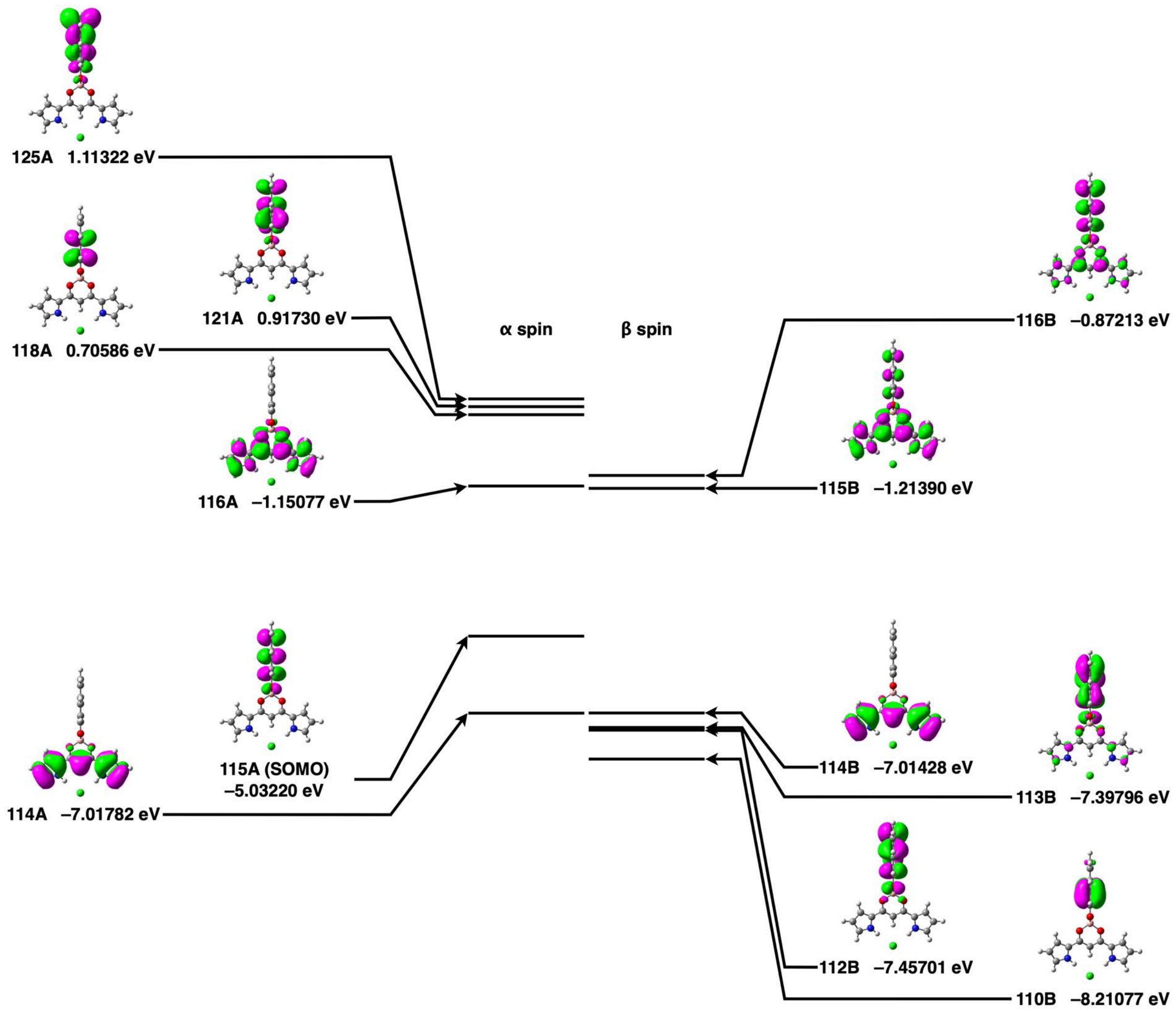

Fig. S46 Molecular orbitals (HOMO/LUMO) of reduced state **3a**$^{+}$-Cl^{-} with α-spin (left) and β-spin (right) estimated at CPCM-CAM-B3LYP/6-31++G(d,p) (CH_2Cl_2). In 115A, the SOMO is localized on the phenalenyl unit, suggesting electron-accepting behavior of the phenalenyl unit.

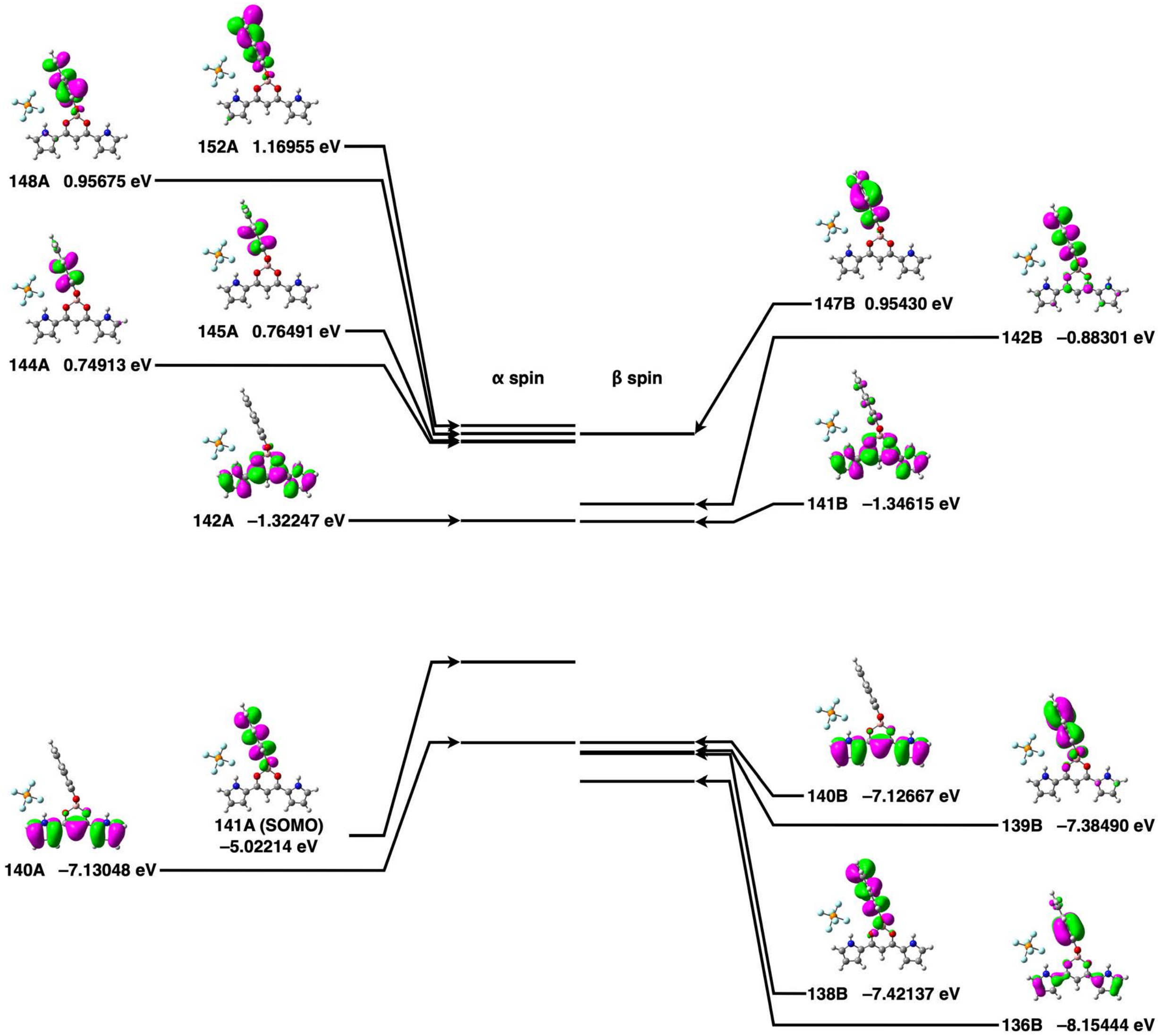

Fig. S47 Molecular orbitals (HOMO/LUMO) of reduced state **3a**$^{+}$-PF_6^{-} with α-spin (left) and β-spin (right) estimated at CPCM-CAM-B3LYP/6-31++G(d,p) (CH_2Cl_2). In 141A, the SOMO is localized on the phenalenyl unit, suggesting electron-accepting behavior of the phenalenyl unit.

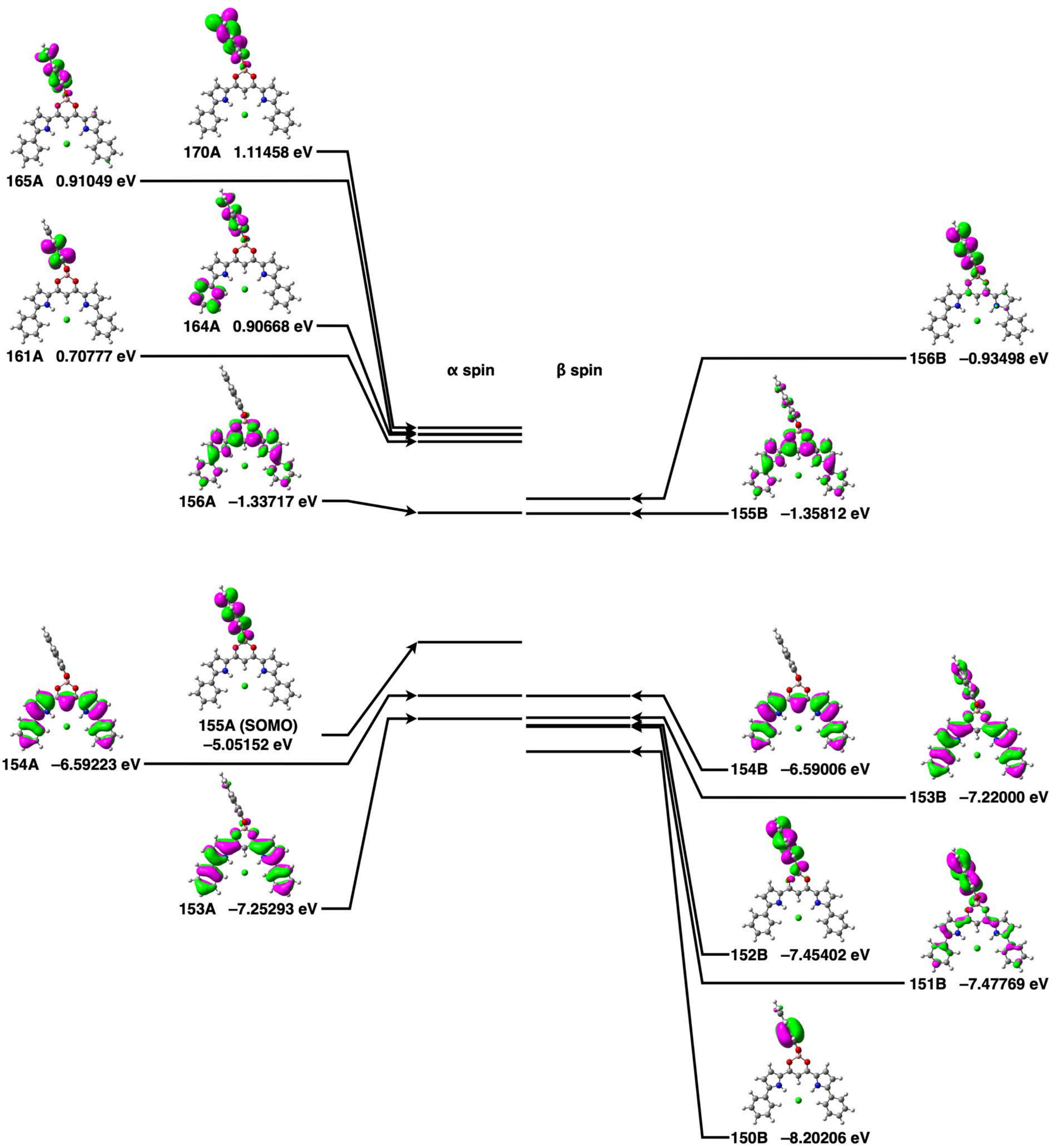

Fig. S48 Molecular orbitals (HOMO/LUMO) of reduced-state **3b**$^{+}$-Cl^{-} with α-spin (left) and β-spin (right) estimated at CPCM-CAM-B3LYP/6-31++G(d,p) (CH_2Cl_2). In 155A, the SOMO is localized on the phenalenyl unit, suggesting electron-accepting behavior of the phenalenyl unit.

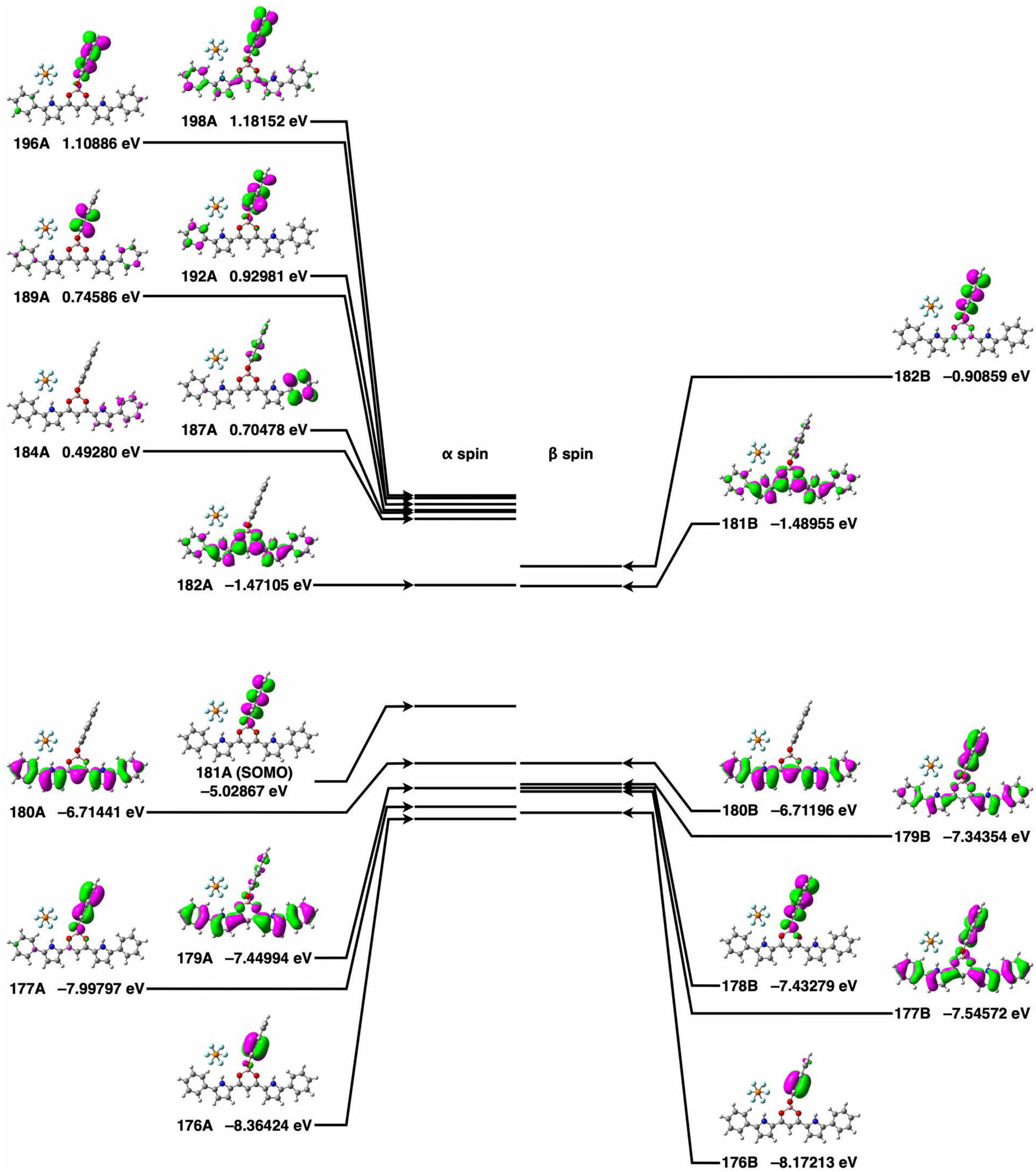

Fig. S49 Molecular orbitals (HOMO/LUMO) of reduced state **3b**$^{+}$-PF_6^{-} with α-spin (left) and β-spin (right) estimated at CPCM-CAM-B3LYP/6-31++G(d,p) (CH_2Cl_2). In 181A, the SOMO is localized on the phenalenyl unit, suggesting electron-accepting behavior of the phenalenyl unit.

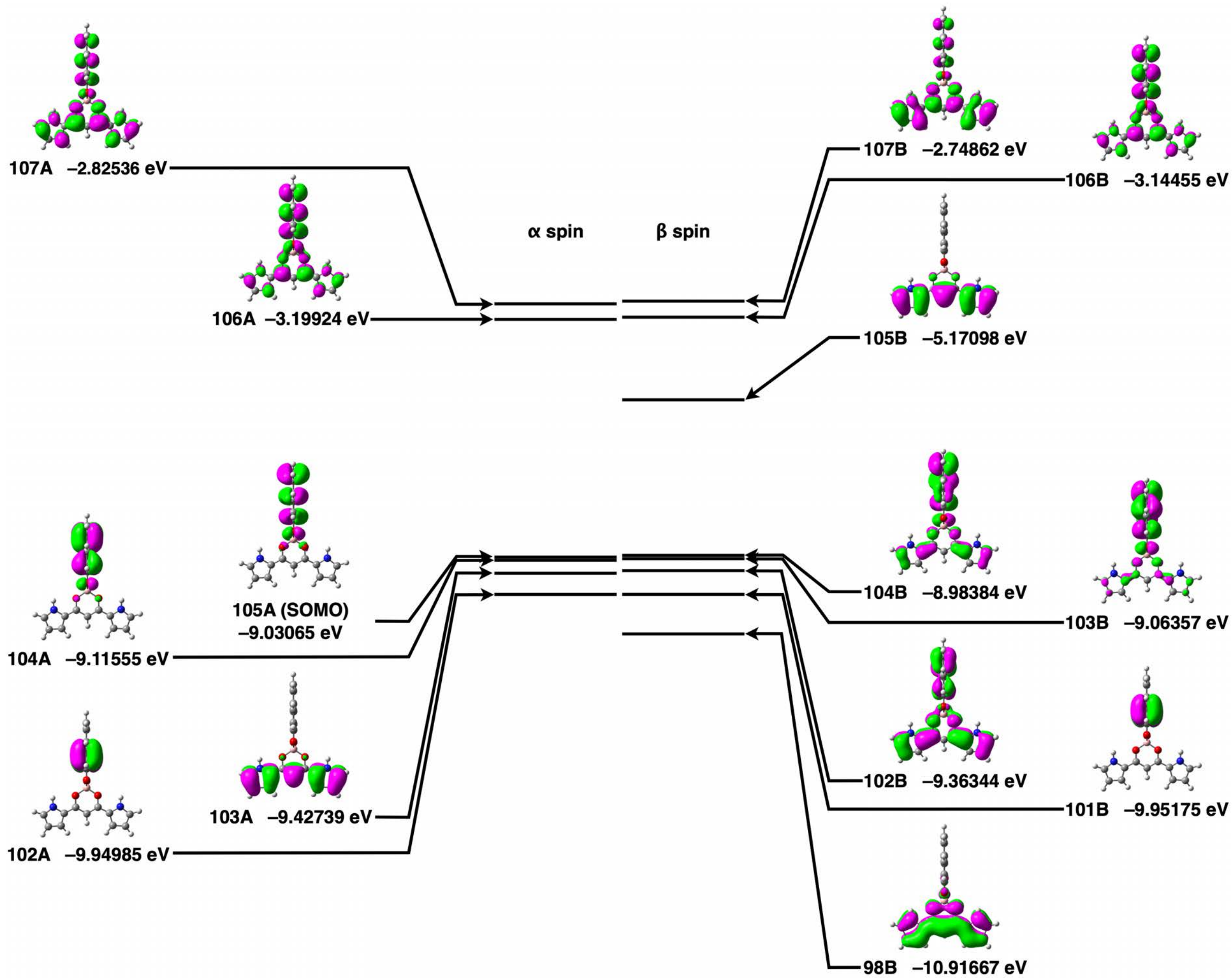

Fig. S50 Molecular orbitals (HOMO/LUMO) of oxidized-state **3a**$^+$ with α-spin (left) and β-spin (right) estimated at CPCM-CAM-B3LYP/6-31++G(d,p) (CH_2Cl_2). In 105A, the SOMO is localized on the phenalenyl unit, although the dipyrrolyldiketone unit should exhibit electron-donating behavior, as observed for **3b**$^+$ (Fig. S51). Such a result may be attributed to the weaker donating ability of the dipyrrolyldiketone unit compared to that of the π-extended **3b**$^+$.

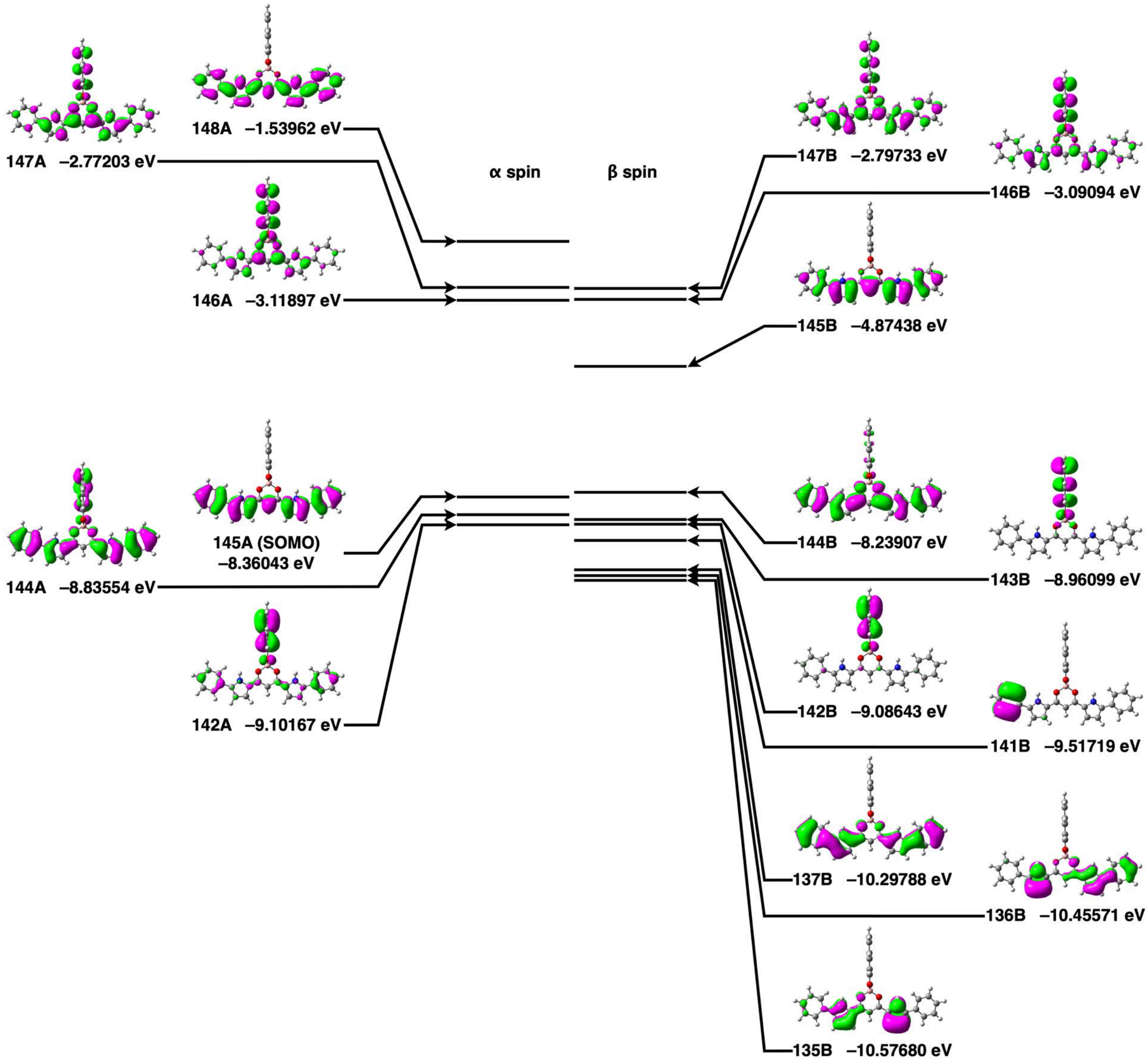

Fig. S51 Molecular orbitals (HOMO/LUMO) of oxidized-state **3b**$^{+}$ with α-spin (left) and β-spin (right) estimated at CPCM-CAM-B3LYP/6-31++G(d,p) (CH_2Cl_2). In 145A, the SOMO is localized on the dipyrrolyldiketone unit, suggesting electron-donating behavior of the dipyrrolyldiketone unit.

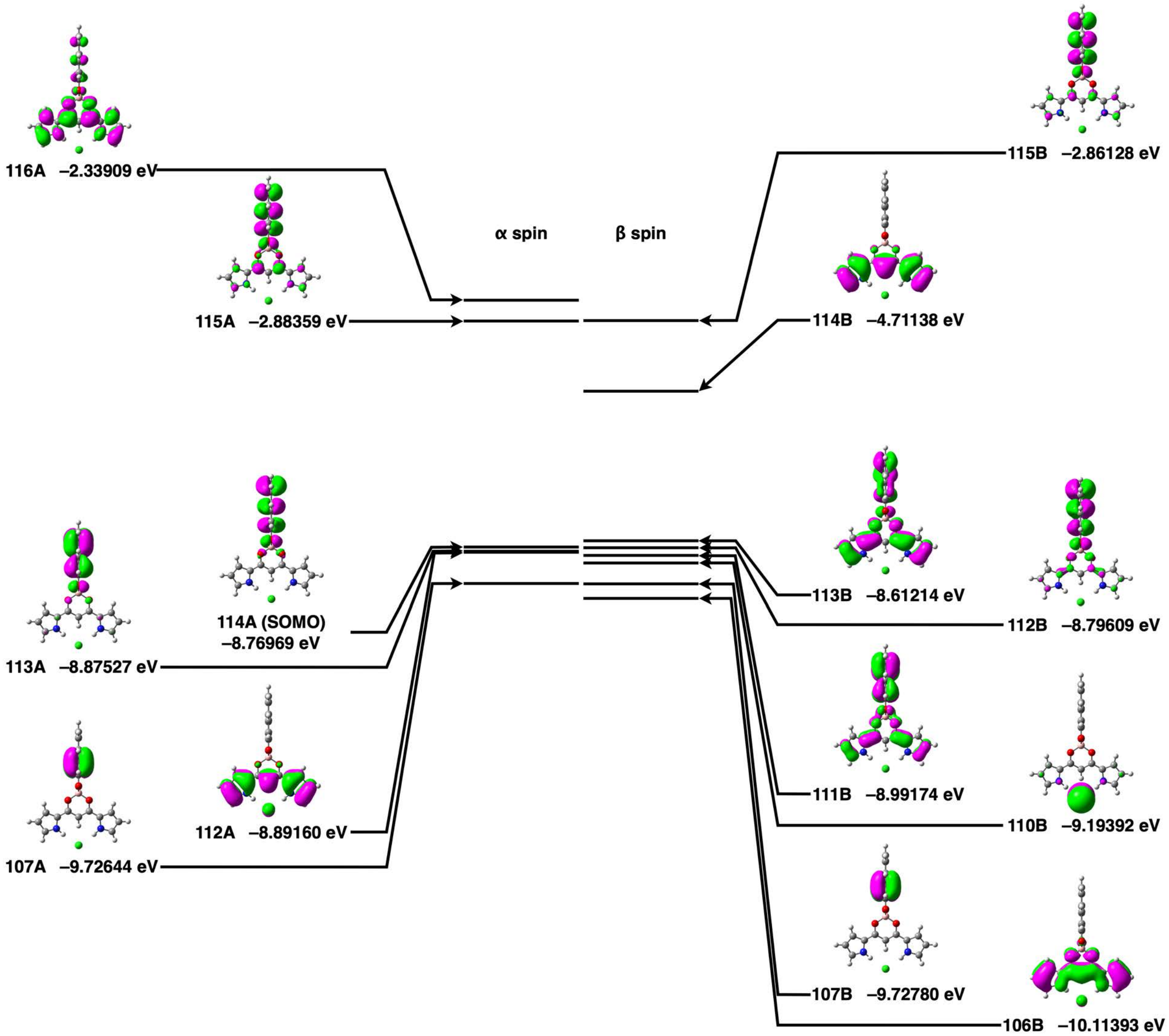

Fig. S52 Molecular orbitals (HOMO/LUMO) of oxidized-state **3a**$^+$-Cl$^-$ with α-spin (left) and β-spin (right) estimated at CPCM-CAM-B3LYP/6-31++G(d,p) (CH_2Cl_2). In 114A, the SOMO is localized on the phenalenyl unit, although the dipyrrolyldiketone unit should exhibit electron-donating behavior, as observed for **3b**$^+$·Cl$^-$ (Fig. S54). Such a result may be attributed to the weaker donating ability of the dipyrrolyldiketone unit compared to that of the π-extended **3b**$^+$.

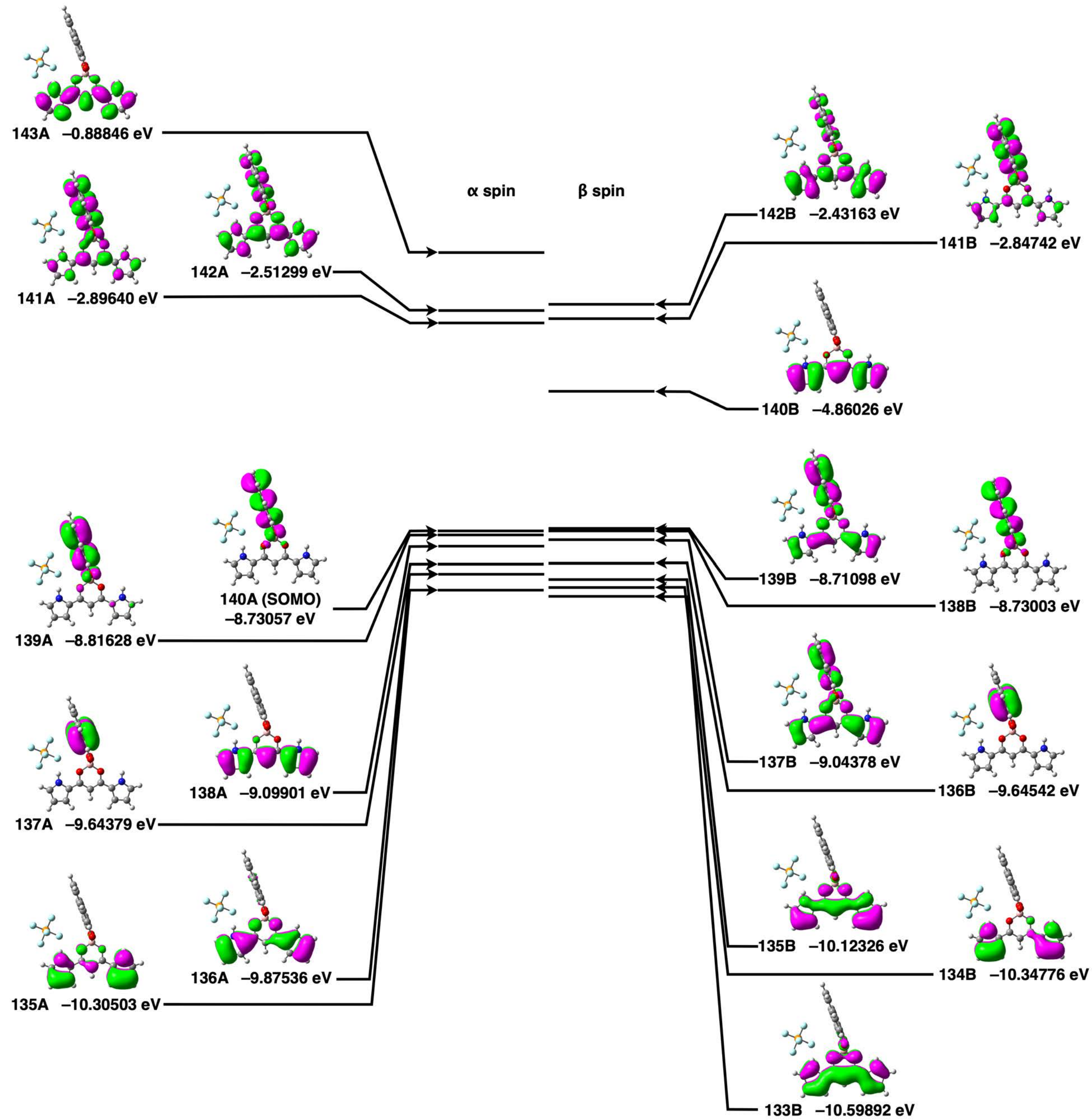

Fig. S53 Molecular orbitals (HOMO/LUMO) of oxidized-state **3a**$^{+}$-PF_6^{-} with α-spin (left) and β-spin (right) estimated at CPCM-CAM-B3LYP/6-31++G(d,p) (CH_2Cl_2). In 140A, the SOMO is localized on the phenalenyl unit, although the dipyrrolyldiketone unit should exhibit electron-donating behavior, as observed for **3b**$^{+}$·Cl^{-} (Fig. S54). Such a result may be attributed to the weaker donating ability of the dipyrrolyldiketone unit compared to that of the π-extended **3b**$^{+}$.

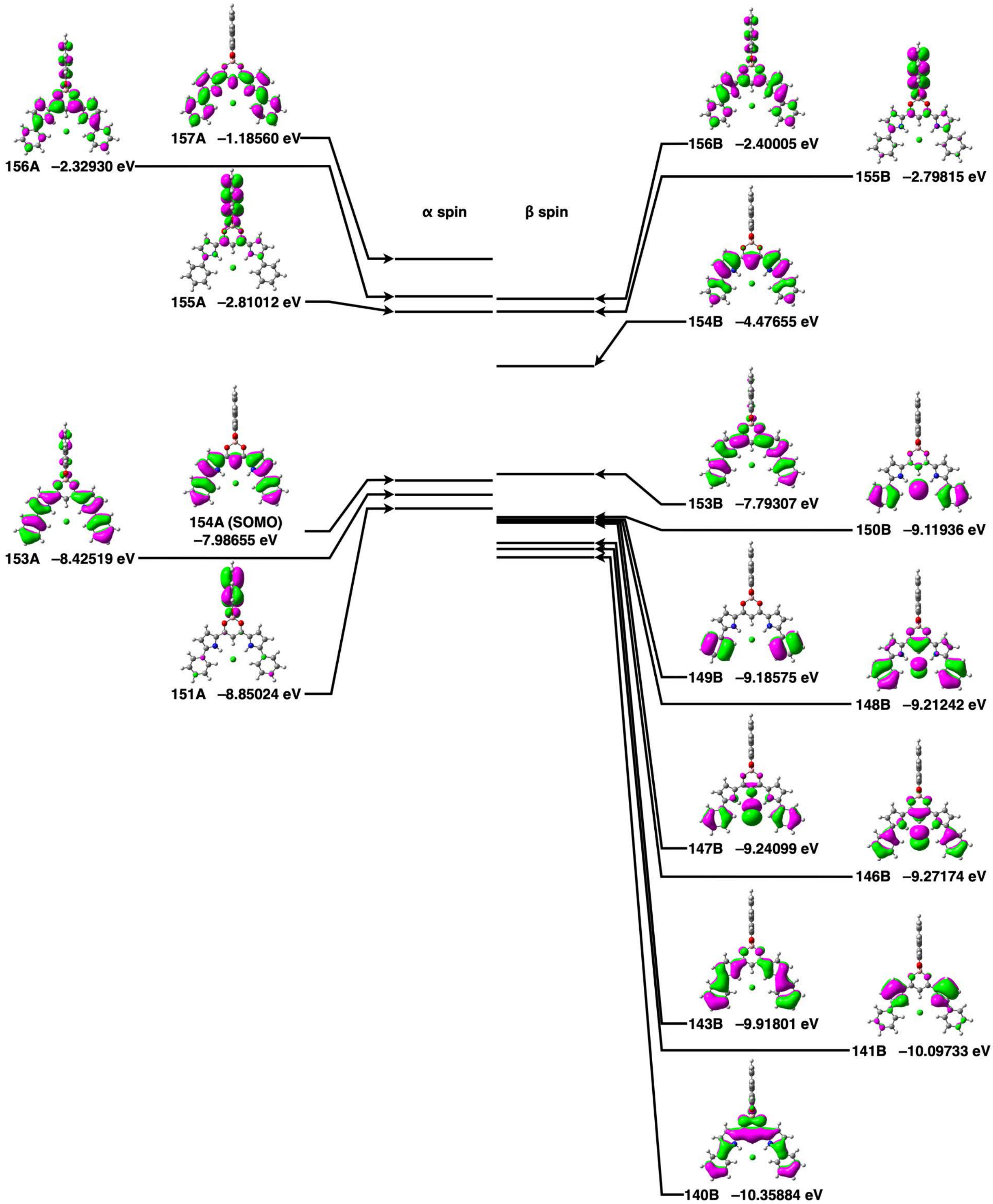

Fig. S54 Molecular orbitals (HOMO/LUMO) of oxidized-state **3b**$^{+}$-Cl^{-} with α-spin (left) and β-spin (right) estimated at CPCM-CAM-B3LYP/6-31++G(d,p) (CH_2Cl_2). In 154A, the SOMO is localized on the dipyrrolyldiketone unit, suggesting electron-donating behavior of the dipyrrolyldiketone unit.

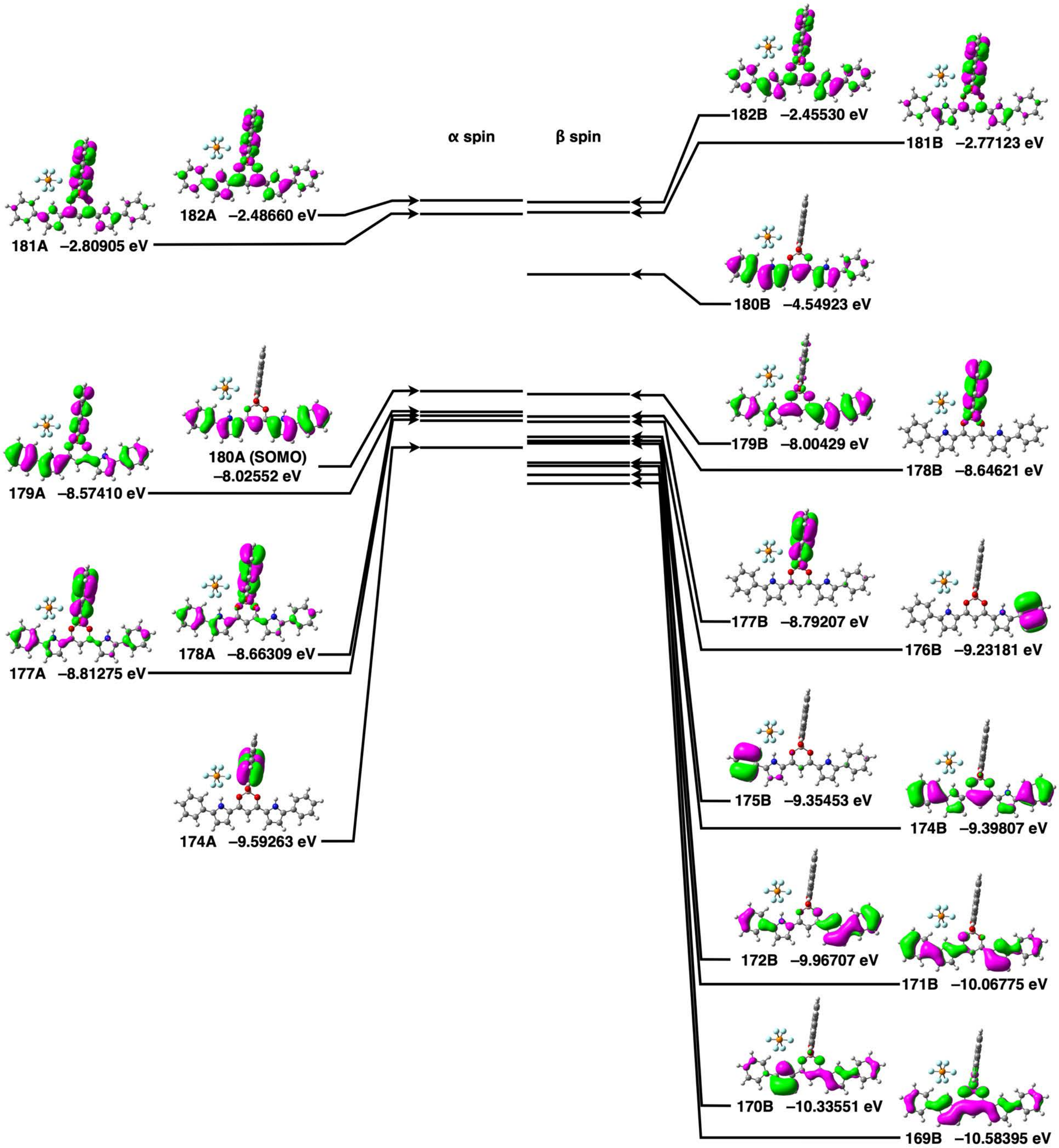

Fig. S55 Molecular orbitals (HOMO/LUMO) of oxidized-state **3b**$^{+}$-PF_6^{-} with α-spin (left) and β-spin (right) estimated at CPCM-CAM-B3LYP/6-31++G(d,p) (CH_2Cl_2). In 180A, the SOMO is localized on the dipyrrolyldiketone unit, suggesting electron-donating behavior of the dipyrrolyldiketone unit.

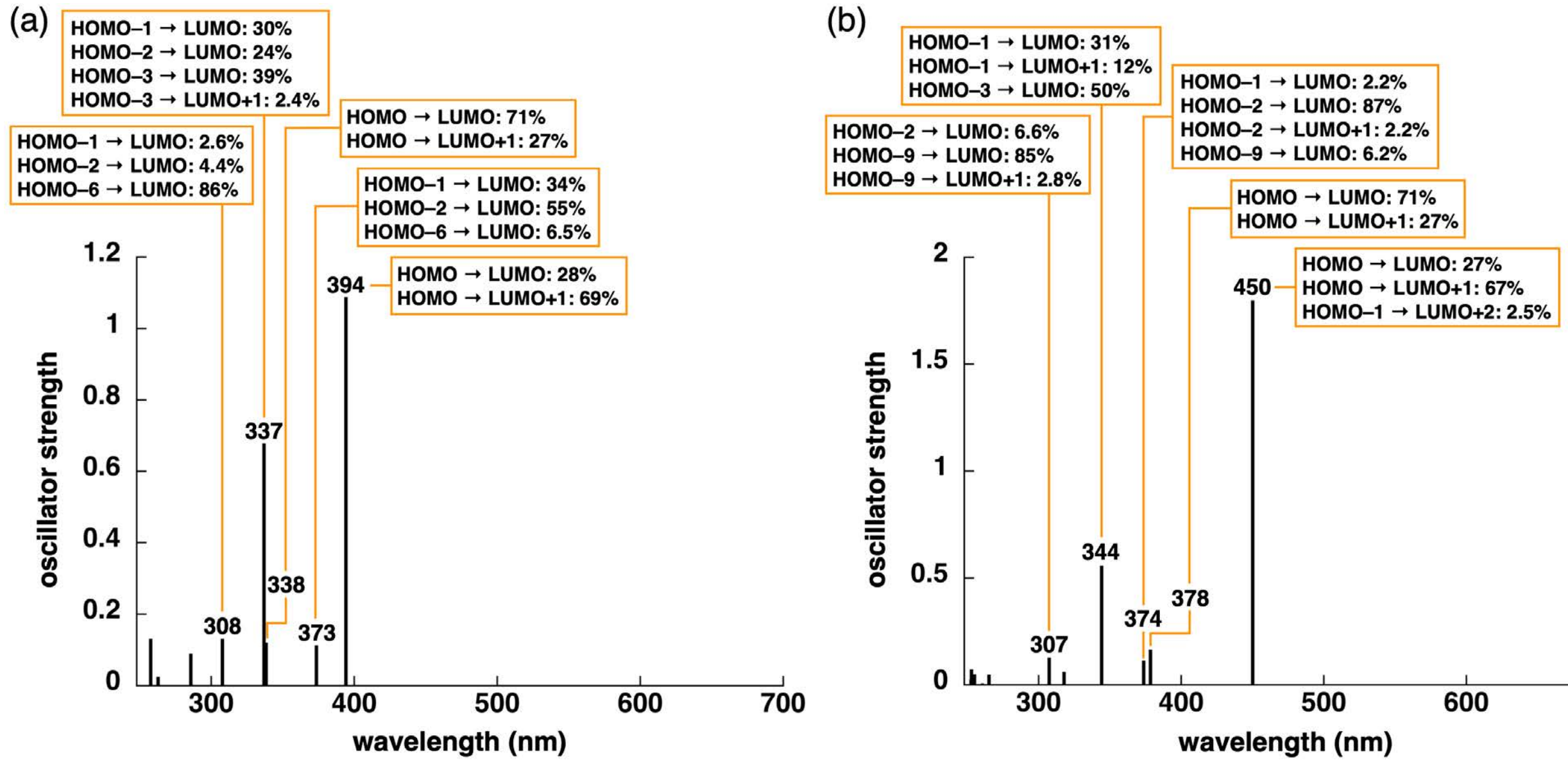

Fig. S56 TD-DFT-based UV/vis absorption stick spectra of (a) **3a**$^{+}$ and (b) **3b**$^{+}$ with the transitions correlated with molecular orbitals (MOs) estimated at CPCM-CAM-B3LYP/6-31++G(d,p) (CH_2Cl_2).

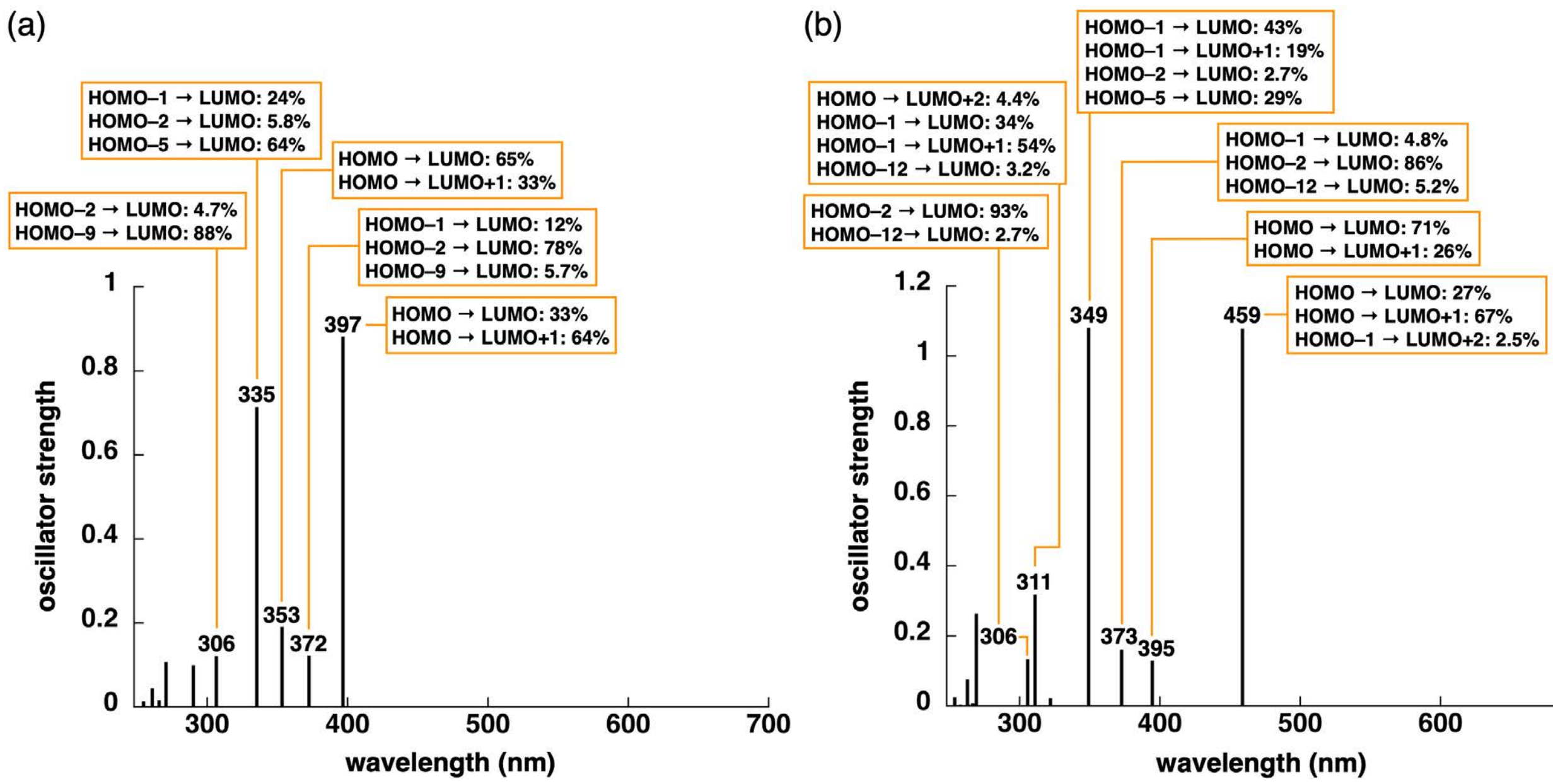

Fig. S57 TD-DFT-based UV/vis absorption stick spectra of (a) **3a**$^{+}$-Cl^{-} and (b) **3b**$^{+}$-Cl^{-} with the transitions correlated with molecular orbitals (MOs) estimated at CPCM-CAM-B3LYP/6-31++G(d,p) (CH_2Cl_2).

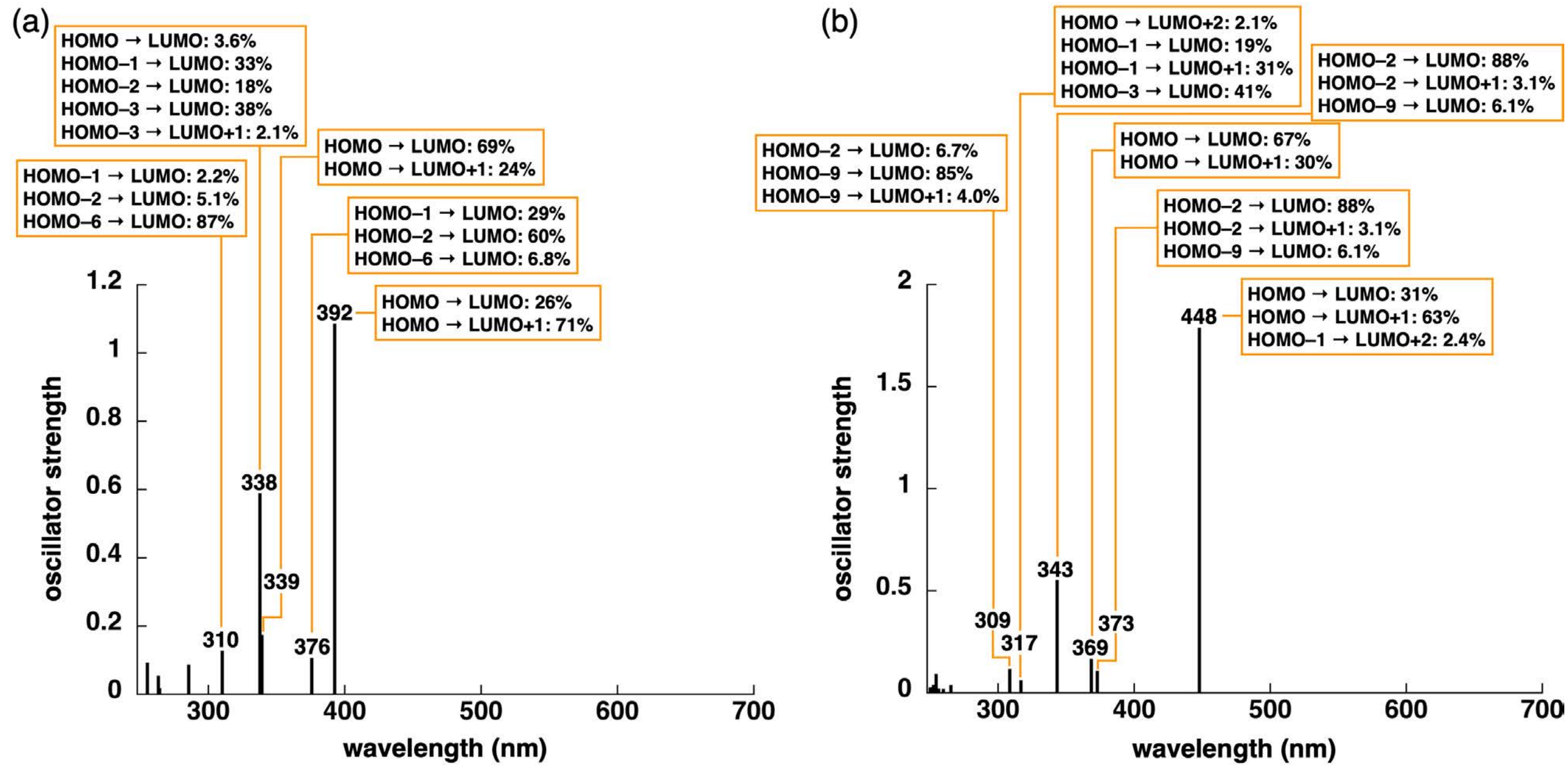

Fig. S58 TD-DFT-based UV/vis absorption stick spectra of (a) **3a**$^+$-PF_6^- and (b) **3b**$^+$-PF_6^- with the transitions correlated with molecular orbitals (MOs) estimated at CPCM-CAM-B3LYP/6-31++G(d,p) (CH_2Cl_2).

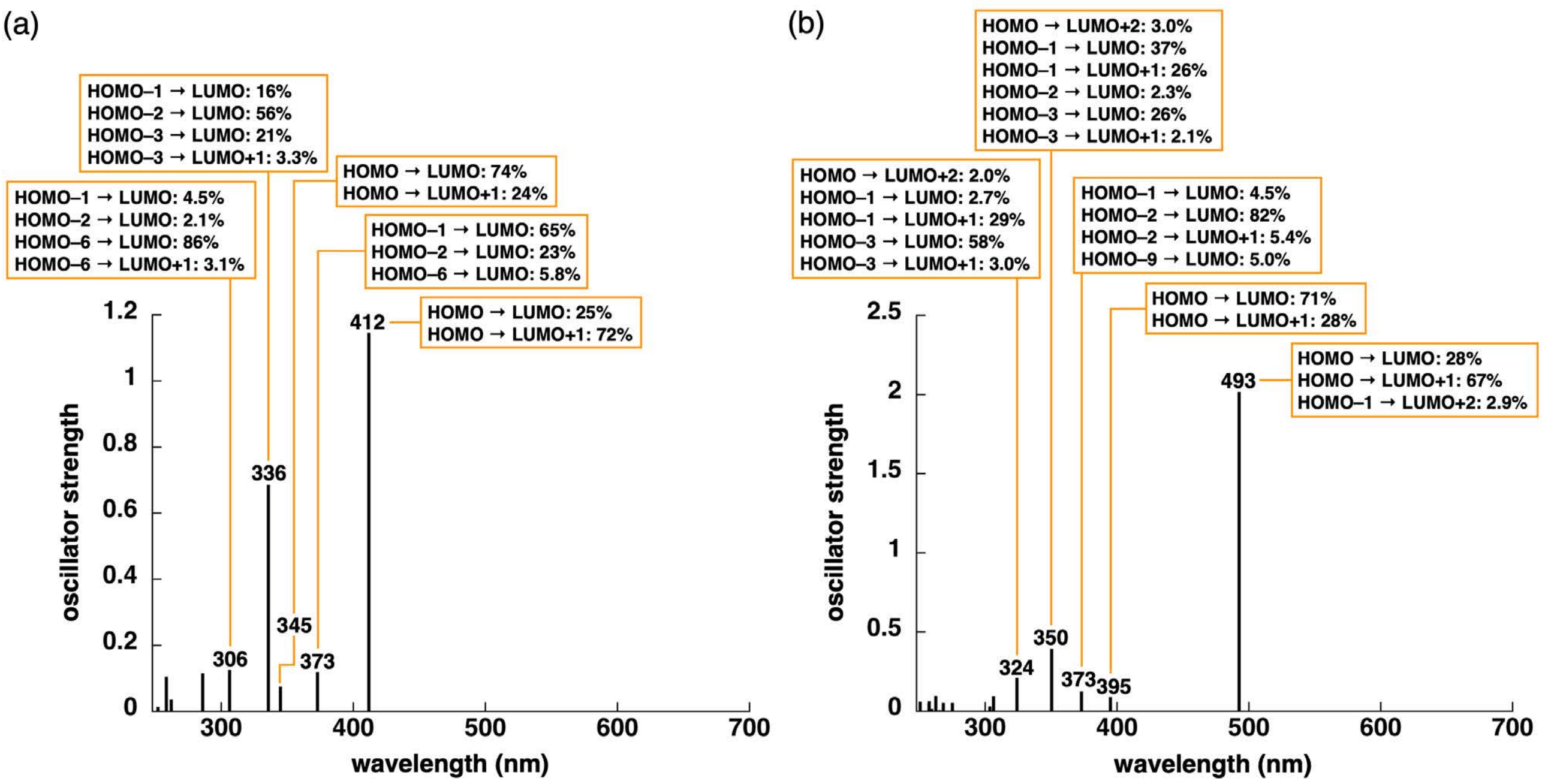

Fig. S59 TD-DFT-based UV/vis absorption stick spectra of S_1-state (a) **3a**$^+$ and (b) **3b**$^+$ with the transitions correlated with molecular orbitals (MOs) estimated at CPCM-CAM-B3LYP/6-31++G(d,p) (CH_2Cl_2).

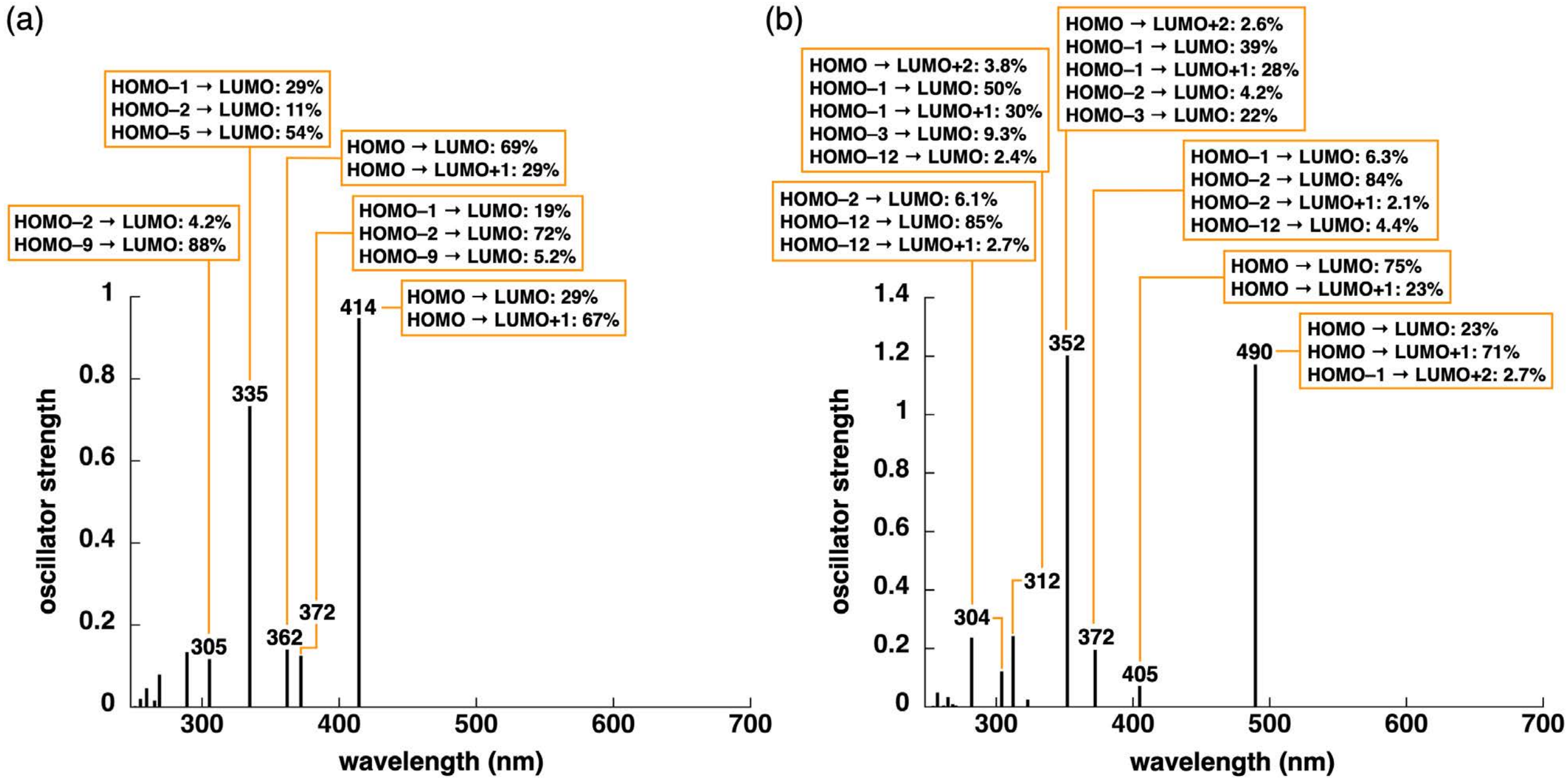

Fig. S60 TD-DFT-based UV/vis absorption stick spectra of S_1-state (a) **3a**$^+$-Cl$^-$ and (b) **3b**$^+$-Cl$^-$ with the transitions correlated with molecular orbitals (MOs) estimated at CPCM-CAM-B3LYP/6-31++G(d,p) (CH_2Cl_2).

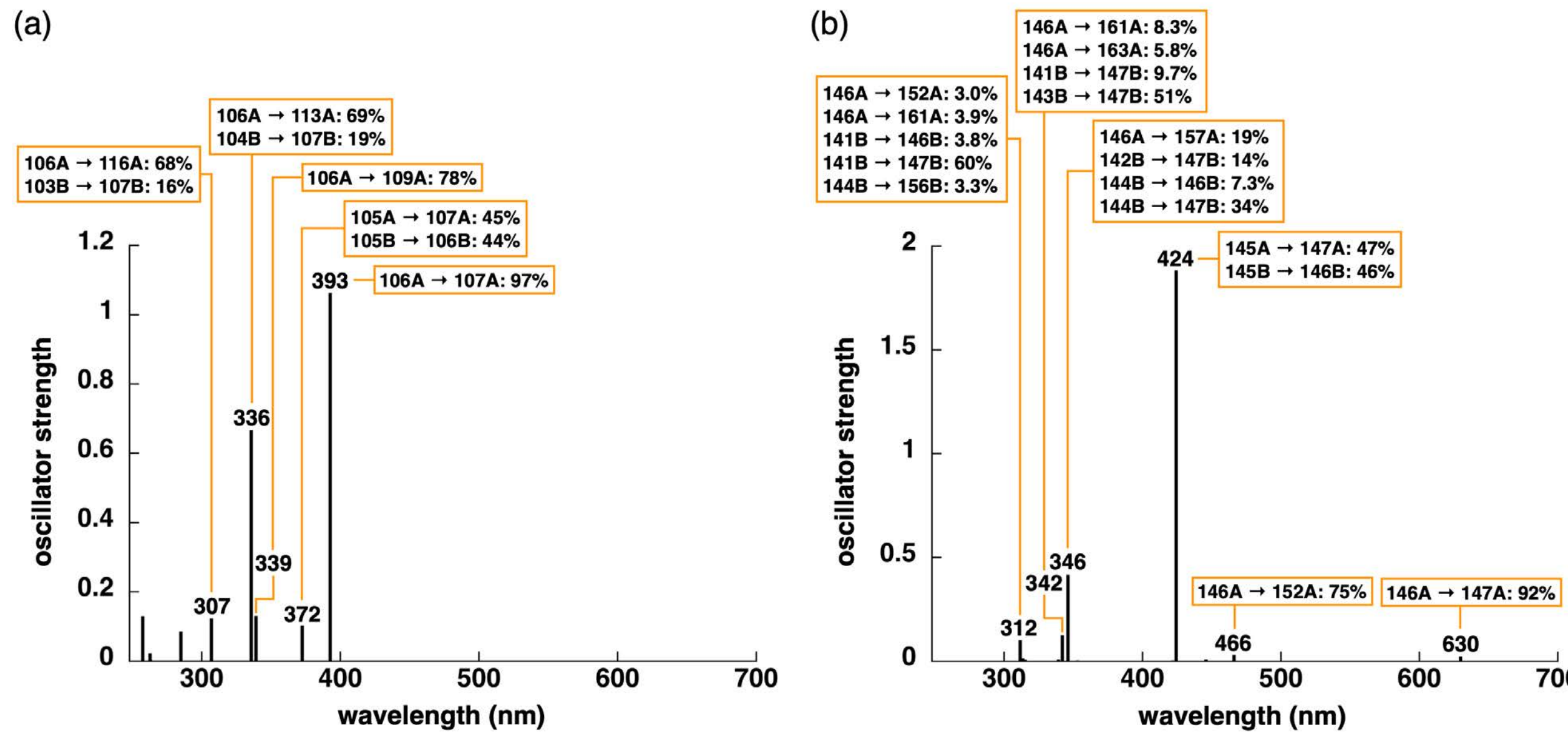

Fig. S61 TD-DFT-based UV/vis absorption stick spectra of reduced-state (a) **3a**$^+$ and (b) **3b**$^+$ with the transitions correlated with molecular orbitals (MOs) estimated at CPCM-CAM-B3LYP/6-31++G(d,p) (CH_2Cl_2).

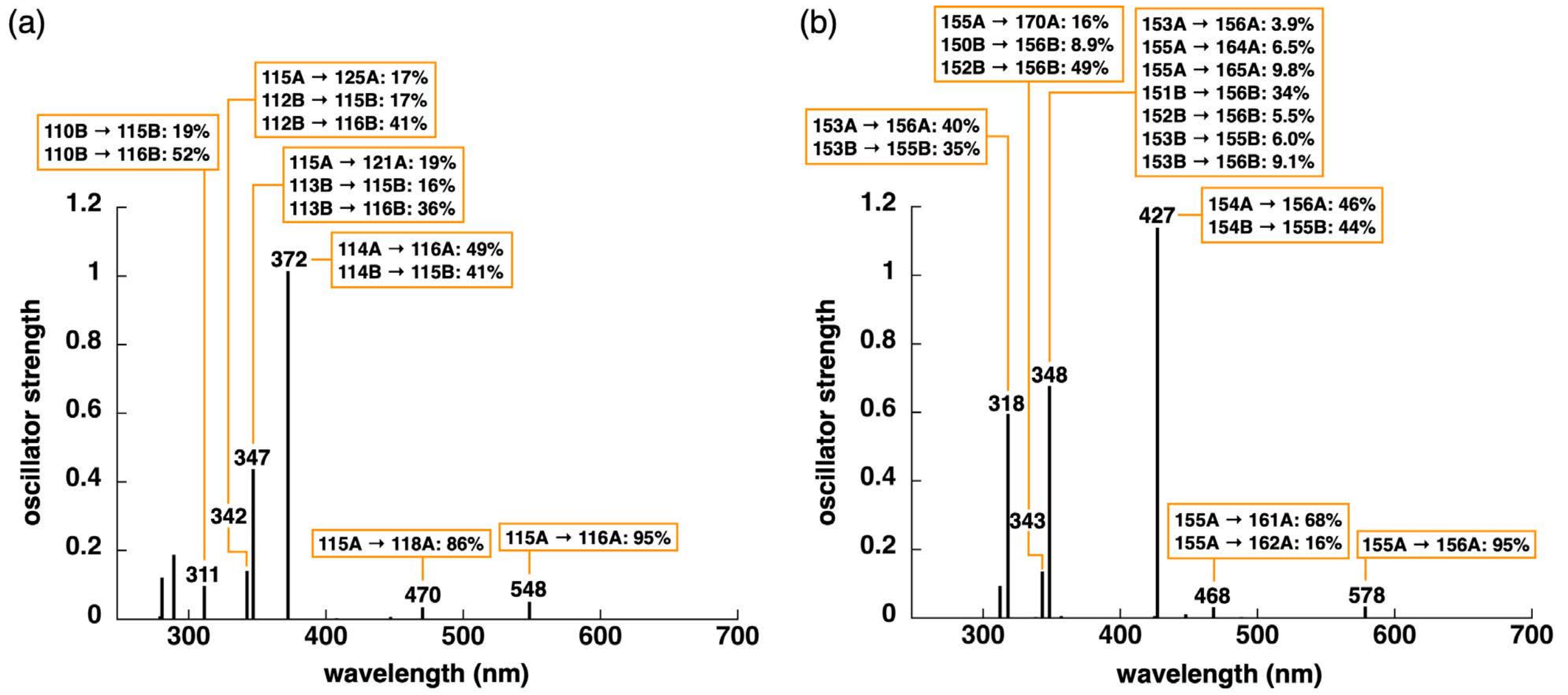

Fig. S62 TD-DFT-based UV/vis absorption stick spectra of reduced-state (a) **3a**$^{+}$-Cl^{-} and (b) **3b**$^{+}$-Cl^{-} with the transitions correlated with molecular orbitals (MOs) estimated at CPCM-CAM-B3LYP/6-31++G(d,p) (CH_2Cl_2).

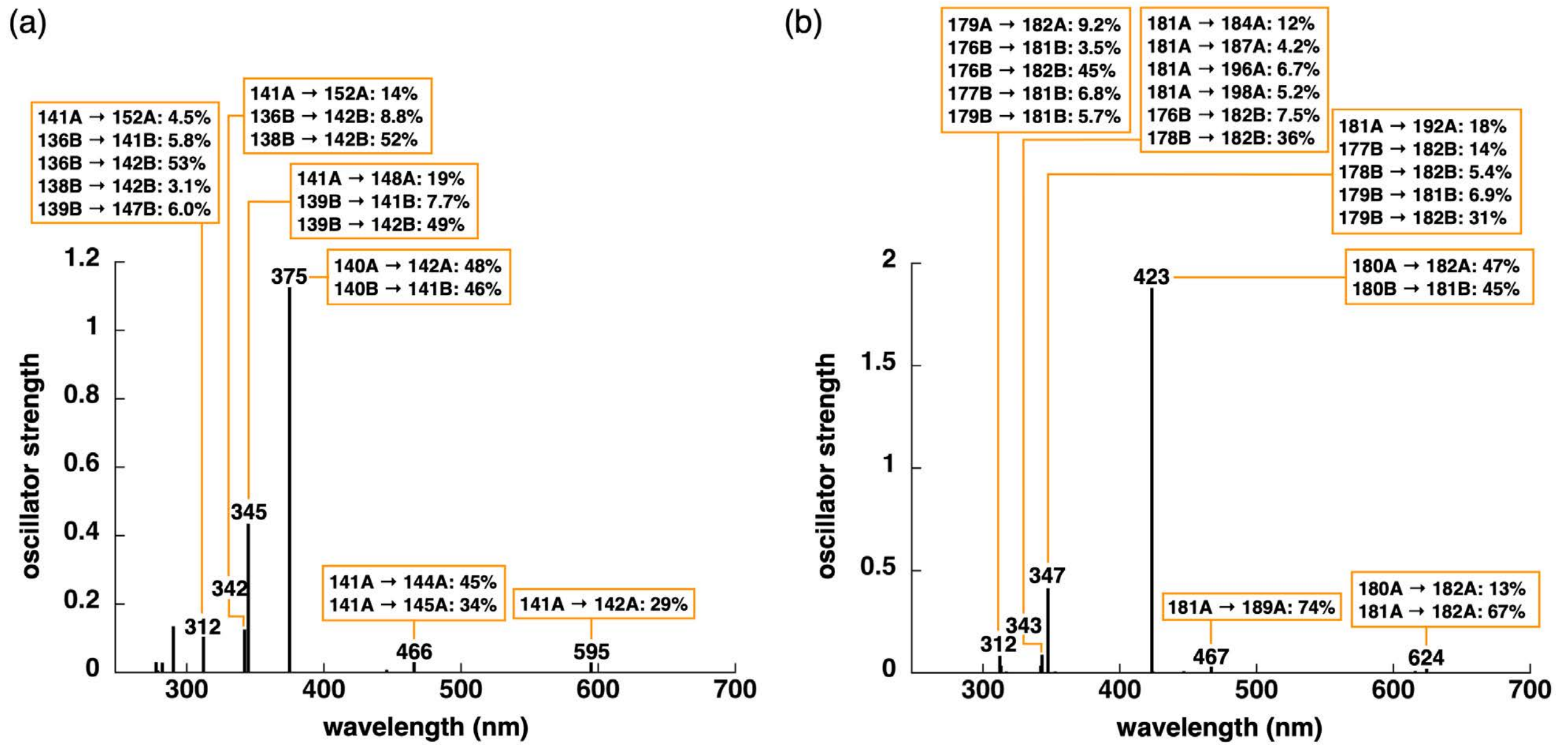

Fig. S63 TD-DFT-based UV/vis absorption stick spectra of reduced-state (a) **3a**$^{+}$-PF_6^{-} and (b) **3b**$^{+}$-PF_6^{-} with the transitions correlated with molecular orbitals (MOs) estimated at CPCM-CAM-B3LYP/6-31++G(d,p) (CH_2Cl_2).

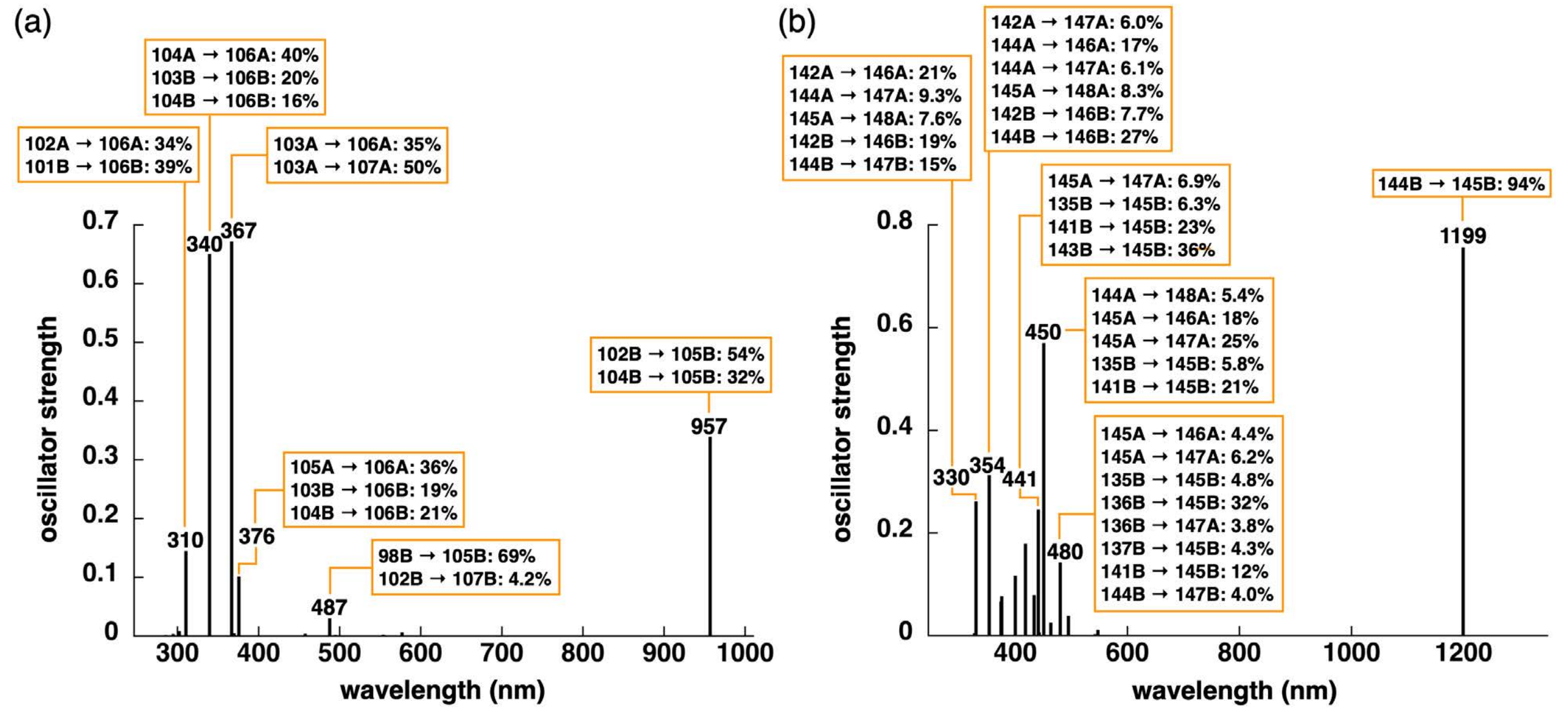

Fig. S64 TD-DFT-based UV/vis absorption stick spectra of oxidized-state (a) **3a**$^{+}$ and (b) **3b**$^{+}$ with the transitions correlated with molecular orbitals (MOs) estimated at CPCM-CAM-B3LYP/6-31++G(d,p) (CH_2Cl_2).

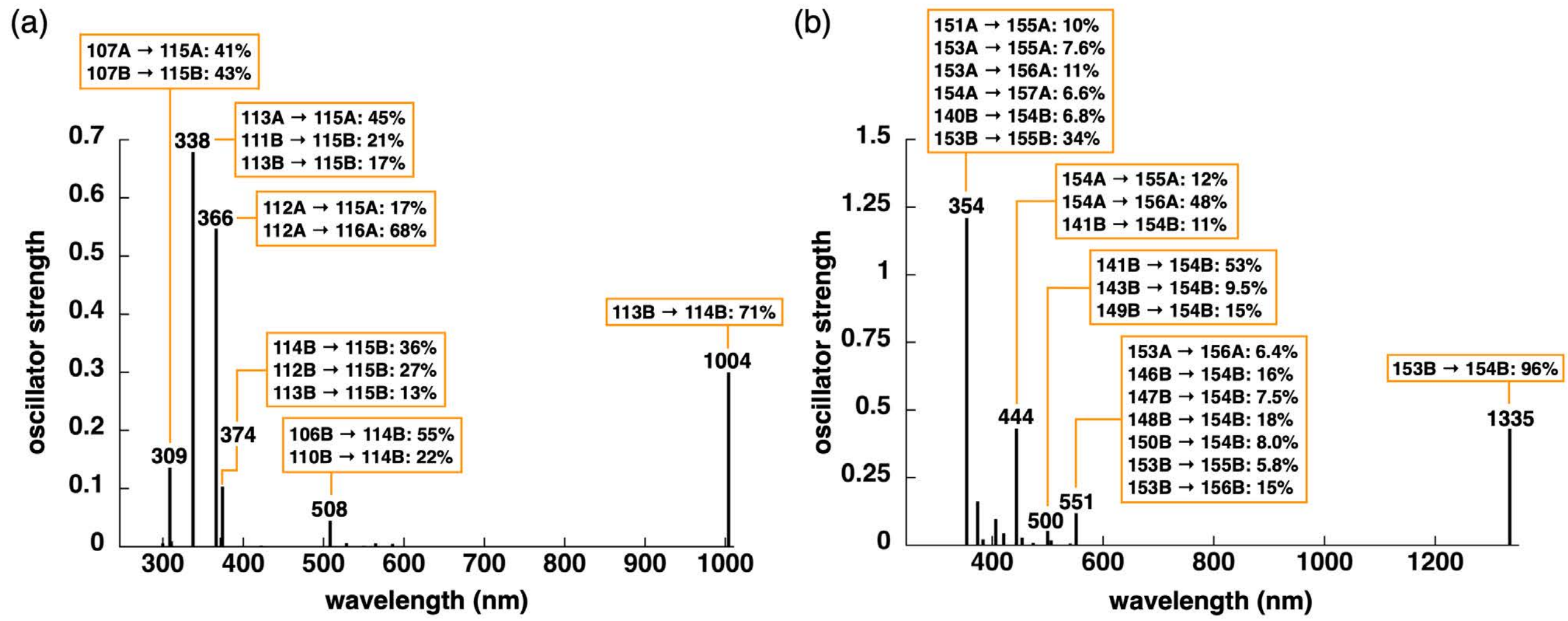

Fig. S65 TD-DFT-based UV/vis absorption stick spectra of oxidized-state (a) **3a**$^{+}$-Cl^{-} and (b) **3b**$^{+}$-Cl^{-} with the transitions correlated with molecular orbitals (MOs) estimated at CPCM-CAM-B3LYP/6-31++G(d,p) (CH_2Cl_2).

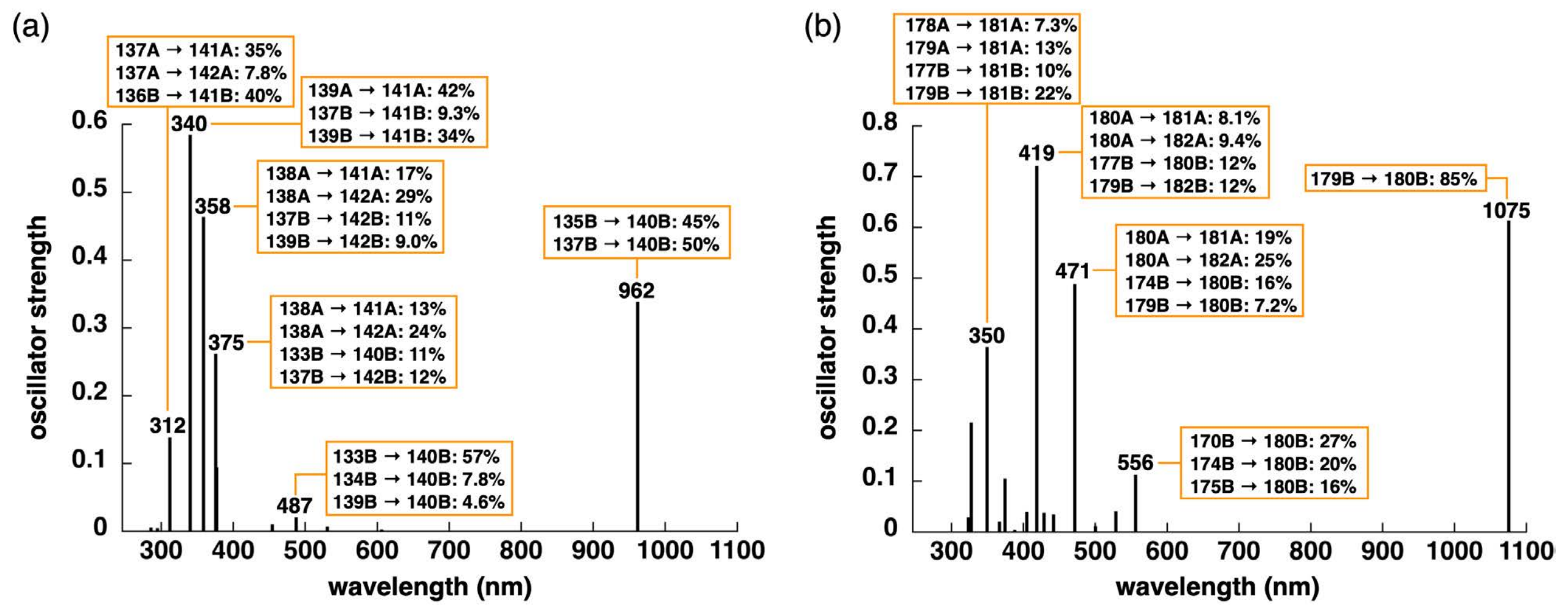

Fig. S66 TD-DFT-based UV/vis absorption stick spectra of oxidized-state (a) **3a**$^{+}$-PF_6^{-} and (b) **3b**$^{+}$-PF_6^{-} with the transitions correlated with molecular orbitals (MOs) estimated at CPCM-CAM-B3LYP/6-31++G(d,p) (CH_2Cl_2).

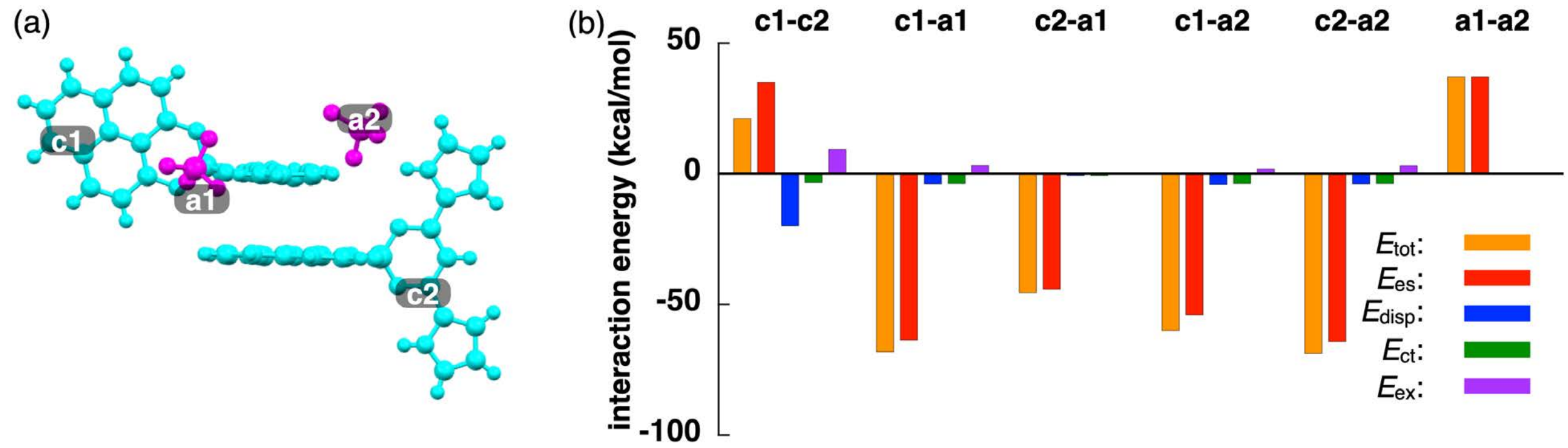

Fig. S67 Single-crystal X-ray structure of **3a**$^{+}$-BF_4^{-} for the EDA calculations: (a) packing structure (anion and cation are colored in magenta and cyan, respectively) and (b) intermolecular interaction energies (kcal/mol) between selected ions estimated at the FMO2-MP2 method using cc-pVDZ.[S11–13]

Table S3 Energies between selected fragments in **3a**$^{+}$-BF_4^{-} (Fig. S67) estimated by EDA calculations based on an FMO2-MP2 using basis set of cc-pVDZ.[S11–13]

fragments	total interaction energy (E_{tot}) (kcal/mol)	electrostatic interaction energy (E_{es}) (kcal/mol)	dispersion interaction energy (E_{disp}) (kcal/mol)	charge-transfer interaction energy (E_{ct+mix}) (kcal/mol)	exchange repulsion interaction energy (E_{ex}) (kcal/mol)
c1-c2	21.138	34.887	–19.822	–3.390	9.463
c1-a1	–68.217	–63.662	–3.933	–3.750	3.128
c2-a1	–45.596	–44.183	–0.769	–0.731	0.086
c1-a2	–60.156	–54.010	–4.190	–3.766	1.811
c2-a2	–68.811	–64.301	–3.896	–3.699	3.085
a1-a2	37.065	37.065	0.000	0.000	0.000

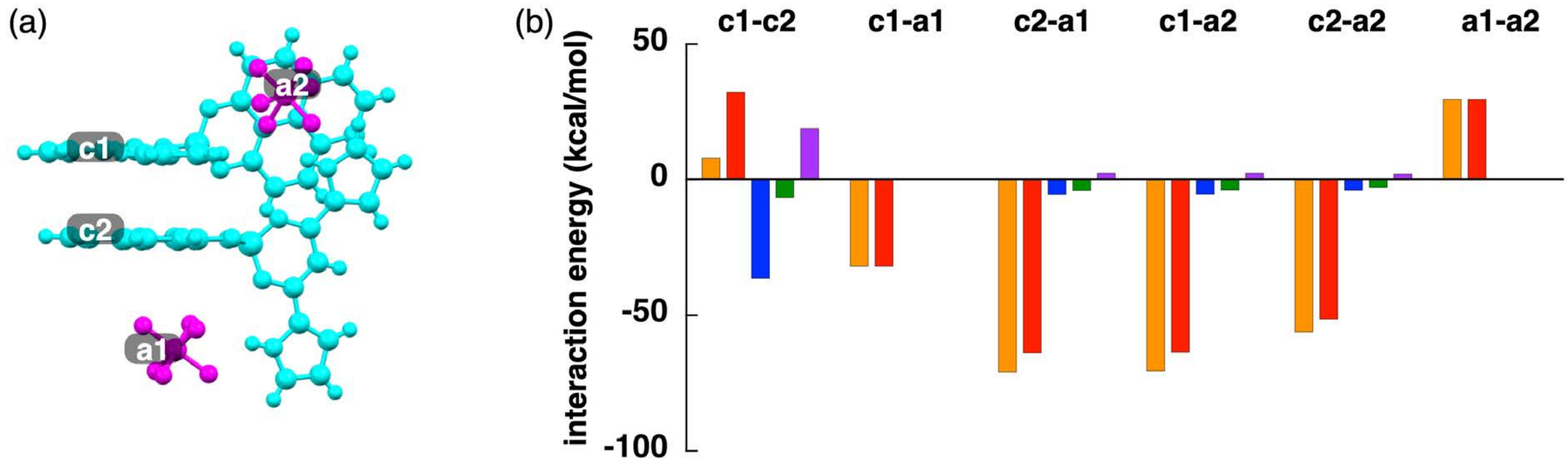

Fig. S68 Single-crystal X-ray structure of **3a**$^{+}$-PF_6^{-} for the EDA calculations: (a) packing structure (anion and cation are colored in magenta and cyan, respectively) and (b) intermolecular interaction energies (kcal/mol) between selected ions estimated at the FMO2-MP2 method using cc-pVDZ.[S11–13]

Table S4 Energies between selected fragments in **3a**$^{+}$-PF_6^{-} (Fig. S68) estimated by EDA calculations based on an FMO2-MP2 using basis set of cc-pVDZ.[S11–13]

fragments	total interaction energy (E_{tot}) (kcal/mol)	electrostatic interaction energy (E_{es}) (kcal/mol)	dispersion interaction energy (E_{disp}) (kcal/mol)	charge-transfer interaction energy (E_{ct+mix}) (kcal/mol)	exchange repulsion interaction energy (E_{ex}) (kcal/mol)
c1-c2	7.935	32.165	–36.406	–6.597	18.772
c1-a1	–31.899	–31.899	0.000	0.000	0.000
c2-a1	–70.978	–63.807	–5.542	–3.896	2.267
c1-a2	–70.478	–63.602	–5.457	–3.694	2.275
c2-a2	–56.202	–51.411	–3.800	–3.023	2.031
a1-a2	29.525	29.525	0.000	0.000	0.000

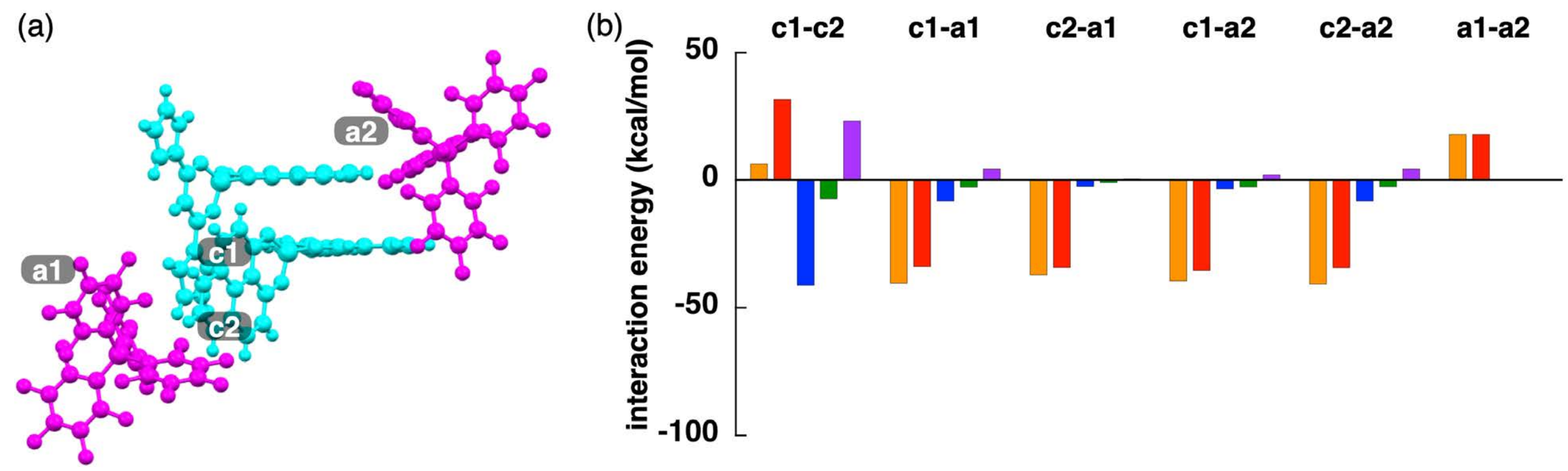

Fig. S69 Single-crystal X-ray structure of **3a**$^{+}$-B(C_6F_5)$_4^{-}$ for the EDA calculations: (a) packing structure (anion and cation are colored in magenta and cyan, respectively) and (b) intermolecular interaction energies (kcal/mol) between selected ions estimated at the FMO2-MP2 method using cc-pVDZ.[S11–13]

Table S5 Energies between selected fragments in **3a**$^{+}$-B(C_6F_5)$_4^{-}$ (Fig. S69) estimated by EDA calculations based on an FMO2-MP2 using basis set of cc-pVDZ.[S11–13]

fragments	total interaction energy (E_{tot}) (kcal/mol)	electrostatic interaction energy (E_{es}) (kcal/mol)	dispersion interaction energy (E_{disp}) (kcal/mol)	charge-transfer interaction energy (E_{ct+mix}) (kcal/mol)	exchange repulsion interaction energy (E_{ex}) (kcal/mol)
c1-c2	6.317	31.601	–41.275	–7.228	23.218
c1-a1	–40.403	–33.854	–8.147	–2.691	4.289
c2-a1	–37.157	–34.194	–2.446	–1.021	0.503
c1-a2	–39.475	–35.402	–3.462	–2.645	2.034
c2-a2	–40.689	–34.278	–8.146	–2.545	4.281
a1-a2	17.899	17.899	0.000	0.000	0.000

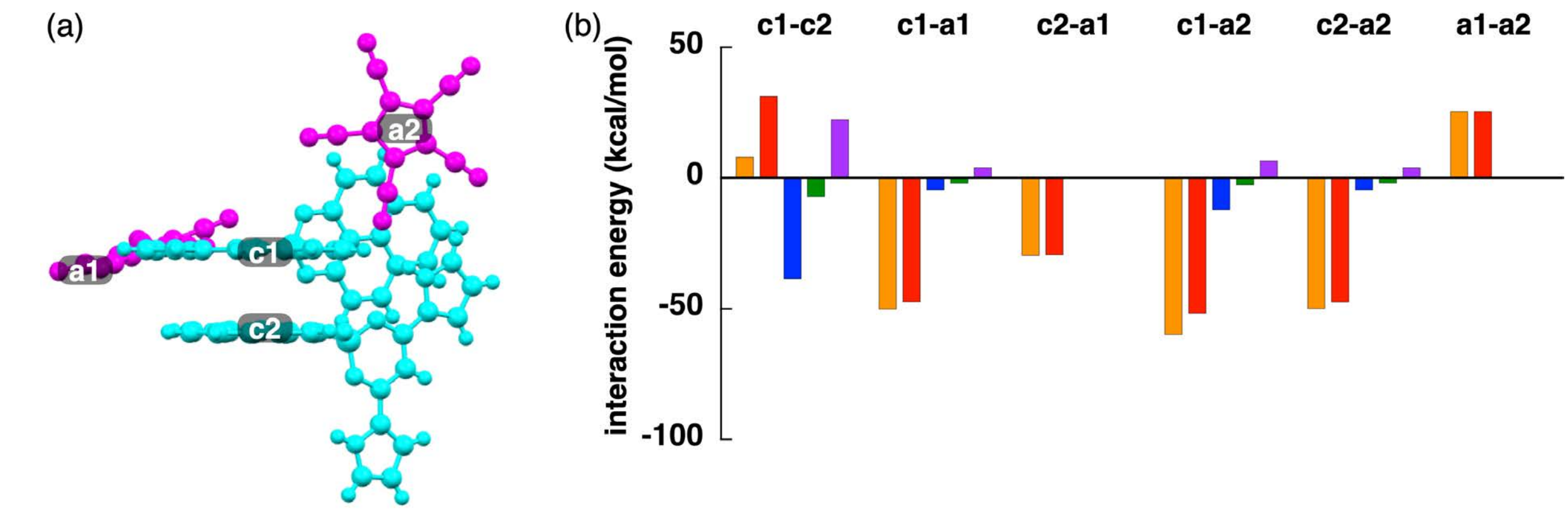

Fig. S70 Single-crystal X-ray structure of **3a**$^{+}$-PCCp^{-} for the EDA calculations: (a) packing structure (anion and cation are colored in magenta and cyan, respectively), and (b) intermolecular interaction energies (kcal/mol) between selected ions estimated at the FMO2-MP2 method using cc-pVDZ.[S11–13]

Table S6 Energies between selected fragments in **3a**$^{+}$-PCCp^{-} (Fig. S70) estimated by EDA calculations based on an FMO2-MP2 using basis set of cc-pVDZ.[S11–13]

fragments	total interaction energy (E_{tot}) (kcal/mol)	electrostatic interaction energy (E_{es}) (kcal/mol)	dispersion interaction energy (E_{disp}) (kcal/mol)	charge-transfer interaction energy (E_{ct+mix}) (kcal/mol)	exchange repulsion interaction energy (E_{ex}) (kcal/mol)
c1-c2	7.904	31.281	–38.496	–7.122	22.241
c1-a1	–50.127	–47.330	–4.630	–2.051	3.884
c2-a1	–29.604	–29.427	–0.170	–0.006	0.000
c1-a2	–59.877	–51.720	–12.183	–2.479	6.505
c2-a2	–49.954	–47.370	–4.593	–1.917	3.926
a1-a2	25.438	25.438	0.000	0.000	0.000

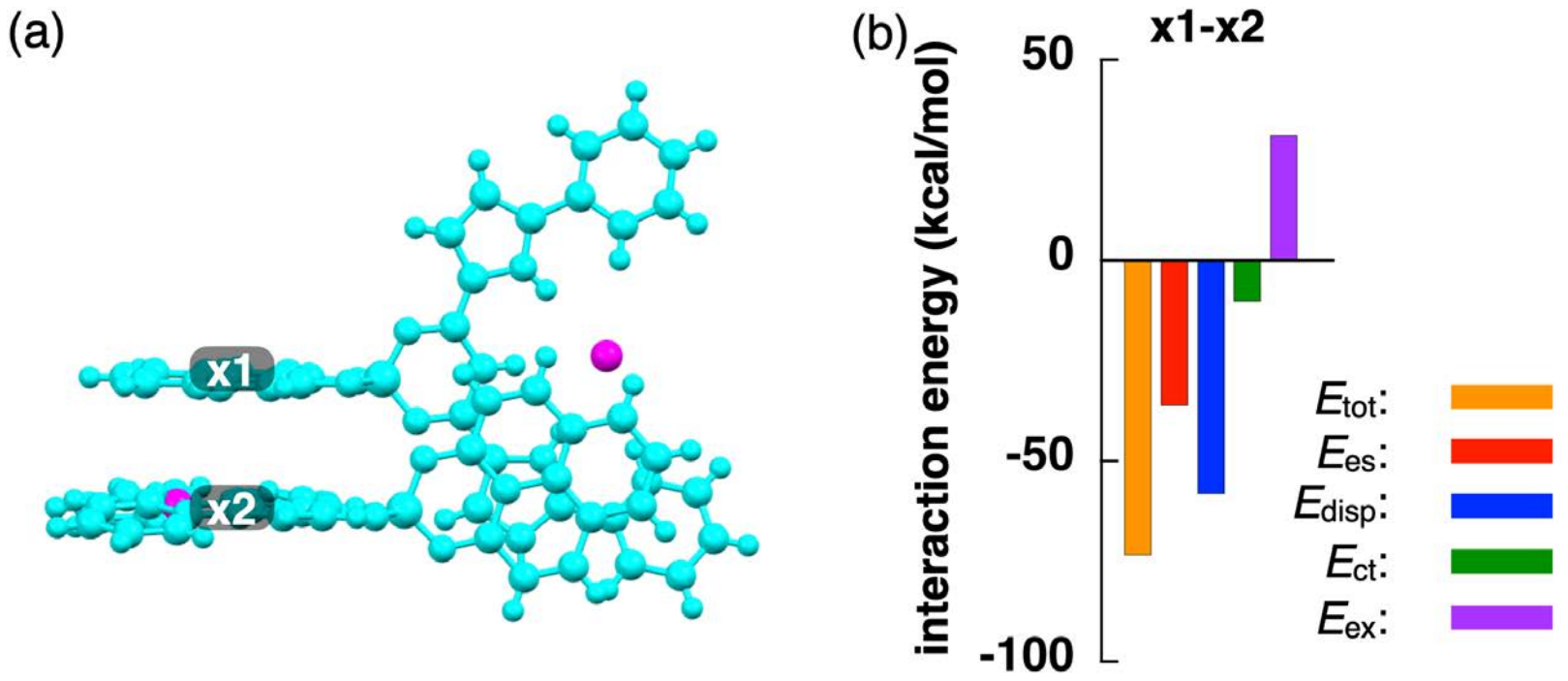

Fig. S71 Single-crystal X-ray structure of **3b**$^{+}$-Cl^{-} for the EDA calculations: (a) packing structure (anion and cation are colored in magenta and cyan, respectively) and (b) intermolecular interaction energies (kcal/mol) between selected ions estimated at the FMO2-MP2 method using cc-pVDZ.[S11–13]

Table S7 Energies between selected fragments in **3b**$^{+}$-Cl^{-} (Fig. S71) estimated by EDA calculations based on an FMO2-MP2 using basis set of cc-pVDZ.[S11–13]

fragments	total interaction energy (E_{tot}) (kcal/mol)	electrostatic interaction energy (E_{es}) (kcal/mol)	dispersion interaction energy (E_{disp}) (kcal/mol)	charge-transfer interaction energy (E_{ct+mix}) (kcal/mol)	exchange repulsion interaction energy (E_{ex}) (kcal/mol)
x1-x2	–73.518	–36.216	–58.23	–10.259	31.187

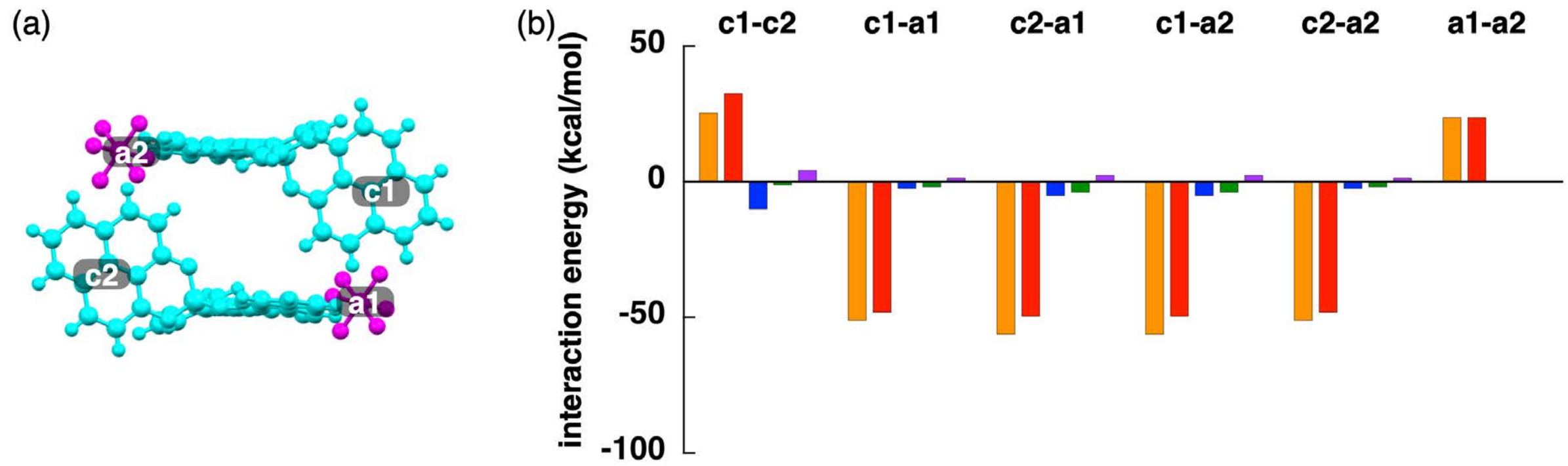

Fig. S72 Single-crystal X-ray structure of **3b**$^{+}$-PF_6^{-} for the EDA calculations: (a) packing structure (anion and cation are colored in magenta and cyan, respectively) and (b) intermolecular interaction energies (kcal/mol) between selected ions estimated at the FMO2-MP2 method using cc-pVDZ.[S11–13]

Table S8 Energies between selected fragments in **3b**$^{+}$-PF_6^{-} (Fig. S72) estimated by EDA calculations based on an FMO2-MP2 using basis set of cc-pVDZ.[S11–13]

fragments	total interaction energy (E_{tot}) (kcal/mol)	electrostatic interaction energy (E_{es}) (kcal/mol)	dispersion interaction energy (E_{disp}) (kcal/mol)	charge-transfer interaction energy (E_{ct+mix}) (kcal/mol)	exchange repulsion interaction energy (E_{ex}) (kcal/mol)
c1-c2	25.295	32.598	–10.125	–1.239	4.062
c1-a1	–51.142	–48.137	–2.417	–1.826	1.238
c2-a1	–56.277	–49.646	–5.026	–3.909	2.304
c1-a2	–56.276	–49.639	–5.026	–3.910	2.299
c2-a2	–51.138	–48.133	–2.419	–1.827	1.241
a1-a2	23.559	23.559	0.000	0.000	0.000

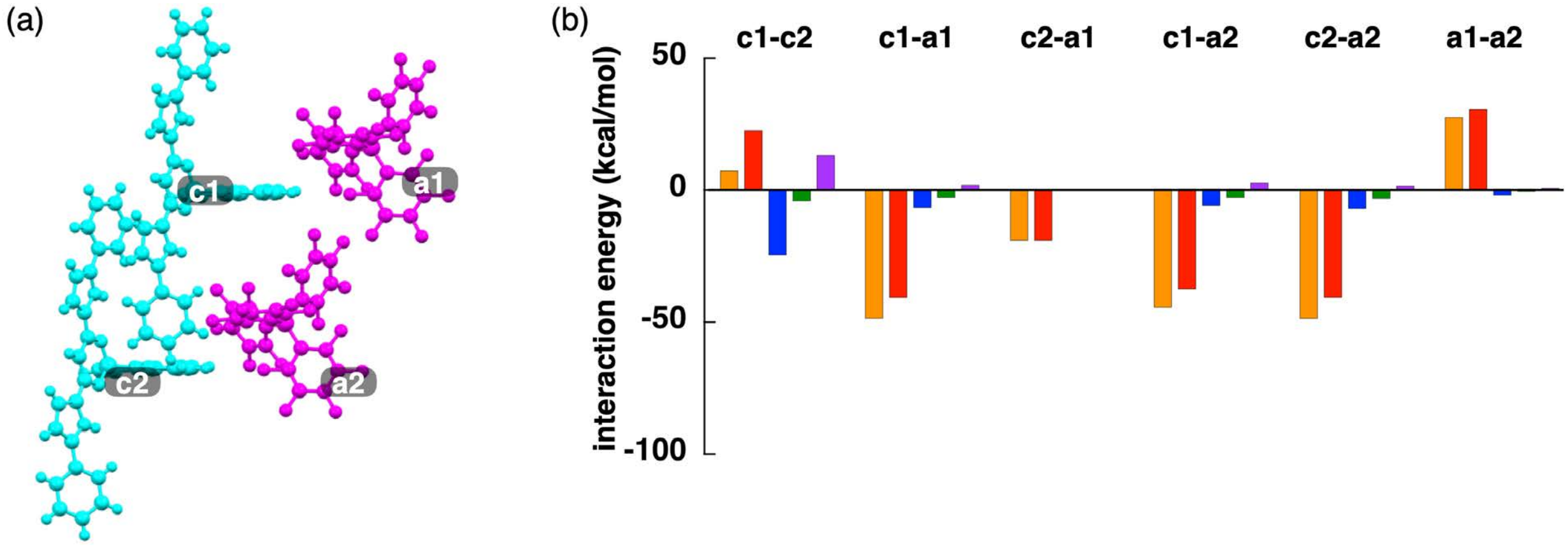

Fig. S73 Single-crystal X-ray structure of **3b**$^{+}$-B(C_6F_5)$_4^{-}$ for the EDA calculations: (a) packing structure (anion and cation are colored in magenta and cyan, respectively) and (b) intermolecular interaction energies (kcal/mol) between selected ions estimated at the FMO2-MP2 method using cc-pVDZ.[S11–13]

Table S9 Energies between selected fragments in **3b**$^{+}$-B(C_6F_5)$_4^{-}$ (Fig. S73) estimated by EDA calculations based on an FMO2-MP2 using basis set of cc-pVDZ.[S11–13]

fragments	total interaction energy (E_{tot}) (kcal/mol)	electrostatic interaction energy (E_{es}) (kcal/mol)	dispersion interaction energy (E_{disp}) (kcal/mol)	charge-transfer interaction energy (E_{ct+mix}) (kcal/mol)	exchange repulsion interaction energy (E_{ex}) (kcal/mol)
c1-c2	7.263	22.579	–24.449	–4.010	13.144
c1-a1	–48.635	–40.721	–6.701	–2.940	1.726
c2-a1	–18.926	–18.926	0.000	0.000	0.000
c1-a2	–44.746	–37.788	–6.129	–3.139	2.309
c2-a2	–48.396	–40.418	–6.741	–2.979	1.742
a1-a2	27.297	30.175	–2.217	–0.950	0.288

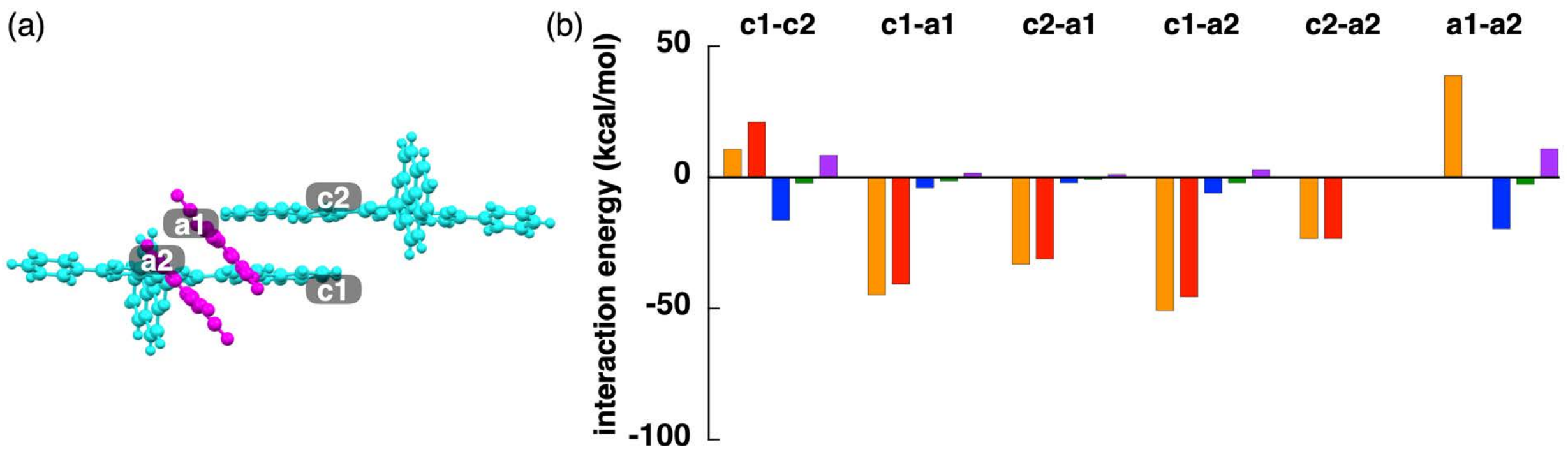

Fig. S74 Single-crystal X-ray structure of **3b**$^{+}$-PCCp^{-} for the EDA calculations: (a) packing structure (anion and cation are colored in magenta and cyan, respectively) and (b) intermolecular interaction energies (kcal/mol) between selected ions estimated at the FMO2-MP2 method using cc-pVDZ.[S11–13]

Table S10 Energies between selected fragments in **3b**$^{+}$-PCCp^{-} (Fig. S74) estimated by EDA calculations based on an FMO2-MP2 using basis set of cc-pVDZ.[S11–13]

fragments	total interaction energy (E_{tot}) (kcal/mol)	electrostatic interaction energy (E_{es}) (kcal/mol)	dispersion interaction energy (E_{disp}) (kcal/mol)	charge-transfer interaction energy (E_{ct+mix}) (kcal/mol)	exchange repulsion interaction energy (E_{ex}) (kcal/mol)
c1-c2	10.622	20.987	–16.326	–2.249	8.211
c1-a1	–44.925	–40.683	–4.195	–1.459	1.412
c2-a1	–33.236	–31.210	–2.134	–0.912	1.019
c1-a2	–50.989	–45.615	–6.073	–2.117	2.816
c2-a2	–23.404	–23.404	0.000	0.000	0.000
a1-a2	38.778	50.321	–19.601	–2.689	10.748

Cartesian Coordination of 3a^{+}-1

–1358.8225623 hartree

C,-2.1243490481,0.1409939308,0.0554060727

C,-1.4801672511,-0.0353245509,-1.1629037583

C,-1.4173604647,-0.0663668033,1.2333220694

C,-1.9988958246,-0.020882968,2.5333380149

C,-3.3133905942,0.1432997436,4.3580832327

C,-2.1289890173,0.043377183,-2.4290526722

C,-3.4548647399,0.2950902209,-2.7792957994

N,-1.2380830322,-0.2405659509,3.6617789284

N,-1.4284991632,-0.1465787822,-3.6011034558
H,-0.2514866366,-0.450898662,3.6406253119
H,-0.4422935409,-0.3567297108,-3.6371077221
C,-3.5371451185,0.2538905311,-4.1775731331
C,-3.3044371234,0.2207536177,2.9589501352
B,0.6544312373,-0.278512042,-0.02286201
O,-0.1900621735,-0.3211265225,-1.2115732491
O,-0.1266476134,-0.3523656364,1.2069255771
O,1.5524916494,-1.4322612261,-0.061350192
C,2.6890067632,1.0931875934,-0.0581125921
C,3.4851939155,-0.0707540104,-0.0938016866
C,3.3064556046,2.3698560086,-0.057631365
C,4.8973470613,0.0275652961,-0.1291616266
C,2.8579366028,-1.3328877668,-0.0939095258
C,4.6700420839,2.4583909506,-0.0918275669
H,2.6744777286,3.2495160799,-0.0298811023
C,5.6804217655,-1.1485140792,-0.1647033448
C,5.5106889425,1.3010900387,-0.128586394
C,3.6445090911,-2.5114163933,-0.1295950499
H,5.1397642562,3.4377677638,-0.091345783
C,5.0076645939,-2.4107134451,-0.1636288716
C,7.077629768,-1.0287076365,-0.1993854045
C,6.9103538028,1.37630564,-0.1639087432
H,3.1390120692,-3.4697734431,-0.1289218842
C,7.6852854359,0.2217866999,-0.1989282725
H,7.3909701796,2.350118978,-0.1637750622
H,8.766173045,0.2973527714,-0.2259890845
O,1.3850875415,1.0055137331,-0.0254348261
H,7.6879122365,-1.926530767,-0.2268256621
H,5.6074451917,-3.3158633848,-0.1908971542
H,-4.2676012926,0.4862911881,-2.0937490588
H,-4.4190524702,0.4064675465,-4.7813845045
H,-4.1518430279,0.4290658767,2.3220963426
H,-4.1623629924,0.2795588243,5.0110057376
H,-3.1773396838,0.3753516906,0.0860421551
C,-2.0175949778,-0.1458802553,4.7611432855
C,-2.2644468899,-0.0239284863,-4.6553075463
H,-1.6116634723,-0.2883366423,5.7517941273
H,-1.9110702176,-0.1404501217,-5.6692162766

Cartesian Coordination of 3a^{+}-2

–1358.8187397 hartree
C,-3.216844188,0.0295414306,-0.1237184618
C,-2.4304215853,-1.1065214433,0.0497976362
C,-2.6472989895,1.2869678576,0.0490142719
C,-3.3760024415,2.5105786607,-0.0065621318
C,-4.8899946259,4.1736396178,-0.1714004397
C,-2.9136209869,-2.4479571049,-0.0045200009
N,-2.7411969756,3.7213476513,0.1695676392
H,-1.7525996077,3.8141048762,0.3506608089
H,-1.1335102174,-3.6979088746,0.2841634034
C,-4.3591490056,-4.1402541863,-0.2039768109
C,-4.726612607,2.7829684496,-0.2208962283
B,-0.4438466432,0.2859841623,0.2443772831
O,-1.1443750848,-0.9919432131,0.3066238018
O,-1.3592620361,1.4182999944,0.3032369314
O,0.4546977388,0.3703114997,1.3951760883
C,1.5764338547,0.1841579987,-1.128335202
C,2.3673344333,0.0868381496,0.0356333636
C,2.1892658637,0.1131665268,-2.4049879028
C,3.7697945822,-0.0806211763,-0.0629477142
C,1.74983017,0.2015835462,1.2977717264
C,3.5417635117,-0.0639266486,-2.4937900478
H,1.5618306209,0.1937296116,-3.2846099409
C,4.5510288241,-0.1495941242,1.1129048851
C,4.3767440616,-0.1672465845,-1.3365667291
C,2.5364999747,0.148456346,2.475484237
H,4.006999588,-0.1272811256,-3.4732579256
C,3.8883845933,-0.0287824645,2.3748173325
C,5.9380067082,-0.3202550968,0.9929695955
C,5.766533416,-0.3374098912,-1.4119429873
H,2.0388863732,0.2418179341,3.4333749823
C,6.5382505405,-0.4161531743,-0.2575759565
H,6.2420954626,-0.4054873431,-2.3858592642
H,7.6114263844,-0.5479081789,-0.3331435674
O,0.2822009504,0.3520474264,-1.039281445
H,6.5467168377,-0.3749978621,1.8906138844
H,4.4873151179,-0.0787905599,3.2795561144
H,-5.0089147966,-2.1520221056,-0.3146275394
H,-5.3196441077,-4.6146051368,-0.3423762259
H,-5.5037162131,2.0525163845,-0.3930117618
H,-5.8095936879,4.7250809187,-0.2973416355
H,-4.2709291305,-0.0460635584,-0.3440254651
C,-3.6400437908,4.7245647759,0.0736963321
C,-3.1063707616,-4.6967833998,-0.00571075
H,-3.3436310586,5.7572171857,0.1842806816
H,-2.8858553494,-5.7526009383,0.0410401125
C,-2.1986985748,-3.6356366552,0.1204480487
N,-4.2385009521,-2.7917411979,-0.2038647162

Cartesian Coordination of 3a^{+}-3

–1358.8135483 hartree
C,-0.1492726984,0.6146333817,-1.1681283432
C,-1.3798204379,-0.0371909011,-1.2459300441
C,0.6485990337,0.4411199292,-0.0374490579
C,1.9615891309,0.97636455,0.1271581125
C,3.9852925408,1.570172767,0.9317606391
C,-2.2464896094,-0.0158609849,-2.379929562
C,-3.502486555,-0.5987946449,-2.5191490557
N,2.6379223092,1.7067437112,-0.8336571571
N,-1.9616306326,0.6221789698,-3.5739974295
H,2.3047428971,1.9297889208,-1.7574725698
H,-1.1164892645,1.1228669892,-3.7959063112
C,-3.9704663424,-0.3054921282,-3.8080034013
C,2.7989941634,0.8868869025,1.2350678359
B,-1.1509006121,-0.7685008648,1.060578299
O,-1.8172294605,-0.7546375561,-0.2360313726
O,0.2181274861,-0.2746682309,0.9766169872
O,-1.902327708,0.115454068,1.9718633021
C,-1.6703668654,-2.5572129648,2.6394878359
C,-2.3737132737,-1.6541410891,3.4620191811
C,-1.571338941,-3.9212429134,3.012788503
C,-2.9824972914,-2.1008905712,4.6597240719
C,-2.4633898863,-0.3004808936,3.0772936494
C,-2.160956362,-4.3471054137,4.1700468523
H,-1.025387524,-4.5954202998,2.3637406575
C,-3.6841229116,-1.1824334348,5.473353803
C,-2.8829457764,-3.4611062468,5.0305989445
C,-3.167795588,0.6194691221,3.8950164486
H,-2.0833084762,-5.3928035205,4.4533170214

C,-3.7534723771,0.1825535152,5.0500119483
C,-4.2797006143,-1.6453954851,6.6553151194
C,-3.4931417905,-3.8827755587,6.2207683917
H,-3.2232704443,1.6543059511,3.5789743245
C,-4.1844617992,-2.9828468201,7.0244015417
H,-3.4221812909,-4.9259180452,6.5142109077
H,-4.6505313829,-3.3247809833,7.9412691455
O,-1.1050061727,-2.1518966283,1.5310579886
H,-4.8209035502,-0.9473473736,7.2870830033
H,-4.2922345041,0.8897171001,5.6740769955
H,-4.0033786878,-1.168972646,-1.7514610644
H,-4.9119214991,-0.6046270015,-4.243575829
H,2.5504425218,0.376163437,2.1530679411
H,4.8467046835,1.6960283414,1.5702527587
H,0.1970922551,1.2231723385,-1.9903516345
C,3.8538145336,2.063661455,-0.3552627746
C,-2.993247437,0.4493261068,-4.4345366465
H,-2.966193744,0.8691604225,-5.4294067104
H,4.5425790058,2.6394985812,-0.9559076665

Cartesian Coordination of 3a$^+$-Cl$^-$-1

–1819.2707422 hartree
C,2.6300179972,0.0005977491,0.065415411
C,1.9746064576,-1.1988874542,-0.1716624979
C,1.9732387319,1.1992514794,-0.1720968447
C,2.6291785953,2.4658369371,-0.1224595243
C,3.1614592819,4.6555545152,-0.1277131263
C,2.6319856297,-2.4647079055,-0.121585301
C,2.1049910751,-3.7473710944,-0.2620775103
N,3.9880642436,2.5970783707,0.0938803804
N,3.9910212399,-2.5943334182,0.0947909789
H,4.6626440636,1.8340014881,0.2114465028
H,4.6647350316,-1.8304531859,0.2121070848
C,3.166749521,-4.6538215328,-0.1260977705
C,2.1007297898,3.7478534044,-0.2633892749
B,-0.110354172,-0.0010677041,-0.3922151012
O,0.6942695626,-1.2148099559,-0.4939585531
O,0.6928917192,1.2135890653,-0.4944128221
O,-1.0654514962,-0.0018317005,-1.5007923985
C,-2.0742877947,-0.0008922156,1.0815963825
C,-2.9270121447,-0.0010004797,-0.0419736484
C,-2.6271840208,-0.0005003078,2.3875826201
C,-4.3327067642,-0.0007393433,0.1267467187
C,-2.3657180832,-0.0015362516,-1.3337912071
C,-3.9845844259,-0.0001910119,2.5446821234
H,-1.9533590086,-0.0004307096,3.235888418
C,-5.1748052034,-0.0009592587,-1.0084253052
C,-4.881536608,-0.0002999502,1.4294531429
C,-3.2109791213,-0.0018135208,-2.4711536796
H,-4.4058102272,0.0001416969,3.5453202863
C,-4.5677479811,-0.001507183,-2.3034742147
C,-6.5647819092,-0.0006806575,-0.8193133949
C,-6.2755890585,-0.000038627,1.5757132582
H,-2.7565331974,-0.0022385094,-3.4545745589
C,-7.1082606431,-0.0002155655,0.4607298692
H,-6.7044621527,0.0002933136,2.5730353947
O,-0.7757401132,-0.001202333,0.9300172737
H,-7.2183988983,-0.000842665,-1.6860658033
H,-5.2126170365,-0.0016891166,-3.1768992016
H,1.0636250668,-3.974954918,-0.4368930257
H,3.1155572839,-5.7315358427,-0.1770055924
H,1.059105948,3.974195312,-0.4382821282
H,3.1090443968,5.733192734,-0.1789864035
H,3.6744288201,0.001239766,0.3397370085
C,4.3121915969,3.9062451851,0.0898096614
C,4.3166315527,-3.9031327408,0.0911650274
Cl,6.0809779155,0.0026250973,0.4639463186
H,5.3387382302,-4.2197623243,0.2391157787
H,5.3339391636,4.2240802848,0.2376563817
H,-8.1842735344,-0.0000072758,0.590596756

Cartesian Coordination of 3a$^+$-Cl$^-$-2

–1819.2659634 hartree
C,3.2441675784,0.7970734886,0.0883206989
C,3.1213554119,-0.5773309708,-0.0046862424
C,2.1207190368,1.5973097417,-0.1373958024
C,2.1496730026,3.0191022557,-0.1580263635
C,2.702728138,5.2034535047,-0.1165470998
C,4.2177775143,-1.491127879,0.1146858449
C,5.5805398342,-1.2816931302,0.3068675212
N,1.0082172293,3.7704352363,-0.3393550612
N,4.0186175894,-2.851219353,0.0419249142
H,0.044673197,3.4200693775,-0.4609109734
H,3.1165408332,-3.2841494826,-0.0969093534
C,6.1968940193,-2.5440834997,0.3495921186
C,3.2214055572,3.9084414803,-0.0178871832
B,0.7126767228,-0.3896654577,-0.221732783
O,1.9509755583,-1.1512211866,-0.2347469782
O,0.9488431897,1.046680423,-0.3686370962
O,-0.1063935435,-0.8302616592,-1.3505284903
C,-1.2517741287,-0.7729479715,1.1701554578
C,-2.0353570073,-0.950006153,0.0114647983
C,-1.8655102882,-0.7835145836,2.4468044623
C,-3.4348386664,-1.1319537757,0.116275436
C,-1.4058884977,-0.9885075536,-1.2484015961
C,-3.2203596545,-0.9407855578,2.5413115038
H,-1.2432028267,-0.6497417841,3.3233899677
C,-4.2041529575,-1.335007655,-1.0518444659
C,-4.0490982524,-1.1185544343,1.3891885163
C,-2.1737914956,-1.21611704,-2.4157940048
H,-3.691222041,-0.9328381589,3.5195000279
C,-3.5284603974,-1.3715426934,-2.3115291414
C,-5.5909497948,-1.5038403742,-0.9267696119
C,-5.4381366528,-1.2913443773,1.4713912333
H,-1.6675296801,-1.2445580549,-3.3730767621
C,-6.2008677767,-1.4785357434,0.3229811416
H,-5.9176152206,-1.2798010177,2.4454064152
H,-7.2738289677,-1.6093190527,0.4027555832
O,0.0458261561,-0.6134204143,1.0750818244
H,-6.189792853,-1.6576955522,-1.8190188192
H,-4.1171745011,-1.5290460855,-3.2100584183
H,6.0666680804,-0.3215198901,0.4022161846
H,7.2485891332,-2.7488658128,0.4856871598
H,4.256174785,3.6362837446,0.1334410023
H,3.2508464707,6.1320882647,-0.0546835692
H,4.2056866837,1.2458523354,0.2858215147
C,1.3288056365,5.0755448167,-0.3141055444
C,5.1997321008,-3.4940472003,0.1815451859
H,0.5682504889,5.8331792705,-0.4353141161
H,5.2558484706,-4.5719650428,0.1532292526

Cl,-2.1148952146,3.3039136585,-0.7072662892

Cartesian Coordination of 3a$^+$-PF$_6^-$

–2299.5626248hartree

H,4.2364241438,2.0986446684,-11.2152356373
C,6.2402898514,3.5373664539,-12.3512435345
H,5.8544087821,2.7566255336,-12.9994011776
C,7.2791094417,4.3529134694,-12.7882816847
H,7.6989432349,4.2091313075,-13.7770947841
C,7.7849384815,5.3481386587,-11.9586298753
H,8.6009336364,5.9769384195,-12.3012552545
F,2.7972542663,8.8184860004,-12.5583559603
F,4.042086028,7.0066374815,-11.8184624353
F,4.1106535495,9.0051096301,-10.6637463361
C,-0.4032300058,8.9992095127,-6.4386015059
H,-1.2841097136,9.5909884638,-6.2378140173
C,0.2995168554,8.1967475311,-5.530489117
H,0.0697524429,8.0479895837,-4.4851444097
C,1.3673035659,7.6228898747,-6.2242899979
C,2.3983237243,6.7364742195,-5.8011296367
C,2.4995446208,6.2055922369,-4.5156271503
H,1.7623772929,6.4462165363,-3.7648251197
C,3.5785930191,5.3947528884,-4.2059897015
C,3.8180806917,4.8405022869,-2.9084942472
C,3.1190632527,4.9541390117,-1.7092591073
H,2.2135553876,5.5228307433,-1.5542588321
C,3.8091432717,4.1965227104,-0.7483508562
H,3.5417576574,4.0657407615,0.2898419424
C,4.9120999268,3.6387128593,-1.3794725084
H,5.6893884583,2.995788562,-0.9942287601
N,1.3051698024,8.0751795374,-7.5246182479
H,1.9483608686,7.8073316182,-8.2656645722
N,4.9066337065,4.0311365605,-2.6726139572
H,5.5889182597,3.7812709244,-3.374533557
O,3.2923089208,6.4425632879,-6.719602656
O,4.4888655619,5.0813851315,-5.1128371489
B,4.3715292183,5.4764137909,-6.5068485853
C,4.584593383,4.100254115,-8.5013495657
C,4.0953999458,3.0594825035,-9.3271019047
H,3.2951655221,2.4319599773,-8.9536221641
C,4.6250431082,2.8856668469,-10.5761831259
C,5.6815390795,3.7088736565,-11.0765075579
C,7.253620217,5.5518172471,-10.676688087
C,7.7500874575,6.5495497042,-9.7815251445
H,8.5698929366,7.1795524624,-10.1127966067
C,7.2261481828,6.7315729671,-8.5310650529
H,7.6063532552,7.4898490989,-7.8572244837
C,6.1416086064,5.9275770631,-8.106311208
C,5.6294189829,4.9289274721,-8.9581149986
C,6.1886179488,4.7307372477,-10.2424325882
O,4.0712818736,4.2774211465,-7.3063451065
O,5.6289858704,6.1103426409,-6.9106489796
P,2.9280972658,7.9956420592,-11.1591508131
F,1.8090568863,8.9733633465,-10.4668983559
F,1.7409914025,6.9812858207,-11.6326213118
F,3.0482869797,7.1737785416,-9.7328622372
C,0.2484794953,8.8978422782,-7.664511734
H,0.0283433988,9.3551335486,-8.617480545

Cartesian Coordination of 3b$^+$-1

–1820.7035917 hartree

C,-2.5051051965,0.2899941648,0.0820265537
C,-1.8584087154,0.0946226785,-1.1322702093
C,-1.8040062538,0.1009387791,1.2669413433
C,-2.3866159219,0.1681463179,2.5609955795
C,-3.7097556574,0.3400614682,4.3751389657
C,-2.4980628939,0.1611443305,-2.3993139316
C,-3.8201784564,0.4229363421,-2.7619845606
N,-1.6308931559,-0.0340885928,3.6969227789
N,-1.7942868509,-0.0444556092,-3.5673571872
H,-0.6571390112,-0.2982454341,3.67763669
H,-0.7985838392,-0.2051553314,-3.5970622089
C,-3.8906578476,0.379837848,-4.155605882
C,-3.695797522,0.408689501,2.980912527
B,0.2685019427,-0.1361407947,0.0208842624
O,-0.5656266596,-0.1983200752,-1.170572321
O,-0.5100667429,-0.1885683609,1.2484620665
O,1.1713827221,-1.2909305715,0.00529963
C,2.3065691175,1.2341278819,-0.0655111688
C,3.1021152323,0.0692841599,-0.0974678704
C,2.9256612328,2.510887041,-0.1013608534
C,4.5134096423,0.1660036495,-0.1642370271
C,2.4742012525,-1.1919275711,-0.0567990451
C,4.2874384473,2.5977089383,-0.1681490087
H,2.2943657493,3.3911244422,-0.0758380622
C,5.2956162605,-1.0109250344,-0.19136744
C,5.1271341569,1.4388685739,-0.2017006448
C,3.2611456796,-2.371861442,-0.0819451237
H,4.7576548472,3.5764456111,-0.1968700206
C,4.622627108,-2.2728042781,-0.1477590378
C,6.6915727234,-0.892780564,-0.2580519778
C,6.5253776391,1.5125658843,-0.2683038384
H,2.7554037851,-3.3295176254,-0.048348856
C,7.2992883654,0.3570530285,-0.2965160225
H,7.0059668644,2.4859578328,-0.2977638638
H,8.3793895261,0.4314498543,-0.3480739534
O,1.0055848696,1.1489762715,-0.0016467522
C,-2.14358055,-0.0866344132,-6.0170825016
C,-1.0664294874,-0.9296528426,-6.3147439478
C,-2.7791483666,0.5949515723,-7.061008075
C,-0.6305898056,-1.0801207621,-7.6254503322
H,-0.5866068715,-1.5023816813,-5.5263903168
C,-2.3448947488,0.4382091701,-8.3707359372
H,-3.6047400499,1.2639277112,-6.8422228732
C,-1.2679704872,-0.3967002109,-8.6569286554
H,0.1995799189,-1.7436465848,-7.8437304679
H,-2.8444665375,0.975095353,-9.1701427294
H,-0.9298980533,-0.5177848316,-9.6805626084
C,-1.8790950096,-0.1102914696,6.1582828509
C,-0.5468790181,0.1971565644,6.4585378703
C,-2.7116897986,-0.5829913409,7.1789061316
C,-0.0576746642,0.0277261634,7.747877282
H,0.105659016,0.6053775491,5.6922090012
C,-2.2215323408,-0.7459498106,8.4679411708
H,-3.7415114246,-0.8415135157,6.956266529
C,-0.8930898551,-0.4443863904,8.7560527646
H,0.9747290242,0.2773449706,7.969076679
H,-2.8768532614,-1.116979927,9.2487982457
H,-0.5116077604,-0.5732353906,9.7633444693
H,7.300915054,-1.7914229644,-0.2793162505

H,5.2218524113,-3.1785333214,-0.1676622945
H,-4.6393634055,0.6114193192,-2.0832835836
H,-4.7742641369,0.5119355269,-4.7618618198
H,-4.5393167232,0.6211290888,2.3401492535
H,-4.5587329849,0.5059264607,5.0212517296
H,-3.5574334016,0.5287992794,0.1052590865
C,-2.4089547547,0.0588028674,4.8033789872
C,-2.6129410465,0.0850725261,-4.6402913977

Cartesian Coordination of 3b$^+$-2

–1820.7000903 hartree

C,0.1401724097,-0.0386466571,1.129655628
C,-1.1418505786,-0.5730569467,1.0297908947
C,0.8608144039,0.2373777598,-0.0287380984
C,2.2068225797,0.6919390047,-0.0460665886
C,4.2999269582,1.4024740719,0.3856475079
C,-1.9384334409,-0.9880690011,2.133064811
C,-3.2437495393,-1.4749206734,2.133295547
N,2.8530551635,0.9562897303,-1.2353567508
N,-1.5181931811,-0.9656639548,3.4516343451
H,2.4059810477,0.8901783769,-2.1378898248
H,-0.5830776709,-0.7643601964,3.7692076648
C,-3.5955650262,-1.7520336471,3.4568794057
C,3.1144797862,0.9654325482,0.9786740956
B,-1.0998313431,-0.230597909,-1.3759290804
O,-1.6979035069,-0.749241702,-0.1550833147
O,0.3147437665,0.0665885666,-1.221843588
O,-1.2376780398,-1.2496483324,-2.4201204066
C,-2.6900473683,1.0918442393,-2.6836527795
C,-2.9144985453,-0.0114360241,-3.5340940507
C,-3.4321793519,2.2870145421,-2.8699390421
C,-3.87479082,0.0667405938,-4.5717997264
C,-2.1373390178,-1.1758411661,-3.3671333641
C,-4.3690518833,2.35058102,-3.8623238413
H,-3.2397766923,3.1222933464,-2.2072025289
C,-4.0668372096,-1.0374696952,-5.4329852454
C,-4.6250702487,1.2523628473,-4.7438667557
C,-2.3194738897,-2.2762308532,-4.24331253
H,-4.9424575743,3.2634300166,-3.9955904004
C,-3.2595729026,-2.2016910315,-5.2323528517
C,-5.0266087192,-0.9411274708,-6.4509295613
C,-5.5741423785,1.3072762033,-5.7740827344
H,-1.7084454,-3.158795009,-4.0961964384
C,-5.7746427052,0.219079639,-6.6168607459
H,-6.1557227658,2.2136880557,-5.9137699554
H,-6.5126602024,0.2776606985,-7.4085149501
O,-1.7952957839,1.026805451,-1.7342307405
C,-2.3380604101,-1.5329073508,5.7156902143
C,-1.5093429994,-0.6459987912,6.4129762752
C,-3.0180741904,-2.5274517005,6.4274396288
C,-1.3570772094,-0.7581960962,7.7895969118
H,-1.0089857292,0.162339401,5.8873716524
C,-2.8688446576,-2.6331469095,7.8040470151
H,-3.6497786615,-3.231478278,5.8961136297
C,-2.0358199229,-1.7520218922,8.4886164731
H,-0.7181134483,-0.0595567483,8.319321497
H,-3.3981380997,-3.4113549162,8.3435752973
H,-1.9192663036,-1.8372183394,9.56373779
H,-5.1818777677,-1.7846913708,-7.1169678403
H,-3.40388941,-3.0503059752,-5.8947969196
H,-3.8570839505,-1.5904737842,1.2524059881
H,-4.5486875217,-2.1139686866,3.8123753131
H,2.9337538701,0.8458547903,2.0371125602
H,5.2149742278,1.6708203917,0.8920032908
H,0.6019100925,0.1299334115,2.0909538903
C,4.1155780861,1.3888760758,-1.0006317821
C,-2.5029903928,-1.4324845218,4.2635469243
C,6.0453808708,2.6913087086,-1.8528551199
C,6.9322986686,3.0314995139,-2.8658216776
C,6.82936958,2.4315184678,-4.1180907417
C,5.8372067006,1.483488571,-4.3507119845
C,4.9518408922,1.1370424567,-3.3375449017
C,5.0435646303,1.7405504574,-2.0778760427
H,6.1170219472,3.1787160851,-0.8862499334
H,7.7020374224,3.7728479181,-2.6793935409
H,7.5226450494,2.6988428509,-4.908456479

Cartesian Coordination of 3b$^+$-3

–1820.6951092 hartree

C,-0.1465571383,0.0808284594,-1.2567629593
C,-1.5119028443,-0.1840120521,-1.1478443009
C,0.6115747786,0.2750464911,-0.1019083424
C,1.9858132861,0.6439734509,-0.0804886355
C,4.0883344021,1.1652006355,0.5445322766
C,-2.4093976276,-0.3063656274,-2.2453981596
C,-3.7618523495,-0.639368463,-2.2281110605
N,2.7414100077,0.9258450094,-1.2058752757
N,-2.0605853608,-0.1123585008,-3.5711368085
H,2.3894610669,1.0222363646,-2.1450550907
H,-1.1815094131,0.2505703003,-3.9038804919
C,-4.220448857,-0.6318632387,-3.5481156215
C,2.8296187452,0.7862281483,1.0186451641
B,-1.2804637188,-0.4023088375,1.2573571804
O,-2.0693918679,-0.3221274752,0.0381039767
O,0.0590477977,0.1379994512,1.0864731008
O,-1.958741838,0.3869344931,2.2879885772
C,-1.6048833117,-2.3185624738,2.7572141609
C,-2.2479185182,-1.4874655878,3.697999756
C,-1.4423466877,-3.7011255145,3.0342406614
C,-2.7319811854,-2.023170522,4.9161495677
C,-2.4024953955,-0.1163264388,3.4098031328
C,-1.9113594961,-4.2122934224,4.2110246592
H,-0.9453159481,-4.3199115455,2.2967115438
C,-3.3739403016,-1.175980778,5.8477584311
C,-2.5688083271,-3.4003700714,5.189488919
C,-3.0471482455,0.7323350211,4.3462591527
H,-1.7852220519,-5.2707871378,4.4196416666
C,-3.5123162875,0.2102012504,5.5199795429
C,-3.8458112402,-1.7265777558,7.0478389795
C,-3.0552250166,-3.9106207817,6.4011510831
H,-3.1535757128,1.7824043299,4.1015392763
C,-3.687498516,-3.0807903375,7.3208825046
H,-2.9345477157,-4.9674634425,6.6198954434
H,-4.0580469667,-3.4909884637,8.2532249625
O,-1.1570619139,-1.8285702764,1.6328556061
C,-3.0784288597,-0.1430952844,-5.8256622089
C,-1.9044869383,-0.4349011682,-6.5301416609
C,-4.2024518564,0.2970744513,-6.5333422426
C,-1.8536767228,-0.2800911181,-7.9101600518
H,-1.0361444875,-0.82435139,-6.0062501117

C,-4.1505315034,0.4448940121,-7.9132251971
H,-5.112866219,0.5421217131,-5.9965753968
C,-2.9760518594,0.1606900211,-8.6051430464
H,-0.9407641798,-0.5183071943,-8.4457762078
H,-5.0275940262,0.790911566,-8.4497472083
H,-2.9372654579,0.2779252012,-9.6828580237
H,-4.3403998515,-1.0834606331,7.7697094736
H,-4.0053612412,0.8628097733,6.2346772947
H,-4.3248796528,-0.8750425596,-1.3376093892
H,-5.2147366731,-0.8765677646,-3.8906881388
H,2.5385832315,0.6095499126,2.0431539926
H,4.9800633131,1.3285407801,1.1310032342
H,0.3171818487,0.1524669723,-2.2297096745
C,4.0107633067,1.2528255046,-0.8452329599
C,-3.1453510825,-0.2946693688,-4.370064955
C,6.0476161057,2.5136674007,-1.4846603296
C,7.0236783335,2.8612712351,-2.4093787239
C,7.0004405384,2.3178254679,-3.691199278
C,5.998819385,1.4175468178,-4.0423191899
C,5.0243847824,1.0630816853,-3.117204602
C,5.0351664685,1.6113372699,-1.8292375142
H,6.0570385734,2.9577980766,-0.4948717609
H,7.8008435243,3.5651320821,-2.1311378263
H,7.7631360934,2.5915509218,-4.4123710542
H,5.9848657927,0.9787039745,-5.0344196096
H,4.2762760738,0.323913421,-3.3894559821

Cartesian Coordination of 3b$^+$-Cl$^-$-1

–2281.1503523 hartree
C,0.8383862944,-0.0003565681,-0.1336715042
C,0.1673323412,-1.2004103381,-0.3247567216
C,0.1662469321,1.1990614858,-0.3249537702
C,0.7987027262,2.4759701838,-0.2909607313
C,1.244445298,4.6792531617,-0.308622705
C,0.8009573021,-2.4767347015,-0.2905812934
C,0.2228737341,-3.7390916927,-0.4106263572
N,2.1581481788,2.6582584699,-0.1192528121
N,2.1605873446,-2.6577320028,-0.1189746605
H,2.842002162,1.9039058343,-0.0217575382
H,2.8437618677,-1.9027241274,-0.0218064352
C,1.2487374953,-4.6796083005,-0.3078541497
C,0.2194631175,3.7377688414,-0.4113123309
B,-1.9276350761,-0.0016534418,-0.4529785836
O,-1.1302411921,-1.213494545,-0.5835231144
O,-1.1313291513,1.2109131098,-0.5837494053
O,-2.9258612549,-0.0022081734,-1.5256217315
C,-3.8388700784,-0.0013989263,1.0923062026
C,-4.7323101941,-0.0013307172,0.0007057019
C,-4.3442764555,-0.0010695075,2.4178780708
C,-6.1310159995,-0.0009418538,0.220726023
C,-4.2185219293,-0.0018065832,-1.3107765828
C,-5.6948226384,-0.000638951,2.6244854353
H,-3.6398569615,-0.001140417,3.2409751059
C,-7.0140914803,-0.0009742244,-0.8828773909
C,-6.6320133555,-0.0005582184,1.5426041371
C,-5.1053438265,-0.0018973818,-2.4165534149
H,-6.079248972,-0.0003527388,3.6398817932
C,-6.4548788373,-0.0014684169,-2.1994184681
C,-8.3961824574,-0.0005658217,-0.6432732929
C,-8.0197210383,-0.0001615692,1.739604919
H,-4.6869870412,-0.0022787618,-3.4158787068
C,-8.8925344933,-0.0001539146,0.6557412868
H,-8.4120079948,0.0001324072,2.7518197679
H,-9.9630860117,0.0001572092,0.8249342003
O,-2.5472911532,-0.001806223,0.8937245677
C,3.8099567023,-4.5176997107,0.0288575184
C,4.9205245183,-3.6782421414,0.1853228658
C,4.0142676638,-5.9047955606,0.0228970001
C,6.195579973,-4.2147891752,0.3307941367
H,4.8057061978,-2.5989111997,0.1945960738
C,5.2890216018,-6.4364561527,0.1687746974
H,3.1721039261,-6.5770760845,-0.095466514
C,6.3876332144,-5.5934628617,0.3235121241
H,7.0428586413,-3.5472327286,0.4503265561
H,5.4242242523,-7.5131685604,0.1615399624
H,7.3837993661,-6.0083854079,0.4372940503
H,-9.0807229301,-0.0005827822,-1.4858445402
H,-7.1317030098,-0.0015051268,-3.0483319731
H,-0.830835456,-3.9288518956,-0.5517336457
H,1.138831253,-5.7521998216,-0.3574960195
H,-0.834400921,3.9265213444,-0.5526132638
H,1.133580021,5.7517289282,-0.3586077193
H,1.8964529032,0.0001392384,0.0851089754
C,2.447069301,3.9852928731,-0.128093894
C,2.4507413089,-3.9844999767,-0.127605635
C,4.0085370233,5.9071262865,0.024039889
C,5.2827772988,6.4400200272,0.1699064506
C,6.3823984492,5.5980536384,0.3230588911
C,6.1918722504,4.219160547,0.3287786395
C,4.9173402491,3.681377314,0.1832779276
C,3.8057683424,4.519797621,0.0283930541
H,3.1655520116,6.5786178109,-0.0929602959
H,5.4167817996,7.5168901178,0.163944858
H,7.3781628829,6.0139374199,0.4368473669
H,7.039950297,3.5523995671,0.4470751805
H,4.8037167509,2.601909076,0.1913007186
Cl,4.2678255656,0.0012906818,0.1771444733

Cartesian Coordination of 3b$^+$-Cl$^-$-2

–2281.143491 hartree
C,-0.8689895202,-2.5359464381,0.1976850963
C,-1.9062644842,-1.6364698784,0.0321510833
C,0.4448170633,-2.1028769853,-0.0089579408
C,1.57318227,-2.966251957,0.028790458
C,2.9635811382,-4.7283672794,0.1206209835
C,-3.2918923593,-1.9829169245,0.122402502
C,-3.9324500384,-3.1966017686,0.3568610365
N,2.8706008516,-2.5126776304,-0.1102006948
N,-4.2704520923,-1.0269968499,-0.0260461317
H,3.1255157786,-1.5207162651,-0.2196186785
H,-4.0711113063,-0.04536434,-0.157790257
C,-5.3113767817,-2.9484701744,0.3544734414
C,1.6240665706,-4.3552712609,0.1752025959
B,-0.3297099486,0.1965331712,-0.1940012774
O,-1.6754493098,-0.3623192726,-0.246850043
O,0.6932166755,-0.8412166585,-0.2800757091
O,-0.168101052,1.0916701699,-1.3389970255
C,0.3119385706,2.1070853444,1.1925616254
C,0.5882484243,2.8609553505,0.0347387054
C,0.5495841213,2.6661515989,2.4732326819

C,1.1126201598,4.1705742633,0.1426062794
C,0.3241957154,2.3026784967,-1.2305178898
C,1.0641816192,3.9283493186,2.5723400989
H,0.3268086092,2.0694241065,3.3495498328
C,1.3786922681,4.9172344129,-1.0276882445
C,1.3642343433,4.721535076,1.4199127346
C,0.5758090581,3.0572800999,-2.4027046113
H,1.2556413904,4.3505186221,3.5541142412
C,1.0921645473,4.3183329617,-2.2944992791
C,1.905076258,6.2107169725,-0.8996214783
C,1.8900173145,6.0183363371,1.5051255783
H,0.3628139421,2.6075739144,-3.3649884671
C,2.1591777094,6.7540996837,0.3552538142
H,2.0872442555,6.4482470442,2.4823175115
H,2.5670576669,7.754894023,0.4379056608
O,-0.1746650983,0.8952371195,1.0964476852
H,2.1139200822,6.7894604127,-1.7940756523
H,1.2951819346,4.8909536961,-3.1942991992
H,-3.44986508,-4.1524035377,0.5012124154
H,-6.0970182947,-3.6780932218,0.4843171446
H,0.7758818148,-5.0122462282,0.3020363915
H,3.3546905429,-5.7299554708,0.2162683775
H,-1.075967629,-3.5678637882,0.436397254
C,3.7258149983,-3.5643533932,-0.0564769403
C,-5.50006056,-1.5866686298,0.1136464305
Cl,3.8009961321,0.6864230522,-0.2677885024
C,-6.7412078234,-0.8140798621,0.0005234663
C,-7.9022912256,-1.2609135245,0.643841595
C,-6.797650431,0.3682862855,-0.7477543982
C,-9.0877077564,-0.5431922792,0.5411603314
H,-7.8717120465,-2.1667451614,1.2400327676
C,-7.9831560428,1.0890447741,-0.8419272017
H,-5.9212018334,0.7206320846,-1.2824259491
C,-9.1325122849,0.6364275029,-0.1991552603
H,-9.9773954788,-0.901983939,1.0483405609
H,-8.0099196901,2.0017327148,-1.4281854474
H,-10.0576635594,1.1980721957,-0.2758280549
C,5.1864816536,-3.4530416821,-0.1595763723
C,5.8523021574,-2.2352377799,0.0236127901
C,5.9442509012,-4.6001690241,-0.4362632109
C,7.2391750145,-2.1719208425,-0.0658024255
H,5.2927964959,-1.3275922034,0.2256263232
C,7.3290284324,-4.5327623207,-0.5206350556
H,5.4485713479,-5.5506020614,-0.6000050802
C,7.9840486219,-3.3169585173,-0.3341719963
H,7.7382535884,-1.2189064805,0.0781593938
H,7.8973012369,-5.4313143419,-0.7383085898
H,9.0658414508,-3.2631398329,-0.401362824

Cartesian Coordination of 3b$^+$-PF$_6^-$

–2761.4417892 hartree
H,-0.0784025254,4.7270690758,3.2006477275
C,0.1118917696,6.1712330809,0.907629276
H,0.2200104159,6.7749437321,1.8033871386
C,0.1881528249,6.7676434192,-0.346248197
H,0.3554798412,7.8354691718,-0.4271803338
C,0.0526558884,5.9980568613,-1.4978700221
H,0.1150459077,6.4680705436,-2.4745318286
F,4.3383898534,2.8034161613,-0.7432344488
F,2.8671000157,2.3890847674,0.9951770904
F,2.4545976293,1.5043510987,-1.0958523343
C,2.7079215821,-4.8940898215,-0.299356466
H,3.1690611945,-5.864176706,-0.4094295117
C,1.3427593827,-4.6120823772,-0.3476125749
H,0.5439620457,-5.3203212002,-0.5134320849
C,1.192407864,-3.2422794105,-0.1213319746
C,0.0218182094,-2.4361819612,-0.0574676722
C,-1.2690486869,-2.9250284867,-0.2577336376
H,-1.4297516517,-3.9614072046,-0.5126181442
C,-2.3473602875,-2.0806167385,-0.0480120449
C,-3.713598631,-2.4939609889,-0.1177572225
C,-4.2966526277,-3.7336012558,-0.3699763266
H,-3.768566766,-4.6589538015,-0.5495791194
C,-5.6851342453,-3.5575603089,-0.3312288599
H,-6.4350269139,-4.3234500767,-0.4630668234
C,-5.9379962978,-2.2129567032,-0.0510447161
N,2.4531020704,-2.7146456404,0.0646632132
H,2.6377675562,-1.7241897587,0.2047437743
N,-4.7366186961,-1.5939862306,0.0767377837
H,-4.5853108534,-0.606829854,0.2294679731
O,0.2218368409,-1.1674069261,0.237377617
O,-2.1690643018,-0.8073843066,0.2618473968
B,-0.8504271919,-0.1778197445,0.18512758
C,-0.5181444583,2.0033226941,1.2302341788
C,-0.3865640168,2.7830454116,2.4050114959
H,-0.4429245399,2.2871162321,3.3665470071
C,-0.1838994166,4.1307938979,2.2994308795
C,-0.1017308524,4.7906291658,1.0334112991
C,-0.1622777657,4.6155053317,-1.4149245468
C,-0.3057654421,3.7818630486,-2.5693556429
H,-0.2479456011,4.2439421219,-3.5501290014
C,-0.5080700881,2.4341684295,-2.47291702
H,-0.6093367178,1.8068248563,-3.3503080118
C,-0.576799077,1.8283317192,-1.1935924018
C,-0.4513777967,2.618096665,-0.03349249
C,-0.2393980732,4.0129992486,-0.1385378946
O,-0.7056692363,0.708003737,1.3369126808
O,-0.7620348909,0.535593272,-1.1012624158
P,3.7103449962,1.465947875,-0.0531530668
F,4.5412704363,0.5237175294,-1.093858554
F,4.9582074479,1.4085736142,0.996788488
F,3.0744345266,0.1132799047,0.6503574245
C,3.3833278198,-3.6949770657,-0.040159111
C,-7.2146395051,-1.5094229742,0.1074952264
C,-7.3136101825,-0.3437563882,0.8772777785
C,-8.3668950121,-2.0073830614,-0.5137278041
C,-8.5327023448,0.3109421397,1.0142328314
H,-6.4434519423,0.0468799386,1.3953796433
C,-9.5854357689,-1.3555464654,-0.3686143949
H,-8.3043523342,-2.9007072965,-1.1260234939
C,-9.672987669,-0.192163205,0.3932037248
H,-8.5922972959,1.211409263,1.6166952361
H,-10.4679532611,-1.7531023678,-0.859084158
H,-10.6242218261,0.317965662,0.5032510866
C,4.8233937037,-3.4668563517,0.1217406589
C,5.3174808084,-2.3636978213,0.8282037037
C,5.7335619337,-4.3801890949,-0.4269219605
C,6.687539344,-2.1793157256,0.9775361829
H,4.6371433837,-1.6428349789,1.2670976169
C,7.1016098249,-4.1960517601,-0.2706494197

H,5.3692796486,-5.2311863635,-0.9923377818
C,7.5844951602,-3.0939684204,0.4321760693
H,7.0531730984,-1.3176486731,1.5263337787
H,7.79237504,-4.911219348,-0.7052774504
H,8.6530707266,-2.948151806,0.5517754015

[S10] M. J. Frisch, G. W. Trucks, H. B. Schlegel, G. E. Scuseria, M. A. Robb, J. R. Cheeseman, G. Scalmani, V. Barone, G. A. Petersson, H. Nakatsuji, X. Li, M. Caricato, A. V. Marenich, J. Bloino, B. G. Janesko, R. Gomperts, B. Mennucci, H. P. Hratchian, J. V. Ortiz, A. F. Izmaylov, J. L. Sonnenberg, D. Williams-Young, F. Ding, F. Lipparini, F. Egidi, J. Goings, B. Peng, A. Petrone, T. Henderson, D. Ranasinghe, V. G. Zakrzewski, J. Gao, N. Rega, G. Zheng, W. Liang, M. Hada, M. Ehara, K. Toyota, R. Fukuda, J. Hasegawa, M. Ishida, T. Nakajima, Y. Honda, O. Kitao, H. Nakai, T. Vreven, K. Throssell, J. A. Montgomery, Jr., J. E. Peralta, F. Ogliaro, M. J. Bearpark, J. J. Heyd, E. N. Brothers, K. N. Kudin, V. N. Staroverov, T. A. Keith, R. Kobayashi, J. Normand, K. Raghavachari, A. P. Rendell, J. C. Burant, S. S. Iyengar, J. Tomasi, M. Cossi, J. M. Millam, M. Klene, C. Adamo, R. Cammi, J. W. Ochterski, R. L. Martin, K. Morokuma, O. Farkas, J. B. Foresman and D. J. Fox, *Gaussian 16*, Revision C.01, Gaussian, Inc., Wallingford CT, 2016.

[S11] Articles for *GAMESS*: (a) M. W. Schmidt, K. K. Baldridge, J. A. Boatz, S. T. Elbert, M. S. Gordon, J. H. Jensen, S. Koseki, N. Matsunaga, K. A. Nguyen, S. Su, T. L. Windus, M. Dupuis and J. A. Montgomery, *J. Comput. Chem.*, 1993, **14**, 1347−1363; (b) M. S. Gordon and M. W. Schmidt, In *Theory and Applications of Computational Chemistry: The First Forty Years*, ed: C. E. Dykstra, G. Frenking, K. S. Kim and G. E. Scuseria, Elsevier, 2005; pp. 1167–1189.

[S12] Report for FMO: K. Kitaura, E. Ikeo, T. Asada, T. Nakano and M. Uebayasi, *Chem. Phys. Lett.*, 1999, **313**, 701–706.

[S13] Report for pair interaction energy decomposition analysis (PIEDA): D. G. Fedorov and K. Kitaura, *J. Comput. Chem.*, 2007, **28**, 222–237.

4. Solution-state properties

Electrochemical analysis. Cyclic voltammograms (CVs) were measured under Ar atmosphere in CH_2Cl_2 solutions containing the sample (0.25 mM) and TBACl or $TBAPF_6$ (0.1 M) as supporting electrolytes using an ALS/CH Instruments 720 electrochemical analyzer with a glassy-carbon disk working electrode (3-mm diameter), an $Ag/AgNO_3$ (0.010 M) reference electrode, and a Pt counter electrode. Spectroelectrochemical analyses were conducted using an ALS/CH Instruments 720 electrochemical analyzer and a JASCO V-670 spectrometer with a thin layer quartz glass cell (path length: 1.0 mm), a Pt mesh working electrode, an $Ag/AgNO_3$ (0.010 M) reference electrode, and a Pt counter electrode. The sample solutions of **3a**$^+$-Cl^- and **3b**$^+$-Cl^- (0.15 mM) contained TBACl (0.1 M) as a supporting electrolyte, and $TBAPF_6$ was used for the solutions of **3a**$^+$-PF_6^- and **3b**$^+$-PF_6^- (0.15 mM). The supporting electrolyte was dried in vacuo at 100 °C for 1 day, and CH_2Cl_2 was distilled from CaH_2 prior to use.

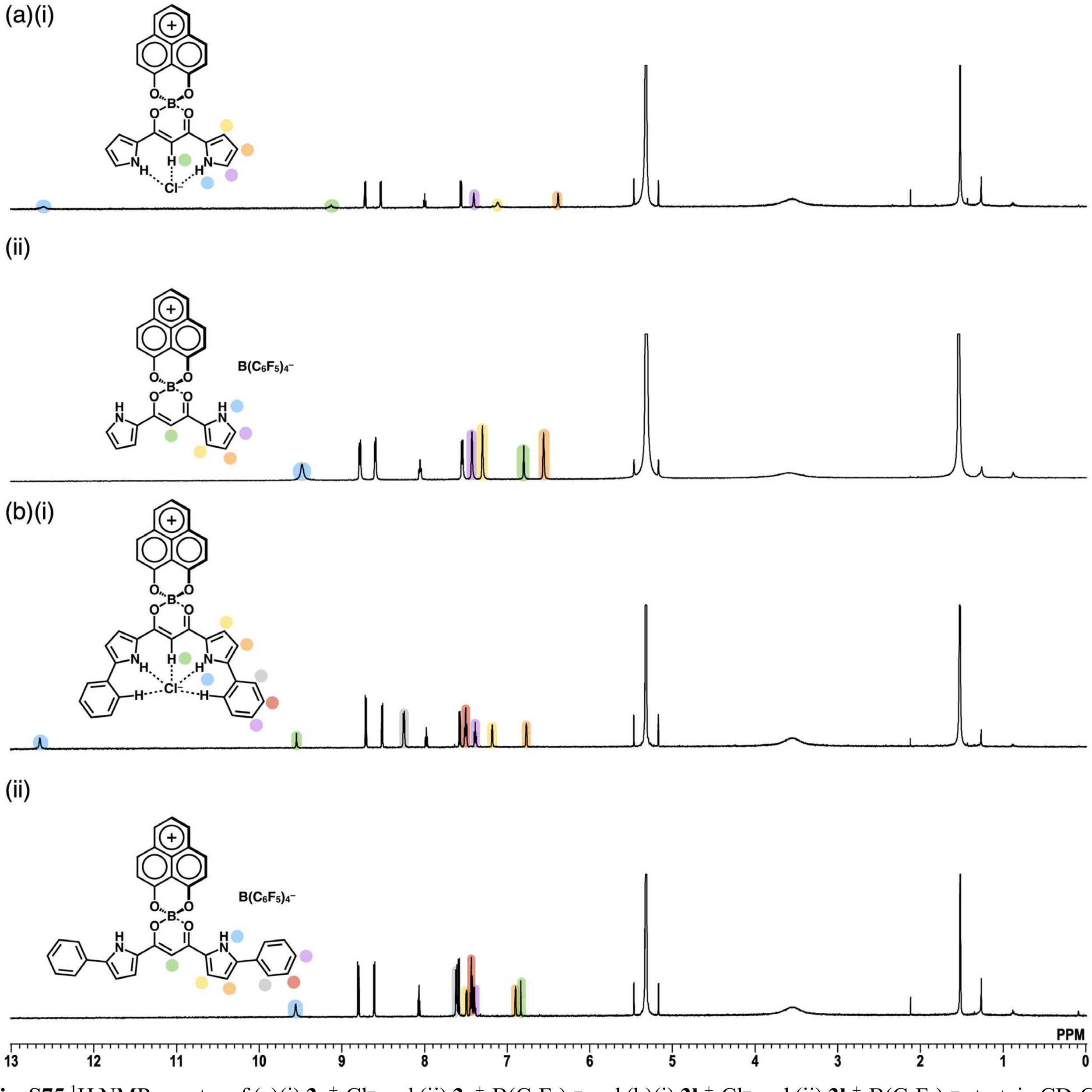

Fig. S75 ^{1}H NMR spectra of (a)(i) **3a**$^+$-Cl^- and (ii) **3a**$^+$-$B(C_6F_5)_4^-$ and (b)(i) **3b**$^+$-Cl^- and (ii) **3b**$^+$-$B(C_6F_5)_4^-$ at r.t. in CD_2Cl_2 (0.25 mM).

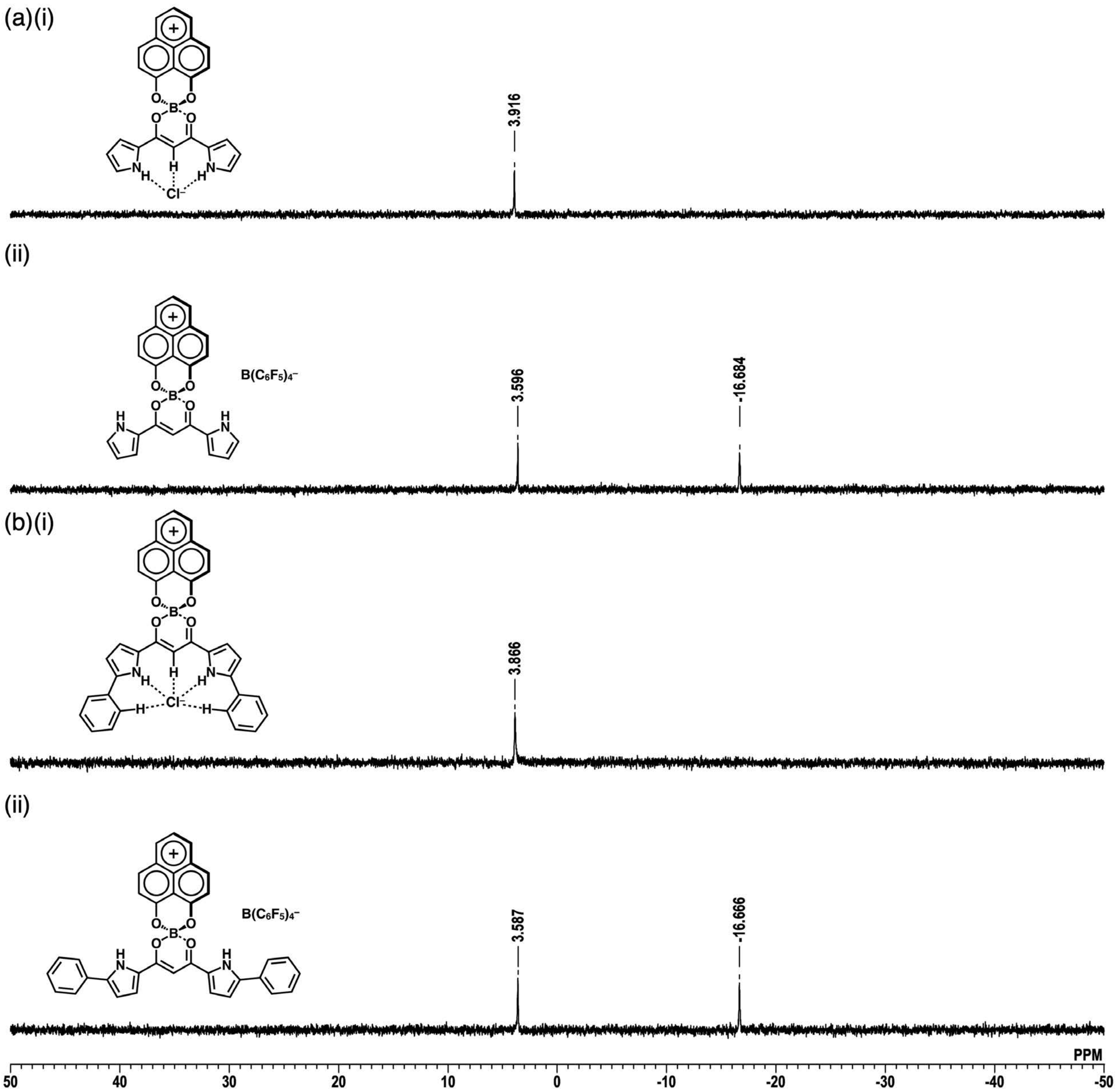

Fig. S76 ^{11}B NMR spectra of (a)(i) **3a**$^{+}$-Cl^{-} and (ii) **3a**$^{+}$-B(C_6F_5)$_4^{-}$ and (b)(i) **3b**$^{+}$-Cl^{-} and (ii) **3b**$^{+}$-B(C_6F_5)$_4^{-}$ at r.t. in CD_2Cl_2 (0.25 mM). These signals are intermediate values between those of boron catecholate and BF_2 complexes (~8.4 and ~0.4 ppm, respectively) in $CDCl_3$.[S1,2b]

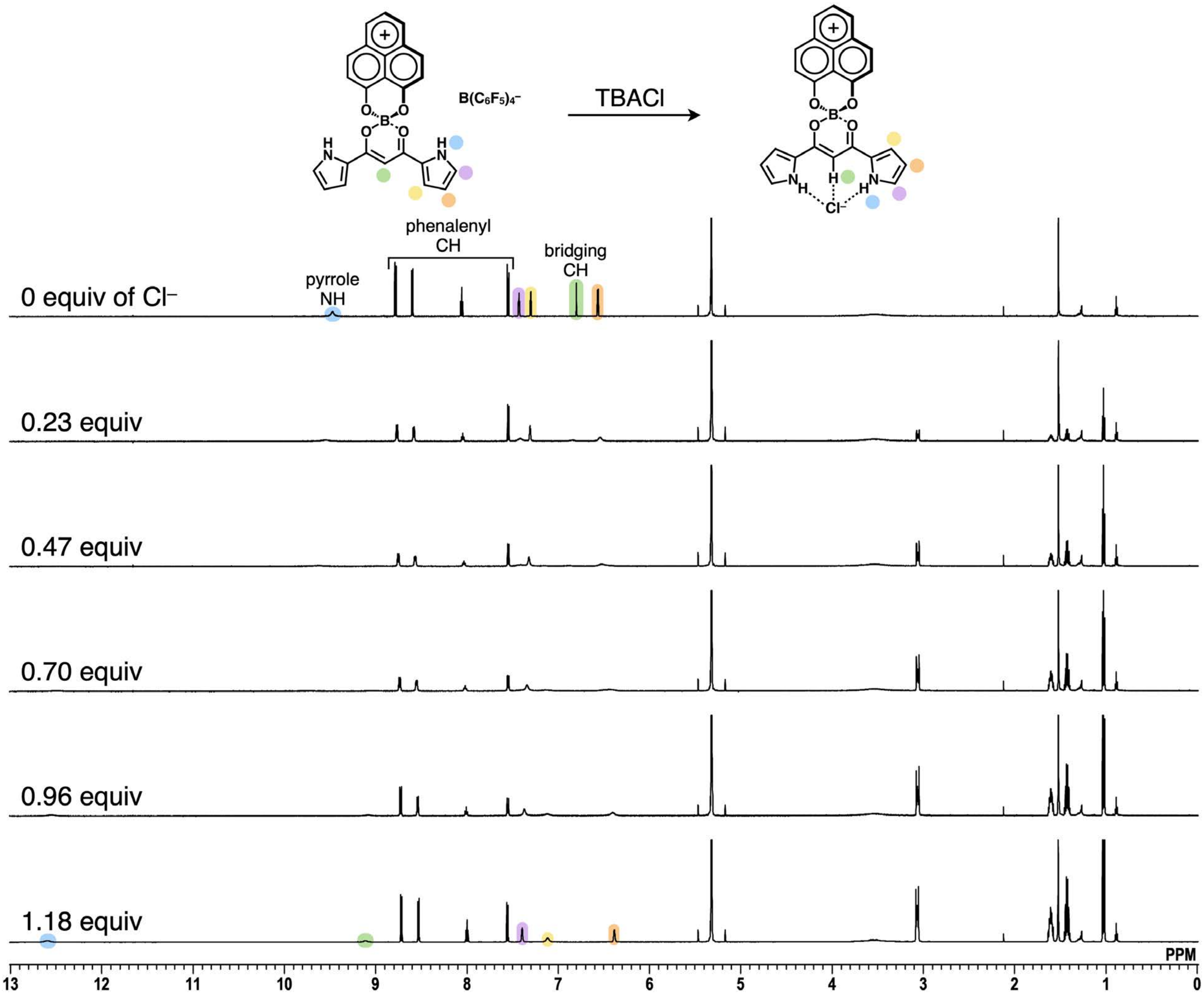

Fig. S77 ^{1}H NMR spectral change of **3a**$^{+}$-B(C_6F_5)$_4^{-}$ upon the addition of Cl^{-} as a TBA salt at r.t. in CD_2Cl_2 (0.5 mM). Upon the addition of Cl^{-} as a TBA salt, the pyrrole NH and bridging CH signals disappeared, and signals corresponding to the Cl^{-} complex appeared.

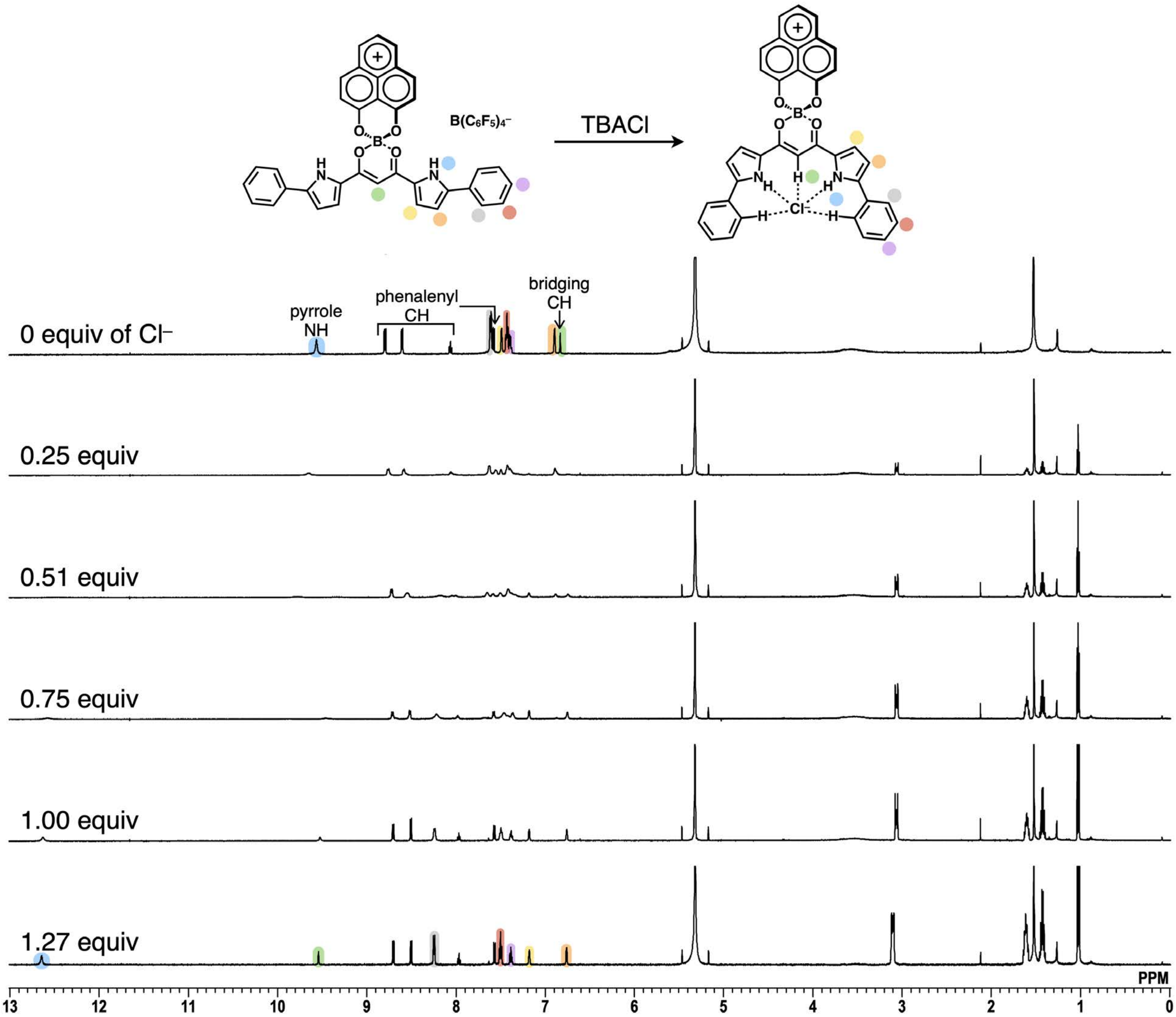

Fig. S78 ^{1}H NMR spectral change of **3b**$^+$-B(C_6F_5)$_4^-$ upon the addition of Cl$^-$ as a TBA salt at r.t. in CD_2Cl_2 (0.5 mM). Upon the addition of Cl$^-$ as a TBA salt, the pyrrole NH and bridging CH signals disappeared, and signals corresponding to the Cl$^-$ complex appeared.

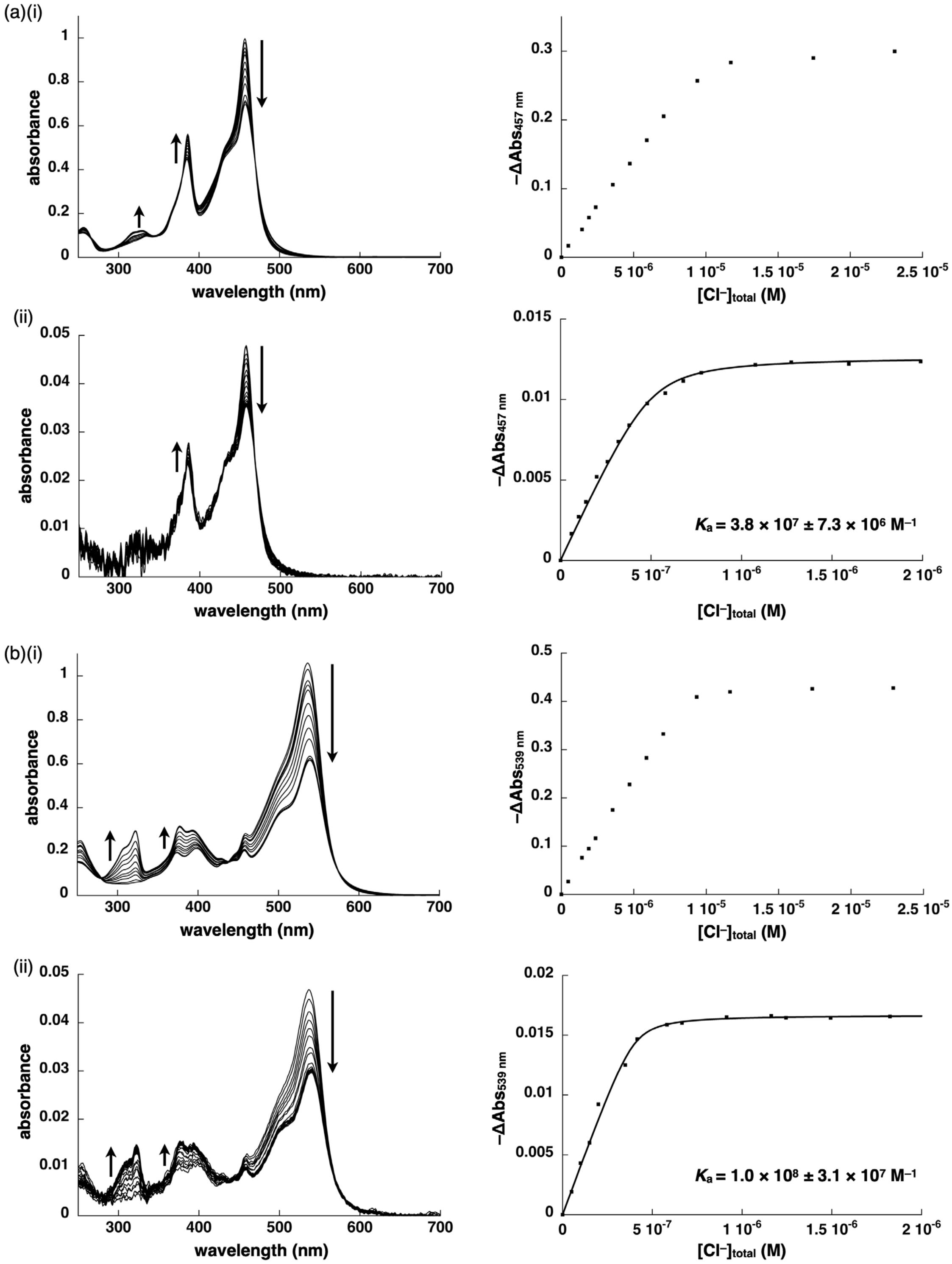

Fig. S79 UV/vis absorption spectral changes (left) and corresponding titration plots and 1:1 fitting curves (right) of (a) **3a**$^+$-B$(C_6F_5)_4^-$ and (b) **3b**$^+$-B$(C_6F_5)_4^-$ ((i) 1.2×10^{-6} M and (ii) 5.0×10^{-7} M) upon the addition of Cl^- as a TBA salt in CH_2Cl_2. The binding constants (K_a) were evaluated under the dilute conditions.

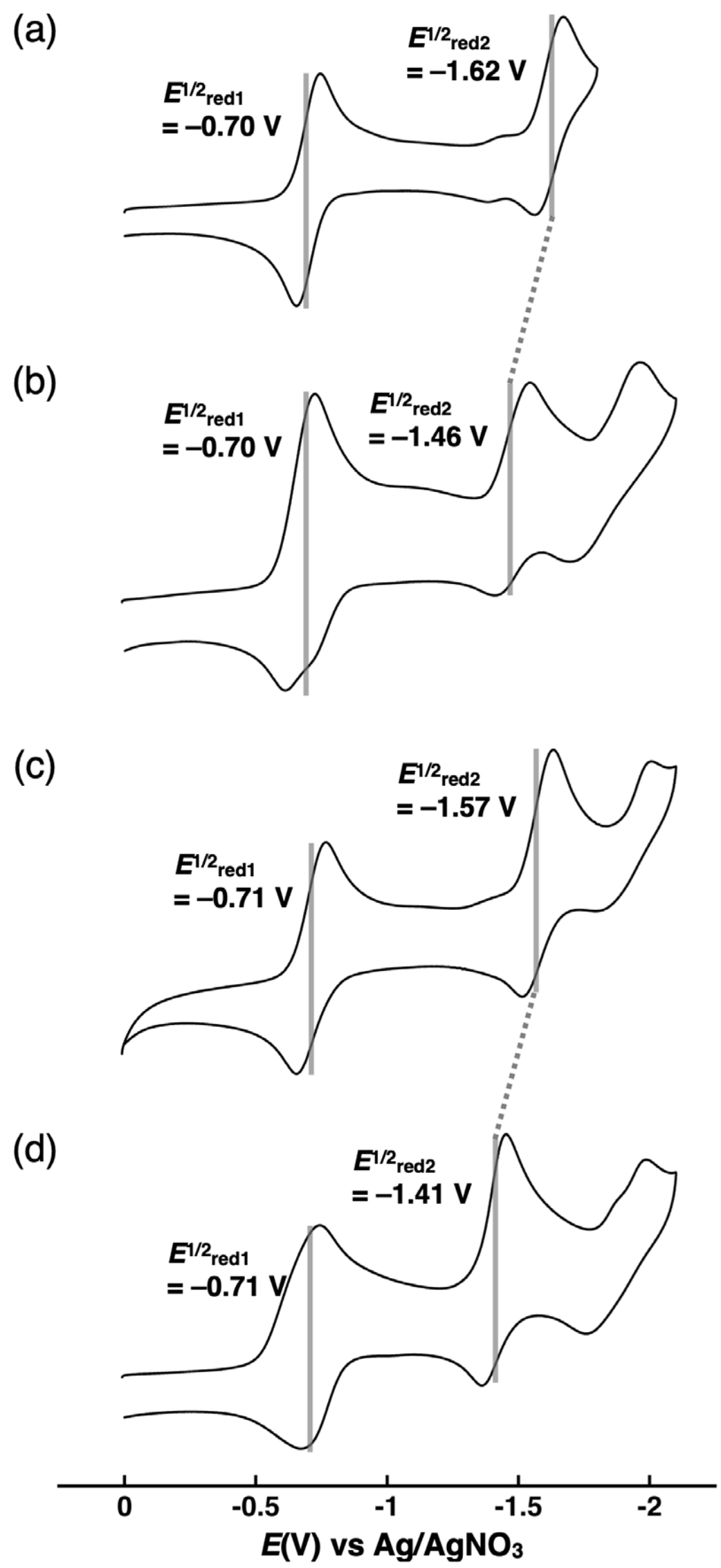

Fig. S80 Cyclic voltammograms (CVs) of (a) **3a**$^{+}$-Cl^{-}, (b) **3a**$^{+}$-PF_6^{-}, (c) **3b**$^{+}$-Cl^{-}, and (d) **3b**$^{+}$-PF_6^{-} in CH_2Cl_2 (2.5×10^{-4} M) containing TBACl (0.1 M) as an electrolyte for the Cl^{-} ion pairs and $TBAPF_6$ (0.1 M) as an electrolyte for the PF_6^{-} ion pairs at a scan rate of 100 mV/s. The first reduction potential did not change in either spectrum, indicating that it is derived from phenalenyl unit. This is consistent with the localization of the LUMO on the phenalenyl unit. On the other hand, the second reduction potential shifts to a lower potential with Cl^{-} binding, suggesting that it is derived from the dipyrrolyldiketone units. Comparison between **3a**$^{+}$ and **3b**$^{+}$ reveals an increase in electron-accepting capacity due to π-extension in **3b**$^{+}$. Ambiguous reversibility of the voltammograms, particularly for **3a** ion pairs, may arise from the formation of small amounts of decomposed species.

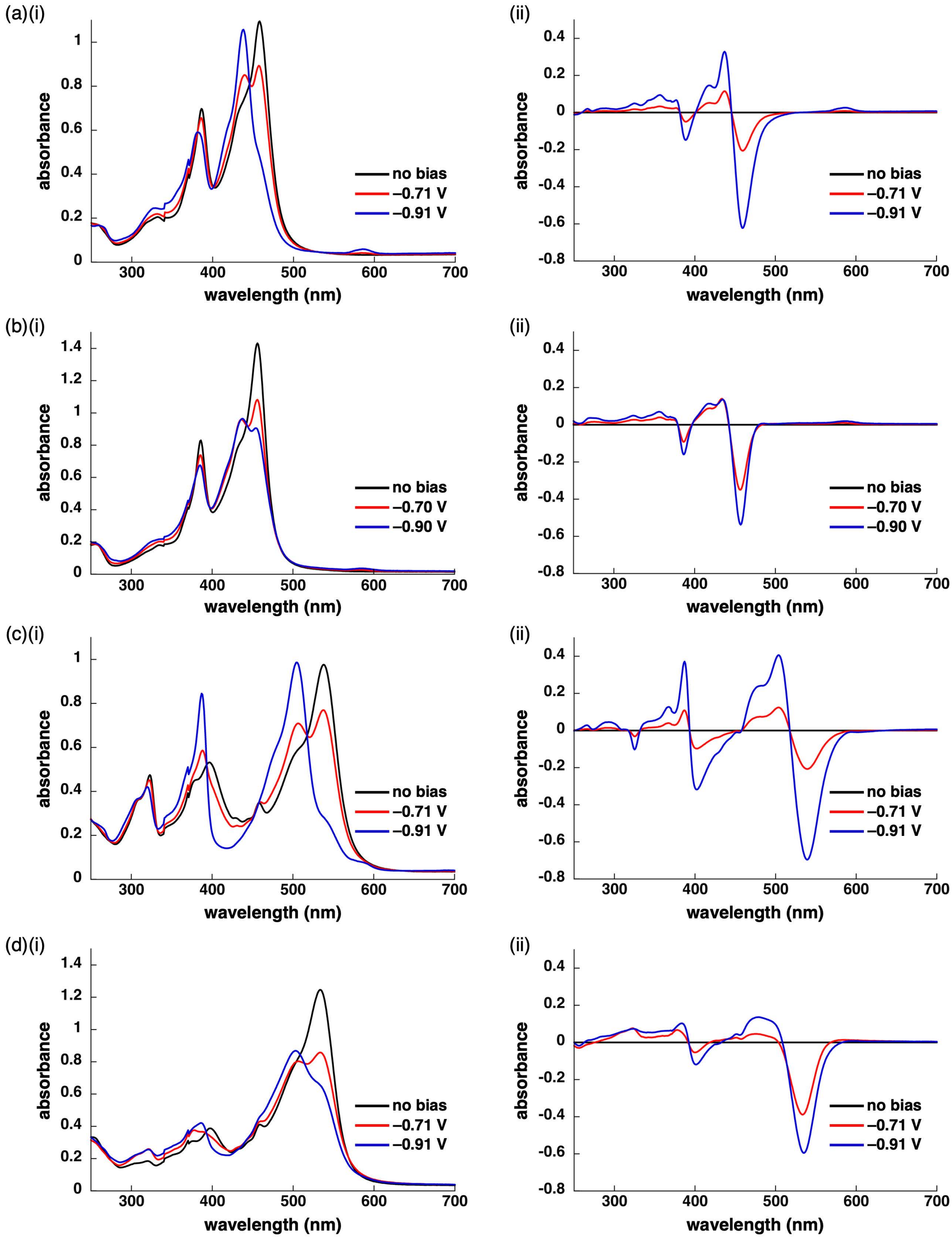

Fig. S81 Spectroelectrochemical analysis of (a) **3a**$^+$-Cl$^-$, (b) **3a**$^+$-PF$_6^-$, (c) **3b**$^+$-Cl$^-$, and (d) **3b**$^+$-PF$_6^-$ for the oxidation states at –0.70/–0.71 V (red) and –0.90/–0.91 V (blue) in CH_2Cl_2 (1.5×10^{-4} M) containing 0.1 M TBACl in (a,c) and $TBAPF_6$ in (b,d) as electrolytes under Ar atmosphere: (i) UV/vis absorption spectral changes and (ii) difference spectra between no bias and applied bias. At –0.71 and –0.91 V for (a,c,d) and –0.70 and –0.90 V for (b), the molar ratios of ground-state species and the corresponding reduced species are supposed to be 50:50 and <1:>99, respectively. Decreased absorption at the λ_{max} (459 nm) and increased absorption at 436 nm with the slightly enhanced absorbance at 585 nm in **3a**$^+$-PF$_6^-$ suggested the reduction of phenalenyl unit influenced the electronic state of the dipyrrolyldiketone unit via the bridging boron. TD-DFT calculations at CPCM-CAM-B3LYP/6-31++G(d,p) (CH_2Cl_2) for one-electron reduced state of **3a**$^+$-Cl$^-$ supported blue shift of λ_{max} with slightly enhanced absorbance at 548 nm (Fig. S57a,62a). **3b**$^+$-Cl$^-$ ion pair also exhibited similar behaviors (Fig. S57b,62b).

5. Transient absorption spectroscopic data

Method for sub-picosecond to nanosecond transient absorption measurements. Transient absorption measurements on the sub-picosecond to nanosecond timescale were conducted by a homemade pump-probe system. An amplified femtosecond laser, Spirit One 1040-8 (Spectra-Physics, 1040 nm, the pulse width: ~270 fs), was split into two beams with a ratio of 1:9. The stronger beam was directed to a noncollinear optical parametric amplifier (NOPA), Spirit-NOPA-3H (Spectra-Physics) to generate the 380-nm femtosecond laser pulse for the pump beam. The pump beam was chopped prior to the sample at 500 Hz for signal differencing. The other weaker beam was focused on deuterated water placed in a 10-mm quartz cuvette to generate the white light continuum for the probe beam. Both pump and probe beams were focused on the sample solution placed in the 2-mm quartz cuvette. The polarization between the pump and probe pulses was set at the magic angle. The intensity of the pump pulse was set to 28, 30, and 32 nJ/pulse. The transmitted probe beam was detected with multichannel detection system, PK120-C-RK (UNISOKU), composed of a CMOS linear image sensor and a polychromator. The instrumental response function was shorter than approximately 100 fs. The sample solution was stirred with a stirrer during the experiments. All the measurements were performed at r.t. The obtained transient absorption spectra were globally analyzed using the four-state sequential decay kinetic models convolved with a Gaussian pulse using the Glotaran program.[S14] The obtained data were analyzed using global analysis based on a sequential two- or three-state kinetic model, which enabled the decomposition of the spectra into evolution-associated spectra (EAS).

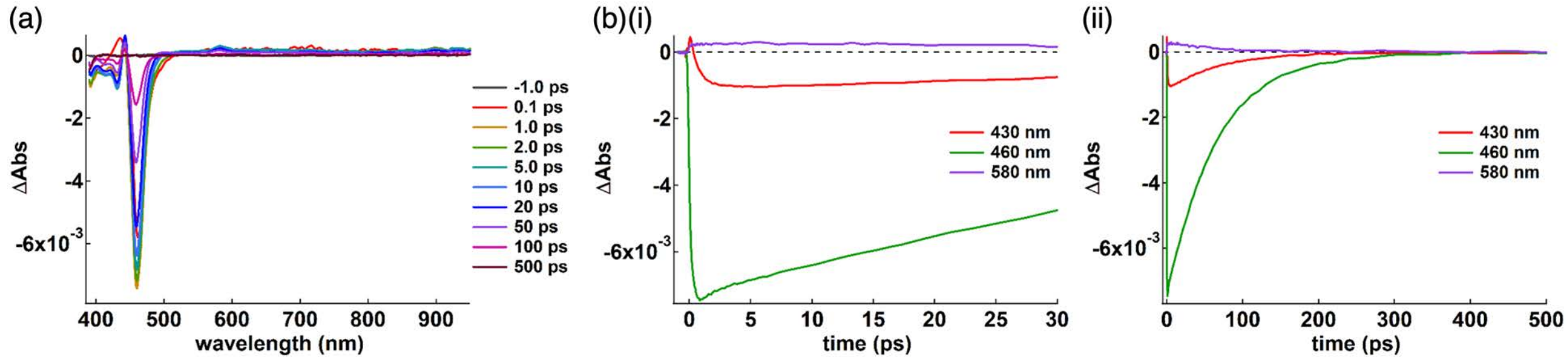

Fig. S82 (a) Transient absorption spectra and (b) dynamics of **3a**$^+$-$B(C_6F_5)_4^-$ in CH_2Cl_2 excited at 380 nm (30 nJ/pulse).

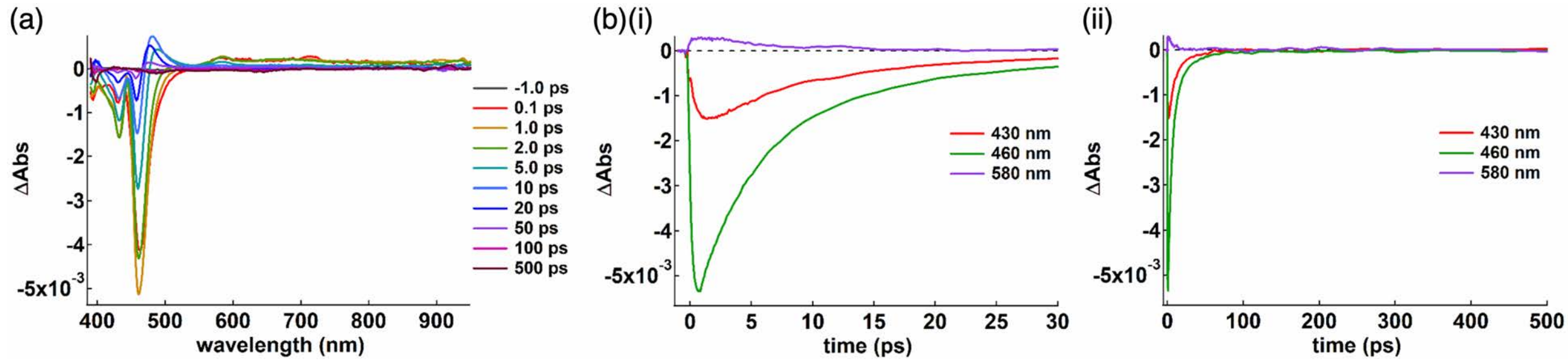

Fig. S83 (a) Transient absorption spectra and (b) dynamics of **3a**$^+$-Cl^-, formed upon the addition of Cl^- as a TBA salt (2.0 equiv) to **3a**$^+$-$B(C_6F_5)_4^-$, in CH_2Cl_2 excited at 380 nm (28 nJ/pulse).

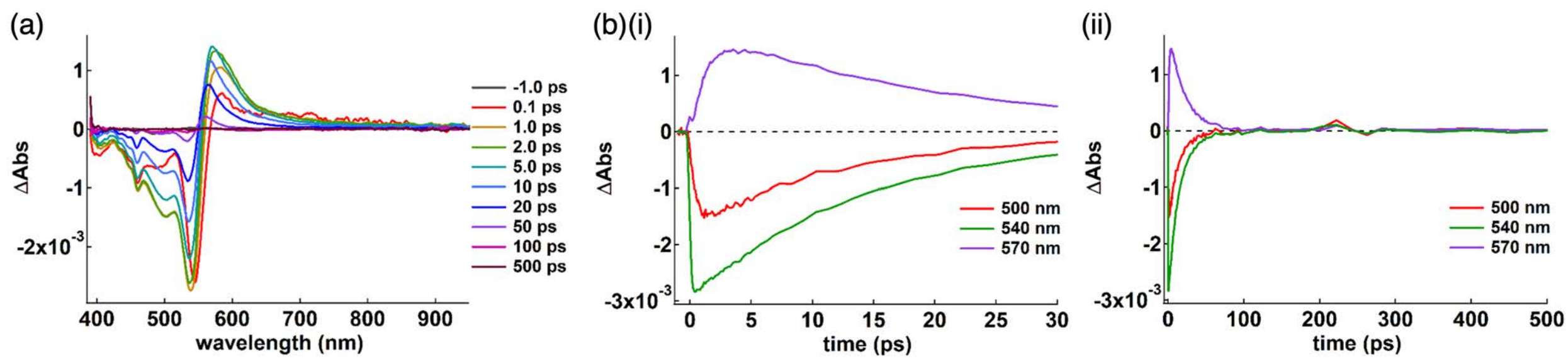

Fig. S84 (a) Transient absorption spectra and (b) dynamics of **3b**$^+$-$B(C_6F_5)_4^-$ in CH_2Cl_2 excited at 380 nm (32 nJ/pulse).

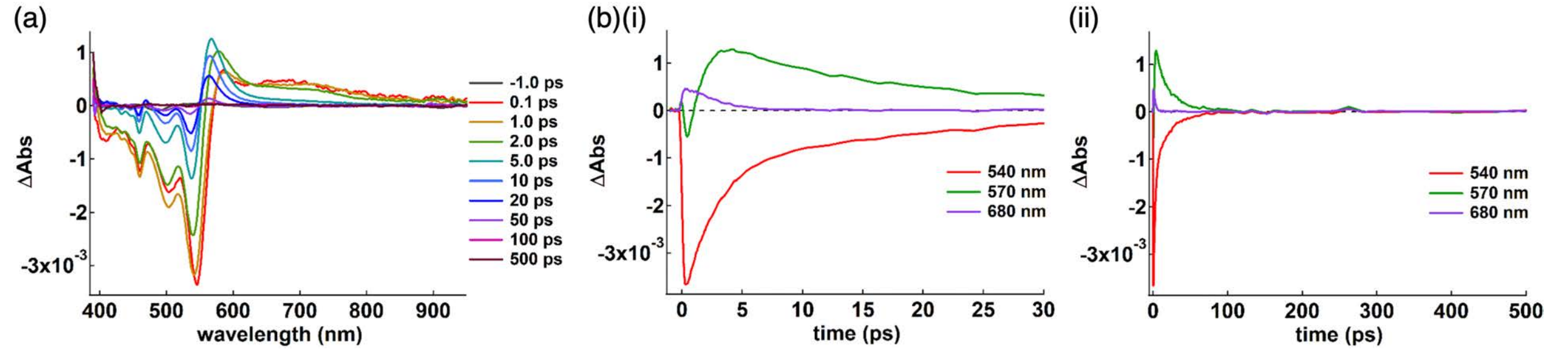

Fig. S85 (a) Transient absorption spectra and (b) dynamics of **3b**$^{+}$-Cl^{-}, formed upon the addition of Cl^{-} as a TBA salt (2.0 equiv) to **3b**$^{+}$-$B(C_6F_5)_4^{-}$, in CH_2Cl_2 excited at 380 nm (30 nJ/pulse).

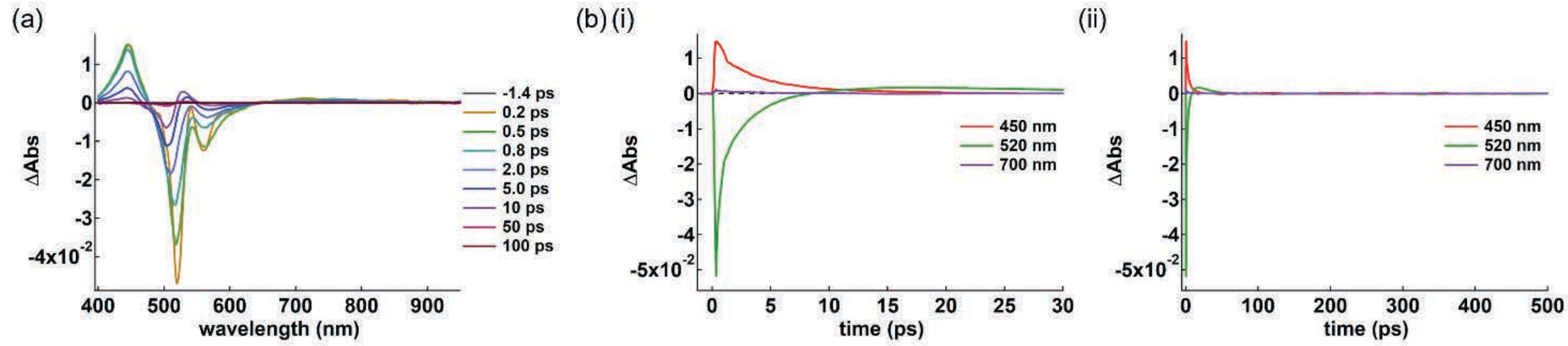

Fig. S86 (a) Transient absorption spectra and (b) dynamics of **1b** in CH_2Cl_2 excited at 520 nm (40 nJ/pulse).

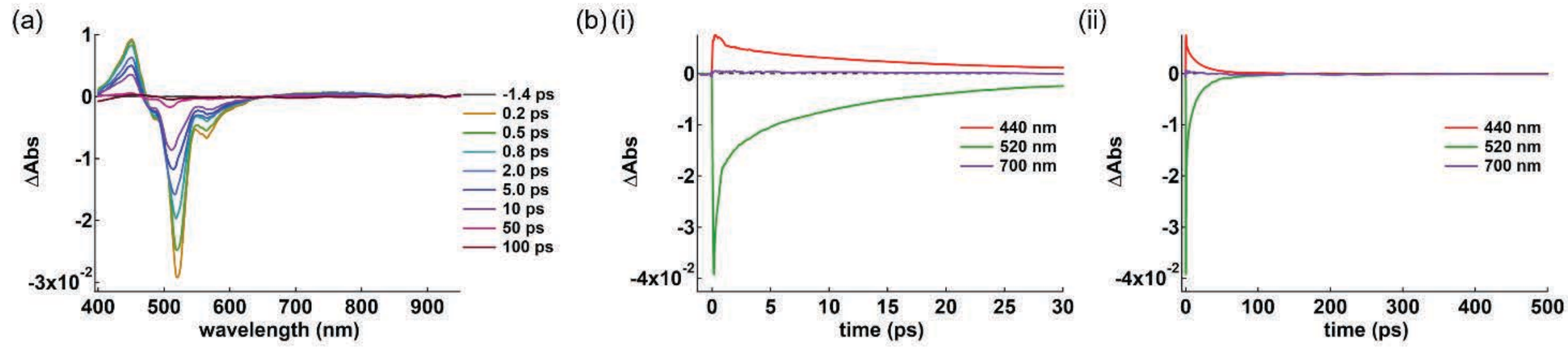

Fig. S87 (a) Transient absorption spectra and (b) dynamics of **1b**·Cl^{-}, formed upon the addition of Cl^{-} as a TBA salt (5000 equiv) to **1b**, in CH_2Cl_2 excited at 520 nm (40 nJ/pulse).

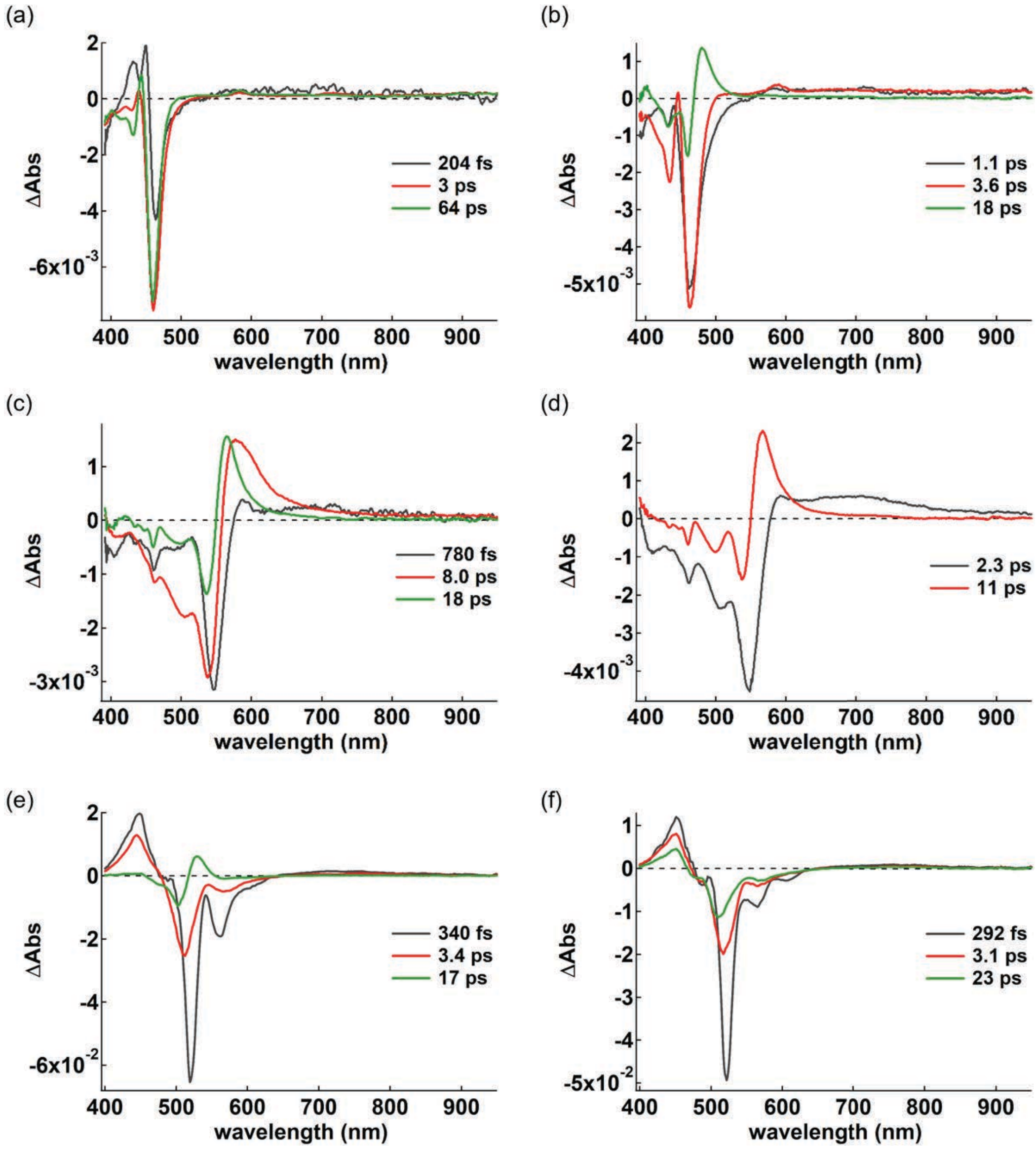

Fig. S88 Evolution-associated spectra (EAS) of the transient absorption spectra of (a) **3a**$^+$-B$(C_6F_5)_4^-$, (b) **3a**$^+$-Cl^-, (c) **3b**$^+$-B$(C_6F_5)_4^-$, and (d) **3b**$^+$-Cl^- in CH_2Cl_2 excited with a 380 nm femtosecond laser pulse and (e) **1b** and (f) **1b**·Cl^- in CH_2Cl_2 excited with a 520 nm femtosecond laser pulse. The Cl^- complexes were prepared according to the procedure described in the captions of Fig. S83,85,87.

[S14] J. J. Snellenburg, S. P. Laptenok, R. Seger, K. M. Mullen and I. H. M. van Stokkum, *J. Stat. Softw.*, 2012, **49**, 1–22.

6. Pressure-dependent UV/vis absorption spectra

Hydrostatic pressure spectroscopy. UV/vis absorption spectra under hydrostatic pressure were acquired using a JASCO V-770 spectrometer equipped with a custom-built high-pressure apparatus, as described in our previous publications.[S15] Briefly, the sample solution was loaded into a quartz inner cell with a 2-mm path length connected to a Teflon tube. The inner cell was placed in an outer pressure cell filled with H_2O as the pressure-transmitting medium. Hydrostatic pressure was applied using a hand pump, and spectra were collected over the pressure range of 0.1–280 MPa through sapphire windows.

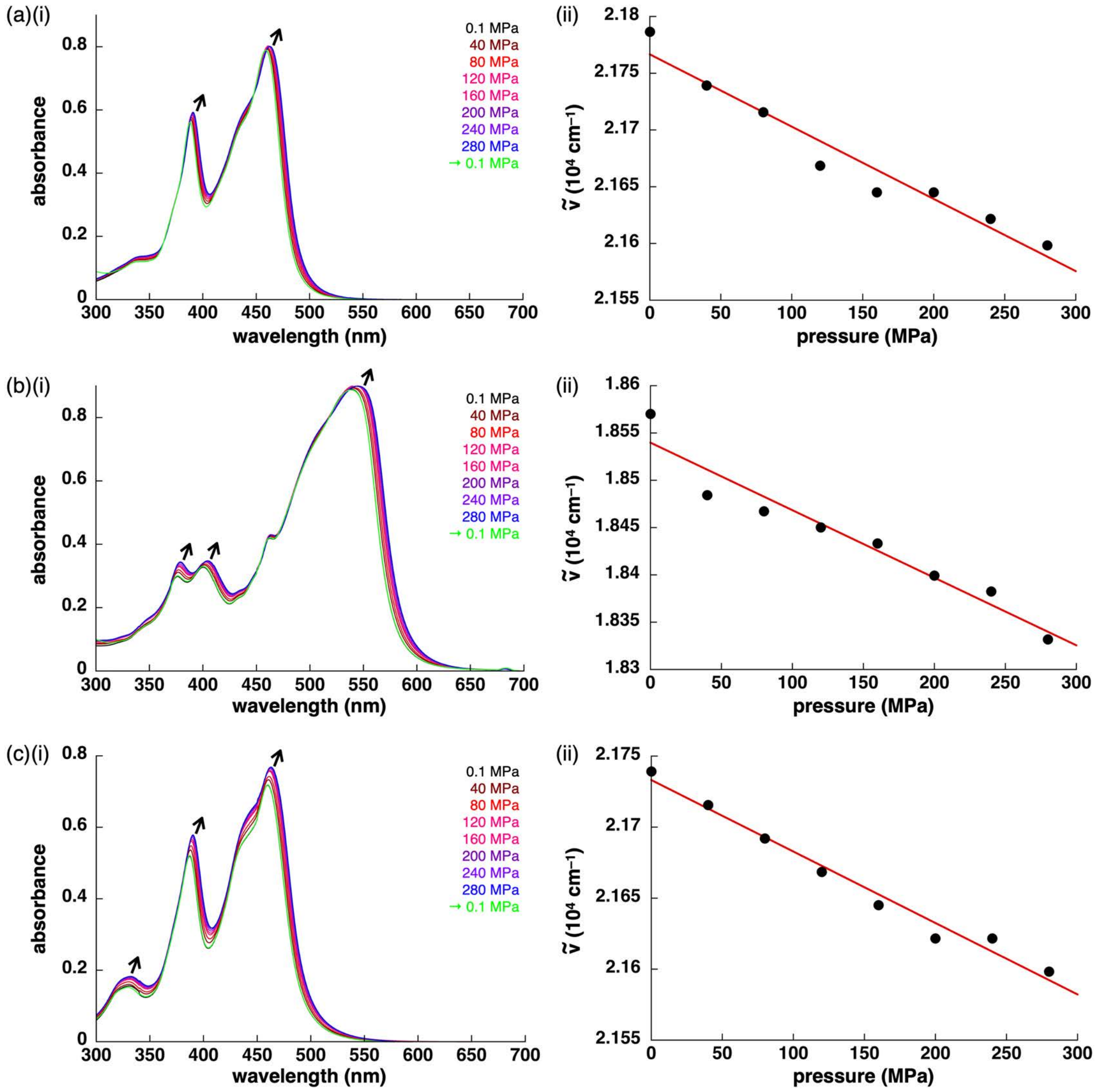

Fig. S89 (i) Pressure-dependent UV/vis absorption spectra and (ii) plots of wavenumber changes for the absorption maxima of (a) **3a**$^+$-B(C_6F_5)$_4^-$ in CH_2Cl_2 (74.6 μM, r = 0.974, slope; –0.637 cm^{-1} MPa^{-1}), (b) **3b**$^+$-B(C_6F_5)$_4^-$ in CH_2Cl_2 (86.2 μM, r = 0.969, slope; –0.714 cm^{-1} MPa^{-1}), (c) **3a**$^+$-Cl$^-$ in CH_2Cl_2 (87.1 μM, r = 0.990, slope; –0.503 cm^{-1} MPa^{-1}), and (d) **3b**$^+$-Cl$^-$ in CH_2Cl_2 (68.0 μM, r = 0.996, slope; –0.616 cm^{-1} MPa^{-1}) at r.t. under the pressures at 0.1, 40, 80, 120, 160, 200, 240, and 280 MPa (260 MPa for **3b**$^+$-Cl$^-$), measured in a high-pressure cell. The green lines represent the spectra at 0.1 MPa depressurized from 280 or 260 MPa. Upon elevated hydrostatic pressures, **3a**$^+$-B(C_6F_5)$_4^-$ and **3b**$^+$-B(C_6F_5)$_4^-$ exhibited reversible red shifts with increased absorbances in the UV/vis absorption spectra. The red shifts are attributed to pressure-induced changes in solvent density and polarizability,[S16] whereas the increased absorption intensity reflects an effective concentration increase.[S17] Plots of the absorption maxima as a function of applied pressure exhibited a larger slope for **3b**$^+$-B(C_6F_5)$_4^-$ (–0.714 cm^{-1} MPa^{-1}) than that for **3a**$^+$-B(C_6F_5)$_4^-$ (–0.637 cm^{-1} MPa^{-1}), indicating a slightly stronger pressure response for the former. Dipole moments of 5.53 and 8.35 D for **3a**$^+$ and **3b**$^+$, respectively, as calculated by CPCM-CAM-B3LYP/6-31++G(d,p) (CH_2Cl_2) may be correlated with the pressure-induced spectral changes due to the increased dielectric constants under high-pressure conditions. The slopes for **3a**$^+$-Cl$^-$ and **3b**$^+$-Cl$^-$ were –0.503 and –0.616 cm^{-1} MPa^{-1}, respectively, which are smaller than those observed for the corresponding B(C_6F_5)$_4^-$ ion pairs. The smaller pressure response of the Cl$^-$ ion pairs is attributed to the structural rigidification caused by Cl$^-$ binding.

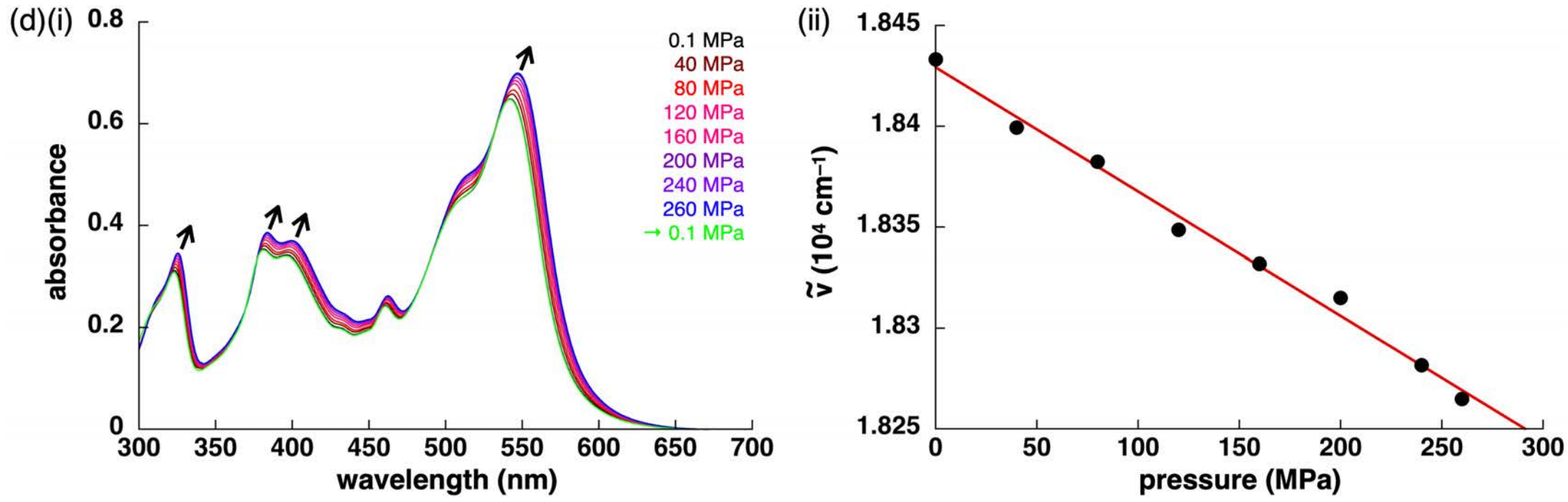

Fig. S89 (Continued)

[S15] (a) H. Mizuno and G. Fukuhara, *Acc*. *Chem*. *Res*., 2022, **55**, 1748–1762; (b) H. Mizuno, M. Kitamatsu, Y. Imai and G. Fukuhara, *ChemPhotoChem*, 2020, **4**, 502–507.
[S16] (a) F. A. Bovey and S. S. Yanari, *Nature*, 1960, **186**, 1042–1044; (b) T. Takagi and H. Teranishi, *J. Chem. Eng. Data*, 1982, **27**, 16–18.
[S17] W. W. Robertson, *J. Chem. Phys.*, 1960, **33**, 362–365.
